# Ultra‐High‐Throughput Discovery of Multifunctional Polyphenolic Coatings on Droplet Microarrays

**DOI:** 10.1002/adma.73612

**Published:** 2026-06-11

**Authors:** Vania Tanda Widyaya, Joaquín E. Urrutia Gómez, Paul Reuß, Alexander Welle, Rolf A. Gattung, Jana Mayer, Peter Krolla, Markus Reischl, Pascal Friederich, Anna A. Popova, Thomas Schwartz, Pavel A. Levkin

**Affiliations:** ^1^ Institute of Biological and Chemical Systems‐Functional Molecular Systems (IBCS‐FMS) Karlsruhe Institute of Technology (KIT) Eggenstein‐Leopoldshafen Germany; ^2^ Institute of Automation and Applied Informatics (IAI) Karlsruhe Institute of Technology (KIT) Eggenstein‐Leopoldshafen Germany; ^3^ Institute of Functional Interfaces (IFG) Karlsruhe Institute of Technology (KIT) Eggenstein‐Leopoldshafen Germany; ^4^ Karlsruhe Nano Micro Facility (KNMF) Karlsruhe Institute of Technology (KIT) Eggenstein‐Leopoldshafen Germany; ^5^ Institute of Nanotechnology (INT) Karlsruhe Institute of Technology (KIT) Eggenstein‐Leopoldshafen Germany; ^6^ Institute of Theoretical Informatics (ITI) Karlsruhe Institute of Technology (KIT) Karlsruhe Germany; ^7^ Institute of Organic Chemistry (IOC) Karlsruhe Institute of Technology (KIT) Karlsruhe Germany

**Keywords:** antimicrobial resistance, droplet microarray technology, functional coatings, plant polyphenols, polyamine‐polyphenolic coatings, surface chemistry, surface functionalization, ultra‐high‐throughput combinatorial synthesis, ultra‐high‐throughput screening

## Abstract

The rapid discovery of functional coatings is vital for advancing technologies in healthcare, energy, and environmental protection, yet it remains limited by the lack of scalable high‐throughput (HT) methods. Here, an ultra‐high‐throughput (UHT) combinatorial strategy is introduced for the miniaturized synthesis and screening of polyamine‐polyphenolic (**PaPp**) coatings (formed in Tris buffer) using droplet microarrays (DMA). Approximately 30 000 coatings were generated from binary and ternary combinations of 51 polyphenols (**Pp**) and 12 polyamines (**Pa**), each produced in 160 nL volumes (<5 mL total reagent use), enabling the rapid identification of hundreds of previously unknown functional materials, including over 225 fluorescent coatings and more than 100 redox‐active, metal‐reducing surfaces. Antibacterial screening uncovered seven coatings with reproducible activity against *Pseudomonas aeruginosa*, and subsequent validation on macroscale substrates confirmed up to <1‐log colony‐forming unit (CFU) reduction against *P. aeruginosa* and *E. coli*. Importantly, five coatings exhibited multiple functionalities, combining surface stability, intrinsic fluorescence, metal‐reducing activity, antibacterial effects, and compatibility with adherent human cells. This UHT approach yields the first comprehensive functionality map of **PaPp** coatings, revealing previously inaccessible multifunctional materials and demonstrating a scalable strategy for the discovery of surface chemistries with tailored optical, redox, and biological properties.

## Graphical abstract

An ultra‐high‐throughput (UHT) combinatorial strategy enables the miniaturized synthesis and screening of ≈30 000 polyamine‐polyphenolic (**PaPp**) coatings using droplet microarrays (DMA). This approach reveals hundreds of previously unknown fluorescent, redox‐active, and antibacterial materials, including multifunctional, cell‐compatible surfaces, establishing a scalable platform for rapidly discovering advanced coatings with tailored optical, chemical, and biological functionalities.

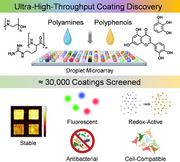

## 1 Introduction

Surface coatings play a crucial role across industries, enhancing protection and performance in applications ranging from anticorrosive automotive finishes [1, 2] and flame‐retardant aerospace coatings [3] to food packaging [4] and medical devices that ensure sterility and prevent contamination [5, 6]. Coatings are typically based on polymers [7, 8], metals [9, 10], or ceramics [11, 12], with polymer coatings dominating the market owing to their versatility, ease of processing, and cost efficiency [13].

Naturally derived plant polyphenols (**Pp**) have recently emerged as attractive coating precursors, as their abundant phenolic hydroxy groups enable covalent reactions including radical coupling, Michael addition, and Schiff base formation, as well as non‐covalent interactions such as hydrogen bonding, *π*–*π* stacking, and electrostatic attraction with diverse substrates [14, 15, 16]. Coatings composed solely of **Pp**, formed from tannic acid, pyrogallol, epicatechin gallate, epigallocatechin gallate, and crude extracts of red wine, cacao beans, and green tea without additional components, were first reported in 2013 by the Messersmith group [17]. More than 8000 **Pp** have been identified to date [18, 19], offering an extensive, largely untapped resource for discovering coatings with diverse functionalities. **Pp** display exceptional chemical and biological activities, including metal reduction and chelation [20, 21], fluorescence [22, 23], self‐assembly [24, 25], antioxidant [26, 27] and antimicrobial activities [28, 29], anticancer effects [30, 31], and biocompatibility [32, 33], among others. Their potential for synergistic and antagonistic interactions further expands the design landscape [34, 35]. A binary combination of 8000 compounds alone will result in ≈3.2 × 10^7^ unique pairs (combination calculation is provided in Section S1).

Despite their advantages, **Pp** suffer from poor intrinsic stability, which limits their use as coatings [36, 37]. To address this limitation, researchers have conjugated **Pp** with polymers [38, 39], hydrogels [40, 41], biomolecules [42, 43], or metals [44, 45]. Among these strategies, polyamines (**Pa**) are particularly attractive owing to their inherent anticorrosive [46, 47], adhesive [48, 49, 50], and antimicrobial properties [51, 52, 53]. Crosslinking **Pa** with **Pp** can yield robust multifunctional polyamine‐polyphenolic (**PaPp**) coatings. Phenol‐amine chemistry has primarily been utilized to formulate adhesives [54, 55], especially those based on tannic acid [54, 56] and dopamine [55, 57], leaving a vast range of **PaPp** compositions unexplored. Only a handful of studies have investigated **PaPp**‐based materials or coatings, demonstrating properties such as antimicrobial activity [58, 59], antifouling behavior [60, 61], anticancer activity [62], biocompatibility [62, 63], and fluorescence [23]. The crosslinking between **Pa** and **Pp** proceeds via oxidation of phenolic groups to phenolquinones, followed by reaction with amine groups of **Pa**, primarily through Michael addition and/or Schiff base reactions [14, 15, 35]. Oxidation is efficiently triggered by atmospheric oxygen, enabling spontaneous coating formation at air‐liquid or air‐solid interfaces under ambient conditions [64, 65]. It has been reported that ultraviolet (UV) irradiation [35, 66] and/or mild alkaline conditions [15, 35] could accelerate and ensure the completion of the process [35, 66].

Given the enormous structural diversity of **Pp** and the growing demand for functional coatings, there is a compelling need for high‐throughput (HT) platforms capable of rapidly generating and screening large combinatorial libraries. This need is particularly acute in the context of antimicrobial resistance [67, 68], where the evolution of resistance mechanisms and the spread of antibiotic‐resistant bacteria are outpacing the discovery of effective solutions such as new active materials or antibiofilm surface designs [69, 70]. This underscores the importance of HT approaches to identify effective antimicrobial materials.

Over the past decade, the droplet microarray (DMA) platform has demonstrated exceptional capability for miniaturized HT synthesis and screening [71, 72, 73]. DMA features high‐density arrays of hydrophilic spots separated by superhydrophobic borders on microscope glass slides, enabling compartmentalized sub‐microliter reactions [72, 74, 75]. It has successfully facilitated chemical reactions, including Suzuki‐Miyaura reaction [75], Ugi reaction [71], and hydrogel synthesis [73], as well as biological studies such as drug‐induced gene expression [72], messenger ribonucleic acid isolation [76], and 3D cell spheroid formation [77]. Compared to microplate‐based HT methods, DMA offers significant advantages: (i) the open planar design facilitates oxygen access, crucial for phenol oxidation; (ii) an efficient confinement of nanoliter droplets without capillary effects caused by physicals wells [78]; (iii) DMA is fabricated using photolithography and photochemical surface functionalization, allowing the formation of thousands of hydrophilic spots on a single microscope glass slide, thus offering higher throughput while minimizing reagent consumption, plastic waste, and cost [78]; (iv) its compatibility with a wide range of analytical tools, including scanning electron microscopy [75, 79], atomic force microscopy [80], contact‐angle goniometry [80, 81], time‐of‐flight secondary ion mass spectrometry (ToF‐SIMS) [80], matrix‐assisted laser desorption/ionization time‐of‐flight [80, 81], Raman and infrared spectroscopy [80], microscopy [72, 73], colorimetric and fluorescence scanning [74, 81], and automated liquid handlers [72, 74, 75]. The primary limitation of DMA is the high rate of evaporation of liquid analytes due to nanoliter volumes, which can be mitigated by humidity control during processing [72, 75]. Despite its versatility, DMA has not yet been utilized for HT synthesis and screening of surface coatings. This study presents the first demonstration of ultra‐HT (UHT) synthesis and in situ screening of coating properties using the DMA platform. Hereafter, “UHT” refers specifically to the platform, coating libraries, and screening assays developed in this work, whereas “HT” is used exclusively for general or comparative references to previously reported high‐throughput studies with lower sample density or throughput.

A relatively small number of HT studies involving **Pp** have been reported [29, 35, 82, 83, 84, 85]. The most relevant work, by Behboodi‐Sadabad et al., investigated the coating‐forming potential of a library comprising 45 phenolic compounds and catecholamines in single, binary, and ternary combinations [35]. A total of 2352 experiments (588 combinations × 4 repetitions) were conducted, leading to the identification of 30 stable, non‐toxic coatings [35]. Most other studies screened comparable or smaller libraries, often unrelated to surface coating applications [29, 82, 83, 84, 85]. Existing studies on HT coating synthesis rely largely on earlier‐generation approaches developed more than a decade ago [86, 87, 88, 89]. This gap underscores the urgent need for next‐generation HT methodologies to accelerate coating discovery.

**FIGURE 1:**
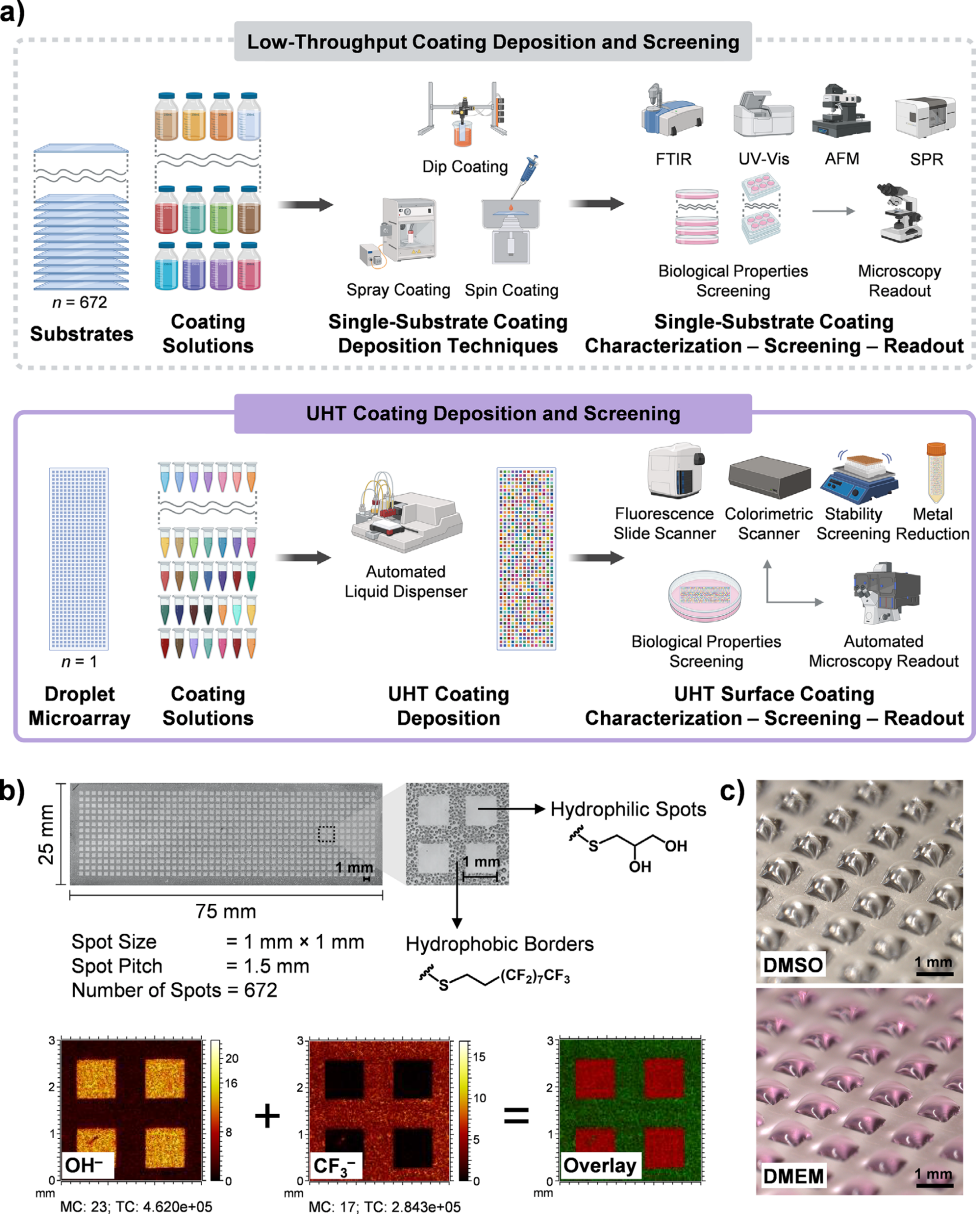
Ultra‐high‐throughput (UHT) coating deposition and screening. (a) Conceptual illustration comparing low‐throughput and UHT coating deposition and functionality screenings. The UHT method integrates miniaturized droplet microarray (DMA; each containing 672 miniaturized single‐substrates) with automated workflows. *n* = the number of substrate units. (b) Image of a scanned DMA after steam exposure, showing 672 hydrophilic spots (area = 1 mm × 1 mm; functionalized with thioglycerol), surrounded by superhydrophobic barriers (functionalized with 1H, 1H, 2H, 2H‐perfluorodecanethiol, PFDT). ToF‐SIMS imaging confirmed successful photopatterning and photochemical functionalization, evidenced by the OH^–^ signal from thioglycerol and the CF_3_^−^ signal from PFDT. The color scale represents the respective ion intensities (counts). MC = maximum ion counts per pixel. TC = total ion counts per image. (c) Microscope images of 160 nL DMSO droplets (top) and 250 nL Dulbecco's Modified Eagle Medium (DMEM, supplemented with 10% fetal bovine serum (FBS); bottom) on the DMA surface.

Here, the DMA platform combined with laboratory automation was employed for UHT combinatorial synthesis and screening of **PaPp** coatings. **PaPp** coatings were formed under ambient conditions, where air‐mediated phenol oxidation drove network formation between **Pa** and **Pp**. The term “crosslinking” is therefore used in a broad sense, referring to this oxidation‐driven network formation process, which may involve a heterogeneous combination of covalent quinone‐amine reactions as well as non‐covalent interactions, including hydrogen bonding, *π*–*π* stacking, and electrostatic interactions. All coatings in this study were formed in Tris buffer (pH 8.5) containing tris(hydroxymethyl)aminomethane, which is known to participate in reactions with oxidized **Pp** and might be incorporated into **PaPp** coatings as the third component [90, 91]. A library of 29 094 coatings (including replicates), derived from 12 **Pa** and 51 **Pp**, was fabricated within 20 days using a total of only 4.7 mL of precursor solution, an outcome unattainable by conventional approaches (Figure 1). UHT screening enabled the rapid identification of stable coatings (in both aqueous and organic environments) exhibiting diverse functionalities, including multicolor fluorescence (green, blue, red), redox activity (reduction of silver ions to silver nanoparticles), antibacterial properties, and the ability to culture adherent cells. Antibacterial screening against the strong biofilm‐forming *Pseudomonas aeruginosa* strain PA49 (PA49) yielded seven active hits that were further validated against PA49, *Escherichia coli* DSM498 (EC498), and *Staphylococcus aureus* A1 (SAA1). Colony‐forming unit (CFU) assays confirmed that two hits reduced PA49 CFU by up to ≈86%, and three hits reduced EC498 CFU by up to ≈84%. UHT cell‐based screening evaluated the coatings for their ability to support the culture of adherent human cervical cancer cells (HeLa) and provided insights into cell morphology, viability, and proliferation. In summary, these UHT approaches allowed mapping of **PaPp** functionalities across the entire coating library.

## 2 Results and Discussion

### 2.1 UHT Combinatorial Synthesis of PaPp Coatings on DMA Chips

**FIGURE 2:**
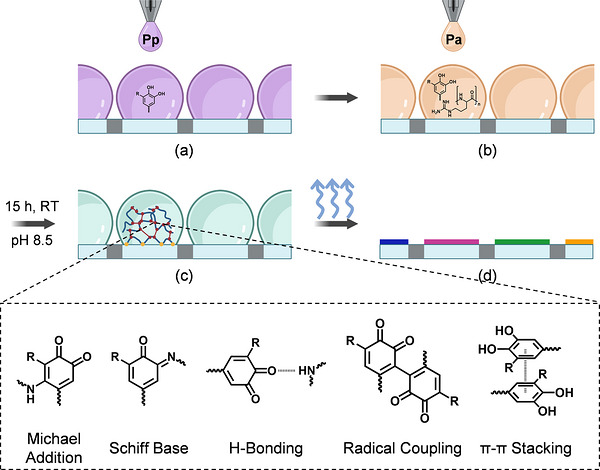
UHT combinatorial synthesis of **PaPp** coatings on DMAs. (a) Polyphenols (**Pp**) solution (80 nL, 1 mg/mL in DMSO) was deposited onto the hydrophilic spots using an automated liquid dispenser. (b) Polyamines (**Pa**) solution (80 nL, 1 mg/mL in 10 mM Tris buffer at pH 8.5) was deposited into the **Pp** solution. (c) Oxidative crosslinking between **Pa** and **Pp** proceeds through a combination of covalent and non‐covalent interactions. (d) Combinatorial **PaPp** coatings were formed on the hydrophilic spots after solvent evaporation.

The **PaPp** library was built from a selection of 12 **Pa** and 51 **Pp** compounds (the respective chemical structures can be seen in Figure 9 and Figures S1–S4). The 12 **Pa** were chosen to include previously reported antimicrobially active polymers with diverse side chains and molecular weights [51, 52, 53]. The 51 **Pp** compounds were selected based on their reported antimicrobial activity [28, 29], and to encompass low‐cost compounds with simple phenolic structures (**Pp1‐Pp4**), flavonoid structures (**Pp5‐Pp25**), and non‐flavonoid structures (**Pp26‐Pp51**). The workflow for UHT combinatorial synthesis of **PaPp** coatings on DMA chips is illustrated in Figure 2. Briefly, arrays of combinatorial 160 nL droplets containing 1:1 (v/v) mixtures of **Pa** and **Pp** were printed onto the hydrophilic spots of the DMA using an automated liquid dispenser. To minimize undesired evaporation during dispensing, **Pp** was first dissolved in dimethyl sulfoxide (DMSO), which has a high boiling point of 189°C, and deposited prior to the addition of the **Pa** solution. **Pa** was dissolved in Tris buffer (pH 8.5) to provide a mildly alkaline environment, ensuring completion of oxidative crosslinking between **Pa** and **Pp**. Following incubation at room temperature (RT) for 15 h and solvent evaporation, combinatorial **PaPp** coatings were formed on the DMA.

A key advantage of the DMA platform is the confinement of reaction droplets to the hydrophilic spots, which ensures that each coating forms only within the hydrophilic spots, independent of the reaction volume. This spatial confinement makes the coating positions predetermined and highly reproducible across the array, which is essential for UHT screening. The total number of unique **PaPp** coating combinations examined in each screening category is summarized in Table 1. In total, 29 094 coatings (including replicates) were synthesized, requiring only 112 DMA chips. For comparison, producing the same number of coatings on conventional single‐substrate units would have required 29 094 individual substrates, excluding additional uncoated units needed for experimental controls. This equates to approximately 260 times more than the DMA approach. The number of DMAs could theoretically have been reduced to 44 if all 672 spots per DMA had been fully utilized; however, specific coating layouts were designed for each screening purpose, necessitating a higher number of DMAs. The advantages of the DMA platform are further underscored by its extremely low reagent consumption and short coating library fabrication time (Table 1). Each coating required only 160 nL of precursor solution (1:1 v/v **Pa:Pp** mixture), just 0.16% of the volume typically required to spin‐coat a 1.5 cm × 1.5 cm single‐substrate (≈0.1 mL). In total, only 4.7 mL of precursor was consumed to fabricate all 29 094 coatings, compared to an estimated 3 L for the single‐substrate approach. In addition, the entire coating library was produced within 20 days, a timeframe that would be physically unfeasible using the single‐substrate format. It is estimated that producing the same number of coatings on single‐substrates would take over two years (assuming 260 workdays per year, excluding holidays).

**TABLE 1:** Comparison of precursor volumes (mL) and synthesis time (days) required for generating the **PaPp** coating library using DMA‐based UHT synthesis vs. conventional single‐substrate synthesis.

	Stability	Fluorescence Properties	Metal‐Reducing Activity	Antibacterial Activity	Compatibility with Human Cells	Total
Combinations (C)^a^	675	675	2040	1182	675	
Replicates (n)	1	6	4	8 + 4^b^	3	
Synthesized coatings (C × *n*)	675	4050	8160	14 184	2025	29 094
Required substrate units						
DMA^c^	15	8	31	54	4	112
Single‐substrate	675	4050	8160	14 184	2025	29 094
Required precursors (mL)						
DMA	0.11	0.65	1.31	2.27	0.32	4.7
Single‐substrate^d^	68	405	816	1418	203	2910
Required time (days)^e^						
DMA	5	3	12	0^f^	0^g^	20
Single‐substrate^h^	14	81	163	284	41	583

^a^ C = 675 refers to 12 **Pa** precursors, 51 **Pp** precursors, and 612 **PaPp** combinations (12 **Pa **× 51 **Pp**). C = 2040 refers to 12 **Pa** precursors, 51 **Pp** precursors, 612 **PaPp** combinations, 105 **PpPp** combinations, and 1260 **PaPpPp** combinations (12 **Pa **× 105 **PpPp**). C = 1182 refers to 12 **Pa** precursors, 30 **Pp** precursors, 360 **PaPp** combinations (12 **Pa** × 30 **Pp**), 60 **PpPp** combinations, and 720 **PaPpPp** combinations (12 **Pa** × 60 **PpPp**).**PpPp** refers to binary mixtures of **Pp** compounds in a 1:1 v/v ratio.

^b^ Used as background controls.

^c^ Number of DMAs depended on the specific layout for each assay.

^d^ A single‐substrate corresponds to a 1.5 cm × 1.5 cm piece, assuming 0.1 mL precursor volume for spin coating.

^e^ Estimation includes handling, precursor preparation, and two‐step washing (in water and ethanol), assuming an 8 h working day.

^f^ Fabricated in parallel with coatings for metal reduction screening.

^g^ Fabricated in parallel with coatings for fluorescence screening.

^h^ Assumes 50 coatings can be produced per day.

### 2.2 UHT PaPp Coating Stability Screening

The long‐term performance and practical application of surface coatings largely depend on their stability. However, many functional coatings tend to detach from substrates when exposed to aqueous or organic environments, temperature fluctuations, or mechanical stress. Polyphenolic coatings are renowned for their ability to deposit on a wide range of materials, including metals, oxides, polymers, and glass, owing to their ability to engage in multiple molecular interactions such as hydrogen bonding, *π*–*π* stacking, and metal‐ligand coordination of phenolic groups [16, 17]. This inherent, substrate‐independent deposition behavior represents one of their most appealing characteristics; however, the resulting stability and adhesion strength can vary substantially across different polyphenolic chemistries. Therefore, systematic evaluation of coating stability under various conditions is crucial for identifying robust materials. Yet, conventional stability assays are typically low‐throughput, assessing one coating at a time and making cross‐comparisons challenging. Here, the DMA platform enables UHT stability screening, allowing thousands of miniaturized coatings to be simultaneously exposed and analyzed under uniform conditions. This strategy offers a rapid and reproducible means to map the stability of **PaPp** coatings across a large combinatorial library.

**FIGURE 3:**
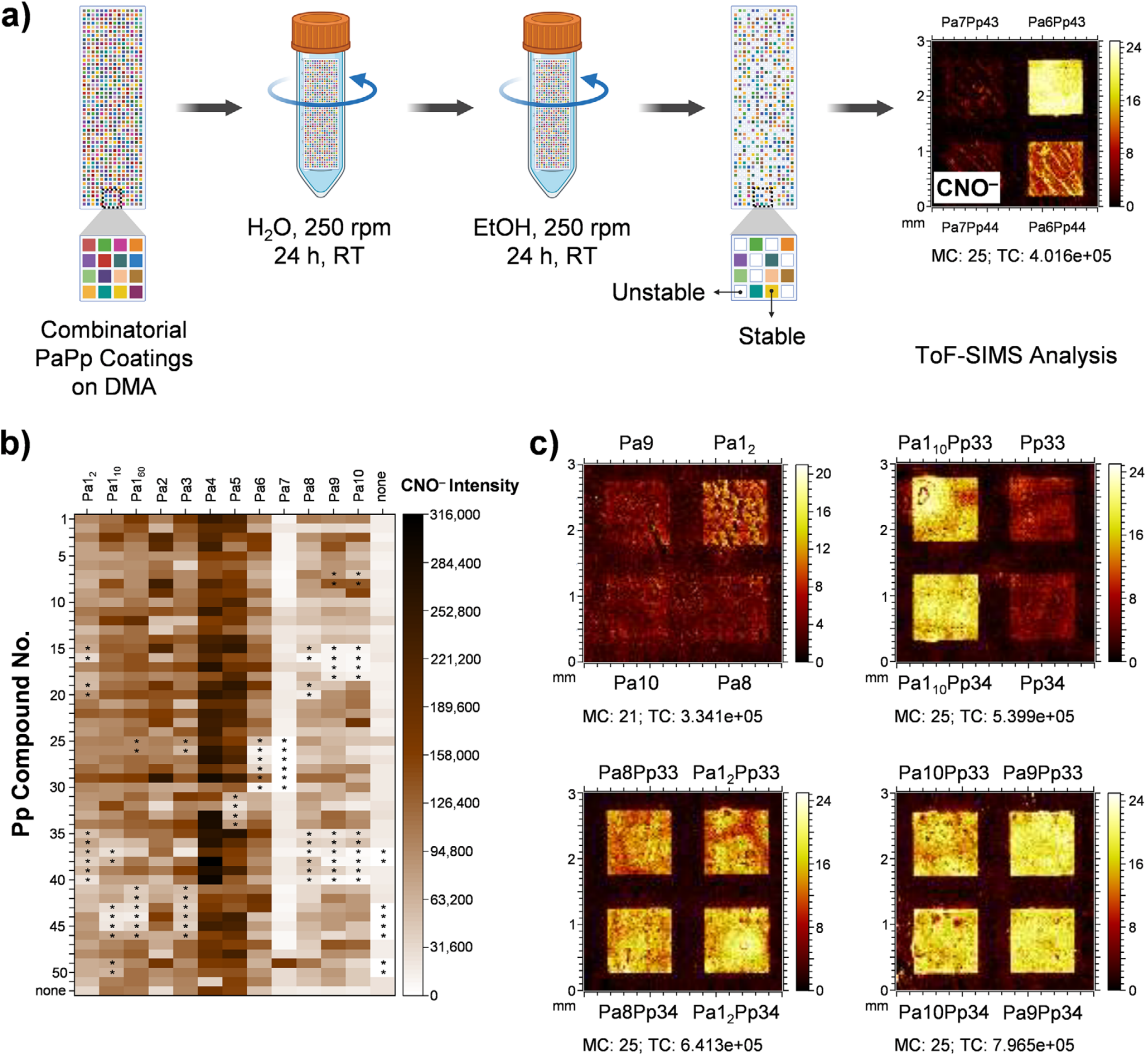
UHT stability screening of **PaPp** coatings. (a) Workflow of UHT coating stability screening. DMA chips carrying **PaPp** coatings were thoroughly washed with water and ethanol, resulting in stable **PaPp** coatings, confirmed by ToF‐SIMS analysis (CNO^−^ signal). (b) Heatmap of CNO^−^ intensities for all 612 **PaPp** combinations (12 **Pa **× 51 **Pp**) and their respective precursors, illustrating coating stability across the library. Measurements were performed on ITO‐coated DMAs, except for coatings marked with an asterisk (*), which were measured on standard DMAs. (c) Representative ToF‐SIMS images showing CNO^−^ signals confirming the presence of stable coatings. The CNO^−^ signals of precursors **Pa8**, **Pa9**, **Pa10**, **Pp33**, and **Pp34** were not detectable, whereas the corresponding **PaPp** coatings exhibited high signal intensities, indicating enhanced stability through crosslinking. Precursor **Pa1_2_** showed a measurable CNO^−^ signal, suggesting its inherent ability to form a stable coating independently. Notably, its crosslinked variants **Pa1_2_Pp33** and **Pa1_2_Pp34** showed even higher intensities. The color scale represents CNO^−^ ion counts. MC = maximum counts in a single pixel. TC = total counts per image.

In this study, the stability of **PaPp** coatings in aqueous and organic environments was evaluated by immersing the coated DMAs in water and ethanol for 24 h each, followed by ToF‐SIMS analysis (Figure 3a). One replicate of 675 unique coatings was distributed across 15 DMAs to facilitate handling (Table 1). This library was composed of 12 **Pa** precursors, 51 **Pp** precursors, and 612 **PaPp** combinations (12 **Pa **× 51 **Pp**). Stable coatings were identified by the presence of CNO^−^ secondary ion signal, which reflects the spatial proximity of nitrogen‐oxygen and serves as an indicator of crosslinking between **Pa** and **Pp**. ToF‐SIMS images of all tested coatings are presented in Figures S5–S16 and visualized as a heatmap (Figure 3b).

The results revealed that **Pp** alone could not form stable coatings, as the films readily detached upon washing. This suggests that the presence of a primary amine in Tris is insufficient to stabilize **Pp** coatings, likely due to the relatively low amine density. In contrast, incorporation of **Pa** markedly enhanced coating retention, indicating that **Pa** acts as an anchoring component that firmly immobilizes the coating on the surface. Nevertheless, reactions between Tris and oxidized **Pp** cannot be fully excluded and may contribute to the nitrogen‐containing signals detected by ToF‐SIMS. Previous studies by Ball et al. and Vecchia et al. have demonstrated that Tris buffer molecules can covalently react with dopamine during polydopamine formation [90, 91]. However, as the Tris concentration in this study was kept constant across all samples, its contribution is not expected to affect the observed compositional trends, which primarily arise from systematic variations in the **Pa** and **Pp** precursors. Accordingly, the coatings are described using the simplified term “**PaPp**” to denote the variable components, while acknowledging potential additional incorporation of tris(hydroxymethyl)aminomethane.

Most **Pa** precursors (**Pa1‐Pa6**) were able to form stable coatings independently, as further supported by the detection of CN^−^ signals by ToF‐SIMS (Figure S17 shows the respective ToF‐SIMS images). This finding aligns with previous studies, which attributed the immobilization of **Pa** to electrostatic interactions between the positively charged amine groups of **Pa** and the negatively charged hydroxyl‐terminated DMA surface [92, 93]. Hence, these stable coatings may exhibit adhesive properties toward charged moieties, microorganisms, or mammalian cells, depending on the density of surface‐exposed amine groups.

Interestingly, CNO^−^ signals were also detected for **Pa1** (branched polyethylenimine) and **Pa3** (poly(allylamine)), despite the absence of oxygen in their chemical structures. This behavior can be attributed to ion‐beam‐induced recombination and rearrangement processes inherent to ToF‐SIMS analysis. Nitrogen‐rich polymers are known to adsorb oxygen‐containing species, such as tightly bound water, from ambient air, and under ion bombardment, small anions such as CNO^−^ are energetically favored. A similar phenomenon was reported by Baio et al. for purely amine‐terminated self‐assembled monolayers [94].

Exceptions were observed for **Pa7** (PEG‐diamine with amino groups only at the termini) and the low‐molecular‐weight polyamines **Pa8** (spermine), **Pa9** (tris(2‐aminoethyl)amine), and **Pa10** (diethylenetriamine), which were unable to form stable coatings independently despite the much higher amine density of **Pa8‐Pa10** compared with **Pa1‐Pa6** (Table 2). This observation highlights the importance of macromolecular architecture and multivalent chain entanglement rather than simple amine density. Nonetheless, their crosslinked **PaPp** coatings exhibited strong CNO^−^ signals, indicating the formation of stable networks (Figure 3c). For example, the absence of CNO^−^ signals for **Pa8**‐**Pa10**, **Pp33** (gallic acid), and **Pp34** (ethyl gallate), and their reappearance after crosslinking, confirmed the oxidative coupling between these **Pa** and **Pp** and the immobilization of the resulting coatings (Figure 3c). These results suggest that for small **Pa** molecules, coating stability is strictly governed by crosslinking efficiency with the **Pp** partner.

**TABLE 2:** Amine densities (mM) of **Pa** precursors at a concentration of 1 mg/mL.

Pa	Repeat Unit Mw (g/mol)	Amines per Repeat Unit	Amine Density (mM)
Pa1^a^	43.07	1	4.6
Pa2	43.07	1	23.2
Pa3^a^	57.09	1	3.5
Pa4^b^	209.08	1	4.8
Pa5^c^	164.63	1	6.1
Pa6^c^	192.65	1^d^	5.2
Pa7^e^	8000	2	0.25
Pa8^f^	202.34	4	19.8
Pa9^f^	146.23	4	27.4
Pa10^f^	103.17	3	29.1

^a^ Pa concentration was 0.2 mg/mL.

^b^ HBr form.

^c^ HCl form.

^d^ Number of guanidinium per repeat unit.

^e^ Mw refers to the molecular weight of the polymer; the number of amines is per chain.

^f^ Mw refers to the total molecular weight of the molecule.

In general, most **PaPp** coatings remained stable. Higher CNO^−^ intensities were observed for coatings incorporating **Pa1‐Pa6** regardless of the **Pp** used, consistent with the results obtained from the **Pa** precursors alone. In addition, within the **Pa1** molecular‐weight series, CNO^−^ intensity increased with molecular weight, demonstrating that longer polymer chains enhance network formation.

On the **Pp** side, median CNO^−^ intensities across all **Pa** networks surprisingly did not correlate with the total number of hydroxyl groups. For instance, coatings containing the galloyl‐rich **Pp44** (pentagalloylglucose) and **Pp45** (tannic acid) displayed relatively lower CNO^−^ intensities despite their high hydroxyl content. In contrast, within the gallate ester series, increasing alkyl chain length was associated with lower CNO^−^ intensities (**Pp34‐Pp37**), indicating a clear structural effect. A comparison between phenolic aldehydes and their corresponding phenolic acid analogues (**Pp26‐Pp28** (dihydroxybenzaldehyde) vs **Pp30** (dihydroxybenzoic acid) and **Pp29** (trihydroxybenzaldehyde) vs **Pp33** (gallic acid)) suggested that aldehyde‐containing **Pp** generally produced higher CNO^−^ intensities. This trend is consistent with the ability of aldehydes to undergo direct Schiff base formation with primary amines, providing an additional covalent coupling pathway beyond oxidative phenol‐amine reactions. When grouped by structural classes, median CNO^−^ intensities showed a subtle decrease from simple phenols (**Pp1‐Pp4**) to flavonoids (**Pp5‐Pp25**) and further to structurally diverse non‐flavonoids (**Pp26‐Pp51**). Although the differences were modest, this trend likely reflects decreasing accessibility of oxidizable phenolic sites and increasing steric complexity, which may reduce the effective crosslinking density between oxidized **Pp** and **Pa**. These observations indicate that **Pp** structural effects on coating stability are multifactorial and cannot be captured by a single molecular descriptor. However, given the semi‐quantitative nature of ToF‐SIMS and the chemical diversity of the **Pp** library, these effects remain modest.

The only **PaPp** combinations that consistently failed to form stable coatings were those containing **Pa7**, likely due to its insufficient number of reactive amine sites required for both crosslinking and surface anchoring. Notably, **Pa7Pp49** (PEG‐diamine + piceatannol) exhibited a strong CNO^−^ signal, and several other **Pa7**‐based coatings (**Pa7Pp11**, **Pa7Pp12**, **Pa7Pp17**, **Pa7Pp48**, **Pa7Pp50**, and **Pa7Pp51**) showed weaker but detectable intensities. This exceptional performance of **Pp49** (piceatannol) can be attributed to its planar, hydrophobic stilbene core that promotes *π*–*π* stacking, combined with multiple hydroxyl groups and a reactive catechol capable of oxidizing to an o‐quinone. Interestingly, the structurally related stilbene **Pp47** (resveratrol), which lacks a catechol moiety, did not form stable coatings when crosslinked with **Pa7**. In contrast, methoxy substitution at the 3,5‐positions, as in **Pp48** (pterostilbene), restored coating stability. Methoxy groups may enhance hydrophobic interactions and *π*–*π* stacking, while also acting as electron donors to facilitate para‐quinone‐methide formation under oxidative conditions, providing electrophilic sites for amine coupling. **Pp50** (magnolol) and **Pp51** (honokiol) are the only compounds containing allyl groups, and therefore, the stability of **Pa7Pp50** and **Pa7Pp51** might originate from these allyl groups. Although these structure‐function correlations shed light on possible crosslinking pathways, the precise mechanisms underlying the reactivity of **Pa7** with **Pp11** (hesperetin), **Pp12** (naringin hydrate), **Pp17** (apigenin), and **Pp48‐Pp51** remain complex and will require advanced computational approaches, such as machine learning.

The CNO^−^ signals were consistently accompanied by CN^−^ signals (the heatmap of CN^−^ count intensities can be seen in Figure S18), with corresponding ion counts summarized in Tables S1–S4. The coating thickness of selected spots was evaluated using laser scanning microscopy operating in white light interferometry mode. Based on the height variation observed in the 3D topographical maps, the coating thickness was estimated to range from a few nanometers up to approximately 100 nm, depending on the specific **PaPp** formulation. This thickness range is consistent with the findings of Behboodi‐Sadabad et al., who reported comparable thicknesses for catecholamine‐polyphenolic coatings fabricated at similar precursor concentrations, as determined by spectroscopic ellipsometry [35]. Representative 3D surface maps are shown in Figure S19. In conclusion, these results demonstrate that a diverse library of novel, durable **PaPp** coatings can be fabricated via direct deposition of polymer solutions on negatively charged surfaces, without the need for post‐deposition crosslinking, offering a simple and efficient strategy for surface modifications.

The DMA chips carrying stable coatings were then subjected to four parallel UHT functionality screening lanes: (1) fluorescence properties, (2) metal‐reducing capability, (3) antibacterial activity, and 4) compatibility with human cell culture (Sections 2.3–2.6).

### 2.3 UHT Functionality Screening of PaPp as Fluorescent Coatings

The chemical structures and intrinsic electronic properties of **Pp**, particularly the aromatic rings with conjugated double bonds that enable *π‐*electron delocalization, give rise to their ability to emit fluorescence [22]. The presence of hydroxyl and other functional groups further modulates the fluorescence behavior by influencing the local electronic environment [95]. Such intrinsic fluorescence properties make polyphenolic coatings promising for diverse applications, including optical sensing, bioimaging, and surface patterning, where stable and tunable emission is desired. When crosslinked with **Pa**, these reactions may lead to extended conjugation or the formation of new emissive species, suggesting that the resulting coatings could display tunable fluorescence. However, the diversity of **Pp** structures, moreover, when combined with multiple **Pa** counterparts, yields an extensive combinatorial library whose fluorescence behavior cannot be readily predicted. Therefore, a systematic and UHT screening approach is required to comprehensively evaluate the fluorescence properties of the resulting coatings, which is enabled by the DMA platform.

**FIGURE 4:**
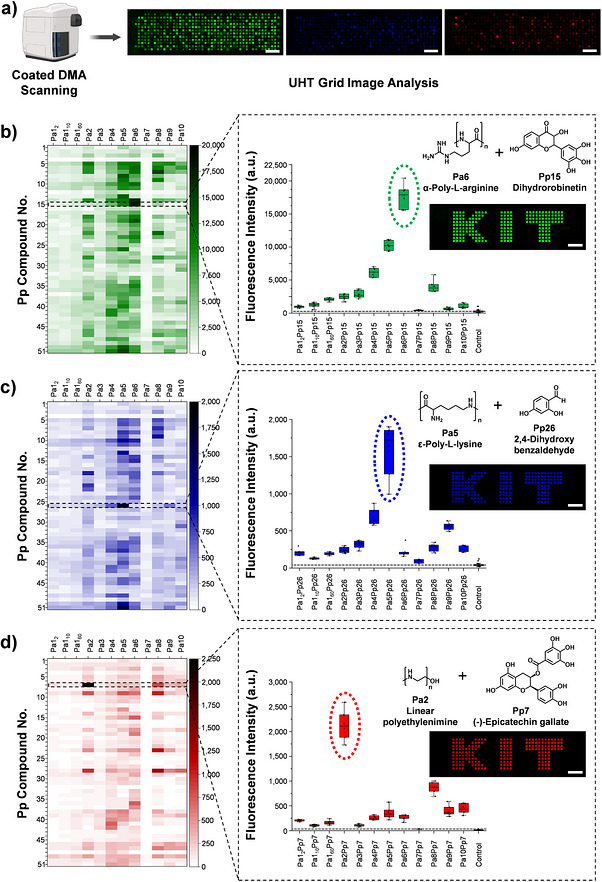
UHT screening of **PaPp** fluorescence properties. (a) Fluorescence properties were screened using a fluorescence slide scanner with a 24‐slide autoloader, generating grid images showing green, blue, and red emission. Fluorescence intensity was quantified using Mapix software with a GenePix Array List (GAL) file. (b–d) Heatmaps of green, blue, and red fluorescence intensities of 612 **PaPp **coatings (12 **Pa × **51 **Pp**). The color scale represents the median fluorescence intensities from six data points per coating. Notably, the green fluorescence was approximately tenfold stronger than blue or red emission. The dashed black boxes highlight the most fluorescent combinations in each channel, which are also shown as boxplots (uncoated spots served as controls). **Pa6Pp15**, **Pa5Pp26**, and **Pa2Pp7** displayed the highest green, blue, and red fluorescence, respectively. The emission profiles were visualized by printing “KIT” using these coatings, confirming their strong fluorescence. Scale bar: 7 mm. The fluorescence images were brightness‐, contrast‐, and saturation‐adjusted for visualization purposes.

In this study, the fluorescence properties of 675 **PaPp** coatings were evaluated using a fluorescence slide scanner equipped with a 24‐slide autoloader and UHT grid image analysis software (Mapix) with a GenePix Array List (GAL) file (Figure 4a). These 675 combinations comprised 12 **Pa** precursors, 51 **Pp** precursors, and 612 **PaPp** combinations, randomly distributed across eight DMAs (six replicates) to minimize spatial bias (Table 1). The resulting grid images showed that most **PaPp** coatings emitted green, blue, or red fluorescence (Figure 4a and Figure S20). Quantitative fluorescence analysis across the **PaPp** library is presented in Figure 4b–d (heatmaps of fluorescence intensities) and Figures S21–S31 (boxplots of fluorescence intensities).

The green fluorescence was approximately tenfold higher in intensity than blue or red emissions (Figure 4b–d). This dominance of green emission is consistent with the findings of Chen et al., who observed pH‐dependent fluorescence of **Pp**‐based carbon dots: blue at pH 1, green at pH 7, and red at pH 12 [22]. Given that **PaPp** coatings were fabricated at pH 8.5, the prevalence of green fluorescence might be rationalized.

Across the entire **PaPp** library, fluorescence intensities followed a consistent trend: coatings containing **Pa5** (ε‐poly‐L‐lysine) and **Pa6** (α‐poly‐L‐arginine) displayed the strongest emissions, followed by those containing **Pa4** (α‐poly‐L‐lysine) and **Pa2** (linear polyethylenimine). In contrast, coatings containing **Pa7** (PEG diamine) did not exhibit fluorescence, consistent with their poor coating stability (Section 2.2). Notably, coatings incorporating **Pa9** (tris(2‐aminoethyl)amine) and **Pa10** (diethylenetriamine), despite possessing the highest nominal amine densities (≈6‐fold higher than **Pa4‐Pa6**; Table 2), produced only moderate fluorescence. This observation demonstrates that simple amine stoichiometry does not govern fluorescence behavior.

Further insight is provided by the **Pa1** (branched polyethylenimine) molecular weight series. Increasing the molecular weight from 2 to 60 kDa resulted in only a modest increase in fluorescence intensity. Thus, neither total chain length nor absolute amine content per chain is sufficient to account for the observed differences. Instead, polymer network architecture emerges as the dominant factor governing emission behavior. The highest‐emitting systems were polypeptide‐based polymers (**Pa4‐Pa6**), which are capable of adopting more ordered and conformationally restricted structures. Such organization likely promotes intermolecular interactions within the coating network, thereby suppressing non‐radiative relaxation pathways and enhancing radiative decay. Consistent with this interpretation, **Pa4** (α‐poly‐L‐lysine) and **Pa5** (ε‐poly‐L‐lysine) exhibited intrinsic autofluorescence (Figure S21 shows boxplots of the respective fluorescence intensities), a phenomenon attributed to clustering‐triggered emission [96, 97]. Aggregation of lysine polymers promotes intermolecular interactions (particularly hydrogen bonding), which activate emissive states [96, 97]. Such aggregation can be induced, for example, by increasing the concentration of the polylysine solution or by drying the solution, which resembles the method used for fabricating the **PaPp** coatings in this study.

Median fluorescence intensities across all **Pa** networks revealed distinct structure‐emission relationships among the 51 **Pp** components. Flavanols bearing catechol B‐rings (**Pp5** ((+)‐catechin hydrate), **Pp6** ((−)‐epicatechin), and **Pp7** ((−)‐epicatechin gallate)) consistently exhibited the highest fluorescence across the entire coating library. In contrast, their pyrogallol B‐ring analogues (**Pp8** ((−)‐epigallocatechin) and **Pp9** ((−)‐epigallocatechin gallate)) displayed significantly lower emission despite containing additional hydroxyl groups. A similar trend was observed for flavanonols, where the catechol‐bearing B‐ring **Pp14** ((+)‐taxifolin) outperformed its pyrogallol analogue **Pp15** (dihydrorobinetin). Glycosylated derivatives, including **Pp12** (naringin hydrate), **Pp13** (hesperidin), **Pp24** (quercetin‐3‐β‐D‐glucoside), and **Pp25** (rutin hydrate), consistently showed reduced fluorescence intensity. Gallate esters (**Pp34‐Pp37**) exhibited a clear hydrophobic optimum, with butyl gallate outperforming both shorter (ethyl) and longer (dodecyl) chain analogues. In addition, all high‐performing **Pp** (**Pp5‐Pp7**, **Pp14**, **Pp18** (luteolin), **Pp46** (pinosylvin), **Pp50** (magnolol), **Pp51** (honokiol)) contain at least two aromatic rings. However, a significant increase in aromatic ring number, as observed for **Pp44** (pentagalloylglucose) and **Pp45** (tannic acid), did not proportionally enhance fluorescence intensity. This behavior suggests the presence of an optimal aromatic density required to enable productive *π*–*π* interactions and cluster formation within the **Pa** network. Together, these findings indicate: (1) that fluorescence in the **PaPp** coatings arises from a cooperative interplay of structural parameters rather than from simple additive contributions, and (2) the existence of structural optima within the **Pp** components that favor emission in the **Pa** network, particularly with respect to aromatic density and hydrophobic balance. Among all coatings, **Pa6Pp15** (α‐poly‐L‐arginine + dihydrorobinetin), **Pa5Pp26** (ε‐poly‐L‐lysine + 2,4‐dihydroxybenzaldehyde), and **Pa2Pp7** (linear polyethylenimine + (−)‐epicatechin gallate) showed the strongest green, blue, and red fluorescence, respectively (Figure 4b–d).

The fluorescence generation mechanism of the **PaPp** coatings was investigated by analyzing excitation and emission spectra in both solution and solid states. Solution‐state spectra were recorded for **Pa5** (representative **Pa**), **Pp26** (representative **Pp**), and their mixtures after 0 h (**Pa5Pp26**_t = 0) and 15 h (**Pa5Pp26**_t = 15) of reaction time. Solid‐state spectra were measured for the **Pa5Pp26** coating. The results are shown in Figure S32.

In dilute solution (39 µM, based on lysine residue molarity), **Pa5** did not exhibit detectable intrinsic fluorescence. In contrast, **Pp26** (36.2 µM) displayed strong blue emission with a maximum at λ_em_ = 480 nm, confirming that **Pp26** is intrinsically fluorescent. The large Stokes shift (≈112 nm) is consistent with an excited‐state intramolecular proton transfer mechanism [98], in which the ortho‐hydroxyl group forms an intramolecular hydrogen bond with the aldehyde carbonyl oxygen. In this process, the phenolic hydroxyl group acts as the proton donor and the carbonyl oxygen as the proton acceptor, leading to emission from the keto tautomer.

The excitation and emission spectra of **Pa5Pp26**_t = 0 were identical to those of **Pp26** alone, indicating that no new emissive species formed immediately upon mixing. After 15 h of reaction at millimolar concentrations (**Pa5**: 3.9 mM, based on lysine residue molarity; **Pp26**: 3.62 mM), the mixture was diluted 100‐fold to micromolar concentrations prior to fluorescence measurements in order to minimize inner filter effects and ensure operation within the linear detection range of the instrument. Following dilution, the emission maximum remained at 480 nm, although noticeable broadening of the excitation spectrum was observed. The absence of an emission redshift in the diluted sample can be rationalized by the reversible hydrolysis of Schiff base (C═N) linkages in an aqueous environment [99]. Upon dilution, the equilibrium shifts toward the dissociated precursors, resulting in fluorescence dominated by unreacted **Pp26**. Furthermore, imine species in dilute solution may undergo efficient non‐radiative relaxation through C═N bond rotation and internal conversion, suppressing imine‐centered emission [100].

In contrast, the thin film coating exhibited markedly different photophysical behavior. The excitation maximum shifted slightly to 372 nm, and the emission maximum showed a clear bathochromic shift to 520 nm, corresponding to green fluorescence. Both excitation and emission spectra were substantially broadened compared to the solution state. These observations indicate that the fluorescence of the **Pa5Pp26** coating is governed by solid‐state effects enabled by covalent Schiff base formation and molecular aggregation.

In the dehydrated and densely packed film, intramolecular motions of the C═N linkage and aromatic units are restricted. This restriction of intramolecular rotation, a key component of aggregation‐induced emission mechanisms, suppresses non‐radiative decay pathways and enhances radiative emission. In addition, the high density of aromatic units and heteroatoms within the polymer network promotes intermolecular electronic coupling, likely involving *π*–*π* stacking and/or intermolecular charge‐transfer interactions. Such through‐space interactions stabilize the excited state and lower its energy, accounting for the bathochromic shift from 480 nm in dilute solution to 520 nm in the coating [101]. The significant spectral broadening can be attributed to inhomogeneous broadening arising from heterogeneous local packing and electronic environments within the polymer network [102, 103].

Unlike the dilute solution, where reversible hydrolysis limits the persistence of the imine chromophore, the solid‐state environment favors stabilization of Schiff base linkages due to high local concentration and limited water accessibility within the densely packed network. The resulting conjugated and aggregation‐locked network governs the final emissive properties of the **Pa5Pp26** coating.

Overall, the fluorescence of the **PaPp** coatings can be described as a reaction‐enabled emission system in which covalent amine‐phenolic coupling reactions establish the emissive framework, while aggregation‐induced restriction of intramolecular motion and intermolecular electronic coupling modulate the spectral position and emission characteristics. In the representative **Pa5Pp26** system, imine (Schiff base) formation provides the dominant covalent linkage. However, across the broader coating library comprising diverse **Pa** and **Pp**, additional coupling pathways, such as conjugate Michael‐type addition to activated aromatic systems, may operate concurrently or preferentially depending on the electronic structure of the **Pp** precursor. Regardless of the specific covalent motif, the formation of a densely crosslinked and dehydrated polymer network restricts intramolecular motion and promotes through‐space interactions, thereby stabilizing the excited state and governing the final emissive properties of the coatings.

Deciphering the precise relationships between the chemical structures of the precursors and the fluorescence of their corresponding **PaPp** coatings remains challenging. The findings presented here clearly demonstrate that **PaPp** coatings function as tunable fluorescent materials, highlighting the potential of **PaPp** coatings for applications in fluorescence‐based sensing, labeling, and optical materials.

### 2.4 UHT Functionality Screening of PaPp as Metal‐Reducing Coatings

Polyphenolic compounds exhibit redox activity arising from the reversible oxidation of phenolic hydroxyl groups to quinones [104]. This property enables **Pp** to act as natural electron donors, capable of reducing metal ions to their metallic states and scavenging reactive oxygen species [20]. Such capabilities are valuable in diverse applications, including catalysis, energy storage, sensing, antimicrobial materials, and biomedical material engineering [20, 44, 104, 105]. Accordingly, **Pp** can mediate electron transfer processes directly at solid interfaces when incorporated into surface coatings, facilitating the in situ formation of metallic nanoparticles or redox‐active surfaces with tunable functionality. Crosslinking with **Pa** stabilizes the resulting **PaPp** coatings and introduces additional chemical versatility. However, this reaction consumes phenolic hydroxyl groups, potentially diminishing their redox properties. As a result, it remains unclear to what extent **PaPp** coatings retain the intrinsic redox activity of their precursor **Pp**. Given the structural diversity and variable oxidation potential of **Pp**, systematic UHT screening is required to map and compare the redox behavior of **PaPp** coatings, an effort made possible by the DMA platform.

**FIGURE 5:**
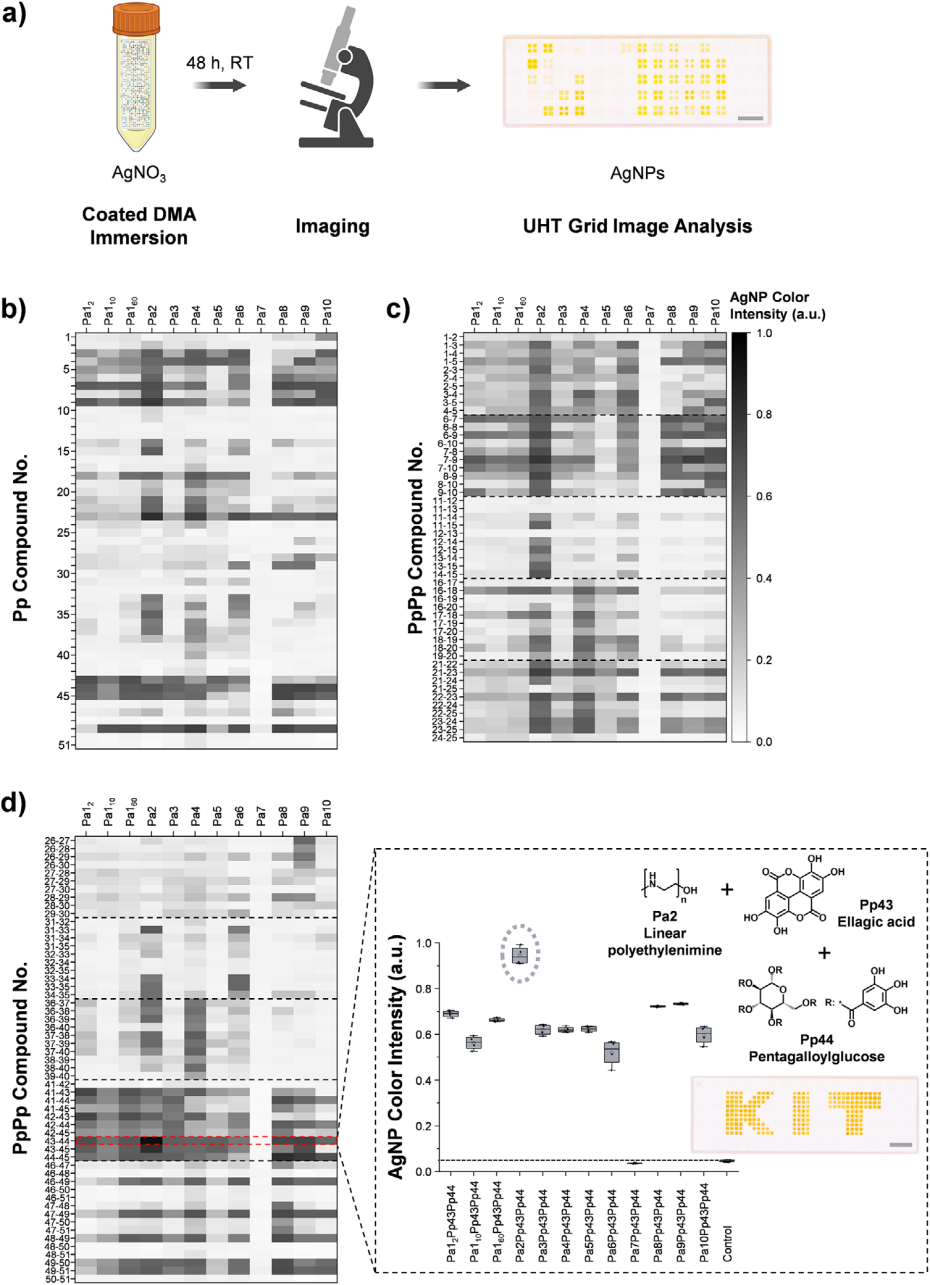
UHT screening of redox activity. (a) Metal redox activity was assessed by immersing coated DMAs in silver nitrate (AgNO_3_, 10 mM) solution; silver ion (Ag^+^) reduction to silver nanoparticles (AgNPs) was visualized using a 3D digital microscope, and the resulting grid images were analyzed using Grid Screener [106]. (b–d) Heatmaps of AgNP color intensity: b) 612 **PaPp** coatings (12 **Pa **× 51 **Pp**) and (c,d) 1260 **PaPpPp** coatings (12 **Pa **× 105 **PpPp**). The color scale represents the median color intensity from four replicates per coating. Higher values indicate greater metal‐reducing capacity. The dashed red box highlights the most active coating, **Pa2Pp43Pp44**. The corresponding boxplot shows AgNP color intensity of **Pp43Pp44** crosslinked with **Pa1_2_‐Pa10**. Uncoated spots served as controls. The silver reduction activity was visualized by printing “KIT” using **Pa2Pp43Pp44**, imaged under a digital microscope. Brightness, contrast, and saturation were adjusted for visualization purposes. Scale bar: 7 mm.

To address this, the redox activity of **PaPp** coatings was evaluated based on their ability to reduce silver ions (Ag^+^) from silver nitrate (AgNO_3_) to metallic silver (Ag^0^), forming silver nanoparticles (AgNPs). Coated DMAs were immersed in AgNO_3_ solution, where the reduction of Ag^+^ to Ag^0^ produced a visible color change from transparent or white to yellow‐orange shades, visualized using a 3D digital microscope (Figure 5a). Variations in color intensity were quantified using Grid Screener [106] and correlated with the reducing strength of the respective **PaPp** coatings, with higher intensity corresponded to greater AgNP formation (Figure 5b–d). The screening involved 2040 unique coatings, derived from 12 **Pa** precursors, 51 **Pp** precursors, 612 **PaPp** combinations, 105 **PpPp** binary combinations, and 1260 **PaPpPp** ternary combinations (12 **Pa **× 105 **PpPp**). Each coating was printed in a 2 × 2 block format (four replicates) separated by one row and/or column of hydrophilic spots, resulting in 8160 coatings distributed across 31 DMAs (Table 1).

UHT screening revealed that **PaPp** coatings reduced Ag^+^ to Ag^0^ to varying extents, as evidenced by differences in the developed AgNP color intensity (Figures S33–S35 and Tables S5–S14 for qualitative analysis, i.e., grid images of **PaPp** coatings after immersion in AgNO_3_ solution; Figure 5b–d and Figure S36–S51 for quantitative analysis, i.e., heatmaps and boxplots of AgNP color intensity, respectively). Analysis of the entire dataset revealed several structure‐function relationships governing the metal‐reducing capability of the coatings, which was primarily dictated by the **Pp** component rather than the **Pa** component.

First, **PaPp** coatings containing multiple catechol or galloyl groups, such as **Pp5** ((+)‐catechin hydrate), **Pp6** ((−)‐epicatechin), **Pp7** ((−)‐epicatechin gallate), **Pp9** ((−)‐epigallocatechin gallate), **Pp18** (luteolin), **Pp23** (myricetin), **Pp43‐Pp45** (ellagic acid, pentagalloylglucose, and tannic acid, respectively), and **Pp49** (piceatannol), exhibited particularly high reducing potential, consistent with their strong electron‐donating capacity (Figure 5b). Coatings containing **Pp** with simpler chemical structures further supported the importance of trihydroxylated phenolic motifs: **Pp3** (pyrogallol) and **Pp4** (hydroxyhydroquinone) outperformed **Pp1** (pyrocatechol) and **Pp2** (resorcinol). This trend was also reflected in the corresponding ternary **PaPpPp** coatings (Figure 5c–d). Among these, **Pa2Pp43Pp44** exhibited the strongest silver‐reducing response, followed by **Pa2Pp43Pp45**, **Pa8Pp44Pp45**, and **Pa2Pp23** (**Pa2** = linear polyethyleneimine, **Pa8** = spermine).

Second, for flavonols, the metal‐reducing capability scaled positively with the number of hydroxyl groups on the B‐ring, as evidenced by the trend **Pp20** (kaempferol, 1 OH) < **Pp21** (morin hydrate, 2 OH) ≈ **Pp22** (quercetin, 2 OH) < **Pp23** (myricetin, 3 OH). A similar trend was observed for stilbenes (**Pp46‐Pp49**), where increasing numbers of hydroxyl groups on the B‐ring enhanced the redox activity of the coatings: **Pp46** (pinosylvin, 0 OH) < **Pp47** (resveratrol, 1 OH) < **Pp49** (piceatannol, 2 OH).

Third, substitution of hydroxyl groups with methoxy or glucoside groups significantly decreased redox activity. This effect was observed for **Pp11‐Pp13** (hesperetin, naringin hydrate, and hesperidin, respectively), **Pp24‐Pp25** (quercetin‐3‐β‐D‐glucoside and rutin hydrate, respectively), **Pp32** (syringic acid), **Pp41** (ferulic acid), **Pp42** (sinapic acid), and **Pp48** (pterostilbene).

In addition, **Pa7**‐containing coatings exhibited negligible AgNP color intensity, consistent with the coating stability screening results (Section 2.2). One coating (**Pa7Pp23**, derived from PEG diamine and myricetin) produced an anomalous response: despite the absence of CNO^−^ and CN^−^ signals in ToF‐SIMS, apparent AgNP formation was detected, which could be attributed to potential cross‐contamination at this spot. Overall, these results demonstrate that **PaPp** coatings largely preserve the intrinsic redox activity of their **Pp** precursors after crosslinking with **Pa**. The UHT dataset further establishes clear structure‐function relationships linking phenolic hydroxyl density and substitution patterns to metal‐reducing activity, providing practical molecular design rules for engineering redox‐responsive surfaces.

### 2.5 UHT Functionality Screening of PaPp as Antibacterial Coatings

Surface functionalization with antibacterial materials represents a promising strategy to combat infections caused by antibiotic‐resistant bacteria [107, 108]. While the discovery of new antibiotics typically requires more than 10 years and costs over $1 billion [109], **Pp** compounds, well known for their intrinsic antibacterial activity [28, 29], are abundant and readily available from natural sources. Thus, **Pp**‐based coatings offer a sustainable and versatile route to address this global health challenge. Moreover, when crosslinked with **Pa** precursors, which have also been reported to exhibit antibacterial effects [51, 52, 53], the resulting **PaPp** coatings may combine the antibacterial functions of both components. However, assessing the antibacterial performance of such coatings remains challenging due to the vast chemical diversity and multitargeted mechanisms of **Pp** [28]. The DMA platform provides an efficient means to overcome this challenge by enabling UHT screening of antibacterial activity across thousands of coatings in parallel.

**FIGURE 6:**
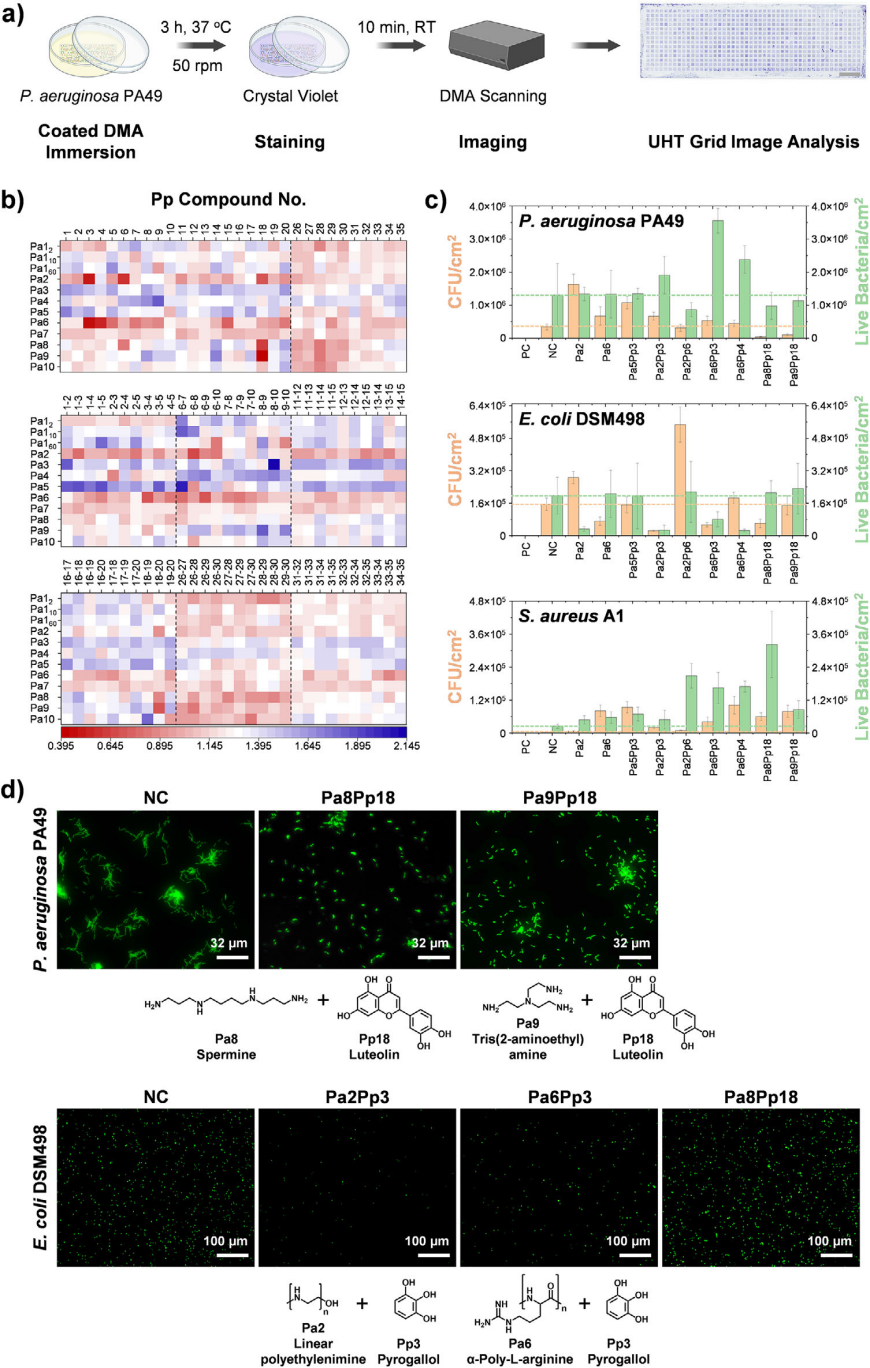
UHT screening of antibacterial activity. (a) Antibacterial activity was screened by incubating coated DMAs with *P. aeruginosa* PA49 (OD_600_ = 0.01 ≈1.4 × 10^7^ CFU/mL), staining with crystal violet (0.5 mg/mL), and quantifying the color intensity using Grid Screener [106]. Scale bar: 7 mm. (b) Heatmap of normalized color intensities (median of eight data points per coating); lower intensity indicates stronger antibacterial activity. (c) Validation of selected hits via CFU and live/dead assays. Data represent means of seven (live bacteria) and fifteen (CFU) data points from triplicate experiments, except for the live/dead assay of **Pa2Pp3** and **Pa6Pp3** against *E. coli* DSM498, for which 13 data points were obtained. Reference coatings: positive control (PC; uncoated coverslips in Müller–Hinton (MH) medium), negative control (NC; uncoated coverslips in bacterial suspension), and non‐hits (**Pa2**, **Pa6**, **Pa5Pp3**). Dashed lines indicate NC level. (d) Representative Syto 9‐stained fluorescence images of *P. aeruginosa* PA49 and *E. coli* DSM498 on **PaPp**‐coated coverslips. Shown are hit coatings with CFU counts below the NC. Brightness, contrast, and saturation were adjusted for visualization purposes.

To explore this capability, the antibacterial potential of **PaPp** coatings was assessed using a crystal violet colorimetric assay. This method is widely used for quantifying bacterial adhesion and biofilm formation [110, 111]. Crystal violet is a triarylmethane dye that binds to the peptidoglycan layer of bacterial cell walls, producing a purple coloration whose intensity reflects the amount of adherent biomass [112]. Lower intensity therefore, indicates fewer bacterial cells and higher antibacterial activity. The screening was performed by immersing the coated DMAs in a suspension of the strong biofilm‐forming strain *P. aeruginosa* PA49 [113] (≈1.4 × 10^7^ CFU/mL, 3 h). Loosely attached bacteria, including dead cells which presumably could not adhere firmly during the short 3 h incubation time, were removed by gentle rinsing. The adherent bacteria were then stained with crystal violet, and the DMAs were scanned using a photo scanner, generating grid images for subsequent color intensity analysis using Grid Screener [106] (Figure 6a). Figure S52a shows a representative scan image and the corresponding Grid Screener detection. Twelve replicates of 1182 coatings were tested, including 12 **Pa** precursors, 30 **Pp** precursors, 360 **PaPp** combinations (12 **Pa** × 30 **Pp**), 60 **PpPp** binary combinations, and 720 **PaPpPp** ternary combinations (12 **Pa** × 60 **PpPp**). The coatings were randomly distributed across 54 DMAs (Table 1). Four of the twelve replicates served as background controls to account for crystal violet adsorption observed for certain **PaPp** coatings (Figure S52b shows a comparison between a test substrate and the corresponding background control, both stained with crystal violet). To compensate for potential bacterial distribution inhomogeneity caused by centrifugal forces during incubation, the printing layout was designed to include local controls: for every two printed rows, one row of uncoated spots was included, enabling local normalization, as illustrated in Figure S52c.

Figure 6b presents the results as heatmaps of median values (eight replicates per coating) normalized to local uncoated controls, and Figures S53–S62 show the corresponding boxplots. Most coatings exhibited normalized crystal violet intensities greater than 1 (Figure 6b), indicating increased bacterial retention and thus adhesive properties of the coatings. This finding aligns with the intrinsic adhesiveness of **Pa** [48, 49, 50] and with the results from the stability screening (Section 2.2). Analysis of the median normalized crystal violet intensities across the **PaPp** coating library revealed several structure‐activity relationships governing antibacterial performance.

On the **Pa** side, first, coatings derived from **Pa6** (α‐poly‐L‐arginine) consistently produced the lowest intensities, highlighting the superior antibacterial performance of guanidinium groups compared to the primary and secondary amines present in the other screened **Pa**. Notably, this occurred despite **Pa6** having a substantially lower amine density than **Pa2** (linear polyethylenimine) or **Pa8‐Pa10** (spermine, tris(2‐aminoethyl)amine, and diethylenetriamine, respectively; Table 2). This observation indicates that the chemical nature of the cationic functionality plays a more critical role than the absolute amine density. Second, high amine density alone does not guarantee antibacterial activity. Although **Pa2** showed the second‐best overall performance, other high‐amine‐density **Pa** (**Pa8‐Pa10**) displayed relatively poor antibacterial activity.

On the **Pp** side, several trends were identified. First, phenolic di‐ and trihydroxybenzaldehydes (**Pp26‐Pp29**) consistently formed one of the top‐performing groups. Second, smaller phenolic molecules generally outperformed bulkier flavonoids. For example, coatings derived from non‐flavonoid phenolics (**Pp26‐Pp35**) typically exhibited stronger antibacterial activity than those derived from flavonoids (**Pp5‐Pp20**). The larger molecular size of flavonoids may introduce steric hindrance, limiting the exposure of active surface functionalities and thus reducing interactions with bacteria. However, simple phenols (**Pp1‐Pp4**), while generally outperforming flavonoids (**Pp5‐Pp20**), were less effective than phenolic aldehydes (**Pp26‐Pp29**). This suggests that **Pp** functional groups, particularly the presence of aldehyde groups, may have a stronger influence on antibacterial activity than molecular size alone.

One notable exception to these trends was **Pp18** (luteolin), which produced highly active coatings despite its relatively large molecular structure. **Pp18**’s high efficacy can be attributed to its planar, highly conjugated structure containing a B‐ring catechol motif, in contrast to structurally related monophenol flavonoids, **Pp17** (apigenin) and **Pp20** (kaempferol), or the less planar flavonoids, **Pp6** ((−)‐epicatechin) and **Pp14** ((+)‐taxifolin). The planar structure likely enables tighter packing of the resulting coating through *π*–*π* stacking interactions, preventing the formation of loosely packed, sterically hindered networks. Furthermore, **Pp18** exhibited pronounced antibacterial synergy specifically with flexible, low‐steric‐hindrance **Pa** (i.e., **Pa8** and **Pa9**), suggesting that rigid, bulky crosslinkers may require highly flexible **Pa** chains to maximize exposure of biocidal surface functionalities.

Coatings with median normalized values below 0.7 were classified as potential antibacterial hits. Based on this criterion, seven candidates were identified: **Pa2Pp3** (0.49; linear polyethylenimine + pyrogallol), **Pa2Pp6** (0.56; linear polyethylenimine + (−)‐epicatechin), **Pa2Pp6Pp8** (0.66; linear polyethylenimine + (−)‐epicatechin + (−)‐epigallocatechin), **Pa6Pp3** (0.46; α‐poly‐L‐arginine + pyrogallol), **Pa6Pp4** (0.52; α‐poly‐L‐arginine + hydroxyhydroquinone), **Pa8Pp18** (0.64; spermine + luteolin), and **Pa9Pp18** (0.40; tris(2‐aminoethyl)amine + luteolin). Interestingly, none of the identified hits contained aldehyde‐functionalized **Pp**. This observation suggests that, within the **PaPp** coating system, the **Pa** component exerts a stronger influence on antibacterial activity than the **Pp** component.

The limited number of active hits contradicts numerous reports on the antibacterial properties of **Pa** [51, 52, 53], **Pp** [28, 29], and their combinations [58, 59, 60, 61]. Several factors may explain this discrepancy:

1. The hydroxyl groups of **Pp** and amine groups of **Pa**, that are responsible for antibacterial activity, may have been largely consumed during the crosslinking reaction.
2. The antibacterial performance of polymers on surfaces and in solution may correlate, but often differs [114, 115]. For example, **Pa2** and **Pa6,** which could form stable coatings independently, did not show antibacterial activity on surfaces (the respective normalized crystal violet color intensities are shown in Figure S53a), despite reports of their killing activity in solution [52, 53].
3. Experimental conditions may have affected **Pp**‐bacteria interactions, as both growth‐promoting and inhibitory effects have been reported [116, 117].
4. The stability screening revealed that **PaPp** coatings are adhesive. As a result, bacterial cells may accumulate on the surfaces, be killed, and then shield the active sites of the polymers, leading to self‐deactivation. Newly approaching bacteria could then attach and proliferate on this layer of dead bacteria. This highlights the importance of incorporating antifouling or repellent functionality to prevent such accumulation [107, 108].
5. Competitive adsorption of medium components may further reduce the antibacterial activity of **PaPp** coatings. Specifically, the nutrient‐rich Müller–Hinton (MH) medium contains amino acids, starch, and other organic molecules that can adsorb onto the coating surface, shielding surface‐exposed active groups through electrostatic interactions or hydrogen bonding, and potentially sterically hindering bacterial contact. In addition, phenolic amino acids present in the MH medium may engage in hydrophobic interactions or *π*–*π* stacking with the **Pp** components of the coatings [118, 119]. The influence of culture medium composition on antibacterial performance has been reported previously [120].

Six of the seven hits were validated using CFU and live/dead assays against *P. aeruginosa *PA49 (Gram‐negative), *E. coli *DSM498 (EC498; Gram‐negative), and *S. aureus* A1 (SAA1; Gram‐positive). Substrates used for the validation were coverslips dip‐coated with the respective polymer mixtures (surface wettability and roughness are listed in Table S15). The results are shown in Figure 6c and the live/dead images in Figure 6d and Figures S63–S68. **Pa8Pp18** and **Pa9Pp18** reduced PA49 CFU by ≈86% and ≈70% relative to the negative control (NC). However, live/dead assays only showed ≈25% and ≈13% reductions, respectively. In addition, the number of live bacteria/cm^2^ detected by the live/dead assay consistently exceeded the CFU/cm^2^ and exhibited a larger standard deviation. These findings suggest that most PA49 cells were viable but nonculturable (VBNC), hence the lower CFU counts. The high standard deviation in the live/dead assay likely arose from the preferential bacterial accumulation at the coverslip edges, leaving the central regions sparsely colonized and resulting in inhomogeneous PA49 distribution.

Three coatings (**Pa2Pp3**, **Pa6Pp3**, **Pa8Pp18**) reduced EC498 CFU by ≈84%, ≈66%, and ≈61%, respectively. Live/dead assays confirmed strong reductions for **Pa2Pp3** (≈86%) and **Pa6Pp3** (≈59%). However, **Pa8Pp18** showed no reduction, suggesting that EC498 cells were likely in a VBNC state, as indicated by the higher live bacteria/cm^2^ compared to the CFU/cm^2^. Interestingly, **Pa6Pp4** reduced live bacteria by ≈87%, but no CFU reduction was observed, implying that the remaining viable EC498 cells were healthy and could proliferate after exposure. Overall, CFU and live/dead data were consistent, indicating that the coatings did not generally promote VBNC in EC498. Notably, some non‐hit coatings also reduced EC498, including **Pa6** (≈54% CFU reduction) and **Pa2** (≈83% live bacteria reduction).

In contrast, no activity was observed against SAA1, where cells likely entered a VBNC state (live bacteria/cm^2^ > CFU/cm^2^). Varying **Pa:Pp** ratios (1:3, 1:5, 3:1, 5:1) in **Pa2Pp3** and **Pa6Pp3** did not improve the antibacterial activity against PA49 or SAA1 (see Figures S69–S72 for the corresponding CFU, number of live bacteria, and live/dead images, respectively). For EC498, increasing the **Pp** fraction in **Pa2Pp3** (**1:3**, **1:5**) had negligible effects, whereas in **Pa6Pp3,** it increased CFU/cm^2^ but reduced live bacteria/cm^2^. In contrast, increasing the **Pa** fraction in both **Pa2Pp3** and **Pa6Pp3** (**3:1**, **5:1**) generally led to higher bacterial counts, particularly in **Pa2Pp3**. These results underscore the need for UHT exploration across broad bacterial libraries and **Pa:Pp** ratios to identify coatings with strain‐specific or broad‐spectrum bioactivity, which our method enables.

Overall, the validated hits achieved <1‐log CFU reduction relative to the NC. While this reduction is statistically significant, it remains insufficient to prevent biofilm formation or to meet regulatory benchmarks for antibacterial medical devices, which typically require ≥4‐log reduction [121]. Further improvements may be achieved by incorporating additional active components or anti‐adhesive materials. Nevertheless, this UHT screening successfully pinpointed promising coatings, laying the groundwork for further optimization and development of bioactive surfaces.

### 2.6 UHT Functionality Screening of PaPp as Human Cell‐Compatible Coatings

Compatibility with human cells is a fundamental requirement for surface coatings intended for biomedical and biointerface applications [122]. Materials that support cell adhesion, spreading, and growth are essential for tissue engineering, implant integration, and the design of biofunctional devices, whereas cytotoxic surfaces can severely compromise performance in biological environments [122]. **Pp** compounds have been reported to modulate cell behavior by influencing protein adsorption and interfacial chemistry [32], while **Pa** are known to promote cell adhesion through their cationic properties [123]. Crosslinking **Pa** with **Pp** therefore, offers a versatile route to generate coatings with diverse interfacial chemistries and potential for tailored cell responses. However, predicting the biological performance of such chemically complex materials remains challenging. Systematic UHT screening is thus needed to evaluate their compatibility with human cells under controlled conditions, a task enabled by the DMA platform.

**FIGURE 7:**
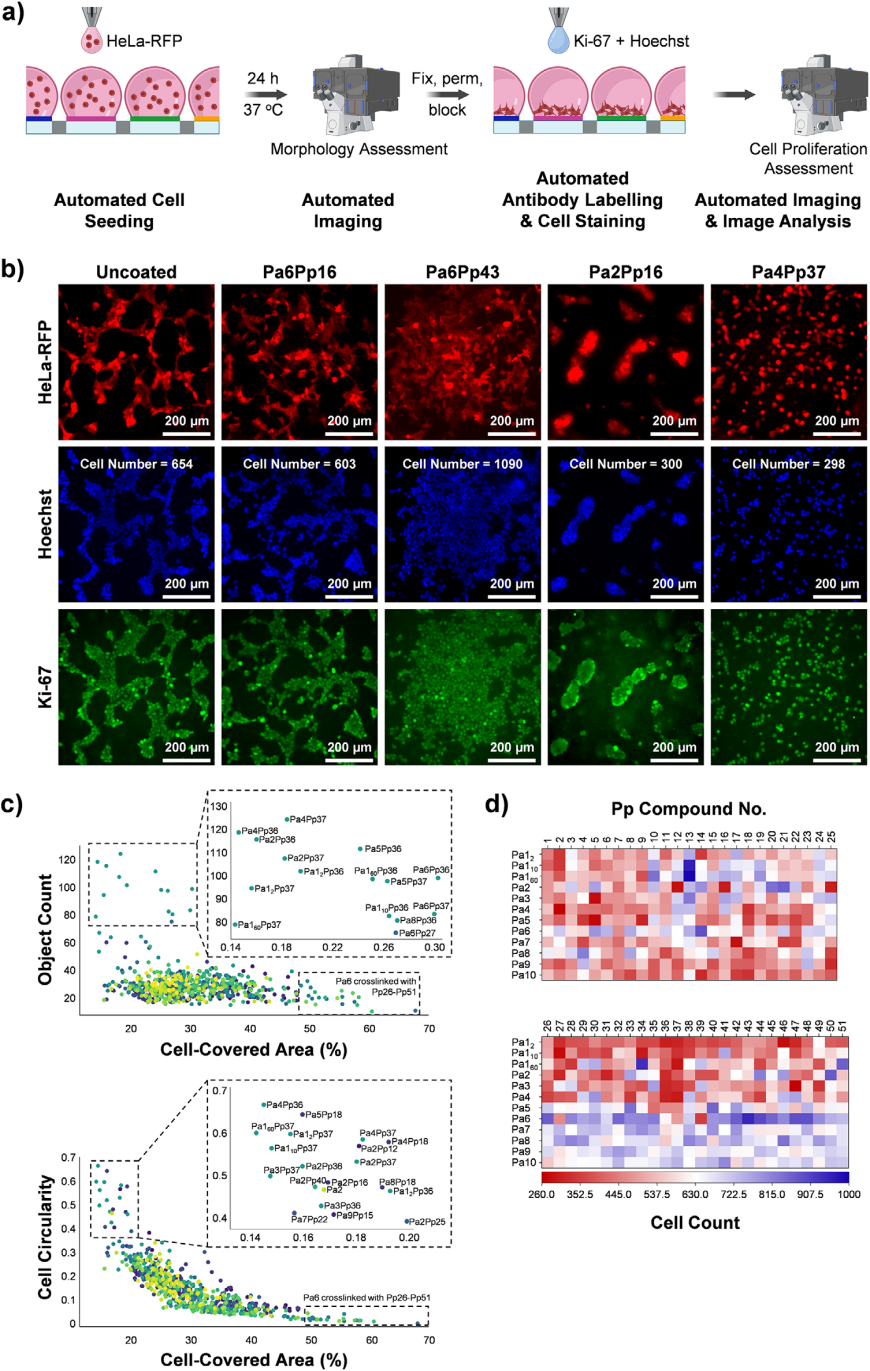
UHT screening of **PaPp** coating compatibility with adherent human cells. (a) The UHT screening employed automated liquid dispenser and fluorescence microscopy. HeLa‐RFP cell suspensions (500 cells/250 nL spot) were seeded on coated DMAs, incubated, and the resulting cell morphology was observed. Afterward, the cells were fixed, permeabilized, and blocked (fix, perm, block). The proliferation marker Ki‐67 (rabbit, 18 µg/mL) and DyLight 488 anti‐rabbit secondary antibody (2 µg/mL, containing 4 ng/mL Hoechst) were sequentially deposited, and fluorescence images were analyzed using ImageJ and CellProfiler [125]. (b) Fluorescence images of HeLa‐RFP cells stained with Hoechst and Ki‐67, showing diverse cell morphologies arising from cell‐**PaPp** coating interactions. Variations in morphology correlated with differences in cell number. Ki‐67 labeling confirmed the compatibility of the coatings with adherent human cells, regardless of their effects on cell morphology. Brightness, contrast, and saturation were adjusted for visualization purposes. (c) Correlations of cell morphological parameters for HeLa‐RFP cells cultured on 675 **PaPp** combinations (12 **Pa**, 51 **Pp**, and 612 **PaPp**). Data shown represent mean values of triplicate samples. (d) Heatmaps of Hoechst‐stained nuclei counts (median of three data points per coating).

**PaPp** coating compatibility with human cell culture was assessed by examining how the coatings influenced the adhesion and morphology of HeLa cells expressing red fluorescent protein (HeLa‐RFP). HeLa‐RFP cells were selected as a model because their strong red fluorescence minimizes background interference from **PaPp** coatings, which predominantly emit in the green channel. Furthermore, the effect of **PaPp** coatings on cell proliferation was quantified based on cell density and Ki‐67 staining, a well‐established marker of active cell division (G1, S, G2, and mitosis phases) [124]. The UHT workflow integrated an automated liquid‐handling system for cell seeding and staining solution dispensing, an automated fluorescence microscope, and UHT image analysis using ImageJ and CellProfiler [125] (Figure 7a). The screening was performed on 675 **PaPp** coatings (12 **Pa**, 51 **Pp**, 612 **PaPp**) distributed across four DMAs (three replicates, Table 1).

Qualitative observations revealed distinct HeLa‐RFP morphologies across the **PaPp** coatings, categorized as spread (e.g., Figure 7b **: uncoated, Pa6Pp16, Pa6Pp43**), clustered (e.g., Figure 7b **: Pa2Pp16**), or rounded (e.g., Figure 7b **: Pa4Pp37**). Binary masks showing the cell‐covered areas on all **PaPp** coatings are provided in Figures S73–S87. On most coatings, cells exhibited behavior similar to those on uncoated spots: they appeared actively spreading or migrating with irregular, jagged edges reflecting active lamellipodia and filopodia extensions, which indicates strong adhesion and thus compatibility with human cell culture. This suggests that the majority of **PaPp** coatings support cell attachment and growth. Interestingly, coatings from crosslinked non‐flavonoid **Pp26‐Pp51** and **Pa6** promoted particularly high surface coverage (e.g., Figure 7b **: Pa6Pp43** (α‐poly‐L‐arginine + ellagic acid); Figures S81–S87: the respective binary masks), implying favorable surface properties for both adhesion and proliferation. Comparable morphologies were also observed on several other coatings, including **Pa1_10_Pp13**, **Pa1_60_Pp13**, **Pa1_60_Pp34**, **Pa2Pp5**, **Pa5Pp33**, **Pa6Pp14**, **Pa8Pp28**, and **Pa8Pp29** (**Pa1_10_** = branched polyethylenimine with Mw = 10 000 g/mol, **Pa1_60_** = branched polyethylenimine with Mw = 60 000 g/mol, **Pa2** = linear polyethylenimine, **Pa5** = ε‐poly‐L‐lysine, **Pa6** = α‐poly‐L‐arginine, **Pa8** = spermine, **Pp5** = (+)‐catechin hydrate, **Pp13** = hesperidin, **Pp14** = (+)‐taxifolin, **Pp28** = 3,5‐dihydroxybenzaldehyde, **Pp29** = 3,4,5‐trihydroxybenzaldehyde monohydrate, **Pp33** = gallic acid, **Pp34** = ethyl gallate).

In contrast, some coatings promoted cell clustering, with cells adhering preferentially to each other rather than to the substrate (e.g., Figure 7b **: Pa2Pp16** (linear polyethylenimine + daidzein)). The resulting compact aggregates, often with smooth boundaries, indicate reduced compatibility with adherent human cells, leading to weakened cell‐surface interactions and clustering as a stress‐induced survival response. Similar behavior was observed on **Pa2**, **Pa2Pp12**, **Pa2Pp25**, **Pa2Pp40**, **Pa4Pp18**, **Pa5Pp18**, **Pa7Pp22**, and **Pa8Pp18** (**Pp12** = naringin hydrate, **Pp18** = luteolin, **Pp22** = quercetin, **Pp25** = rutin hydrate, **Pp40** = chlorogenic acid).

Another distinct morphology was characterized by spherical/rounded cells (e.g., Figure 7b **: Pa4Pp37** (α‐poly‐L‐lysine + dodecyl gallate)). This morphology suggests that cells were able to adhere to the surface but failed to spread, implying poor formation of focal adhesions. Most **Pp36**‐ and **Pp37**‐based coatings induced this morphology, particularly when crosslinked with **Pa1_2_‐Pa5** (Figures S83d and S84a show the respective binary masks), indicating that these surfaces provide the least compatible environment for HeLa‐RFP cells. Notably, **Pp36** (octyl gallate) and **Pp37** (dodecyl gallate) are the only **Pp** precursors in the present library bearing relatively long alkyl chains, which increase surface hydrophobicity and thereby hinder HeLa‐RFP cell attachment and spreading. In contrast, the structurally related **Pp34** (ethyl gallate) and **Pp35** (butyl gallate), which have much shorter alkyl chains, did not induce spherical cell morphology.

Morphological parameters were quantified, supporting the qualitative observations (Figure 7c and Figures S88–S89). Spherical cells exhibited a smaller cell‐covered area, whereas the object count and their circularity were comparatively high. The high surface coverage observed for crosslinked **Pa6** with **Pp26‐Pp51** was likewise confirmed. As expected, increased cell coverage correlated with a lower object count and reduced circularity (Figure 7c), indicating enhanced cell spreading and more uniform surface colonization. The linear relationship between cell circularity and solidity was also verified, with clustered and spherical cells exhibiting higher values of both parameters (Figure S88), consistent with more compact and less‐spread morphologies. Correlations between cell solidity and covered area, cell circularity with object count, and cell solidity with object count further validate these morphological trends (Figure S89).

Cell proliferation activity was quantified by counting Hoechst‐stained nuclei. Cell numbers on most **PaPp** coatings were comparable to those on uncoated spots, averaging approximately 400–650 cells/0.45 mm^2^ (boxplots of Hoechst‐stained nuclei counts are shown in Figures S90–S95). In general, deviations from this range correlated with the observed morphologies: coatings that supported extensive cell spreading showed higher densities, whereas those that induced clustering or spherical morphologies showed lower densities (Figure 7b,d and Figures S90–S95).

Taken together, analyses of cell morphology and cell numbers revealed several general structure‐function relationships within the **PaPp** coating system. First, **Pa** bearing guanidinium groups markedly enhanced human cell proliferation compared with those containing primary amines. For example, **Pa4** (α‐poly‐L‐lysine) and **Pa6** (α‐poly‐L‐arginine) are both polypeptides with similar molecular weight ranges but differ in their side‐chain functionalities. The median cell numbers across the library showed that **Pa6**‐based coatings supported ≈710 cells/0.45 mm^2^, whereas **Pa4**‐based coatings supported ≈480 cells/0.45 mm^2^.

Second, the molecular weight of **Pa** modestly influenced cell proliferation, as demonstrated within the **Pa1** molecular‐weight series. Although a slight increase in cell numbers was observed with increasing molecular weight, the overall cell densities remained within the range observed for the control (uncoated spots). Third, non‐flavonoid **Pp** (**Pp26‐Pp51**) crosslinked with small‐molecule **Pa** (**Pa8‐Pa10**) generally promoted higher cell proliferation compared with many other combinations. Fourth, a strict aliphatic chain‐length threshold was observed. Within the screened **Pp** library, **Pp** bearing alkyl chains of ≥ 8 carbons switched the coatings from cell‐adhesive to cell‐repellent behavior, indicating a hydrophobicity threshold that suppresses cell spreading.

Finally, glycosylated **Pp** derivatives promoted higher cell densities than their corresponding aglycones. Representative examples include **Pp10** ((±)‐naringenin) vs. **Pp12** (naringin hydrate), **Pp11** (hesperetin) vs. **Pp13** (hesperidin), and **Pp22** (quercetin) vs. **Pp24** (quercetin‐3‐β‐D‐glucoside) and **Pp25** (rutin hydrate). However, in general, the cell densities of both aglycones and glycosylated derivatives remained within the range observed for the control (uncoated spots).

Furthermore, Ki‐67 immunolabeling revealed that cells on all **PaPp** coatings were Ki‐67‐positive, indicating that they remained metabolically and mitotically active, even when clustered or rounded (Figure 7b). These results suggest that **PaPp** coatings inducing such morphologies are not cytotoxic but rather provide surfaces that support weak initial attachment and sustained metabolism while preventing the formation of stable focal adhesions and proper spreading. Consequently, cells entered an abnormal yet proliferative, stress‐associated state. Ki‐67‐labeled cell densities were generally consistent with the Hoechst‐stained cell numbers. Representative fluorescence images of Hoechst‐ and Ki‐67‐stained cells on selected coatings are presented in Figures S96–S99.

In conclusion, most **PaPp** coatings were compatible with HeLa‐RFP cells. A small subset exhibited partial compatibility toward adherent cells, as they were non‐cytotoxic yet lacked the features needed for normal spreading. Nonetheless, coatings that promoted clustering may be useful for investigating cell‐cell interactions or for generating 3D‐like cell aggregates in a 2D environment, whereas those that induced spherical morphologies could serve as platforms for single‐cell studies. Together, these findings demonstrate the potential of **PaPp** coatings as a tunable platform for modulating cell‐material interactions through molecular design.

### 2.7 Discussion

An extensive combinatorial library of novel **PaPp** coatings was fabricated and screened, yielding comprehensive coating‐functionality maps that can serve as design guidelines for further coating and material development. Approximately 30 000 coatings were generated using the DMA platform, consuming 260‐fold less substrate, 620‐fold less reagents, and requiring 30‐fold less time compared with conventional single‐substrate synthesis. This remarkable efficiency was enabled by the DMA technology, which supports UHT parallel synthesis and screening in nanoliter volumes. Table 1 summarizes the material and time savings achieved by the UHT approach relative to traditional one‐at‐a‐time coating fabrication.

**FIGURE 8:**
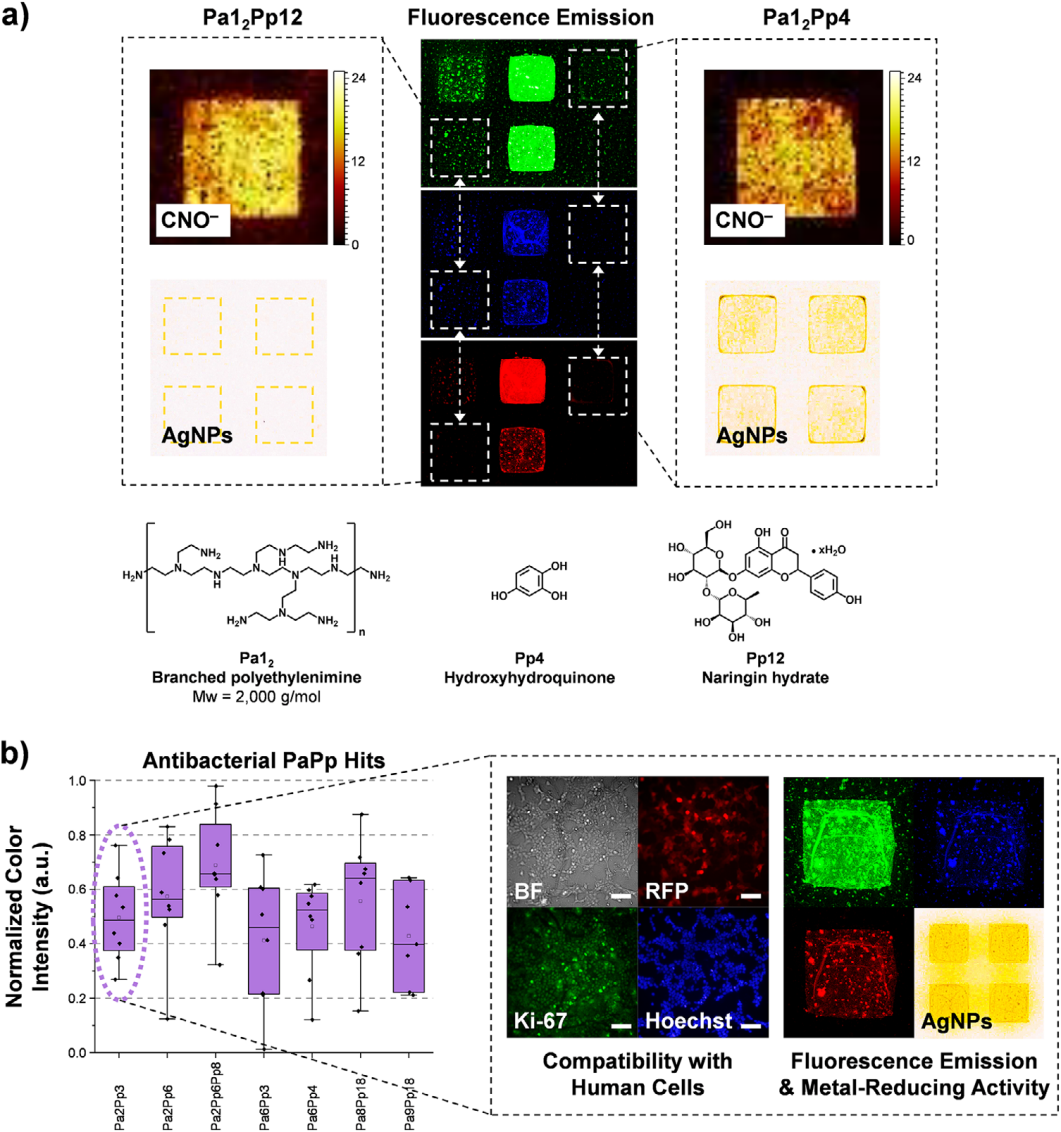
Discovery of multifunctional coatings enabled by UHT screening. (a) Representative results showing that not all stable coatings exhibited multiple functionalities. **Pa1_2_Pp4** and **Pa1_2_Pp12** were confirmed as stable coatings by ToF‐SIMS imaging. However, neither was fluorescent, and only **Pa1_2_Pp4** demonstrated silver‐reducing capability. (b) UHT antibacterial activity screening of 1182 unique coatings identified seven hits as promising antibacterial polymer coatings (normalized color intensity <0.7). Multifunctional coatings were identified by correlating these antibacterial hits with their ability to culture human cells, fluorescence properties, and redox activity (e.g., **Pa2Pp3**). BF = bright field. Scale bar: 100 µm. Spot size: 1 mm × 1 mm. The brightness, contrast, and saturation of the fluorescence and AgNP images were adjusted for visualization purposes.

The UHT workflow enabled the discovery of both specific and multifunctional coatings. Chemical and physical property screenings revealed that most **PaPp** coatings were resistant to aqueous and organic solvents, exhibited fluorescence, and were capable of reducing metal ions. However, not all coatings combined these functionalities. For example, **Pa1_2_Pp12** formed a stable coating, as confirmed by the CNO^−^ signal in ToF‐SIMS analysis, yet lacked fluorescence properties and metal‐reducing activity, as indicated by the absence of fluorescence emission and AgNP color formation (Figure 8a). In contrast, **Pa1_2_Pp4** produced a stable, metal‐reducing coating that was non‐fluorescent (Figure 8a). These examples highlight the diversity of accessible functionalities within the **PaPp** library.

The UHT antibacterial activity screening identified seven promising candidates: **Pa2Pp3**, **Pa2Pp6**, **Pa2Pp6Pp8**, **Pa6Pp3**, **Pa6Pp4**, **Pa8Pp18**, and **Pa9Pp18** (Figure 8b). By integrating property maps across all assays, cross‐comparison of antibacterial activity, fluorescence properties, redox behavior, and human cell compatibility was enabled. For example, **Pa2Pp3** (linear polyethylenimine + pyrogallol) fulfilled all four UHT functionality criteria, emerging as a multifunctional coating candidate (Figure 8b). Other coatings that met all criteria included **Pa2Pp6** (linear polyethylenimine + (−)‐epicatechin), **Pa6Pp3** (α‐poly‐L‐arginine + pyrogallol), **Pa8Pp18** (spermine + luteolin), and **Pa9Pp18** (tris(2‐aminoethyl)amine + luteolin), rendering them promising materials for biomedical coatings, biosensing interfaces, catalytic systems, and environmental applications where both robustness and reactivity are required.

**FIGURE 9:**
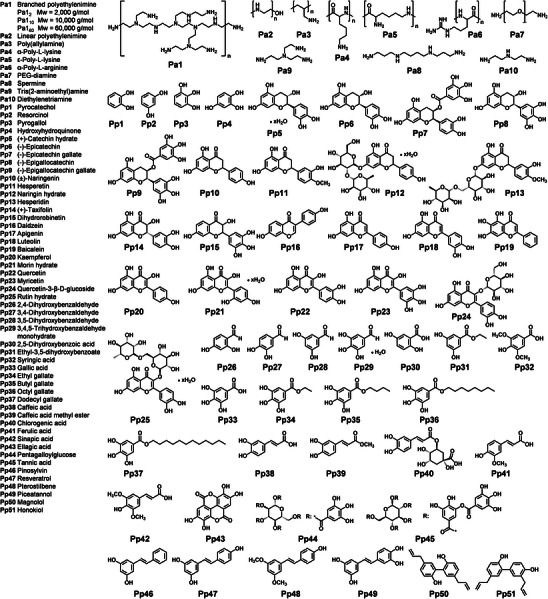
Chemical structures of the 12 **Pa** precursors and 51 **Pp** precursors. The classification of the precursors is shown in Figures S1–S4.

Tables S16–S27 list the complete dataset of 675 **PaPp** coatings (12 **Pa**, 51 **Pp**, 612 **PaPp**; Figure 9 shows the chemical structures of all precursors) with their stability and screening outcomes. The results indicate that the intrinsic chemical and physical characteristics of the **Pp** component are largely retained after crosslinking with **Pa**. Likewise, human cell compatibility was maintained, as evidenced by Ki‐67 positivity. In contrast, antibacterial performance deviated from literature reports, suggesting that further optimization of composition and chemical diversity is necessary to discover more potent antibacterial materials. Alternatively, antibacterial screening of redox‐active coatings represents a logical next step enabled by the DMA platform. For instance, AgNPs are well established as highly effective antibacterial agents [126]. Accordingly, AgNO_3_‐treated coatings may reveal additional antibacterial candidates. Furthermore, incorporation of silver ions or in situ formation of AgNPs on the seven identified antibacterial hits could leverage these materials as a foundation for developing coatings with antibacterial performance approaching clinically relevant benchmarks. Together, these findings demonstrate how UHT synthesis and screening can reveal the full landscape of coating functionalities and interrelations, relationships that would likely remain hidden using conventional low‐throughput methods. This combinatorial UHT approach provides a sustainable platform not only for identifying multifunctional coatings but also for understanding how different functional motifs interplay within complex materials systems.

While the present study focused on **PaPp** coatings, the methodology is not chemically limited to this material class. The open‐array architecture of DMA enables extension to a wide range of polymeric and hybrid systems, including polyelectrolyte complexes, polysaccharides, protein‐ and peptide‐based films, dynamic covalent networks, and supramolecular assemblies. The absence of enclosed microchannels further ensures compatibility with material classes that are challenging for conventional microfluidic platforms. For example, the system is well‐suited for photopolymerization studies, where unobstructed light exposure is required to initiate crosslinking in acrylate‐ or vinyl‐based systems. Similarly, inorganic or hybrid thin films prepared via sol–gel chemistry or nanoparticle deposition can be accommodated without sedimentation or clogging issues. Beyond synthetic materials, enzymes, DNA, peptides, or signaling proteins could be incorporated directly into confined droplets to generate “smart” coatings with catalytic, recognition, or bioactive functions. The DMA's compatibility with both aqueous and organic solvents further broadens the accessible chemical space for polymeric and biomolecular systems.

When compared with other established HT platforms, DMA possesses distinct architectural advantages for coating discovery, particularly for systems that rely on oxidative crosslinking. Standard microwell plates, while widely accessible, feature a deep‐well geometry that results in a low surface‐area‐to‐volume ratio. Consequently, oxygen diffusion to the bottom of the wells can be limited, potentially leading to heterogeneous or incomplete polymerization. In contrast, DMA employs a flat, open‐surface geometry that increases the surface‐area‐to‐volume ratio and facilitates rapid oxygen access, thereby accelerating oxidative crosslinking.

Enclosed microfluidic chips similarly impose constrains on oxygen transport, which is governed by the gas permeability of the device material. Furthermore, while microfluidic platforms are highly effective for synthesizing polymer particles such as nano‐ and microgels, they are less suited for coating discovery. Formation of solid polymer layers along closed microchannel walls can result in channel narrowing or clogging, and in situ characterization of thin films within enclosed channels remains technically challenging.

Inkjet printing represents another HT coating discovery strategy, offering precise droplet deposition and digital pattern control. However, it can suffer from coffee‐ring effects and uncontrolled spreading on unpatterned substrates, which may lead to non‐uniform film morphology. The hydrophilic‐superhydrophobic patterning of DMA confines droplets within predefined domains, maintaining spatial fidelity and promoting uniform coating formation. Chemical vapor deposition provides excellent control over inorganic thin‐film growth and is widely used for uniform and scalable coatings. Nevertheless, CVD is typically limited to volatile precursors and thermally robust systems, and it does not readily support rapid combinatorial screening of complex liquid‐phase libraries or biomolecular formulations.

Collectively, microwell plates, microfluidic chips, inkjet printing, and CVD each offer specific advantages depending on the application. The DMA platform complements these technologies by enabling rapid combinatorial discovery and down‐selection of coating chemistries under mild, solution‐based conditions. Promising candidates identified via DMA can subsequently be optimized or scaled using more conventional deposition methods.

Despite these advances, several limitations remain intrinsic to the UHT combinatorial approach. The nanoliter‐scale DMA format, while enabling remarkable throughput, may introduce a low risk of cross‐contamination between adjacent droplets for specific chemistries, particularly those involving volatile or highly reactive compounds capable of diffusing through vapor or humidity. Minor misting or aerosol transfer during droplet dispensing can also contribute to occasional cross‐contamination. These effects are not generally observed but can be mitigated by controlling ambient humidity, optimizing dispensing parameters, and employing enhanced droplet confinement or enclosed environmental setups to minimize aerosol‐ and vapor‐mediated cross‐contamination. In addition, DMA may not fully capture coating behaviors under macroscale conditions, such as shear stress or heterogeneous surface morphologies. Furthermore, quantitative mechanical characterization at this scale remains challenging, although chemical stability can be effectively monitored by spectroscopic means. Therefore, future work should focus on integrating UHT datasets with predictive modeling and translating nanoscale coating performance to macroscale applications. Addressing these challenges will further accelerate the development of adaptive, multifunctional, and application‐specific surface materials.

## 3 Conclusion

The discovery of new functional coatings is essential for advancing technologies in materials science, energy, healthcare, and environmental applications, yet progress has long been constrained by slow, sequential synthesis and screening. Here, we implemented an ultra‐high‐throughput (UHT) combinatorial strategy that integrates droplet microarray (DMA) technology with wet‐chemical coating formation, enabling thousands of reactions to proceed in parallel within nanoliter droplets confined on hydrophilic microspots bordered by superhydrophobic regions.

Using this platform, nearly 30 000 polyamine‐polyphenolic (PaPp) coatings were synthesized and analyzed using less than 5 mL of total reagents, representing one of the largest and most chemically diverse coating libraries reported to date. Systematic mapping across 12 polyamines (**Pa**) and 51 polyphenols (**Pp**) revealed hundreds of previously unknown fluorescent coatings, more than one hundred redox‐active compositions, and seven PaPp coatings with reproducible antibacterial activity, including combinations achieving up to <1‐log bacterial reduction on macroscale substrates. Stability assays showed that the vast majority of PaPp materials form robust, solvent‐resistant networks, confirming their suitability for applications requiring durable surface modification.

One important outcome of this work is the identification of multifunctional materials that combine several independent attributes, such as fluorescence + redox activity, redox activity + antibacterial performance, or fluorescence + redox + cell compatibility. Notably, coatings such as Pa2Pp3 (linear polyethylenimine + pyrogallol), Pa2Pp6 (linear polyethylenimine + (−)‐epicatechin), Pa6Pp3 (α‐poly‐L‐arginine + pyrogallol), Pa8Pp18 (spermine + luteolin), and Pa9Pp18 (tris(2‐aminoethyl)amine + luteolin) simultaneously exhibited surface stability, intrinsic fluorescence, metal‐reducing capability, antibacterial effects, and compatibility with adherent human cells, demonstrating the potential of PaPp chemistry to generate complex, synergistic functionality from simple building blocks. These multifunctional coatings represent previously inaccessible material classes that would have been extremely unlikely to be discovered using traditional one‐by‐one screening.

Beyond these specific findings, the UHT‐DMA workflow provides a generalizable and scalable platform for the discovery of functional surface chemistries far beyond the PaPp system. Its compatibility with diverse wet‐chemical and photochemical processes enables systematic exploration of coatings with tunable optical, electrical, mechanical, adhesive, catalytic, or biological properties. The combinatorial format further allows interrogation of compositional gradients, crosslinking densities, and structure‐function relationships at a scale and resolution not previously achievable.

In summary, this work establishes a material‐efficient, massively parallel strategy for the rapid discovery, mapping, and optimization of multifunctional coatings. The multifunctional PaPp materials uncovered here illustrate the vast untapped potential of polyphenolic chemistry and highlight how UHT miniaturization can accelerate the development of next‐generation coatings and surfaces across scientific and industrial domains.

## 4 Experimental Section

### 4.1 Materials and Chemicals

All chemicals and solvents (reagent grade) were used as received unless otherwise indicated. Thioglycerol or 3‐mercapto‐1,2‐propanediol, branched polyethylenimine (**Pa1_2_**, 2000 g/mol, diluted to 20 wt.% in H_2_O), branched polyethylenimine (**Pa1_60_**, 60 000 g/mol, 50 wt.% in H_2_O, further diluted to 20 wt.% in H_2_O), poly(ethylene glycol) diamine (**Pa7**, 8000 g/mol), spermine (**Pa8**), tris(2‐aminoethyl)amine (**Pa9**), hesperetin (**Pp11**), naringin hydrate (**Pp12**), rutin hydrate (**Pp25**), 3,5‐dihydroxybenzaldehyde (**Pp28**), and ellagic acid (**Pp43**) were purchased from Thermo Fisher Scientific (Schwerte, Germany).

Vinyltrimethoxysilane, 1*H*,1*H*,2*H*,2*H*‐perfluorodecanethiol (PFDT), 2,2‐dimethoxy‐2‐phenylacetophenone, branched polyethylenimine (**Pa1_10_**, 10 000 g/mol, diluted to 20 wt.% in H_2_O), poly(allylamine) (**Pa3**, 17 000 g/mol, 20 wt. % in H_2_O), pyrocatechol (**Pp1**), pyrogallol (**Pp3**), hydroxyhydroquinone (**Pp4**), (+)‐catechin hydrate (**Pp5**), (−)‐epicatechin (**Pp6**), morin hydrate (**Pp21**), 2,4‐dihydroxybenzaldehyde (**Pp26**), 3,4‐dihydroxybenzaldehyde (**Pp27**), 2,5‐dihydroxy benzoic acid (**Pp30**), gallic acid (**Pp33**), caffeic acid (**Pp38**), sinapic acid (**Pp42**), and tannic acid (**Pp45**) were obtained from Sigma–Aldrich (Darmstadt, Germany).

Silver nitrate, α‐poly‐L‐arginine (**Pa6**, 5000‐15 000 g/mol), diethylenetriamine (**Pa10**), and resorcinol (**Pp2**) were received from Merck KGaA (Darmstadt, Germany). Linear polyethylenimine (**Pa2**, 100 000x000A0;g/mol) was purchased from Polysciences (Hirschberg, Germany), and α‐poly‐L‐lysine (**Pa4**, 4000‐20 000 g/mol) from MP Biomedicals (Eschwege, Germany). ε‐poly‐L‐lysine (**Pa5**, 3500‐4500 g/mol) and gallic acid n‐dodecyl ester (**Pp37**) were received from abcr (Karlsruhe, Germany).

The following chemicals were purchased from BLDpharm (Reinbek, Germany): (−)‐epicatechin gallate (**Pp7**), (−)‐epigallocatechin gallate (**Pp9**), (±)‐naringenin (**Pp10**), hesperidin (**Pp13**), (+)‐taxifolin (**Pp14**), daidzein (**Pp16**), apigenin (**Pp17**), luteolin (**Pp18**), quercetin‐3‐β‐D‐glucoside (**Pp24**), ethyl‐3,5‐dihydroxybenzoate (**Pp31**), syringic acid (**Pp32**), ethyl gallate (**Pp34**), octyl gallate (**Pp36**), chlorogenic acid (**Pp40**), ferulic acid (**Pp41**), pentagalloylglucose (**Pp44**), pinosylvin (**Pp46**), resveratrol (**Pp47**), pterostilbene (**Pp48**), piceatannol (**Pp49**), magnolol (**Pp50**), and honokiol (**Pp51**).

(−)‐Epigallocatechin (**Pp8**), kaempferol (**Pp20**), and myricetin (**Pp23**) were obtained from Activate Scientific (Prien, Germany). Hydrochloric acid 37%, hydrogen peroxide 30%, Tris, and dihydrorobinetin (**Pp15**) were received from Carl Roth (Karlsruhe, Germany). Baicalein (**Pp19**) and quercetin (**Pp22**) were purchased from Apollo Scientific (Manchester, United Kingdom), 3,4,5‐trihydroxybenzaldehyde monohydrate (**Pp29**) from Chem‐Impex (Wood Dale, USA), butyl gallate (**Pp35**) from Tokyo Chemical Industry (TCI, Zwijndrecht, Belgium), and caffeic acid methyl ester (**Pp39**) from Indofine (Hillsborough, USA). Aerosil 200 silica nanoparticles were purchased from Evonik Industries AG (Essen, Germany).

Nexterion Glass B microscope glass slides (25 mm × 75 mm × 1 mm, width × length × thickness) were purchased from SCHOTT Technical Glass Solutions GmbH (Jena, Germany). Indium tin oxide (ITO)‐coated glass slides (8‐12 Ohms, 25 mm × 75 mm × 1.1 mm, width × length × thickness) were purchased from Diamond Coatings (West Midlands, United Kingdom). Glass coverslips (18 mm × 18 mm and 15 mm × 15 mm, width × length; thickness #4) were purchased from ORSAtec GmbH (Bobingen, Germany). The photomask was obtained from Rose Fotomasken (Bergisch Gladbach, Germany).

### 4.2 Fabrication of Droplet Microarray (DMA)

DMAs were fabricated using photolithography and photochemical surface modification.

*Step 1: Preparation of silica nanoparticle suspension*. Glass slides were coated with silica nanoparticles prior to the photolithography process. The nanoparticle suspension was prepared by mixing 250 mg of silica nanoparticles with 30 mL of ethanol. The mixture was sonicated for 30 min at 30°C using an ultrasonic device (Elmasonic S 30 H, Elma Schmidbauer GmbH, Singen, Germany). Subsequently, 340 µL of vinyltrimethoxysilane and 200 µL of hydrochloric acid 37% were added, followed by a 60 min, 30°C of sonication. The suspension was then aged in the dark for 24 h at RT.

*Step 2: Surface coating and engineering*. Glass slides were first activated using a UV‐ozone cleaning system (10 min; Jelight Company Inc., California, USA). The activated slides were then spin‐coated with the silica nanoparticle suspension (500 µL per cycle; 1000 rpm, 500 rpm/s acceleration, 15 s, 5 cycles; SPIN150i spin coater, SPS Europe, Ingolstadt, Germany). Coated slides were cured at 150°C for 1 h on a preheated heating plate (Präzitherm PZ60, Harry Gestigheim GmbH, Düsseldorf, Germany), and subsequently rinsed with ethanol and dried with nitrogen. Photolithography was performed by first depositing PFDT solution (300 µL, 10% v/v in isopropanol) onto each slide. A photomask (14 × 48 = 672 spots; each spot = 1 mm × 1 mm) was placed on the slides, followed by UV irradiation for 90 s (UVA cube OAI model 30, Dr. Hönle AG, Gilching, Germany). The slides were then rinsed with acetone, dried with nitrogen, and backfilled with thioglycerol (300 µL, 10% v/v in a 1:1 ethanol‐water mixture) under UV irradiation for 90 s. Finally, the slides were rinsed with ethanol and dried with nitrogen, resulting in DMAs.

ITO‐coated DMAs were fabricated following the same protocol as described for standard DMAs. The only difference was in the surface activation step: ITO slides were immersed in hydrogen peroxide 30% for 45 min at RT. After activation, the slides were thoroughly rinsed with water and acetone and dried with nitrogen.

### 4.3 UHT Combinatorial Synthesis of PaPp Coatings on DMA

Precursor solutions were prepared at a concentration of 1 mg/mL: **Pa** in 10 mM Tris buffer (pH 8.5) and **Pp** in DMSO. The amine density of each **Pa** precursor is listed in Table 2. These solutions were loaded into an automated liquid‐dispending device (Certus Flex, Fritz Gyger AG, Gwatt, Switzerland). The desired precursor combinations were printed onto predefined spots of the DMAs, with 80 nL of **Pp** solution dispensed first, followed by 80 nL of **Pa** solution. For **Pa**‐only combinations, **Pp** was replaced with DMSO; conversely, **Pa** was replaced with 10 mM Tris buffer (pH 8.5) for **Pp**‐only combinations. Binary **PpPp** combinations were generated by sequentially dispensing two different **Pp** precursors (40 nL each). After printing, the DMAs were placed in a humidified 4‐well plate to prevent droplet evaporation. Humidity was maintained by placing wet pads soaked in 1.5 mL of 10 mM Tris buffer (pH 8.5) beneath the DMAs. The plates were then incubated in the dark for 15 h at RT. Following incubation, the solvent was evaporated under ambient conditions.

### 4.4 UHT Stability Screening

Coated DMAs were subsequently washed with water (24 h, RT, 250 rpm), followed by ethanol (24 h, RT, 250 rpm), yielding stable combinatorial **PaPp** coatings. The stability of the coatings was evaluated using a ToF‐SIMS 5 instrument (ION‐TOF GmbH, Münster, Germany) equipped with a Bi cluster primary ion source and a reflectron‐type ToF analyzer. An ultra‐high vacuum of <3 × 10^−8^ mbar was applied. Measurements were conducted in high‐current bunched mode of the Bi primary ion gun, resulting in short Bi_3_^+^ primary ion pulses with an energy of 25 keV and a target current of 0.35 pA (100 µs cycle time), providing a lateral resolution of approximately 4 µm. Large‐area scans were performed to acquire 3 mm × 3 mm images (100 pixels/mm, 25 frames) in both negative and positive secondary ion polarities. Negative ion spectra were calibrated using C ^−^, CH ^−^, O ^−^, C_2_^−^, and C_2_H ^−^ signals, while positive ion spectra were calibrated using C^+^, CH^+^, CH_2_^+^, CH_3_^+^, C_2_H_2_^+^, C_2_H_3_^+^, and Si^+^ signals. Unless otherwise stated, ToF‐SIMS measurements were performed on ITO‐coated DMAs.

### 4.5 PaPp Coating Thickness Measurement

The thickness of **PaPp** coatings deposited on DMAs was evaluated using a laser scanning microscope (VK‐X4000, Keyence Co., Osaka, Japan) equipped with a white light interferometry module. Three‐dimensional topographical maps were acquired over representative surface areas.

### 4.6 UHT Fluorescence Property Screening

DMAs carrying combinatorial **PaPp** coatings (six replicates of each coating) were sequentially washed with water and ethanol (each washing step 24 h, RT, 250 rpm) to remove unstable coatings. The washed DMAs were then analyzed using a fluorescence scanner equipped with a 24‐slide autoloader (InnoScan 1100 AL, Innopsys, Carbonne, France). Scanning was performed at excitation wavelengths of 635, 532, and 488 nm. The resolution was set to 5 µm/pixel, with a scanning speed of 35 µm/s and a photomultiplier tube (PMT) gain of 5. Mean fluorescence intensity of each spot was quantified using Mapix software (Innopsys, Carbonne, France) based on a GenePix Array List (GAL) file with a feature size of 870 µm in diameter.

### 4.7 Fluorescence Spectroscopy

Fluorescence spectra of **Pa5Pp26** coating cast on a glass coverslip were recorded using a Fluorolog‐3 spectrofluorometer (Horiba JobinYvon IBH FL‐322, HORIBA Europe GmbH, Oberursel, Germany). A coated substrate was prepared according to the *Dip‐Coating of Coverslips* protocol described below and measured directly in the solid state. Solution‐state fluorescence spectra were recorded using a SpectraMax iD3 microplate reader (Molecular Devices GmbH, München, Germany). The PMT gain was set to low to ensure measurements within the linear response range of the detector. **Pa5** was initially dissolved at 1 mg/mL in 10 mM Tris buffer (pH 8.5) and diluted to 0.5 mg/mL by addition of DMSO (1:1 v/v). **Pp26** was dissolved at 1 mg/mL in DMSO and diluted to 0.5 mg/mL by the addition of 10 mM Tris buffer (pH 8.5) (1:1 v/v). The **Pa5Pp26** mixture was prepared by combining equal volumes (1:1 v/v) of 1 mg/mL **Pa5** solution (in 10 mM Tris buffer, pH 8.5) and 1 mg/mL **Pp26** solution (in DMSO). Prior to fluorescence measurements, all solution samples were diluted 100‐fold using a 1:1 v/v mixture of 10 mM Tris buffer (pH 8.5) and DMSO to obtain micromolar concentrations. This dilution was performed to minimize inner filter effects and to ensure operation within the linear detection range of the instrument.

### 4.8 UHT Metal‐Reducing Activity Screening

Unstable **PaPp** coatings were first sequentially removed by thorough washing with water and ethanol (each washing step 24 h, RT, 250 rpm), yielding stable coatings. To evaluate the metal‐reducing capability of **PaPp** coatings (four replicates of each coating), the coated DMAs were immersed in a 10 mM aqueous solution of AgNO_3_ for 48 h at RT, followed by rinsing with water and drying under a nitrogen stream. Images of the coatings were acquired using a digital microscope (VHX‐7000, Keyence Co., Osaka, Japan) equipped with a 20× magnification lens. The color intensity of reduced Ag^+^ on the **PaPp** coatings was quantified using the Grid Screener software [106] (RGB mode in greyscale, minimum value = R255 G255 B255, maximum value = R100 G100 B100).

### 4.9 UHT Antibacterial Activity Screening

**PaPp**‐coated DMAs were first sequentially washed with water and ethanol (24 h each, RT, 250 rpm) to remove unstable coatings. *Pseudomonas aeruginosa* PA49, isolated from a wastewater plant [127], was used to evaluate the antibacterial activity of **PaPp** coatings (eight replicates of each coating). The bacteria were cultured overnight at 37°C with shaking at 100 rpm in Müller‐Hinton (MH) medium (Merck KGaA, Darmstadt, Germany), then diluted to an optical density at 600 nm (OD_600_) of 0.01, corresponding to approximately 1.4 × 10^7^ CFU/mL, in diluted MH medium (1:4 MH:water). Each **PaPp**‐coated DMA was immersed in 30 mL of the diluted bacterial suspension in a Petri‐dish and incubated for 3 h at 37°C with shaking at 50 rpm. After incubation, the slides were gently rinsed with 1× Dulbecco's phosphate‐buffered saline (DPBS, Gibco, Life Technologies GmbH, Darmstadt, Germany) and air‐dried in a clean bench for 1 h. For staining, the dried slides were immersed in an aqueous solution of crystal violet (0.5 mg/mL; Carl Roth, Karlsruhe, Germany) for 10 min at RT. The slides were then rinsed with water and again dried in a clean bench for 1 h. Stained DMAs were imaged using a photo scanner in positive film mode at 6400 dpi. The color intensity of stained bacteria was quantified using Grid Screener software [106] (HSV mode, saturation channel). All intensity values were first background‐corrected by subtracting the corresponding values from background control spots (four replicates of each coating, Figure S52b). The resulting values were then normalized to local control spots (Figure S52c), according to the following equation:

$$\begin{array}{ccc} & & \mathrm{Normalized}\;\mathrm{color}\;\mathrm{intensity}\;\\ & & \;\;=\frac{\mathrm{Valu}e_{\mathrm{sample}}-\;\mathrm{Valu}e_{\mathrm{sample}\;\mathrm{background}}}{\mathrm{Valu}e_{\mathrm{local}\;\mathrm{control}}-\;\mathrm{Valu}e_{\mathrm{local}\;\mathrm{control}\;\mathrm{background}}} \end{array}$$

where Value_sample_ is the color intensity of **PaPp**‐coated spots immersed in PA49 suspension, Value_sample background_ is the color intensity of **PaPp**‐coated spots immersed in 1:4 MH:water in the absence of PA49, Value_local control_ is the color intensity of uncoated spots immersed in PA49 suspension, and Value_local control background_ is the color intensity of uncoated spots immersed in 1:4 MH:water in the absence of PA49.

### 4.10 Dip‐Coating of Coverslips

Glass coverslips were activated using a UV‐ozone cleaning system (30 min; Jelight Company Inc., California, USA). Precursor solutions were prepared at 1 mg/mL: **Pa** in 10 mM Tris buffer (pH 8.5) and **Pp** in DMSO. The activated coverslips were immersed in a 1:1 v/v mixture of **Pa** and **Pp**. For **Pa**‐only coatings, **Pp** was replaced with DMSO. To obtain coatings with higher fractions of one precursor (**Pa2Pp3** and **Pa6Pp3** with **Pa:Pp** ratios of 1:3, 1:5, 3:1, 5:1), the respective precursor was adjusted to higher concentrations (3 or 5 mg/mL). Dip‐coating was performed for 24 h in the dark at RT without agitation. Following dip‐coating, the coverslips were subsequently washed with ethanol (6 h, RT, 250 rpm) and water (12 h, RT, 250 rpm). Surface wettability was characterized using a Drop Shape Analyzer (DSA30B, KRÜSS Scientific, Hamburg, Germany) and surface roughness using confocal microscopy (MarSurf CM select, Mahr GmbH, Göttingen, Germany).

### 4.11 Colony‐Forming Unit (CFU) Assay

The bacterial strains *Pseudomonas aeruginosa* PA49, *Escherichia coli* DSM498, and *Staphylococcus aureus* A1 were cultured overnight in MH medium at 37°C and 100 rpm. The cultures were then diluted 1:100 and incubated further. After 1–2 h, the OD_600_ was measured, and the cultures were diluted to approximately 5 × 10^5^ CFU/mL in 1× PBS. **PaPp**‐coated coverslips were sterilized in 80% ethanol for 1 h, placed in 12‐well square plates, and immersed in 2 mL of the diluted bacterial suspension. Samples were incubated on a rocking platform (4 h, 37°C, 30 oscillations/min; PMR‐30, Grant Instruments, Royston, United Kingdom). After incubation, the bacterial suspension was removed, and each well was gently washed three times with 2 mL of 1× PBS. The coverslips were then transferred to Petri‐dishes, and surface‐attached bacteria were recovered by adding 2 mL of 1× PBS to each coverslip and scrapping the surface with a sterile cell scrapper. Scrapping was performed in two perpendicular directions, five times each, to ensure complete detachment of bacteria. The recovered suspensions were serially diluted in 1× PBS, and 5 × 10 µL of each dilution was spotted onto MH agar plates. The plates were incubated statically for 18 h at 37°C to allow colony formation. The resulting CFUs were then counted. Each experiment was performed in triplicate.

### 4.12 Live/Dead Staining Assay

The incubation protocol followed that of the CFU assay. After incubation and removal of the bacterial suspension, each well was washed three times with 2 mL of 1× cell wash buffer (CWB). Each coverslip was then stained with 200 µL of staining solution prepared from the LIVE/DEAD BacLight Bacterial Viability Kit (Thermo Fisher Scientific, Schwerte, Germany; 2.25 µL Syto 9 and 1.5 µL propidium iodide (PI) in 1 mL of 1× CWB) for 15 min at RT in the dark. After staining, the coverslips were rinsed with 1× CWB, and fluorescence images were acquired using an Axio Imager M2 microscope equipped with an Apotome 2 system (Carl Zeiss, Oberkochen, Germany). Three images were taken per coverslip, and each experiment was performed in triplicate (except for **Pa2Pp3** and **Pa6Pp3**, which were performed five times against *E. coli*). The images were analyzed using CellProfiler software [125]. The highest and lowest cell counts (considered outliers) were excluded from the final live bacteria/cm^2^ calculation.

### 4.13 UHT Human Cell‐Compatibility Screening

#### 4.13.1 Cell Culture

HeLa‐RFP cells (AMS Biotechnology Europe Limited, Abingdon, United Kingdom) were cultured in Dulbecco's Modified Eagle Medium (DMEM, Gibco, Life Technologies GmbH, Darmstadt, Germany) supplemented with 10% heat‐inactivated fetal bovine serum (FBS, Gibco), 1% penicillin‐streptomycin (Gibco), and 5 µg/mL of puromycin (Life Technologies GmbH). The cells were cultured at 37°C in a humidified atmosphere with 5% CO_2_ and split every two days.

#### 4.13.2 Cell Printing on **PaPp**‐Coated DMAs

**PaPp**‐coated DMAs were sequentially washed with water and ethanol (24 h each, RT, 250 rpm) to remove loosely attached or unstable coatings. The DMAs were then sterilized by immersion in ethanol prior to cell printing, followed by air‐drying on a clean bench for 20 min. A cell suspension (2 × 10^6^ cells/mL) was dispensed onto each spot using I.DOT One dispenser (Dispendix GmbH, Stuttgart, Germany) under 70% relative humidity. Each cell droplet had a volume of 250 nL and contained approximately 500 cells. The cell‐printed DMAs were immediately placed in a humidity chamber consisting of a 10 cm Petri‐dish containing 2 mL of DPBS and a humidifying pad moistened with 7 mL DPBS on the lid. The cells were then incubated for 24 h (37°C, 5% CO_2_).

#### 4.13.3 Cell Morphology Observation

After 24 h of incubation, cell morphology was examined using an automated fluorescence microscope (Thunder 3D Imager, Leica Microsystems GmbH, Wetzlar, Germany). For imaging, the DMAs were placed in a 4‐well plate containing humidifying pads and sealed with Parafilm to minimize evaporation. Fluorescence images were acquired with an exposure time of 170 ms, and corresponding brightfield images were also captured. One image was taken per spot. Binary segmentation masks of the fluorescence images were generated using ImageJ Fiji (National Institutes of Health Inc, USA) and analyzed using a custom Python workflow. Each sample image was processed independently to identify connected components corresponding to individual cell clusters. Component identification and attribute quantification were performed using the *skimage.measure* module from the *scikit‐image* library [128], enabling extraction of parameters such as area and perimeter, as well as derived metrics including solidity and circularity. For each image, all attributes were aggregated into a morphological feature vector by computing the area‐weighted mean of the cluster‐level descriptors, enabling quantitative comparison across images and experimental conditions. Experiment‐level feature vectors were obtained by averaging the corresponding sample‐level vectors. These morphological descriptors supported statistical analyses across **Pa** and **Pp** conditions and facilitated visualization and dimensionality reduction for downstream evaluation.

#### 4.13.4 Antibody Labeling and Microscopy Imaging

All solution dispensing was performed using I.DOT One dispenser. Prior to antibody labeling, cells were fixed with paraformaldehyde (PFA, Life Technologies GmbH) by dispensing 83.3 nL of 16% PFA into each cell droplet, resulting a final concentration of 4% PFA. The DMAs were incubated for 20 min at RT and subsequently washed once by dipping into DPBS. Cells were then permeabilized by immersion in 0.1% v/v Triton X‐100 (Life Technologies GmbH) in DPBS for 15 min at RT. After permeabilization, the DMAs were washed once with 0.1% v/v Tween 20 (Life Technologies GmbH) in DPBS and blocked with 5% v/v bovine serum albumin (Life Technologies GmbH) in DPBS for 1 h at RT.

Primary antibody Ki‐67 (from rabbit, 18 µg/mL in DPBS, Life Technologies GmbH) was dispensed onto each spot (350 nL/spot). The DMAs were then placed in a humidity chamber (10 cm Petri‐dish filled with 2 mL DPBS and a lid pad wetted with 7 mL DPBS), sealed with Parafilm, and incubated for 15 h at 4°C. Following incubation, the DMAs were washed once with 0.1% v/v Tween 20 in DPBS, and then 350 nL of secondary antibody solution (goat anti‐rabbit IgG (H+L), DyLight 488, 2 µg/mL in DPBS, Invitrogen, Life Technologies GmbH) containing 4 ng/mL Hoechst 33342 (Life Technologies GmbH) was dispensed onto each spot. The DMAs were incubated for 2 h in the dark at RT, then washed once by dipping into DPBS. After washing, the DMAs were placed in a Parafilm‐sealed 4‐well plate humidity chamber for imaging. The antibody‐stained cells were imaged using an automated Thunder 3D Imager. The acquired images were analyzed using CellProfiler [125] to quantify the number of cells per spot.

### 4.14 Illustration

Illustrations were partially created with BioRender.com.

## Conflicts of Interest

The authors declare no conflicts of interest.

## Supporting information




**Supporting File**: adma73612‐sup‐0001‐SuppMat.pdf.

## Data Availability

Raw data that support the findings of this study are available under https://doi.org/10.35097/xhk635uz30mm59xu.
