# Supplementary material for: Ultra‐High‐Throughput Discovery of Multifunctional Polyphenolic Coatings on Droplet Microarrays
Source: Adv Mater. 2026 Jun 11;38(39):e73612. doi: 10.1002/adma.73612 (PMC13361275; doi:10.1002/adma.73612)
Supplement: Supplementary file 1 — Supporting File: adma73612‐sup‐0001‐SuppMat.pdf. [file ADMA-38-e73612-s001.pdf]

Supporting Information

Ultra-High-Throughput Discovery of Multifunctional Polyphenolic Coatings on Droplet Microarrays

Vania Tanda Widyaya[1], Joaquín E. Urrutia Gómez[2], Paul Reuß[1], Alexander Welle[3,4], Rolf A. Gattung[2], Jana Mayer[1], Peter Krolla[3], Markus Reischl[2], Pascal Friederich[5,6], Anna A. Popova[1], Thomas Schwartz[3], Pavel A. Levkin*[1,7]

[1]Institute of Biological and Chemical Systems-Functional Molecular Systems (IBCS-FMS), Karlsruhe Institute of Technology (KIT), Hermann-von-Helmholtz-Platz 1, 76344 Eggenstein-Leopoldshafen, Germany

[2]Institute of Automation and Applied Informatics (IAI), Karlsruhe Institute of Technology (KIT), Hermann-von-Helmholtz-Platz 1, 76344 Eggenstein-Leopoldshafen, Germany

[3]Institute of Functional Interfaces (IFG), Karlsruhe Institute of Technology (KIT), Hermann-von-Helmholtz-Platz 1, 76344 Eggenstein-Leopoldshafen, Germany

[4]Karlsruhe Nano Micro Facility (KNMF), Karlsruhe Institute of Technology (KIT), Hermann-von-Helmholtz-Platz 1, 76344 Eggenstein-Leopoldshafen, Germany

[5]Institute of Nanotechnology (INT), Karlsruhe Institute of Technology (KIT), Hermann-von-Helmholtz-Platz 1, 76344 Eggenstein-Leopoldshafen, Germany

[6]Institute of Theoretical Informatics (ITI), Karlsruhe Institute of Technology (KIT), Am Fasanengarten 5, 76131 Karlsruhe, Germany

[7]Institute of Organic Chemistry (IOC), Karlsruhe Institute of Technology (KIT), Kaiserstrasse 12, 76131 Karlsruhe, Germany

1. Combinatorial Formula

The number of binary combinations derived from 8,000 polyphenol compounds was calculated using the combination formula:

$$nCr = \frac{n!}{(r!\,(n-r)!)}$$

where *n* is the total number of objects (i.e., 8,000) and *r* is the number of elements selected (i.e., 2 for binary combinations).

2. Chemical Structures of Polyamines (**Pa**) and Polyphenols (**Pp**)

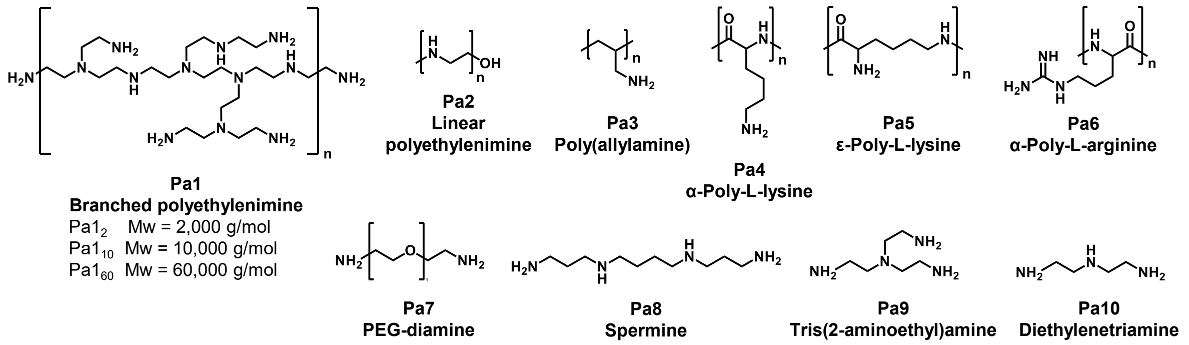

Figure S1. Chemical structures of **Pa** precursors screened in this study. **Pa1** was screened at three different molecular weights (Mw), respectively.

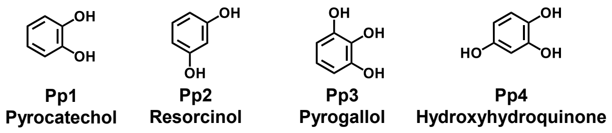

Figure S2. **Pp** with a basic chemical structure of a single phenyl ring attached to hydroxyl groups screened in this study.

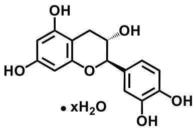

Pp5
(+)-Catechin hydrate

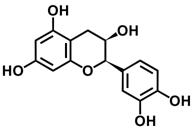

Pp6
(-)-Epicatechin

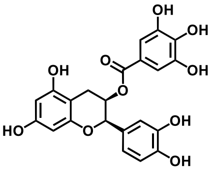

Pp7
(-)-Epicatechin gallate

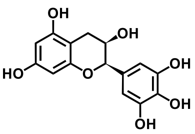

Pp8
(-)-Epigallocatechin

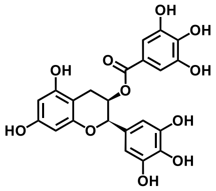

Pp9
(-)-Epigallocatechin gallate

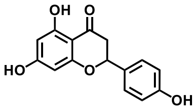

Pp10
(±)-Naringenin

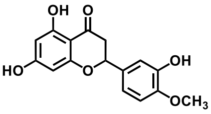

Pp11
Hesperetin

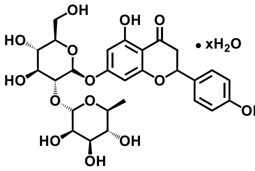

Pp12
Naringin hydrate

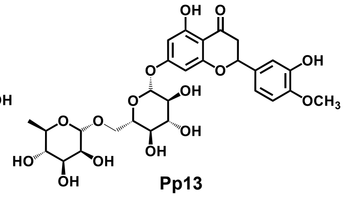

Pp13
Hesperidin

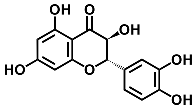

Pp14
(+)-Taxifolin

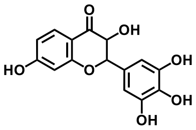

Pp15
Dihydrorobinetin

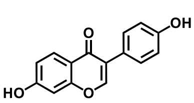

Pp16
Daidzein

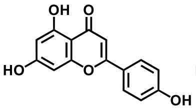

Pp17
Apigenin

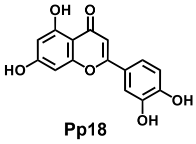

Pp18
Luteolin

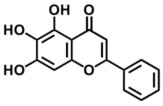

Pp19
Baicalein

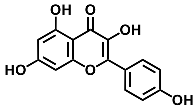

Pp20
Kaempferol

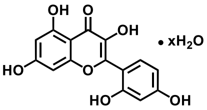

Pp21
Morin hydrate

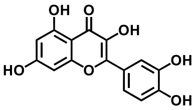

Pp22
Quercetin

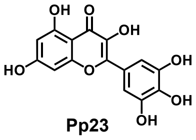

Pp23
Myricetin

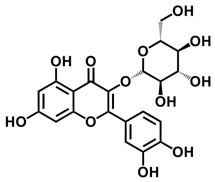

Pp24
Quercetin-3-β-D-glucoside

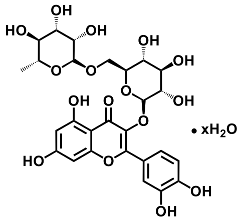

Pp25
Rutin hydrate

Figure S3. Chemical structures of flavonoid **Pp** screened in this study: flavanols (**Pp5-Pp9**), flavanones (**Pp10-Pp13**), flavanonols (**Pp14-Pp15**), flavones (**Pp16-Pp19**), and flavonols (**Pp20-Pp25**).

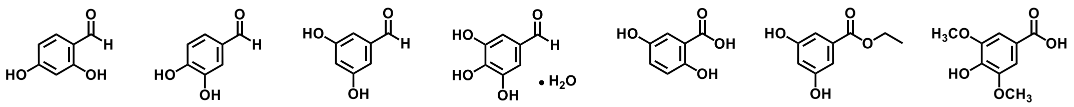

Pp26 2,4-Dihydroxy benzaldehyde; **Pp27** 3,4-Dihydroxy benzaldehyde; **Pp28** 3,5-Dihydroxy benzaldehyde; **Pp29** 3,4,5-Trihydroxy benzaldehyde monohydrate; **Pp30** 2,5-Dihydroxy benzoic acid; **Pp31** Ethyl-3,5-Dihydroxybenzoate; **Pp32** Syringic acid

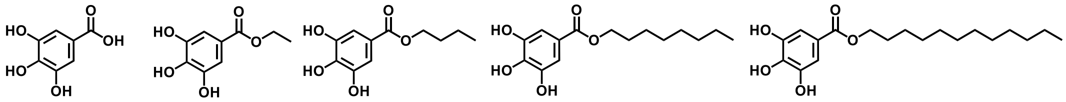

Pp33 Gallic acid; **Pp34** Ethyl gallate; **Pp35** Butyl gallate; **Pp36** Octyl gallate; **Pp37** Dodecyl gallate

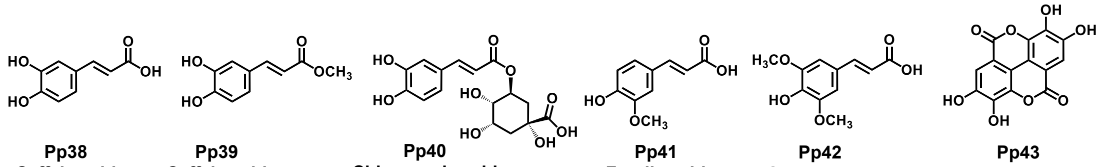

Pp38 Caffeic acid; **Pp39** Caffeic acid methyl ester; **Pp40** Chlorogenic acid; **Pp41** Ferulic acid; **Pp42** Sinapic acid; **Pp43** Ellagic acid

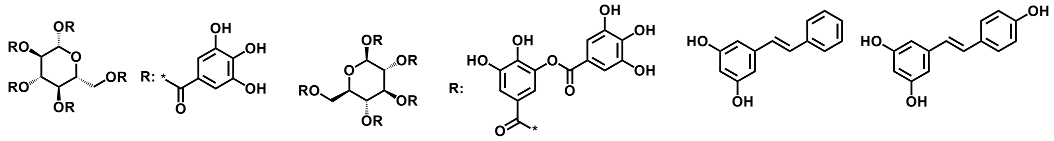

Pp44 Pentagalloylglucose; **Pp45** Tannic acid; **Pp46** Pinosylvin; **Pp47** Resveratrol

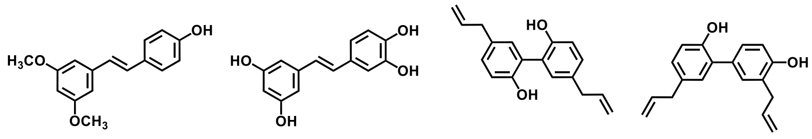

Pp48 Pterostilbene; **Pp49** Piceatannol; **Pp50** Magnolol; **Pp51** Honokiol

Figure S4. Chemical structures of non-flavonoid **Pp** screened in this study: phenolic aldehydes (**Pp26-Pp29**), phenolic acids (**Pp30-Pp45**), stilbenes (**Pp46-Pp49**), and lignans (**Pp50-Pp51**).

3. UHT Screening of Coating Stability

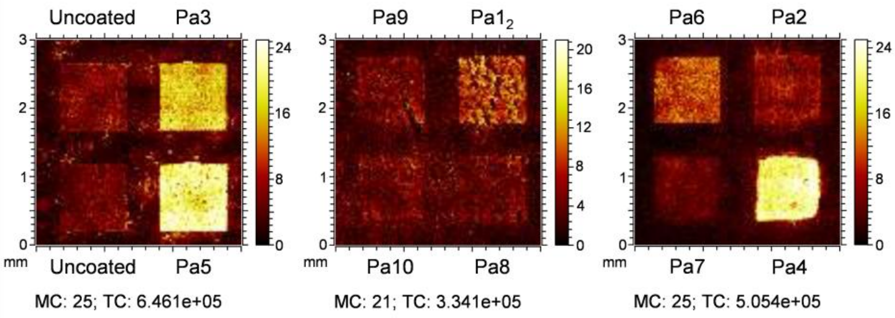

Figure S5. Screening of coating stability using ToF-SIMS (CNO^- signal): imaging of individual precursors **Pa1-Pa10**, randomly distributed on the DMA. Uncoated spots serve as reference controls. Spot size: 1 mm × 1 mm. The color scale represents the CNO^- intensities (ion counts). MC = maximum counts in a single pixel. TC = total counts across the entire image. ToF-SIMS images of **$Pa1_{10}$** and **$Pa1_{60}$** are shown separately in **Figure S6**, top left.

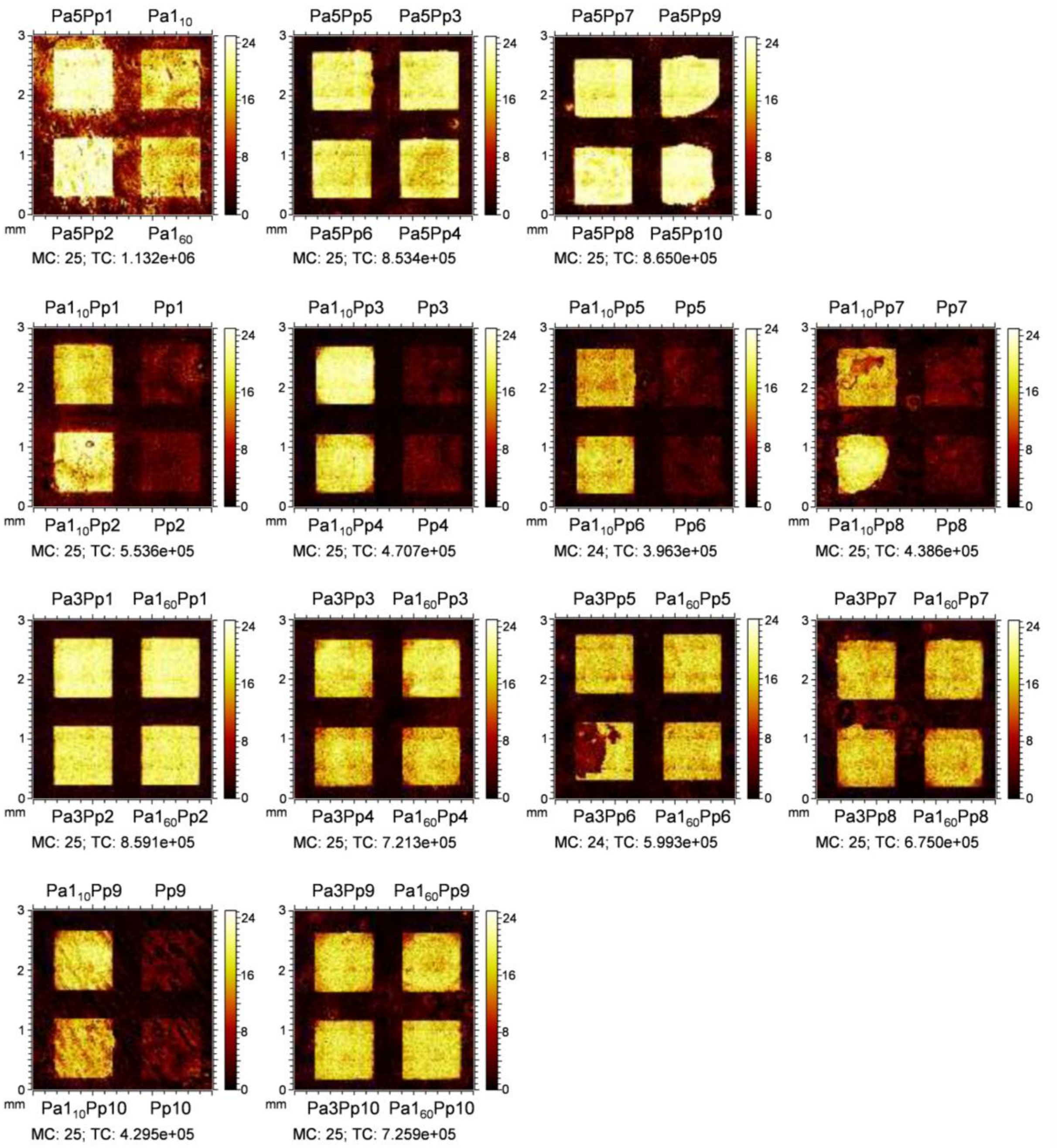

Figure S6. Screening of coating stability using ToF-SIMS (CNO^- signal): imaging of coatings made of **Pa1$_{10}$**, **Pa1$_{60}$**, **Pa3**, and **Pa5** crosslinked with **Pp1-Pp10**, respectively. Images of the individual precursors **Pa1$_{10}$** and **Pa1$_{60}$** (top left), as well as precursors **Pp1-Pp10**, are also included. Each coating has a dimension of 1 mm × 1 mm. The color scale represents the CNO^- intensities (ion counts). MC = maximum counts per pixel. TC = total counts per image.

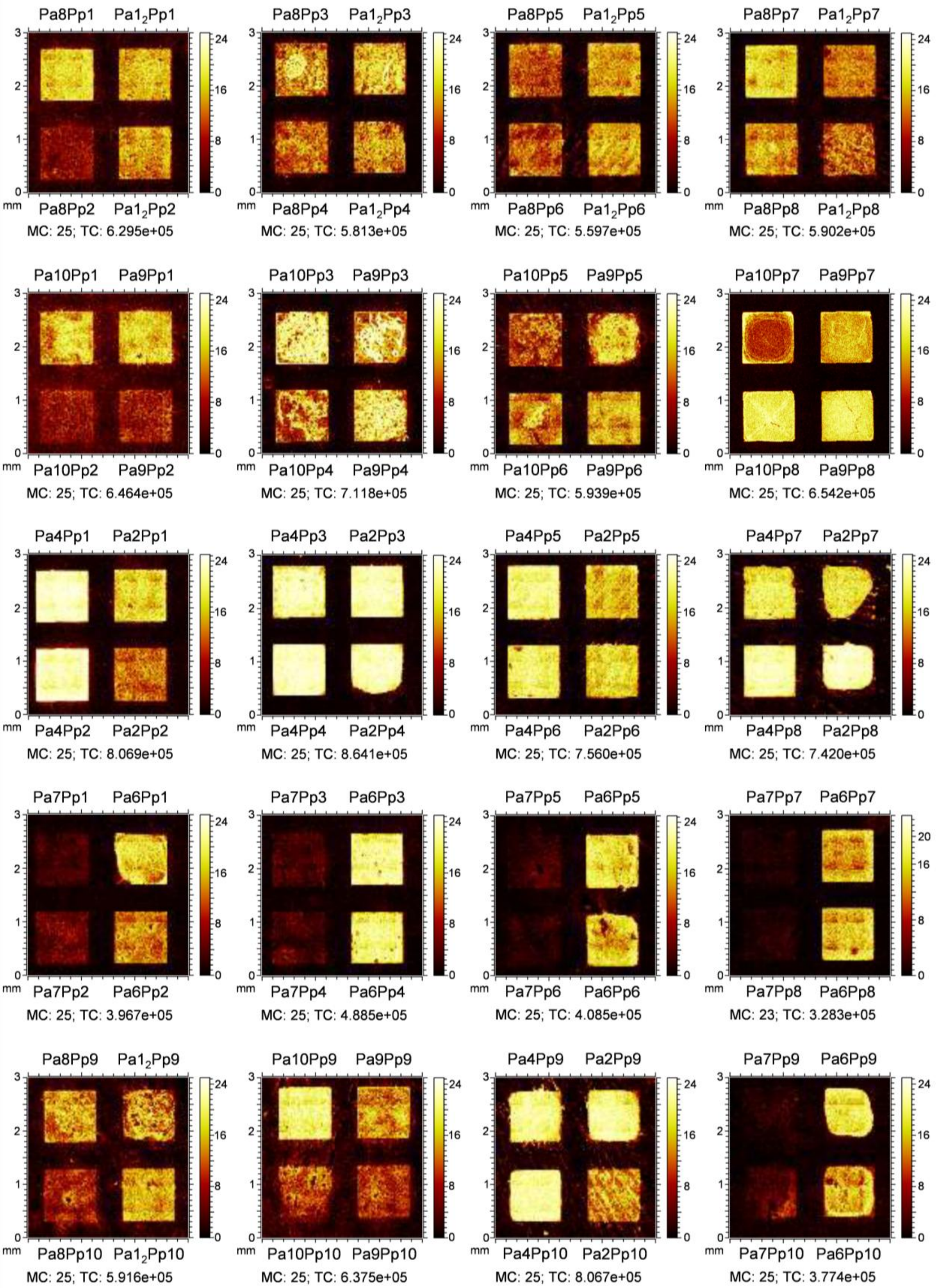

Figure S7. Screening of coating stability using ToF-SIMS (CNO^- signal): imaging of coatings made of **Pa1$_2$**, **Pa2**, **Pa4**, and **Pa6-Pa10** crosslinked with **Pp1-Pp10**, respectively. Each coating has a dimension of 1 mm × 1 mm. The color scale represents the CNO^- intensities (ion counts). MC = maximum counts in a single pixel. TC = total counts across the entire image.

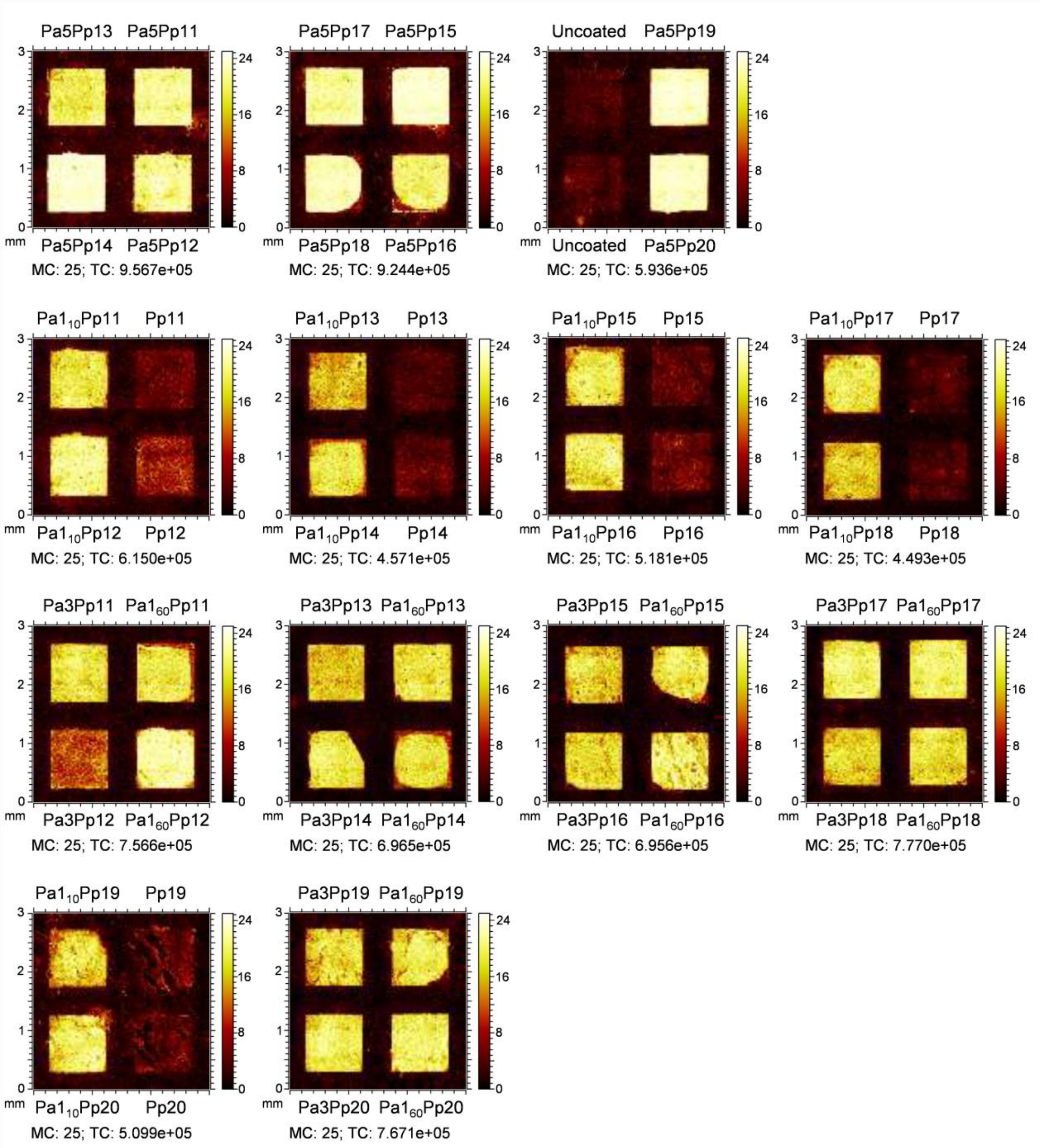

Figure S8. Screening of coating stability using ToF-SIMS (CNO^- signal): imaging of coatings made of **Pa1$_{10}$**, **Pa1$_{60}$**, **Pa3**, and **Pa5** crosslinked with **Pp11-Pp20**, respectively. Images of the individual precursors **Pp11-Pp20** are also included. Uncoated spots serve as reference controls. Each coating has a dimension of 1 mm × 1 mm. The color scale represents the CNO^- intensities (ion counts). MC = maximum counts in a single pixel. TC = total counts across the entire image.

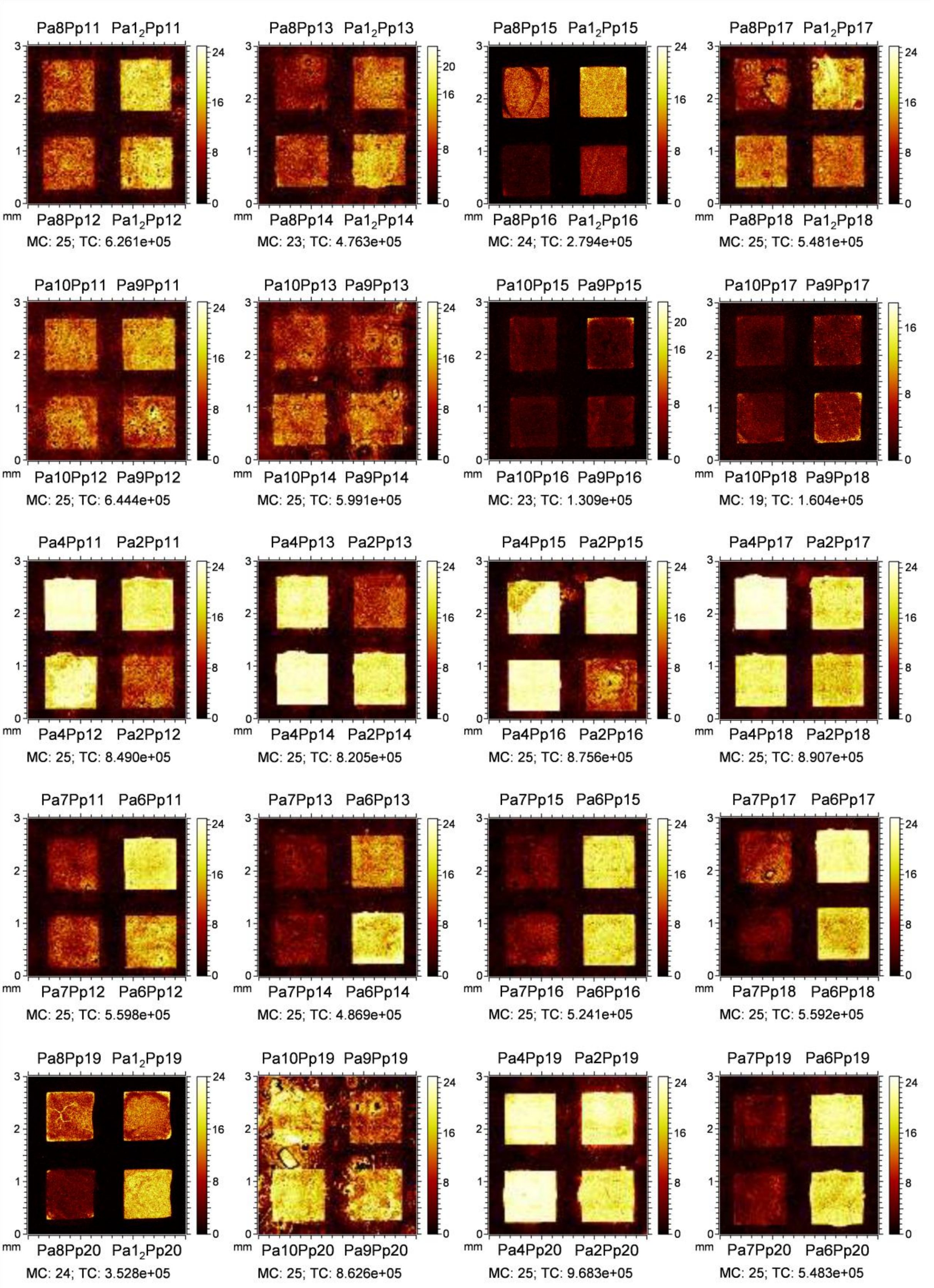

Figure S9. Screening of coating stability using ToF-SIMS (CNO^- signal): imaging of coatings made of **Pa1$_2$**, **Pa2**, **Pa4**, and **Pa6-Pa10** crosslinked with **Pp11-Pp20**, respectively. Each coating has a dimension of 1 mm × 1 mm. The color scale represents the CNO^- intensities (ion counts). MC = maximum counts in a single pixel. TC = total counts across the entire image.

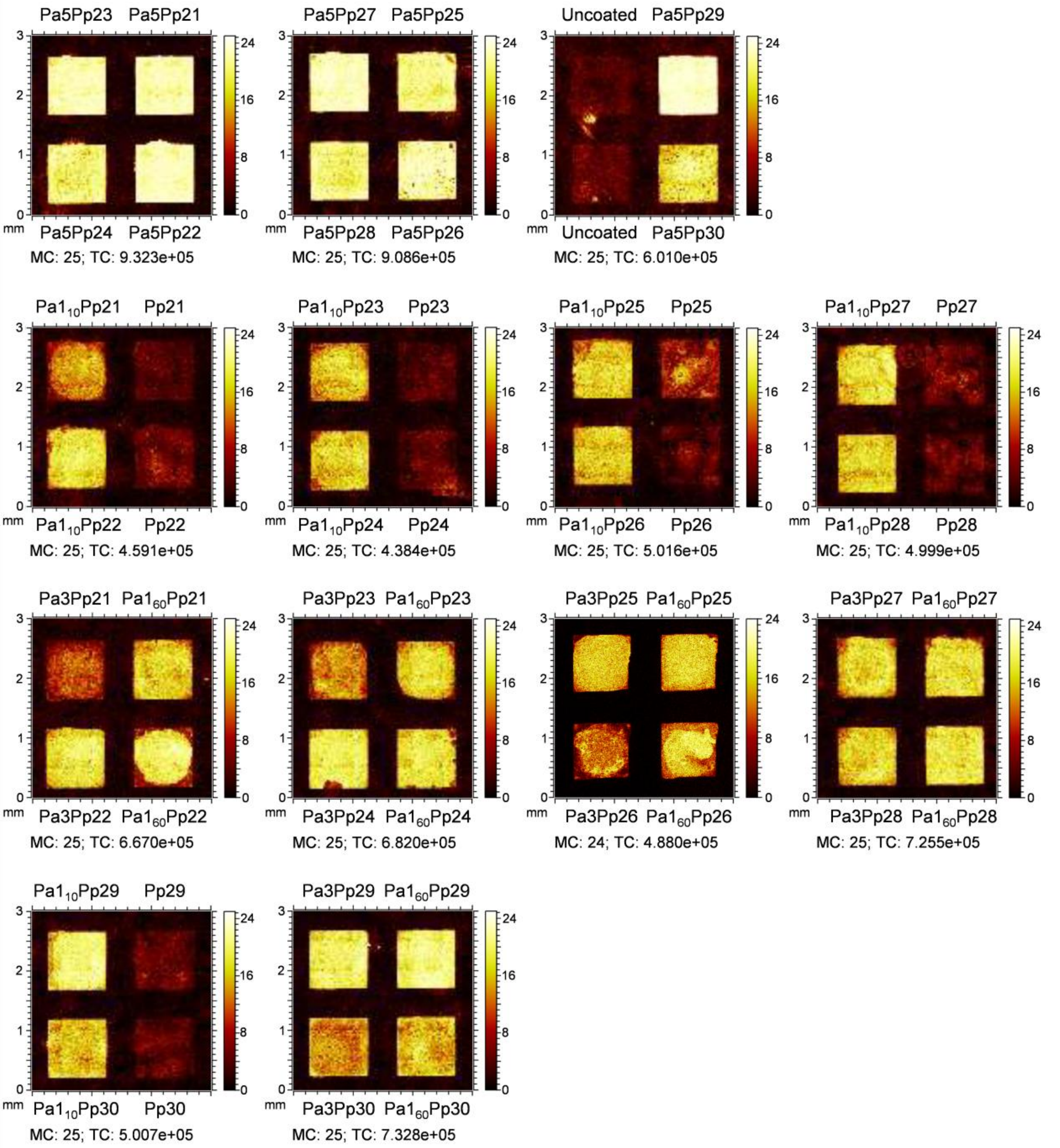

Figure S10. Screening of coating stability using ToF-SIMS (CNO^- signal): imaging of coatings made of **Pa1$_{10}$**, **Pa1$_{60}$**, **Pa3**, and **Pa5** crosslinked with **Pp21-Pp30**, respectively. Images of the individual precursors **Pp21-Pp30** are also included. Uncoated spots serve as reference controls. Each coating has a dimension of 1 mm × 1 mm. The color scale represents the CNO^- intensities (ion counts). MC = maximum counts in a single pixel. TC = total counts across the entire image.

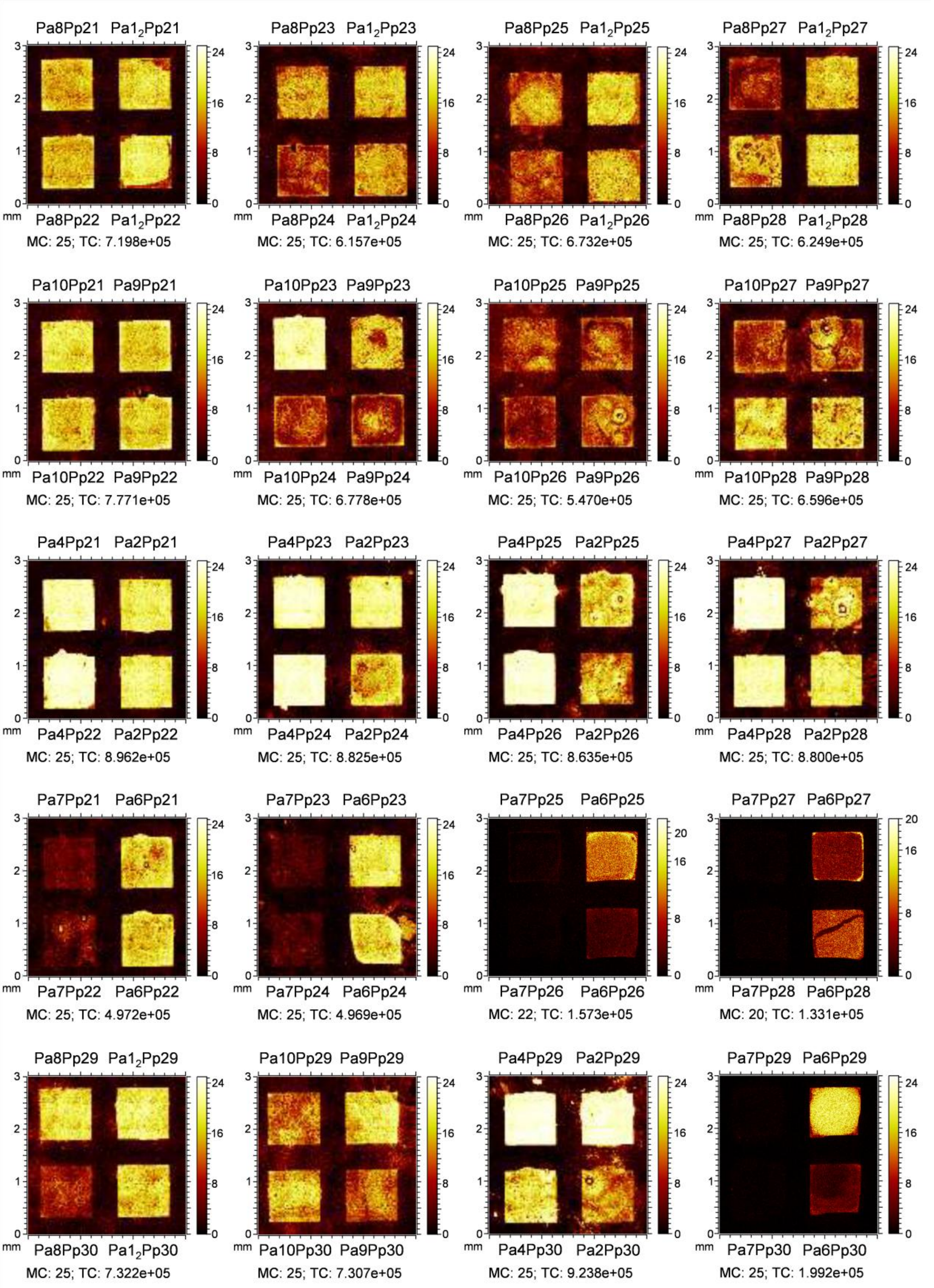

Figure S11. Screening of coating stability using ToF-SIMS (CNO^- signal): imaging of coatings made of **Pa1$_2$**, **Pa2**, **Pa4**, and **Pa6-Pa10** crosslinked with **Pp21-Pp30**, respectively. Each coating has a dimension of 1 mm × 1 mm. The color scale represents the CNO^- intensities (ion counts). MC = maximum counts in a single pixel. TC = total counts across the entire image.

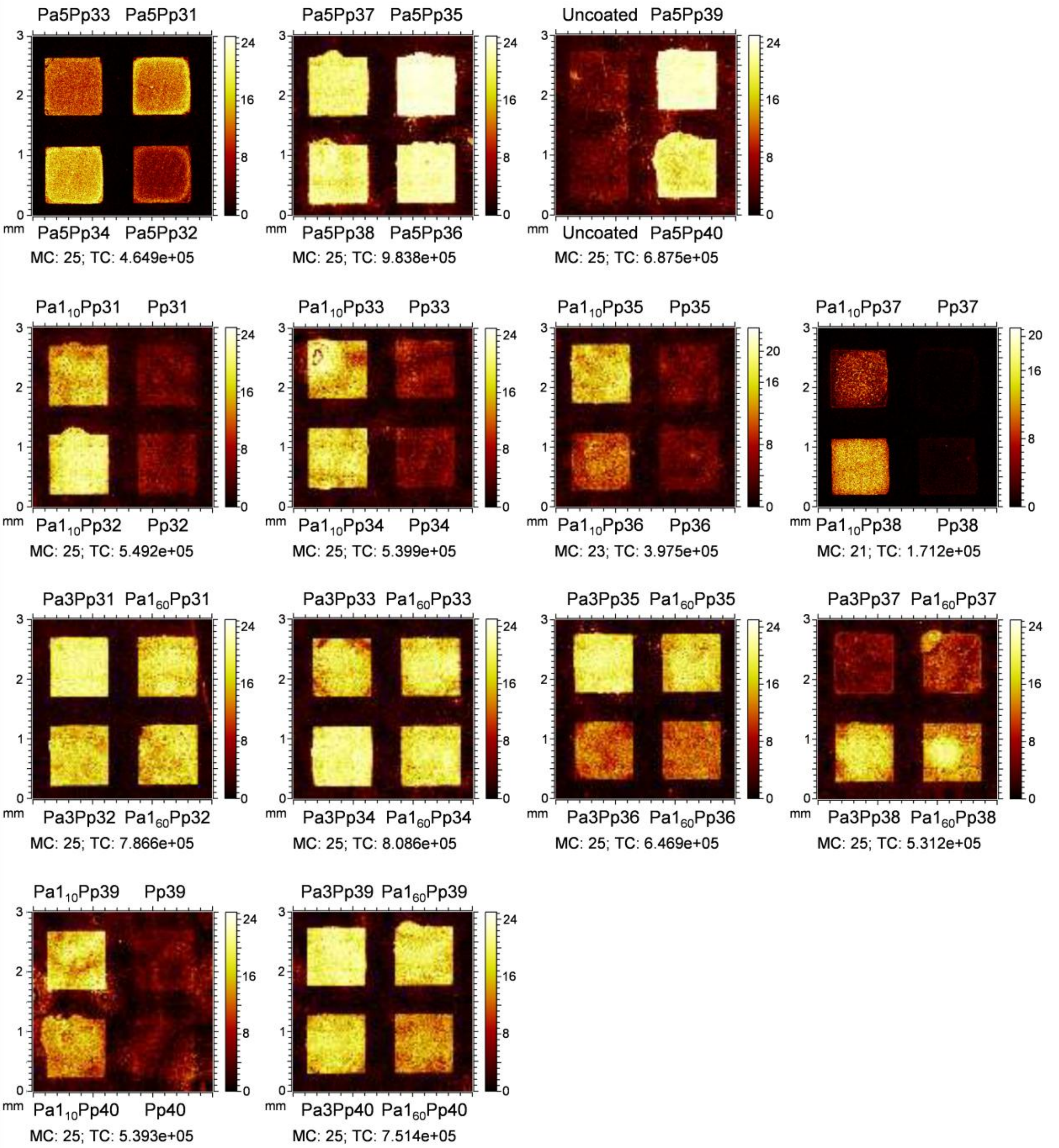

Figure S12. Screening of coating stability using ToF-SIMS (CNO^- signal): imaging of coatings made of **Pa1$_{10}$**, **Pa1$_{60}$**, **Pa3**, and **Pa5** crosslinked with **Pp31-Pp40**, respectively. Images of the individual precursors **Pp31-Pp40** are also included. Uncoated spots serve as reference controls. Each coating has a dimension of 1 mm × 1 mm. The color scale represents the CNO^- intensities (ion counts). MC = maximum counts in a single pixel. TC = total counts across the entire image.

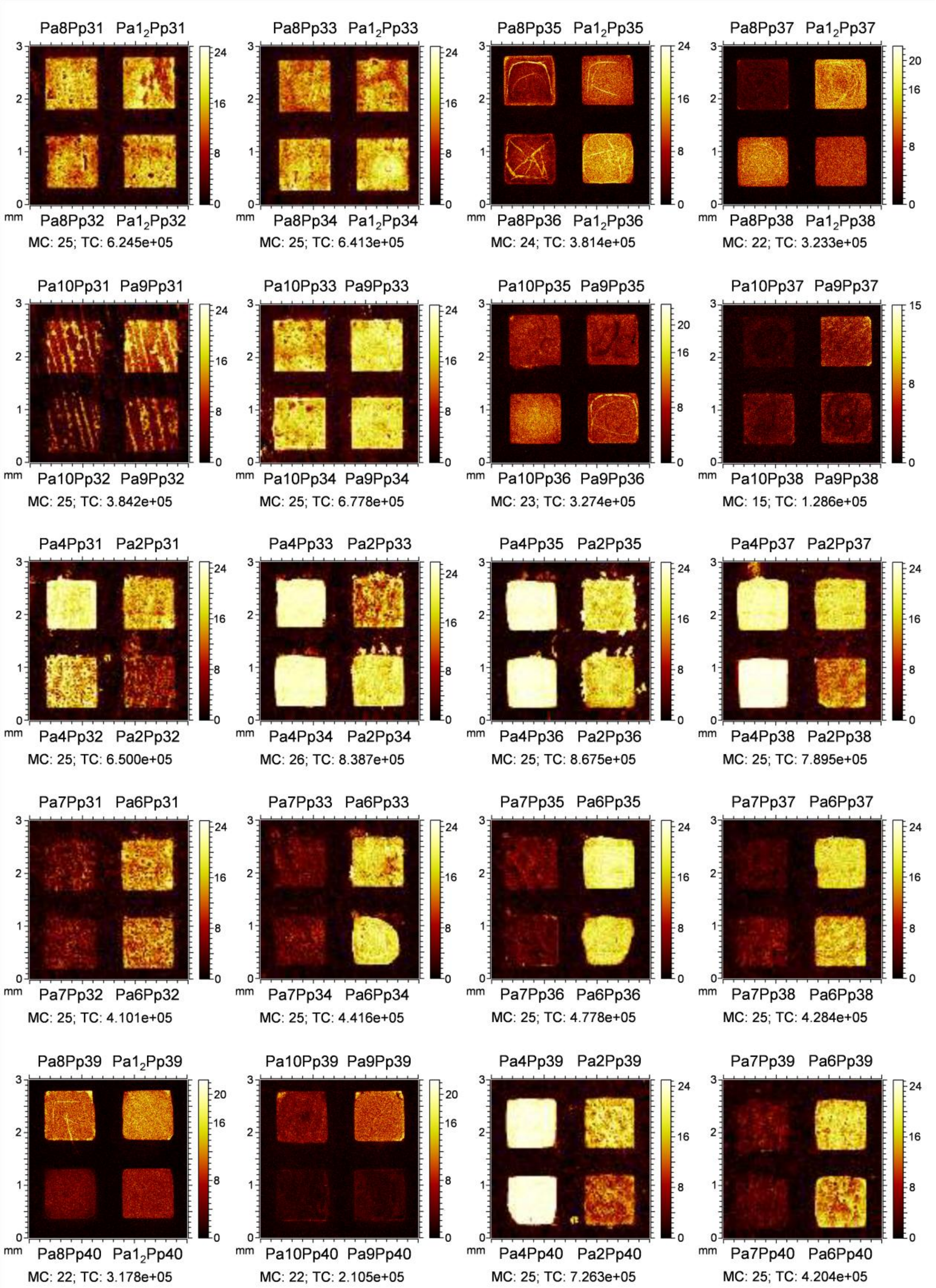

Figure S13. Screening of coating stability using ToF-SIMS (CNO^- signal): imaging of coatings made of **Pa1$_2$**, **Pa2**, **Pa4**, and **Pa6-Pa10** crosslinked with **Pp31-Pp40**, respectively. Each coating has a dimension of 1 mm × 1 mm. The color scale represents the CNO^- intensities (ion counts). MC = maximum counts in a single pixel. TC = total counts across the entire image.

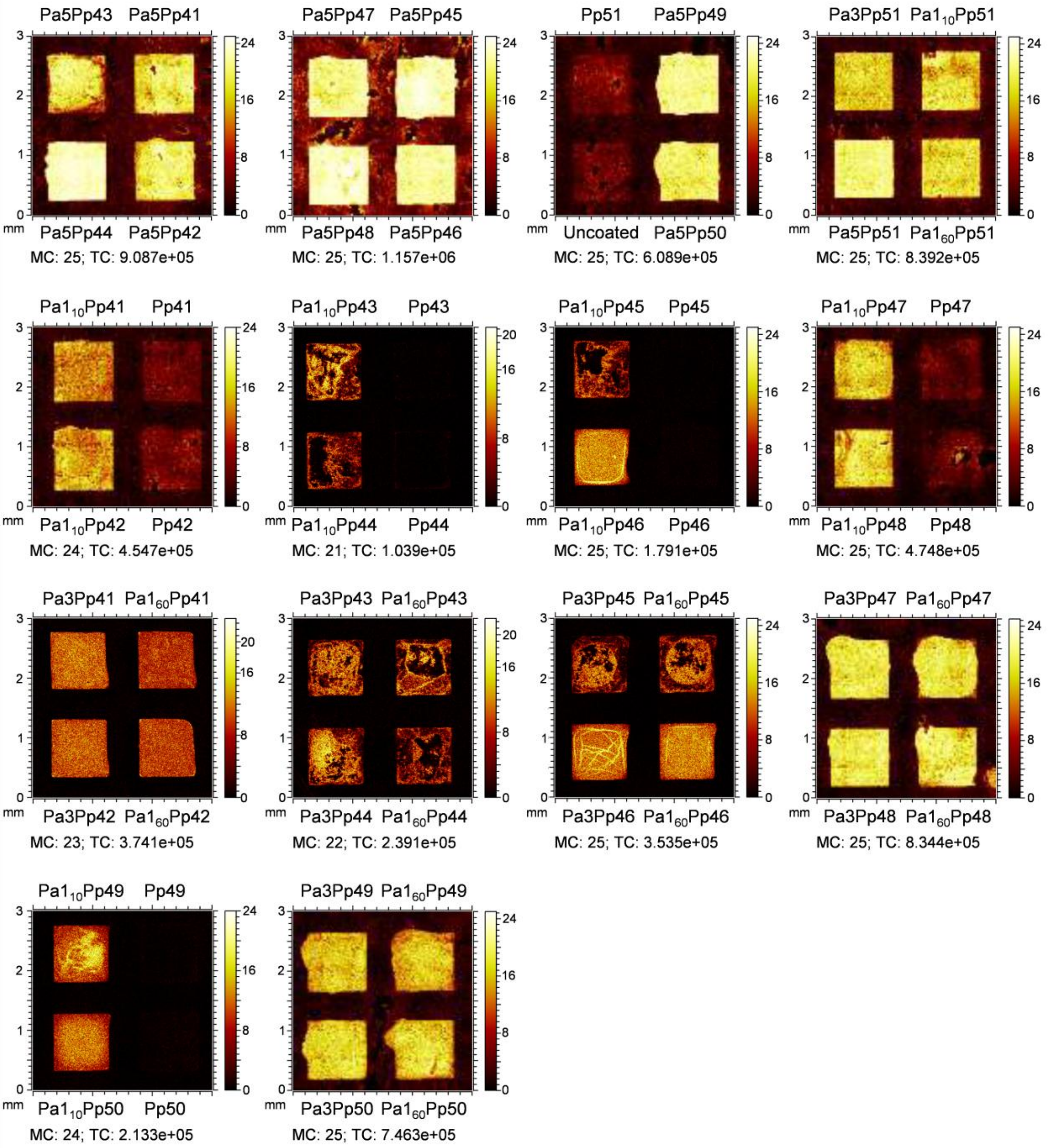

Figure S14. Screening of coating stability using ToF-SIMS (CNO^- signal): imaging of coatings made of **Pa1$_{10}$**, **Pa1$_{60}$**, **Pa3**, and **Pa5** crosslinked with **Pp41-Pp51**, respectively. Images of the individual precursors **Pp41-Pp51** are also included. Uncoated spots serve as reference controls. Each coating has a dimension of 1 mm × 1 mm. The color scale represents the CNO^- intensities (ion counts). MC = maximum counts in a single pixel. TC = total counts across the entire image.

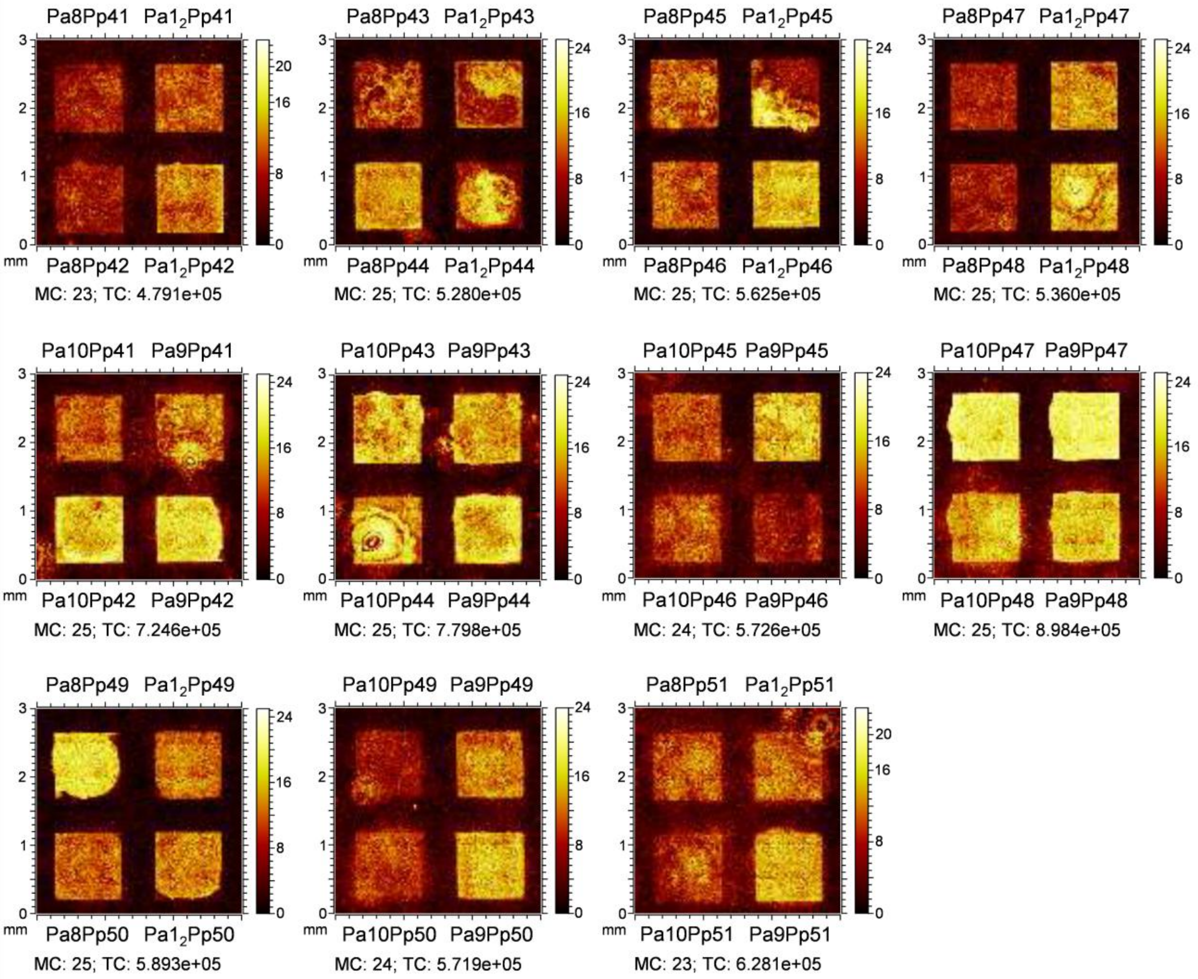

Figure S15. Screening of coating stability using ToF-SIMS (CNO^- signal): imaging of coatings made of **Pa1$_2$** and **Pa8-Pa10** crosslinked with **Pp41-Pp51**, respectively. Each coating has a dimension of 1 mm × 1 mm. The color scale represents the CNO^- intensities (ion counts). MC = maximum counts in a single pixel. TC = total counts across the entire image.

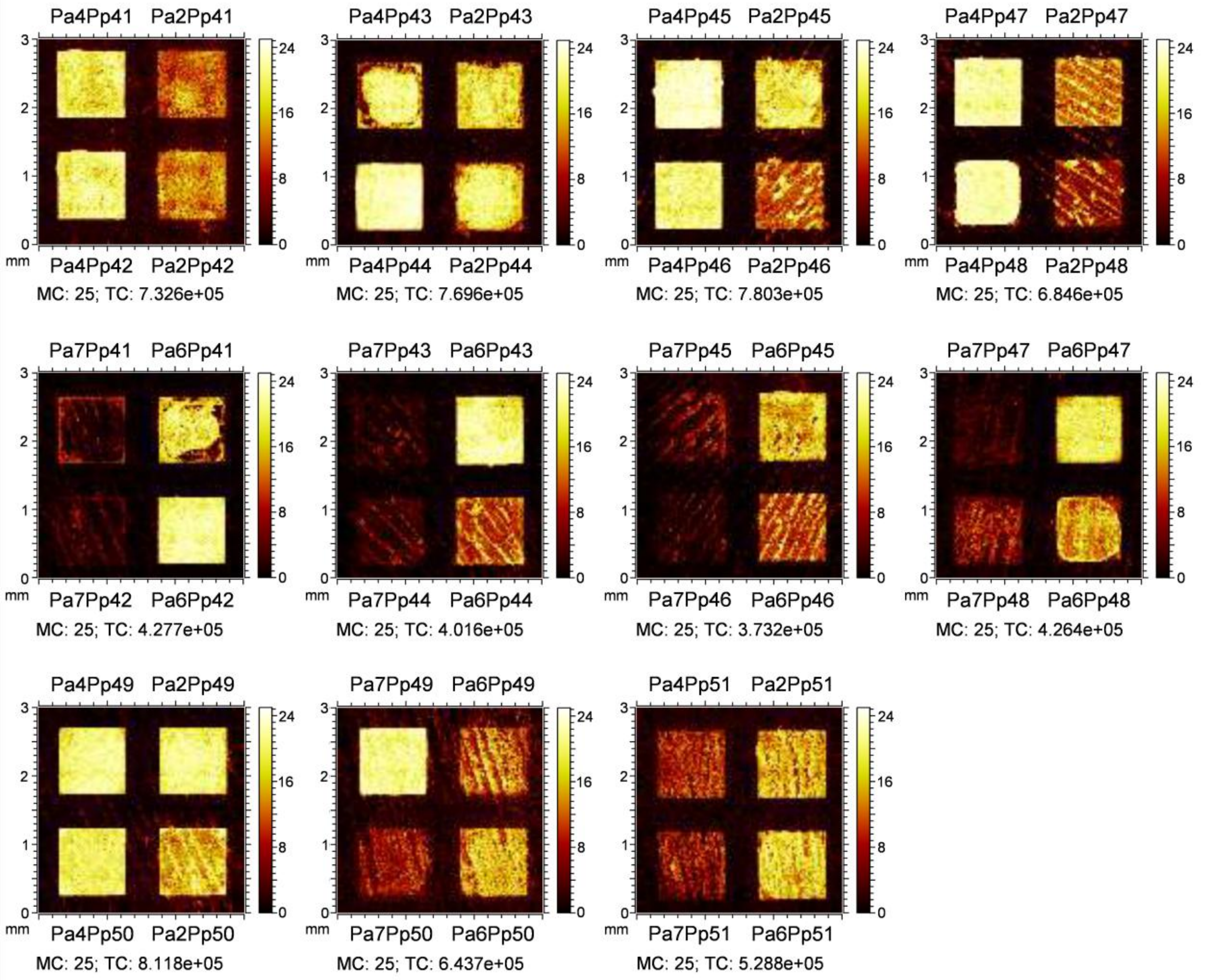

Figure S16. Screening of coating stability using ToF-SIMS (CNO⁻ signal): imaging of coatings made of **Pa2**, **Pa4**, **Pa6**, and **Pa7** crosslinked with **Pp41-Pp51**, respectively. Each coating has a dimension of 1 mm × 1 mm. The color scale represents the CNO⁻ intensities (ion counts). MC = maximum counts in a single pixel. TC = total counts across the entire image.

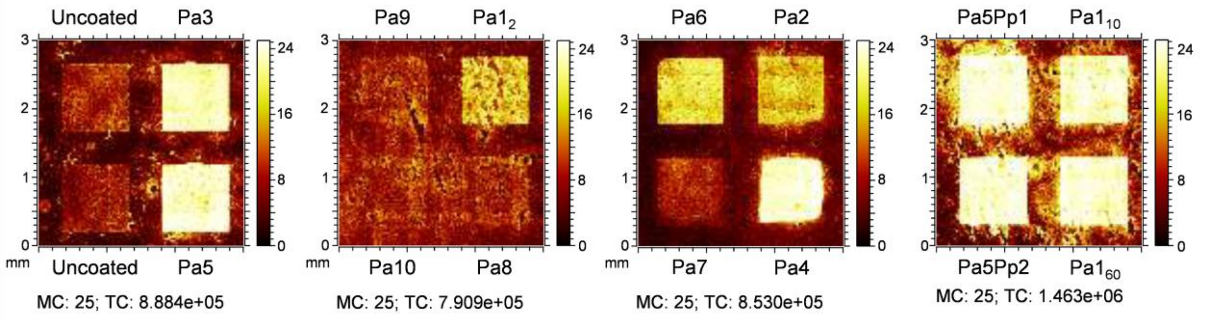

Figure S17. Screening of coating stability using ToF-SIMS (CN⁻ signal): imaging of individual precursors **Pa1-Pa10**, randomly distributed on the DMA. Uncoated spots serve as reference controls. Spot size: 1 mm × 1 mm. The color scale represents the CN⁻ intensities (ion counts). MC = maximum counts in a single pixel. TC = total counts across the entire image.

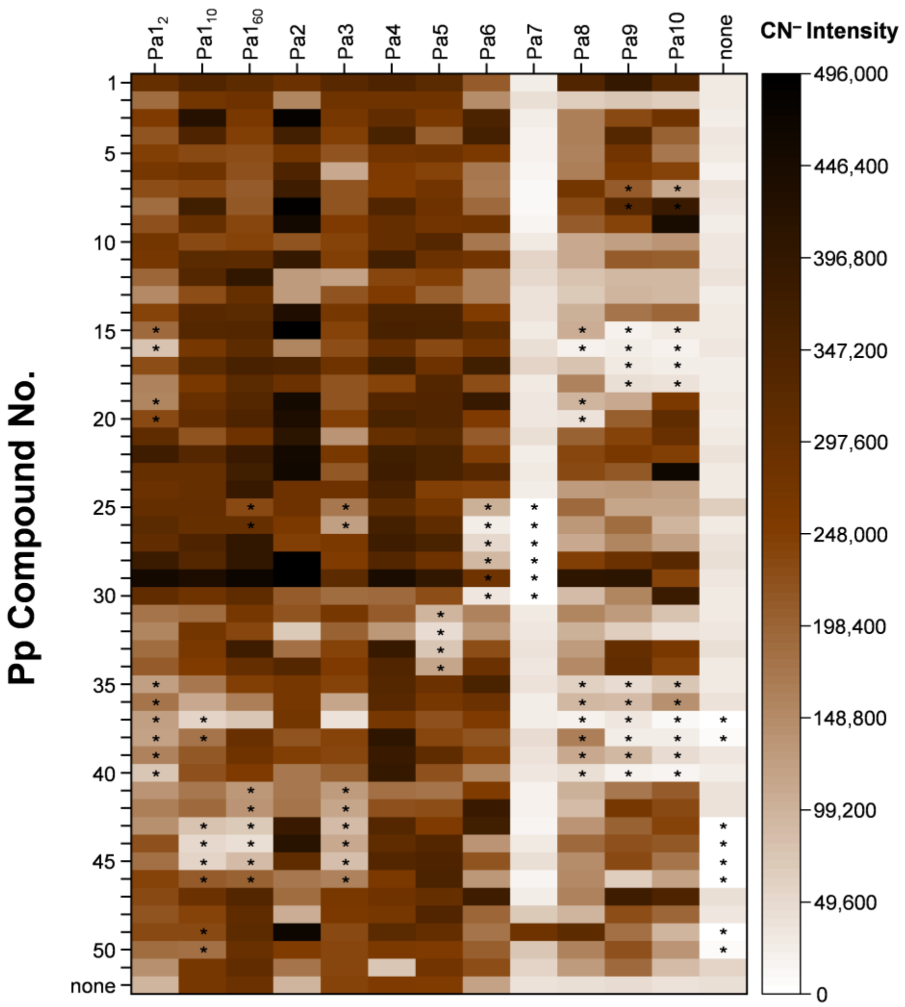

Figure S18. Stability screening of **PaPp** coatings analyzed by ToF-SIMS. Heatmap of CN^- count intensities for all 612 **PaPp** combinations (12 **Pa** × 51 **Pp**) and their respective precursors, illustrating coating stability across the library. Measurements were performed on ITO-coated DMAs, except for coatings marked with an asterisk (*), which were measured on standard DMAs.

Table S1. Ion count intensities of CNO^- and CN^- from **PaPp** coatings measured by ToF-SIMS, reflecting the coating stability. Data are shown for uncoated ITO-DMA and DMA (controls), precursor **Pa**, precursor **Pp**, and **PaPp** coatings formed by crosslinking **Pa** with **Pp1-Pp10**, respectively. Measurements were performed on ITO-coated DMAs, except for coatings marked with an asterisk (*), which were measured on standard DMAs.

Coatings	CNO^-	CN^-
Uncoated ITO-DMA	24971	43912
Uncoated DMA	703	1274
$Pa1_2$	34082	96296
$Pa1_{10}$	114646	267026
$Pa1_{60}$	106576	291898
Pa2	31571	95398
Pa3	97865	241586
Pa4	175290	255555
Pa5	153491	248568
Pa6	49955	118521
Pa7	18977	39438
Pa8	15156	40852
Pa9	17625	45193
Pa10	15310	40126
Pp1	14856	30881
Pp2	15116	28762
Pp3	10996	23779
Pp4	17356	35796
Pp5	13546	29333
Pp6	9803	19220
Pp7	16877	39679
Pp8	14635	34418
Pp9	11743	25955
Pp10	15855	34538
Pp11	18785	31883
Pp12	36865	37439
Pp13	13203	24260
Pp14	18299	28337
Pp15	18867	29747
Pp16	22004	35656
Pp17	13519	24639
Pp18	13839	25655
Pp19	14623	27559
Pp20	14067	22891
Pp21	14468	29206
Pp22	24602	36999
Pp23	14162	27252
Pp24	17208	27747
Pp25	48428	67494
Pp26	15382	27132
Pp27	23305	40474
Pp28	19329	43169
Pp29	18898	34752
Pp30	18459	31082
Pp31	17811	28158
Pp32	23421	32101
Pp33	26886	42530
Pp34	19978	29736
Pp35	18163	28767
Pp36	16948	37556
Pp37*	872	2474
Pp38*	2346	6279
Pp39	16540	32208
Pp40	14665	25242
Pp41	20066	38665
Pp42	21781	40447
Pp43*	561	1455
Pp44*	502	1393
Pp45*	606	1639
Pp46*	1348	3986
Pp47	15650	44321
Pp48	17149	35257
Pp49*	933	2967
Pp50*	1665	4863
Pp51	25041	56005

Coatings	CNO^-	CN^-
$Pa1_2Pp1$	95520	299641
$Pa1_{10}Pp1$	115994	341835
$Pa1_{60}Pp1$	169833	319553
Pa2Pp1	105984	288838
Pa3Pp1	175219	327121
Pa4Pp1	248139	346062
Pa5Pp1	223990	323092
Pa6Pp1	104760	208215
Pa7Pp1	11428	26465
Pa8Pp1	107943	342500
Pa9Pp1	100071	394082
Pa10Pp1	105863	330908
$Pa1_2Pp2$	76821	188506
$Pa1_{10}Pp2$	125140	274364
$Pa1_{60}Pp2$	141760	284539
Pa2Pp2	62220	153662
Pa3Pp2	137473	282263
Pa4Pp2	201970	281864
Pa5Pp2	203642	283887
Pa6Pp2	70966	145839
Pa7Pp2	22636	44242
Pa8Pp2	27114	63836
Pa9Pp2	34806	74965
Pa10Pp2	31087	67244
$Pa1_2Pp3$	102187	256001
$Pa1_{10}Pp3$	177040	419992
$Pa1_{60}Pp3$	122450	262672
Pa2Pp3	217295	482610
Pa3Pp3	126758	268186
Pa4Pp3	213077	308373
Pa5Pp3	150960	263730
Pa6Pp3	164932	349081
Pa7Pp3	11432	24441
Pa8Pp3	90822	165926
Pa9Pp3	124124	231589
Pa10Pp3	143148	282280
$Pa1_2Pp4$	77327	219481
$Pa1_{10}Pp4$	125944	344009
$Pa1_{60}Pp4$	99402	243546
Pa2Pp4	203240	368966
Pa3Pp4	101505	247002
Pa4Pp4	237319	353277
Pa5Pp4	103749	203463
Pa6Pp4	159914	361783
Pa7Pp4	11025	21674
Pa8Pp4	63358	163036
Pa9Pp4	143224	332073
Pa10Pp4	71589	201245
$Pa1_2Pp5$	77281	246753
$Pa1_{10}Pp5$	75174	232677
$Pa1_{60}Pp5$	95174	225737
Pa2Pp5	90698	274776
Pa3Pp5	90971	217857
Pa4Pp5	162376	276899
Pa5Pp5	158220	282827
Pa6Pp5	111134	257134
Pa7Pp5	8945	18113
Pa8Pp5	50940	160217
Pa9Pp5	97064	276591
Pa10Pp5	53073	174337

Coatings	CNO^-	CN^-
$Pa1_2Pp6$	84295	266244
$Pa1_{10}Pp6$	93124	282324
$Pa1_{60}Pp6$	91480	221040
Pa2Pp6	114995	344371
Pa3Pp6	35443	112410
Pa4Pp6	142960	252538
Pa5Pp6	130156	240140
Pa6Pp6	77847	172027
Pa7Pp6	6192	15681
Pa8Pp6	55058	165427
Pa9Pp6	86532	259272
Pa10Pp6	73261	240950
$Pa1_2Pp7$	68004	226970
$Pa1_{10}Pp7$	74359	238104
$Pa1_{60}Pp7$	94004	211492
Pa2Pp7	125657	376024
Pa3Pp7	97601	216594
Pa4Pp7	141918	251158
Pa5Pp7	161852	278834
Pa6Pp7	63669	169927
Pa7Pp7	4274	11028
Pa8Pp7	103693	272023
Pa9Pp7*	88122	209867
Pa10Pp7*	43597	116402
$Pa1_2Pp8$	61434	185707
$Pa1_{10}Pp8$	143808	374079
$Pa1_{60}Pp8$	92133	213510
Pa2Pp8	237475	489976
Pa3Pp8	95667	217252
Pa4Pp8	214285	338227
Pa5Pp8	177900	286065
Pa6Pp8	82018	191393
Pa7Pp8	5764	12381
Pa8Pp8	82604	231184
Pa9Pp8*	139123	327670
Pa10Pp8*	135077	384173
$Pa1_2Pp9$	78064	224688
$Pa1_{10}Pp9$	107176	298796
$Pa1_{60}Pp9$	99580	234750
Pa2Pp9	186773	459021
Pa3Pp9	111810	256906
Pa4Pp9	179556	303837
Pa5Pp9	164486	277798
Pa6Pp9	120783	280975
Pa7Pp9	6738	15357
Pa8Pp9	78117	209055
Pa9Pp9	76640	241538
Pa10Pp9	153985	446729
$Pa1_2Pp10$	85852	271527
$Pa1_{10}Pp10$	76866	233175
$Pa1_{60}Pp10$	114284	239868
Pa2Pp10	78512	220098
Pa3Pp10	112211	239019
Pa4Pp10	185701	302857
Pa5Pp10	218913	329824
Pa6Pp10	81511	174187
Pa7Pp10	13824	30624
Pa8Pp10	38094	110281
Pa9Pp10	47277	126245
Pa10Pp10	44781	137820

Table S2. Ion count intensities of CNO^- and CN^- from **PaPp** coatings measured by ToF-SIMS, reflecting the coating stability. Data are shown for **PaPp** coatings formed by crosslinking **Pa** with **Pp11-Pp25**, respectively. Measurements were performed on ITO-coated DMAs, except for coatings marked with an asterisk (*), which were measured on standard DMAs.

Coatings	CNO^-	CN^-
Pa1$_{2}$Pp11	109284	270234
Pa1$_{10}$Pp11	127346	323962
Pa1$_{60}$Pp11	126662	317310
Pa2Pp11	143472	392590
Pa3Pp11	125543	244422
Pa4Pp11	247843	367393
Pa5Pp11	175139	291192
Pa6Pp11	123620	278215
Pa7Pp11	29000	57628
Pa8Pp11	49005	112142
Pa9Pp11	78480	208405
Pa10Pp11	66627	203867
Pa1$_{2}$Pp12	95897	196358
Pa1$_{10}$Pp12	167277	331996
Pa1$_{60}$Pp12	195036	399532
Pa2Pp12	51742	130335
Pa3Pp12	50239	121512
Pa4Pp12	160458	236856
Pa5Pp12	180365	244728
Pa6Pp12	83406	166282
Pa7Pp12	38063	52618
Pa8Pp12	54505	79888
Pa9Pp12	59655	90505
Pa10Pp12	60704	94562
Pa1$_{2}$Pp13	55788	152835
Pa1$_{10}$Pp13	92171	228064
Pa1$_{60}$Pp13	119002	298095
Pa2Pp13	41543	128861
Pa3Pp13	94877	217453
Pa4Pp13	174790	254882
Pa5Pp13	128022	204594
Pa6Pp13	71492	164997
Pa7Pp13	21408	37370
Pa8Pp13	24071	68186
Pa9Pp13	42317	99117
Pa10Pp13	38388	92935
Pa1$_{2}$Pp14	79621	241145
Pa1$_{10}$Pp14	125220	327584
Pa1$_{60}$Pp14	120522	323493
Pa2Pp14	149094	435461
Pa3Pp14	136489	268199
Pa4Pp14	229192	347906
Pa5Pp14	240358	348308
Pa6Pp14	108786	248962
Pa7Pp14	20659	39111
Pa8Pp14	34527	103395
Pa9Pp14	58394	179804
Pa10Pp14	55534	194128
Pa1$_{2}$Pp15*	71536	190318
Pa1$_{10}$Pp15	133645	330549
Pa1$_{60}$Pp15	127553	337576
Pa2Pp15	218381	492044
Pa3Pp15	114193	240946
Pa4Pp15	239913	356646
Pa5Pp15	257977	352494
Pa6Pp15	131336	319853
Pa7Pp15	14455	27384
Pa8Pp15*	43823	107378
Pa9Pp15*	8791	21028
Pa10Pp15*	8990	30156

Coatings	CNO^-	CN^-
Pa1$_{2}$Pp16*	30302	77123
Pa1$_{10}$Pp16	117956	269235
Pa1$_{60}$Pp16	142997	319237
Pa2Pp16	61508	155852
Pa3Pp16	106780	229463
Pa4Pp16	215175	306313
Pa5Pp16	149688	231799
Pa6Pp16	125017	282233
Pa7Pp16	22558	44274
Pa8Pp16*	6408	18808
Pa9Pp16*	10001	25058
Pa10Pp16*	7369	21938
Pa1$_{2}$Pp17	97821	228071
Pa1$_{10}$Pp17	128987	322158
Pa1$_{60}$Pp17	143080	357804
Pa2Pp17	133821	347990
Pa3Pp17	149709	283428
Pa4Pp17	269778	373030
Pa5Pp17	181537	287463
Pa6Pp17	176703	370689
Pa7Pp17	32563	57593
Pa8Pp17	36576	81149
Pa9Pp17*	11367	27475
Pa10Pp17*	7242	22642
Pa1$_{2}$Pp18	59146	159425
Pa1$_{10}$Pp18	98164	263274
Pa1$_{60}$Pp18	114232	323652
Pa2Pp18	97568	292947
Pa3Pp18	106431	218561
Pa4Pp18	146185	239931
Pa5Pp18	216015	337893
Pa6Pp18	94293	227293
Pa7Pp18	16095	30377
Pa8Pp18	60974	157856
Pa9Pp18*	18121	43889
Pa10Pp18*	12685	40463
Pa1$_{2}$Pp19*	55849	157209
Pa1$_{10}$Pp19	117161	293574
Pa1$_{60}$Pp19	128539	329294
Pa2Pp19	178414	452863
Pa3Pp19	109561	217080
Pa4Pp19	226825	334709
Pa5Pp19	256448	340496
Pa6Pp19	174538	389228
Pa7Pp19	16112	31806
Pa8Pp19*	44424	96530
Pa9Pp19	49857	111105
Pa10Pp19	94012	261025
Pa1$_{2}$Pp20*	87378	234243
Pa1$_{10}$Pp20	136691	309018
Pa1$_{60}$Pp20	147414	342877
Pa2Pp20	142930	439233
Pa3Pp20	141895	247880
Pa4Pp20	257942	355874
Pa5Pp20	237121	341814
Pa6Pp20	112884	253508
Pa7Pp20	19849	35154
Pa8Pp20*	14774	36158
Pa9Pp20	89104	205308
Pa10Pp20	113137	315621

Coatings	CNO^-	CN^-
Pa1$_{2}$Pp21	109489	314963
Pa1$_{10}$Pp21	77677	219056
Pa1$_{60}$Pp21	102059	284281
Pa2Pp21	137477	412759
Pa3Pp21	53473	139449
Pa4Pp21	199987	299909
Pa5Pp21	201060	323499
Pa6Pp21	87025	209677
Pa7Pp21	18899	41737
Pa8Pp21	76855	198723
Pa9Pp21	93691	239565
Pa10Pp21	117113	295952
Pa1$_{2}$Pp22	133459	376872
Pa1$_{10}$Pp22	122702	332261
Pa1$_{60}$Pp22	144909	387455
Pa2Pp22	160329	459147
Pa3Pp22	131248	261920
Pa4Pp22	246021	370214
Pa5Pp22	220395	353209
Pa6Pp22	93638	246122
Pa7Pp22	15395	26324
Pa8Pp22	85090	236009
Pa9Pp22	98711	262623
Pa10Pp22	86130	247911
Pa1$_{2}$Pp23	91723	299539
Pa1$_{10}$Pp23	97588	298458
Pa1$_{60}$Pp23	112242	373395
Pa2Pp23	164262	463543
Pa3Pp23	71335	215224
Pa4Pp23	239098	374551
Pa5Pp23	211058	353638
Pa6Pp23	124228	326381
Pa7Pp23	13408	31107
Pa8Pp23	77633	232676
Pa9Pp23	74448	215539
Pa10Pp23	179721	466709
Pa1$_{2}$Pp24	82021	290574
Pa1$_{10}$Pp24	99367	301964
Pa1$_{60}$Pp24	126232	389022
Pa2Pp24	78524	285048
Pa3Pp24	136157	285582
Pa4Pp24	243801	356981
Pa5Pp24	143839	244339
Pa6Pp24	102949	240565
Pa7Pp24	14138	24322
Pa8Pp24	44348	126853
Pa9Pp24	53093	134990
Pa10Pp24	43751	125091
Pa1$_{2}$Pp25	101095	305358
Pa1$_{10}$Pp25	105999	302928
Pa1$_{60}$Pp25*	96252	230976
Pa2Pp25	104930	284110
Pa3Pp25*	89685	173390
Pa4Pp25	214896	316767
Pa5Pp25	162298	273332
Pa6Pp25*	51480	102083
Pa7Pp25*	2402	4381
Pa8Pp25	85881	190889
Pa9Pp25	57916	115077
Pa10Pp25	50387	117210

Table S3. Ion count intensities of CNO^- and CN^- from **PaPp** coatings measured by ToF-SIMS, reflecting the coating stability. Data are shown for **PaPp** coatings formed by crosslinking **Pa** with **Pp26-Pp40**, respectively. Measurements were performed on ITO-coated DMAs, except for coatings marked with an asterisk (*), which were measured on standard DMAs.

Coatings	CNO^-	CN^-
Pa1$_{2}$Pp26	95889	321083
Pa1$_{10}$Pp26	95367	298209
Pa1$_{60}$Pp26*	95531	299867
Pa2Pp26	100294	261120
Pa3Pp26*	59000	123747
Pa4Pp26	262239	361200
Pa5Pp26	204615	312090
Pa6Pp26*	9474	25547
Pa7Pp26*	1292	3465
Pa8Pp26	49785	134909
Pa9Pp26	64232	185474
Pa10Pp26	25130	96047
Pa1$_{2}$Pp27	94052	307789
Pa1$_{10}$Pp27	111525	346670
Pa1$_{60}$Pp27	120978	400099
Pa2Pp27	104064	247114
Pa3Pp27	110265	258742
Pa4Pp27	272371	378189
Pa5Pp27	223851	352701
Pa6Pp27*	18700	51557
Pa7Pp27*	1058	2396
Pa8Pp27	38182	110121
Pa9Pp27	60253	154755
Pa10Pp27	38698	126069
Pa1$_{2}$Pp28	118485	378907
Pa1$_{10}$Pp28	99978	331054
Pa1$_{60}$Pp28	122734	397661
Pa2Pp28	146959	494012
Pa3Pp28	99997	249543
Pa4Pp28	199281	337199
Pa5Pp28	143113	285820
Pa6Pp28*	35135	90283
Pa7Pp28*	1300	3007
Pa8Pp28	70836	244653
Pa9Pp28	92140	292178
Pa10Pp28	102606	327061
Pa1$_{2}$Pp29	143293	459504
Pa1$_{10}$Pp29	153173	437596
Pa1$_{60}$Pp29	160224	469776
Pa2Pp29	261080	495459
Pa3Pp29	140891	318368
Pa4Pp29	287530	444245
Pa5Pp29	245805	398194
Pa6Pp29*	121921	279709
Pa7Pp29*	1487	4405
Pa8Pp29	122401	406748
Pa9Pp29	121202	410844
Pa10Pp29	63001	240520
Pa1$_{2}$Pp30	99479	306782
Pa1$_{10}$Pp30	90151	282738
Pa1$_{60}$Pp30	95046	312194
Pa2Pp30	74003	211670
Pa3Pp30	69602	187814
Pa4Pp30	113305	192163
Pa5Pp30	132234	229643
Pa6Pp30*	11339	32924
Pa7Pp30*	1261	4209
Pa8Pp30	42224	89342
Pa9Pp30	66126	155831
Pa10Pp30	100278	380154

Coatings	CNO^-	CN^-
Pa1$_{2}$Pp31	87820	178840
Pa1$_{10}$Pp31	91552	185215
Pa1$_{60}$Pp31	125002	269270
Pa2Pp31	83201	217774
Pa3Pp31	164455	267461
Pa4Pp31	154917	211396
Pa5Pp31*	61650	97216
Pa6Pp31	68074	158801
Pa7Pp31	17976	31249
Pa8Pp31	88374	154864
Pa9Pp31	66599	128337
Pa10Pp31	39794	80557
Pa1$_{2}$Pp32	80513	155556
Pa1$_{10}$Pp32	152213	272165
Pa1$_{60}$Pp32	99002	234861
Pa2Pp32	30148	71900
Pa3Pp32	102536	199304
Pa4Pp32	94557	133301
Pa5Pp32*	26002	46349
Pa6Pp32	64410	134089
Pa7Pp32	21357	35452
Pa8Pp32	72332	99644
Pa9Pp32	28914	65023
Pa10Pp32	15690	36263
Pa1$_{2}$Pp33	68909	188850
Pa1$_{10}$Pp33	94411	261891
Pa1$_{60}$Pp33	134856	377790
Pa2Pp33	71359	184323
Pa3Pp33	112677	241458
Pa4Pp33	284659	388921
Pa5Pp33*	50349	73297
Pa6Pp33	100913	229931
Pa7Pp33	18135	37494
Pa8Pp33	65228	130434
Pa9Pp33	124755	305331
Pa10Pp33	89824	264878
Pa1$_{2}$Pp34	103471	211550
Pa1$_{10}$Pp34	112929	249556
Pa1$_{60}$Pp34	128752	302187
Pa2Pp34	136814	333639
Pa3Pp34	159564	252557
Pa4Pp34	280433	340777
Pa5Pp34*	81988	116981
Pa6Pp34	132382	291932
Pa7Pp34	19299	33012
Pa8Pp34	82764	149340
Pa9Pp34	118603	306882
Pa10Pp34	97506	242233
Pa1$_{2}$Pp35*	51198	125466
Pa1$_{10}$Pp35	79749	175013
Pa1$_{60}$Pp35	115963	246179
Pa2Pp35	114473	267495
Pa3Pp35	148521	241631
Pa4Pp35	261942	331146
Pa5Pp35	272362	292618
Pa6Pp35	167403	354040
Pa7Pp35	20667	36229
Pa8Pp35*	25614	58672
Pa9Pp35*	22589	48736
Pa10Pp35*	29716	76501

Coatings	CNO^-	CN^-
Pa1$_{2}$Pp36*	74024	179150
Pa1$_{10}$Pp36	48008	110058
Pa1$_{60}$Pp36	60049	163623
Pa2Pp36	112221	266383
Pa3Pp36	57083	114754
Pa4Pp36	269475	326365
Pa5Pp36	203649	266472
Pa6Pp36	123940	290430
Pa7Pp36	14541	26190
Pa8Pp36*	37793	91652
Pa9Pp36*	36220	86623
Pa10Pp36*	52652	141674
Pa1$_{2}$Pp37*	52261	122411
Pa1$_{10}$Pp37*	26986	57455
Pa1$_{60}$Pp37	38322	73476
Pa2Pp37	121138	271852
Pa3Pp37	20027	36123
Pa4Pp37	202887	268457
Pa5Pp37	161521	224701
Pa6Pp37	110915	253148
Pa7Pp37	11013	25847
Pa8Pp37*	8701	21851
Pa9Pp37*	15565	32876
Pa10Pp37*	4059	11113
Pa1$_{2}$Pp38*	37894	119209
Pa1$_{10}$Pp38*	60121	178376
Pa1$_{60}$Pp38	110304	297455
Pa2Pp38	64848	220416
Pa3Pp38	117400	242580
Pa4Pp38	315753	410261
Pa5Pp38	155136	237038
Pa6Pp38	93565	216438
Pa7Pp38	18648	47630
Pa8Pp38*	53651	166138
Pa9Pp38*	8430	26113
Pa10Pp38*	7933	23336
Pa1$_{2}$Pp39*	59314	159395
Pa1$_{10}$Pp39	100827	216291
Pa1$_{60}$Pp39	130058	280127
Pa2Pp39	107443	244347
Pa3Pp39	158300	234599
Pa4Pp39	276985	384684
Pa5Pp39	231587	313530
Pa6Pp39	109426	242740
Pa7Pp39	18213	38270
Pa8Pp39*	46961	108456
Pa9Pp39*	39813	91328
Pa10Pp39*	19531	49092
Pa1$_{2}$Pp40*	24884	72572
Pa1$_{10}$Pp40	77888	222200
Pa1$_{60}$Pp40	78227	256868
Pa2Pp40	52168	173925
Pa3Pp40	116673	204888
Pa4Pp40	299986	396614
Pa5Pp40	135275	224120
Pa6Pp40	71412	156330
Pa7Pp40	14601	34201
Pa8Pp40*	18256	41212
Pa9Pp40*	7085	19541
Pa10Pp40*	5603	15599

Table S4. Ion count intensities of CNO^- and CN^- from **PaPp** coatings measured by ToF-SIMS, reflecting the coating stability. Data are shown for **PaPp** coatings formed by crosslinking **Pa** with **Pp41-Pp51**, respectively. Measurements were performed on ITO-coated DMAs, except for coatings marked with an asterisk (*), which were measured on standard DMAs.

Coatings	CNO^-	CN^-
$Pa1_2Pp41$	46973	138179
$Pa1_{10}Pp41$	65175	175084
$Pa1_{60}Pp41$*	40551	130975
Pa2Pp41	64577	175395
Pa3Pp41*	55305	127097
Pa4Pp41	119501	183806
Pa5Pp41	123597	180141
Pa6Pp41	118354	252028
Pa7Pp41	7512	19409
Pa8Pp41	34107	103196
Pa9Pp41	61608	175301
Pa10Pp41	54700	207744
$Pa1_2Pp42$	56540	164475
$Pa1_{10}Pp42$	76438	192235
$Pa1_{60}Pp42$*	44566	139666
Pa2Pp42	72517	178685
Pa3Pp42*	51110	115416
Pa4Pp42	156403	221831
Pa5Pp42	148286	227858
Pa6Pp42	182970	387382
Pa7Pp42	8799	20103
Pa8Pp42	32812	89063
Pa9Pp42	106682	268960
Pa10Pp42	85767	230957
$Pa1_2Pp43$	52210	142275
$Pa1_{10}Pp43$*	30711	79656
$Pa1_{60}Pp43$*	25869	68186
Pa2Pp43	125837	387190
Pa3Pp43*	38443	87534
Pa4Pp43	169928	329778
Pa5Pp43	136209	256145
Pa6Pp43	174867	371898
Pa7Pp43	7461	15372
Pa8Pp43	48019	135571
Pa9Pp43	76685	200506
Pa10Pp43	83477	243169
$Pa1_2Pp44$	86461	221805
$Pa1_{10}Pp44$*	17878	52625
$Pa1_{60}Pp44$*	16293	44840
Pa2Pp44	162391	417511
Pa3Pp44*	48276	110249
Pa4Pp44	208656	311437
Pa5Pp44	236239	336681
Pa6Pp44	53700	136405
Pa7Pp44	10035	28819
Pa8Pp44	73518	191301
Pa9Pp44	85111	224021
Pa10Pp44	94728	212735
$Pa1_2Pp45$	80862	184257
$Pa1_{10}Pp45$*	20782	54797
$Pa1_{60}Pp45$*	34553	85973
Pa2Pp45	125478	311211
Pa3Pp45*	39346	85157
Pa4Pp45	237405	334697
Pa5Pp45	225430	343832
Pa6Pp45	82103	219232
Pa7Pp45	14406	41829
Pa8Pp45	58382	146322
Pa9Pp45	86135	234009
Pa10Pp45	49275	177356

Coatings	CNO^-	CN^-
$Pa1_2Pp46$	90699	240471
$Pa1_{10}Pp46$*	72036	208580
$Pa1_{60}Pp46$*	70024	202069
Pa2Pp46	67369	172876
Pa3Pp46*	69816	161830
Pa4Pp46	155304	257733
Pa5Pp46	190996	349379
Pa6Pp46	55511	135137
Pa7Pp46	7050	15945
Pa8Pp46	53487	151382
Pa9Pp46	30671	66731
Pa10Pp46	51623	120369
$Pa1_2Pp47$	74103	230139
$Pa1_{10}Pp47$	100027	289000
$Pa1_{60}Pp47$	118880	336250
Pa2Pp47	69619	188316
Pa3Pp47	123746	265827
Pa4Pp47	182906	281575
Pa5Pp47	155518	297980
Pa6Pp47	134049	374941
Pa7Pp47	8533	25337
Pa8Pp47	40428	158630
Pa9Pp47	140150	376770
Pa10Pp47	126545	346073
$Pa1_2Pp48$	93387	217436
$Pa1_{10}Pp48$	109612	242100
$Pa1_{60}Pp48$	131167	312684
Pa2Pp48	40272	107941
Pa3Pp48	124793	259492
Pa4Pp48	192181	266263
Pa5Pp48	229876	338365
Pa6Pp48	79513	195655
Pa7Pp48	30983	71103
Pa8Pp48	36080	98119
Pa9Pp48	94002	229123
Pa10Pp48	83583	196356
$Pa1_2Pp49$	66623	230253
$Pa1_{10}Pp49$*	71841	231143
$Pa1_{60}Pp49$	98962	316424
Pa2Pp49	173572	472256
Pa3Pp49	94766	232032
Pa4Pp49	165604	323313
Pa5Pp49	171665	304720
Pa6Pp49	65837	178144
Pa7Pp49	170983	279879
Pa8Pp49	114406	320033
Pa9Pp49	65850	181127
Pa10Pp49	32076	95892
$Pa1_2Pp50$	66254	188042
$Pa1_{10}Pp50$*	62974	180971
$Pa1_{60}Pp50$	117298	296064
Pa2Pp50	94746	249777
Pa3Pp50	109514	237507
Pa4Pp50	138797	259415
Pa5Pp50	141243	255826
Pa6Pp50	88322	205391
Pa7Pp50	29537	73532
Pa8Pp50	56261	161995
Pa9Pp50	84652	221154
Pa10Pp50	45268	136737

Coatings	CNO^-	CN^-
$Pa1_2Pp51$	53198	140530
$Pa1_{10}Pp51$	105562	267234
$Pa1_{60}Pp51$	116494	310664
Pa2Pp51	81644	179167
Pa3Pp51	103219	237318
Pa4Pp51	39077	72677
Pa5Pp51	155947	279227
Pa6Pp51	105315	228220
Pa7Pp51	31185	55257
Pa8Pp51	51343	127033
Pa9Pp51	76381	189816
Pa10Pp51	43799	88116

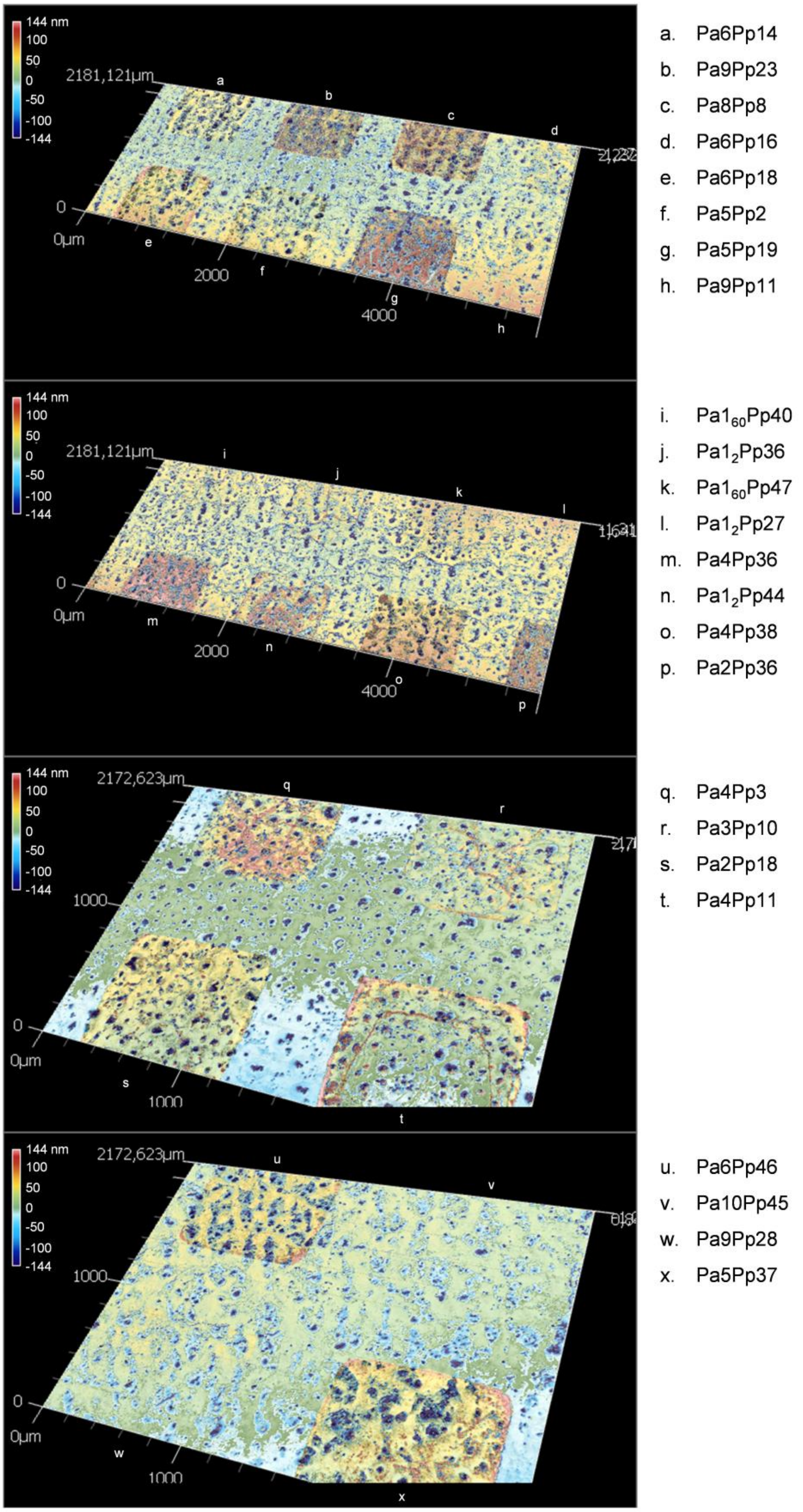

Figure S19. Three-dimensional topographical maps of representative **PaPp** coatings acquired using laser scanning microscopy (white light interferometry mode). The color scale bar indicates the relative height variation within the scanned area.

4. UHT Screening of Fluorescence Properties

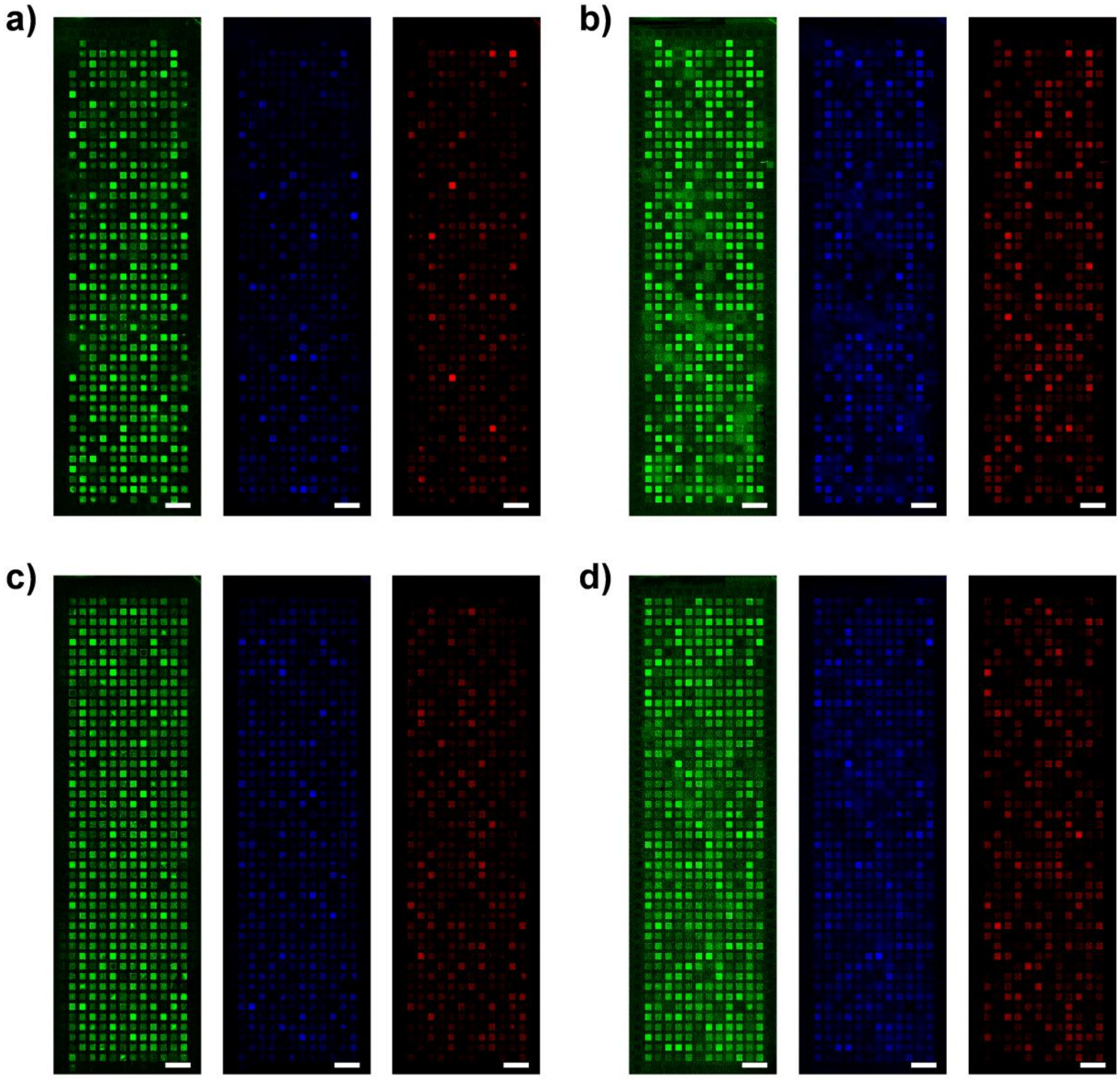

Figure S20. UHT screening of fluorescence properties: fluorescence scans of DMAs coated with 675 **PaPp** combinations (12 **Pa**, 51 **Pp**, and 612 **PaPp**). Triplicates of the 675 coatings were randomly distributed across four DMAs (a-d). Each DMA was prepared in duplicate, resulting in six replicates for each combination. a) Coatings from precursors **Pa1-Pa4**, **Pp1-Pp25**, and their corresponding crosslinked **PaPp** combinations. b) Coatings from precursors **Pa5-Pa10**, **Pp1-Pp25**, and their corresponding crosslinked **PaPp** combinations. c) Coatings from precursors **Pa1-Pa4**, **Pp26-Pp51**, and their corresponding crosslinked **PaPp** combinations. d) Coatings from precursors **Pa5-Pa10**, **Pp26-Pp51**, and their corresponding crosslinked **PaPp** combinations. Spot size: 1 mm × 1 mm. The brightness, contrast, and saturation of the images were edited for visualization purpose. Scale bar: 4 mm

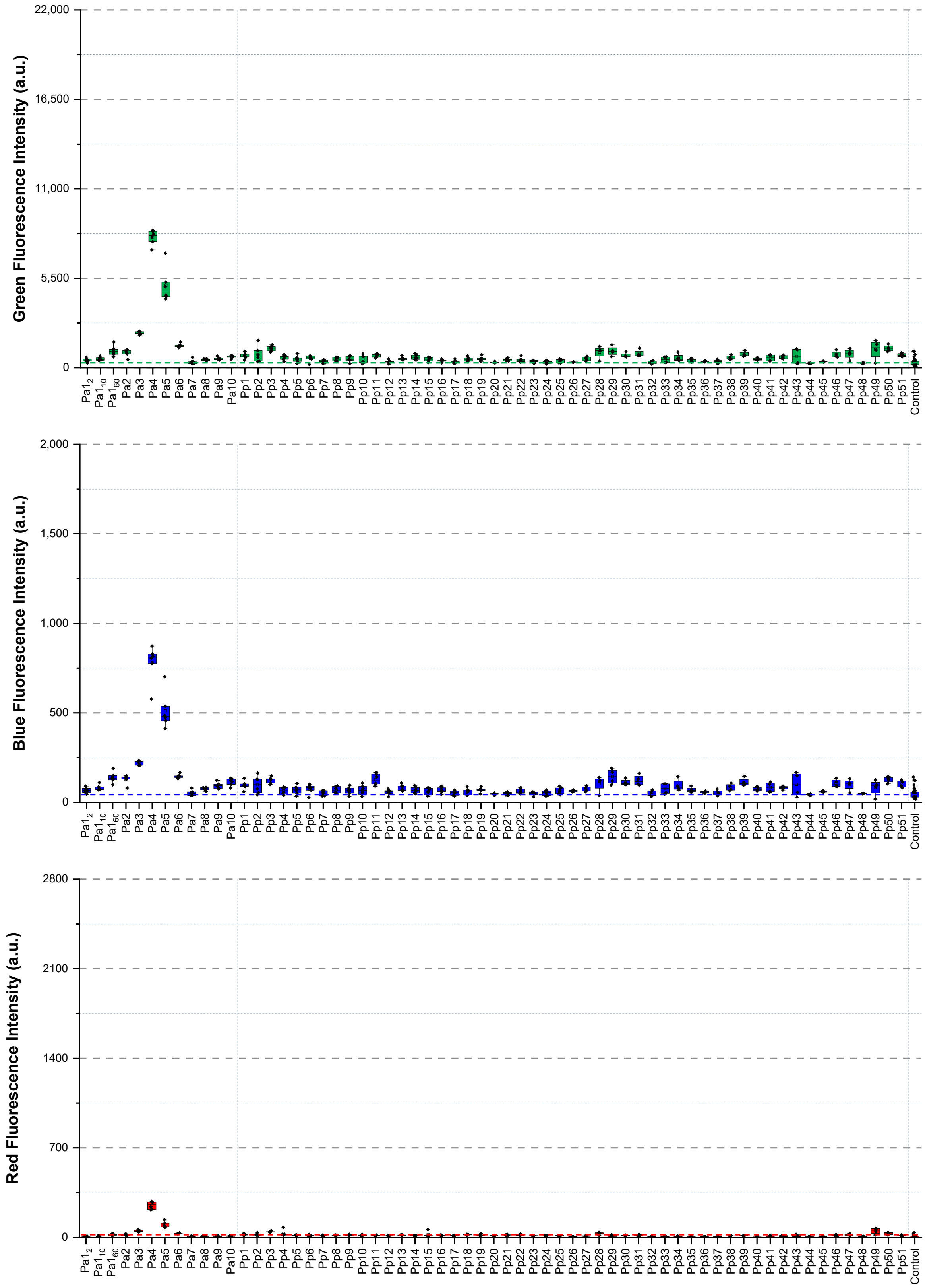

Figure S21. UHT screening of fluorescence properties: fluorescence intensities of individual precursors **Pa1₂-Pa10** and **Pp1-Pp51**. Uncoated spots served as controls.

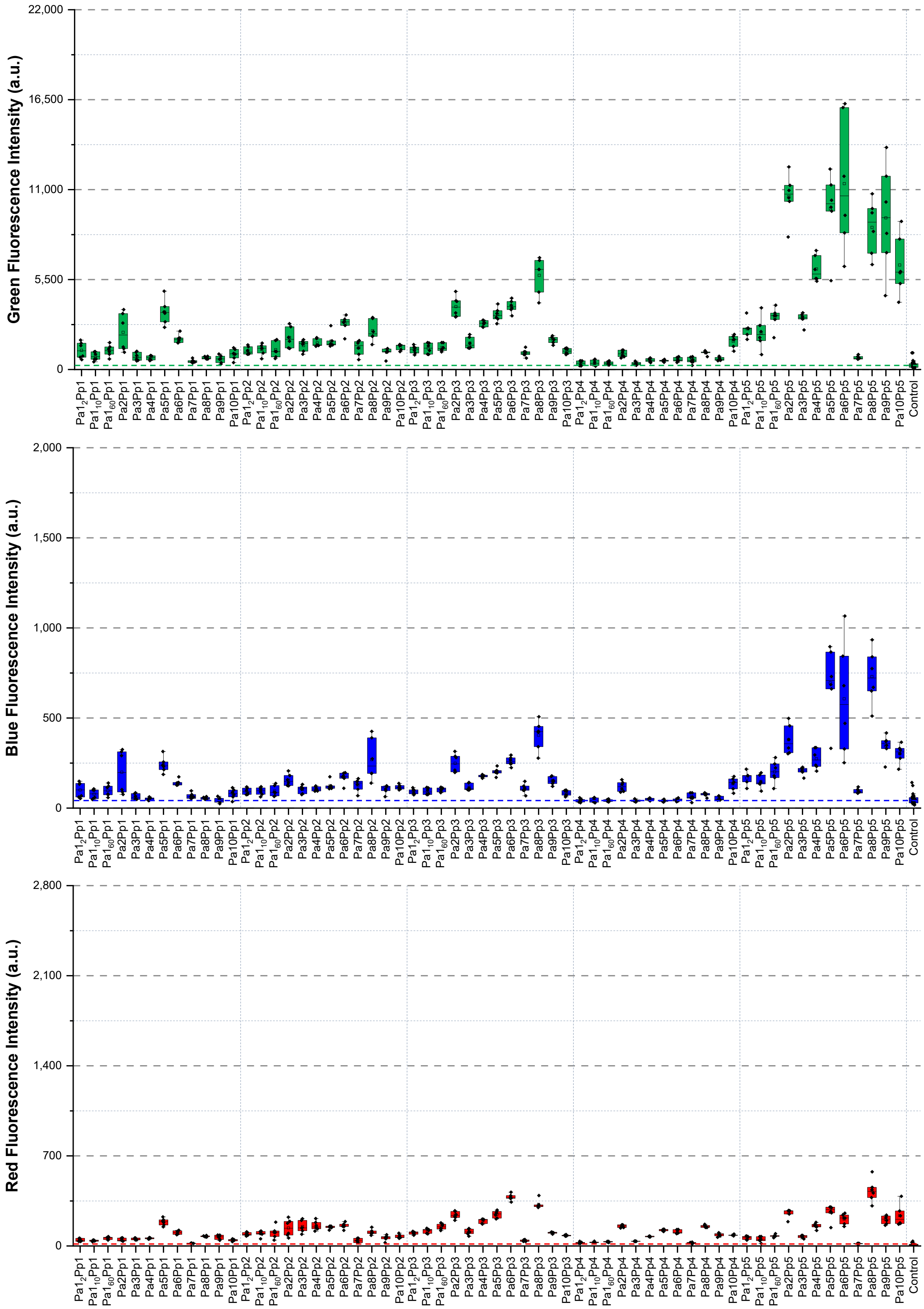

Figure S22. UHT screening of fluorescence properties: fluorescence intensities of **PaPp** coatings made of **Pa1$_2$-Pa10** crosslinked with **Pp1-Pp5**, respectively. Uncoated spots served as controls.

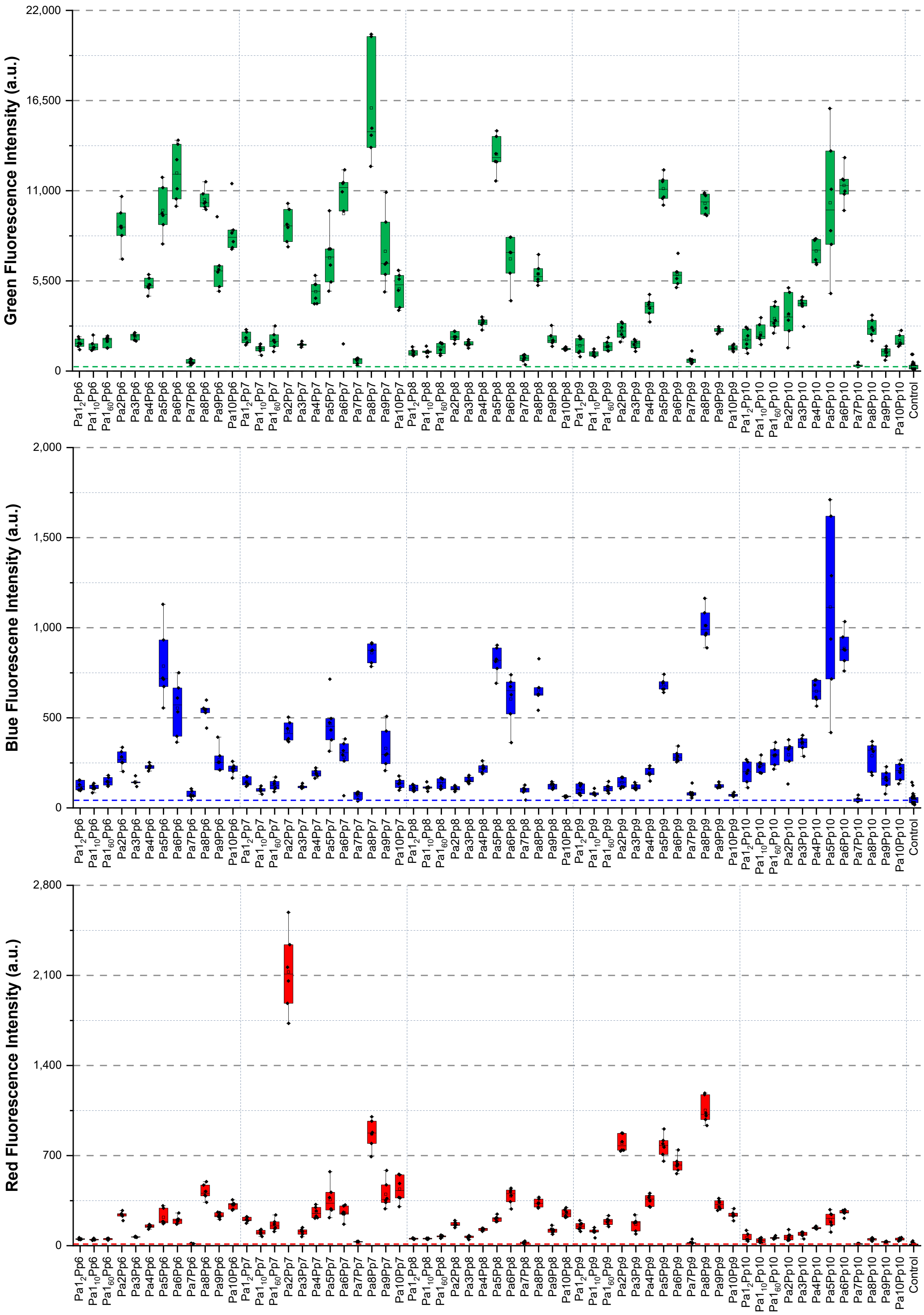

Figure S23. UHT screening of fluorescence properties: fluorescence intensities of **PaPp** coatings made of **Pa1$_2$-Pa10** crosslinked with **Pp6-Pp10**, respectively. Uncoated spots served as controls.

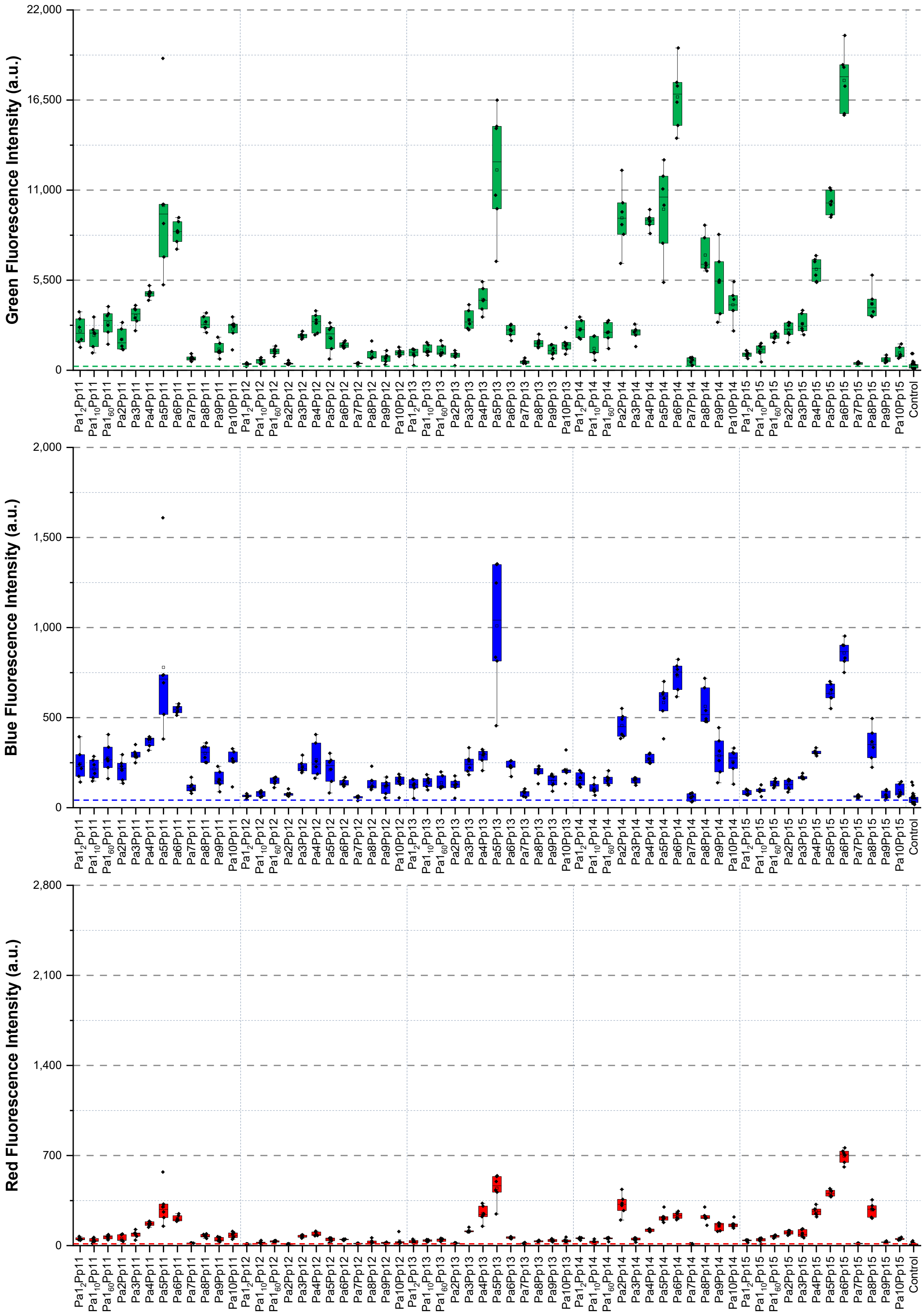

Figure S24. UHT screening of fluorescence properties: fluorescence intensities of **PaPp** coatings made of **Pa1$_2$-Pa10** crosslinked with **Pp11-Pp15**, respectively. Uncoated spots served as controls.

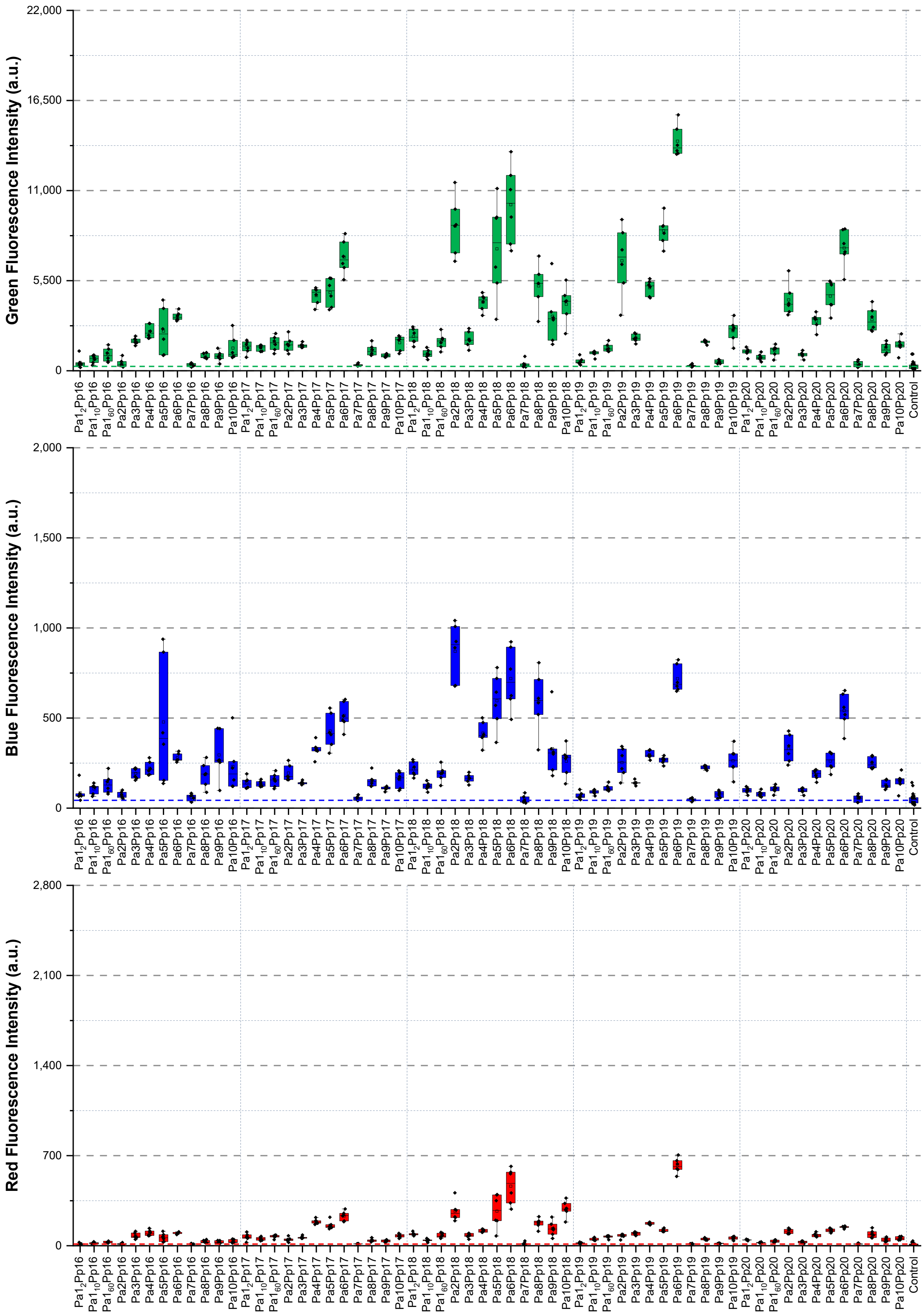

Figure S25. UHT screening of fluorescence properties: fluorescence intensities of **PaPp** coatings made of **Pa1$_2$-Pa10** crosslinked with **Pp16-Pp20**, respectively. Uncoated spots served as controls.

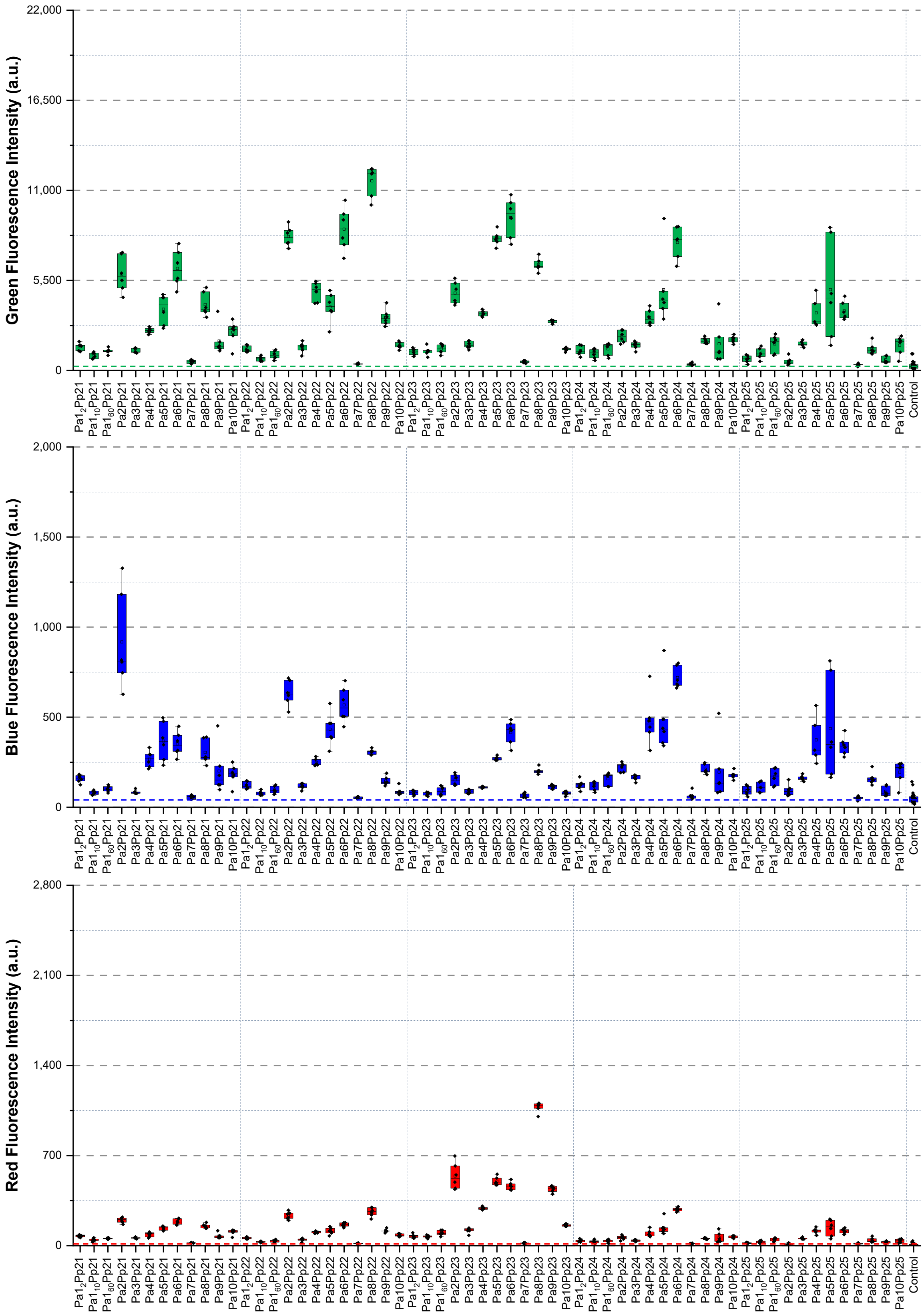

Figure S26. UHT screening of fluorescence properties: fluorescence intensities of **PaPp** coatings made of **Pa1$_2$-Pa10** crosslinked with **Pp21-Pp25**, respectively. Uncoated spots served as controls.

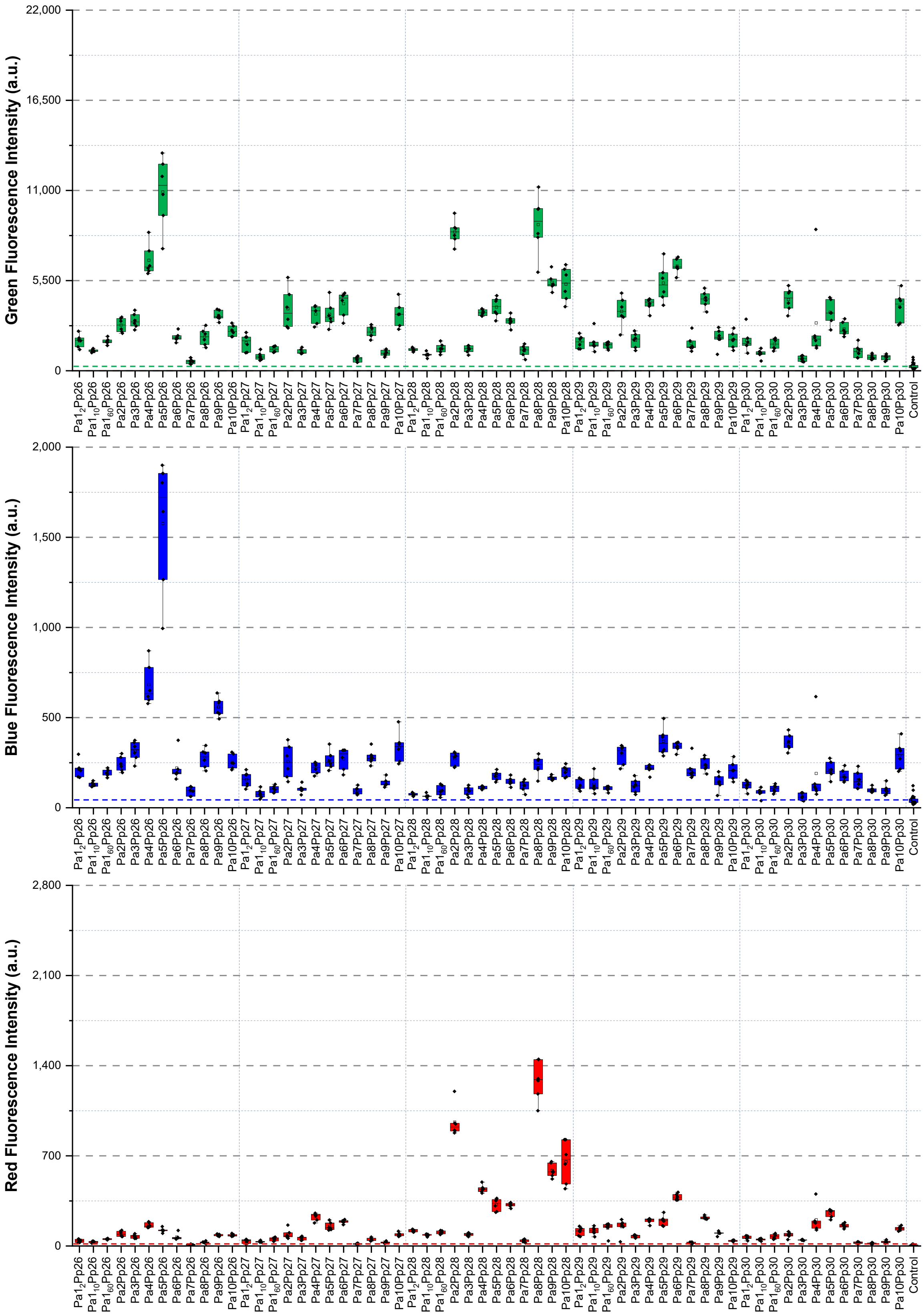

Figure S27. UHT screening of fluorescence properties: fluorescence intensities of **PaPp** coatings made of **Pa1$_2$-Pa10** crosslinked with **Pp26-Pp30**, respectively. Uncoated spots served as controls.

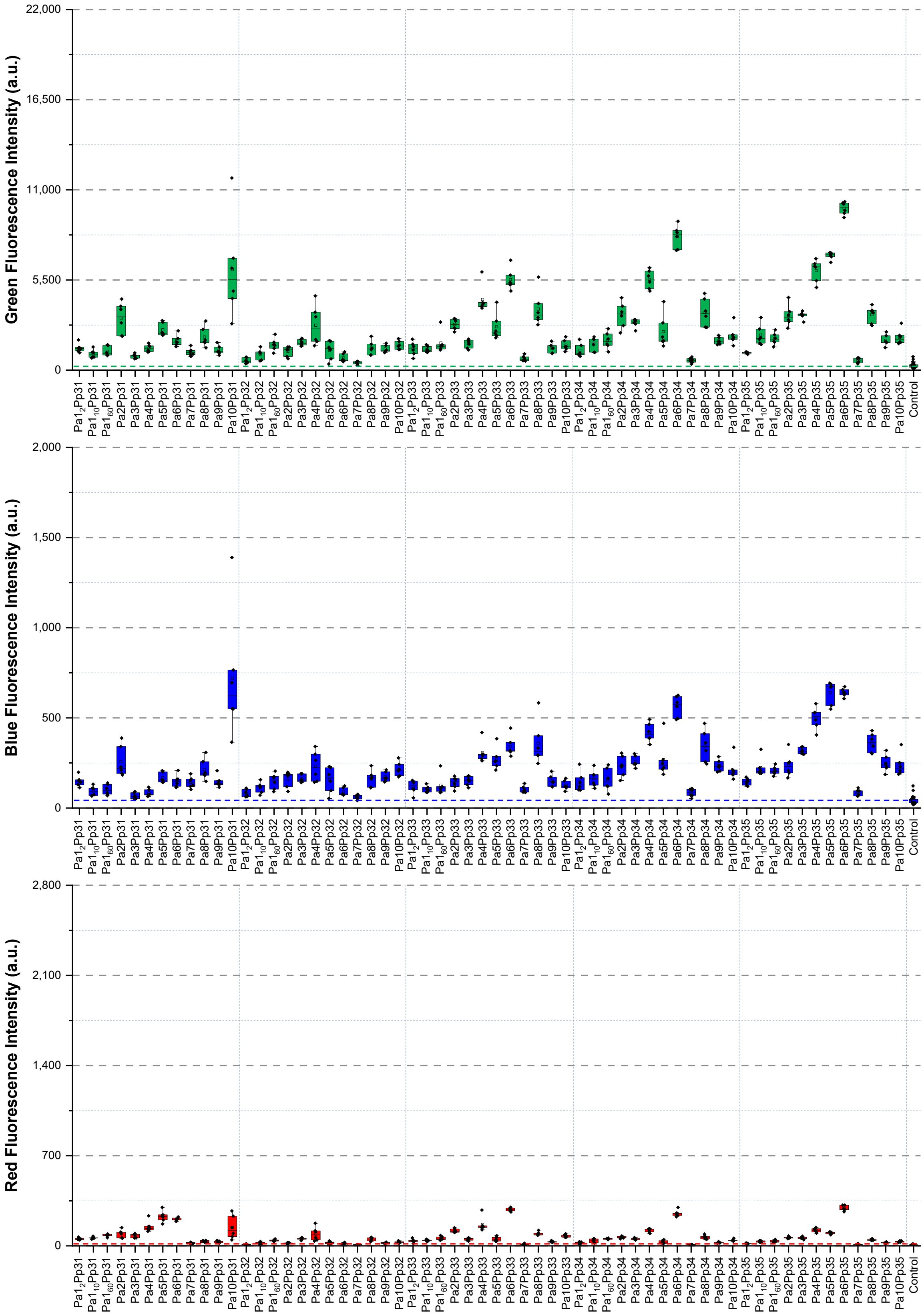

Figure S28. UHT screening of fluorescence properties: fluorescence intensities of **PaPp** coatings made of **$Pa1_2$-Pa10** crosslinked with **Pp31-Pp35**, respectively. Uncoated spots served as controls.

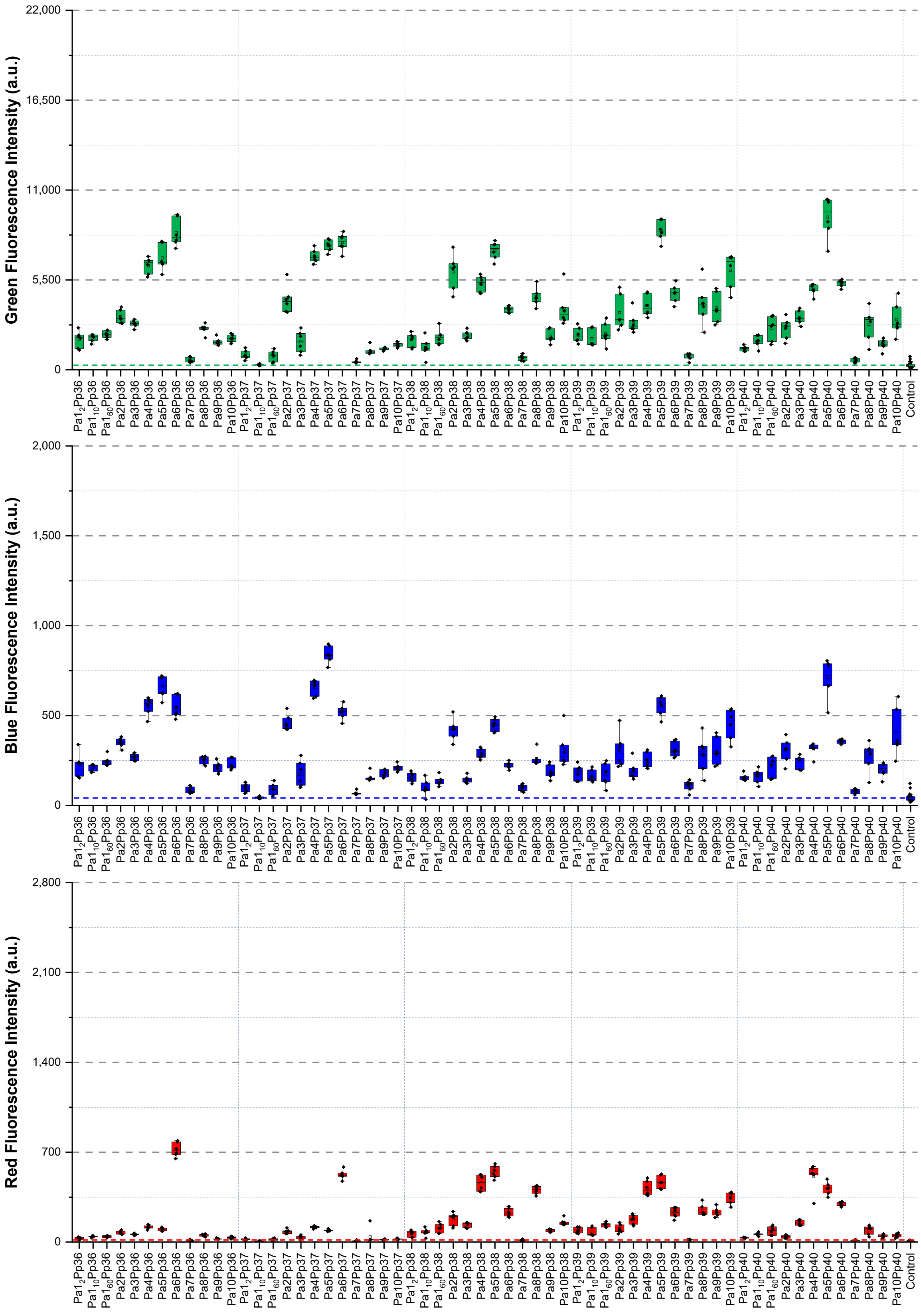

Figure S29. UHT screening of fluorescence properties: fluorescence intensities of **PaPp** coatings made of **Pa1$_2$-Pa10** crosslinked with **Pp36-Pp40**, respectively. Uncoated spots served as controls.

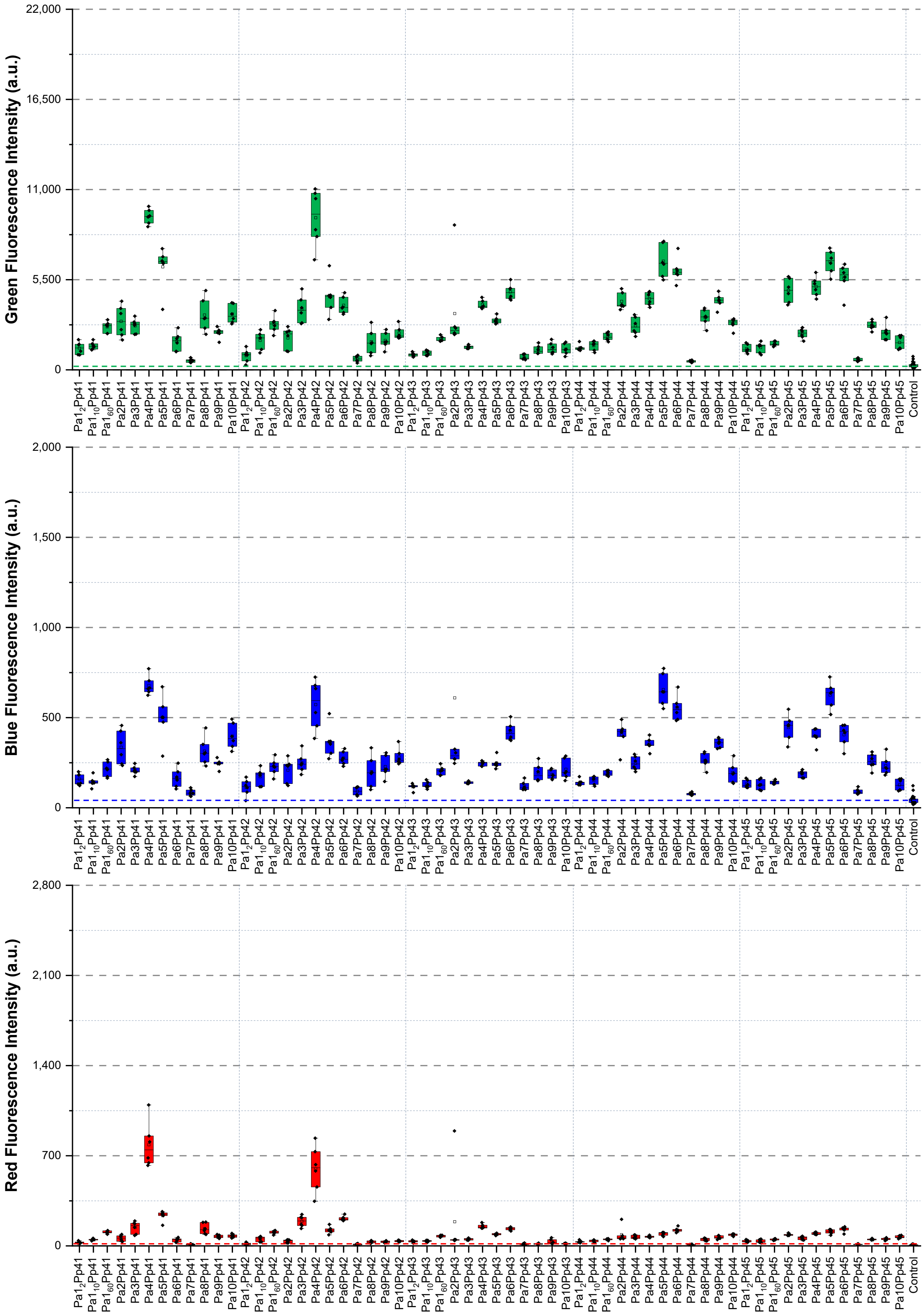

Figure S30. UHT screening of fluorescence properties: fluorescence intensities of **PaPp** coatings made of **Pa1$_2$-Pa10** crosslinked with **Pp41-Pp45**, respectively. Uncoated spots served as controls.

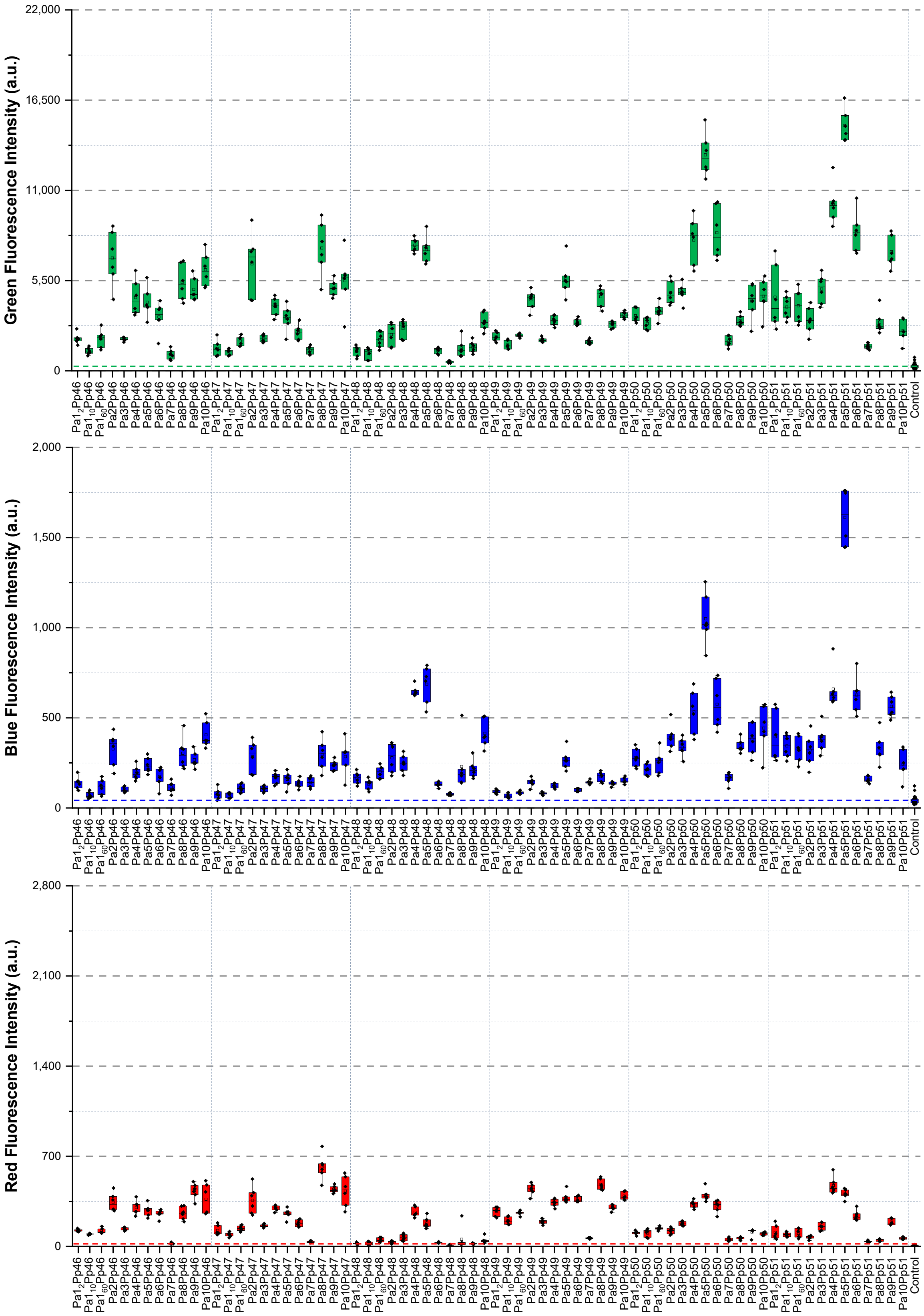

Figure S31. UHT screening of fluorescence properties: fluorescence intensities of **PaPp** coatings made of **Pa1$_2$-Pa10** crosslinked with **Pp46-Pp51**, respectively. Uncoated spots served as controls.

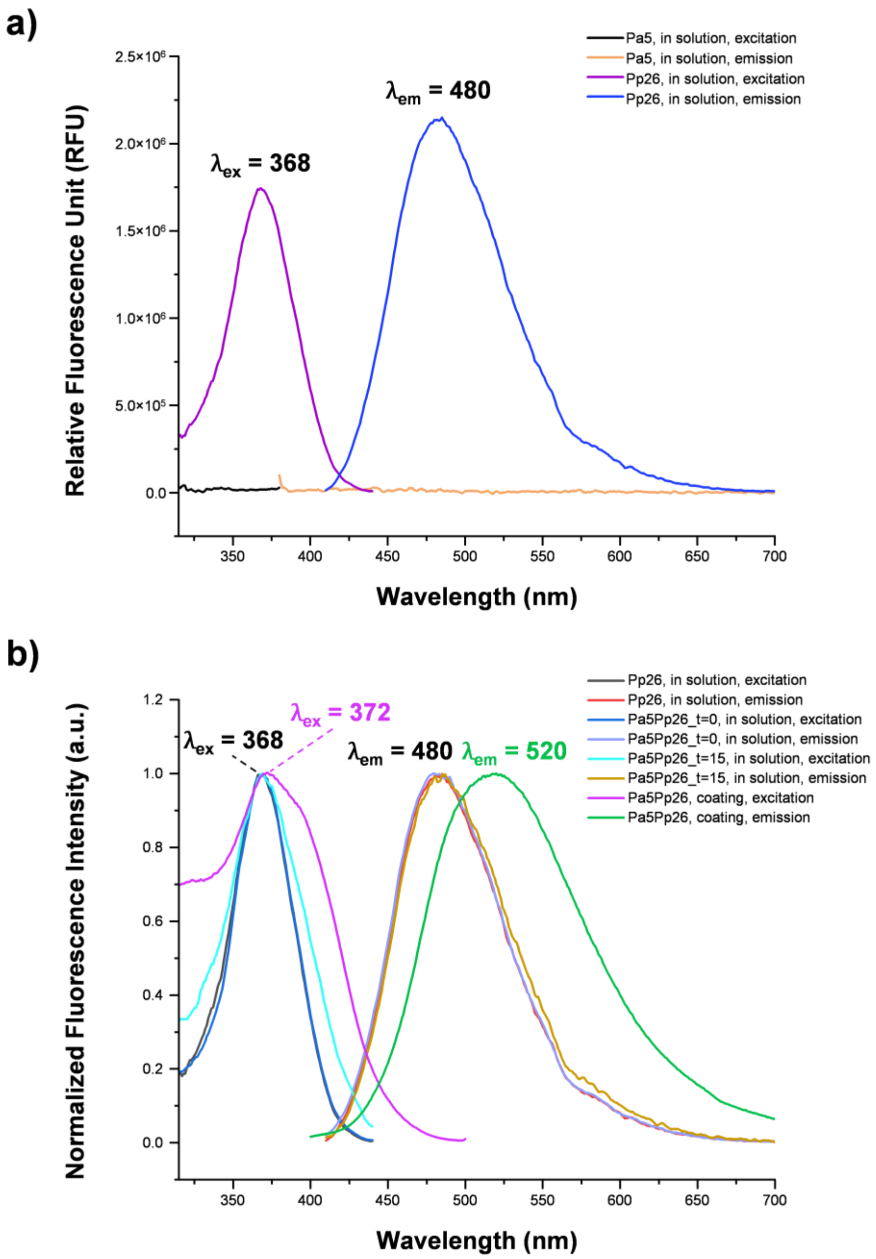

Figure S32. Excitation and emission spectra. a) Spectra of **Pa5** (39 µM, lysine residue molarity) and **Pp26** (36.2 µM) in a 1:1 v/v DMSO/Tris buffer mixture (10 mM, pH 8.5). **Pa5** exhibited no detectable fluorescence, whereas **Pp26** showed strong blue emission with a maximum at λ_{em} = 480 nm. b) Spectra of a mixture of **Pa5** (3.9 mM, lysine residue molarity) and **Pp26** (3.62 mM) after different mixing times (t = 0 h and t = 15 h). The samples were diluted to 39 µM (**Pa5**, lysine residue molarity) and 36.2 µM (**Pp26**) prior to fluorescence measurements. No significant spectral shift was observed after dilution. In contrast, **Pa5Pp26** coating exhibited a significant bathochromic emission shift (λ_{em} = 520 nm, green emission), accompanied by broadening of both the excitation and emission spectra.

5. UHT Screening of Metal-Reducing Activity

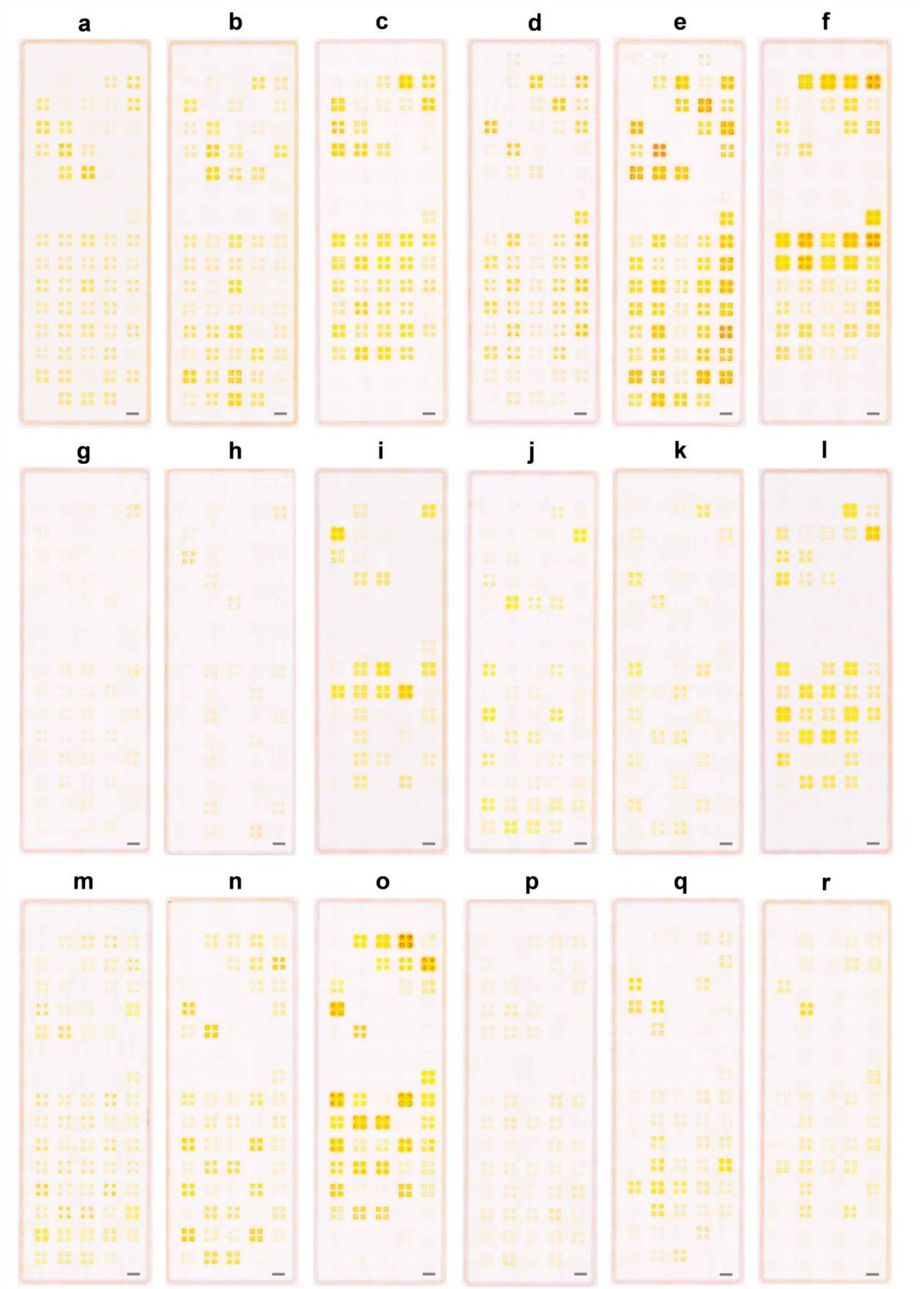

Figure S33. Images of **PaPp** coatings after immersion in $AgNO_3$ solution (10 mM, 48 h, RT). Color development indicates the in-situ formation of AgNPs via Ag^+ reduction. The corresponding coating assignment is shown in **Figure S35** and **Tables S5–S14**. The library shown comprises coatings based on **Pp1-Pp30**. Scale bar: 2.5 mm.

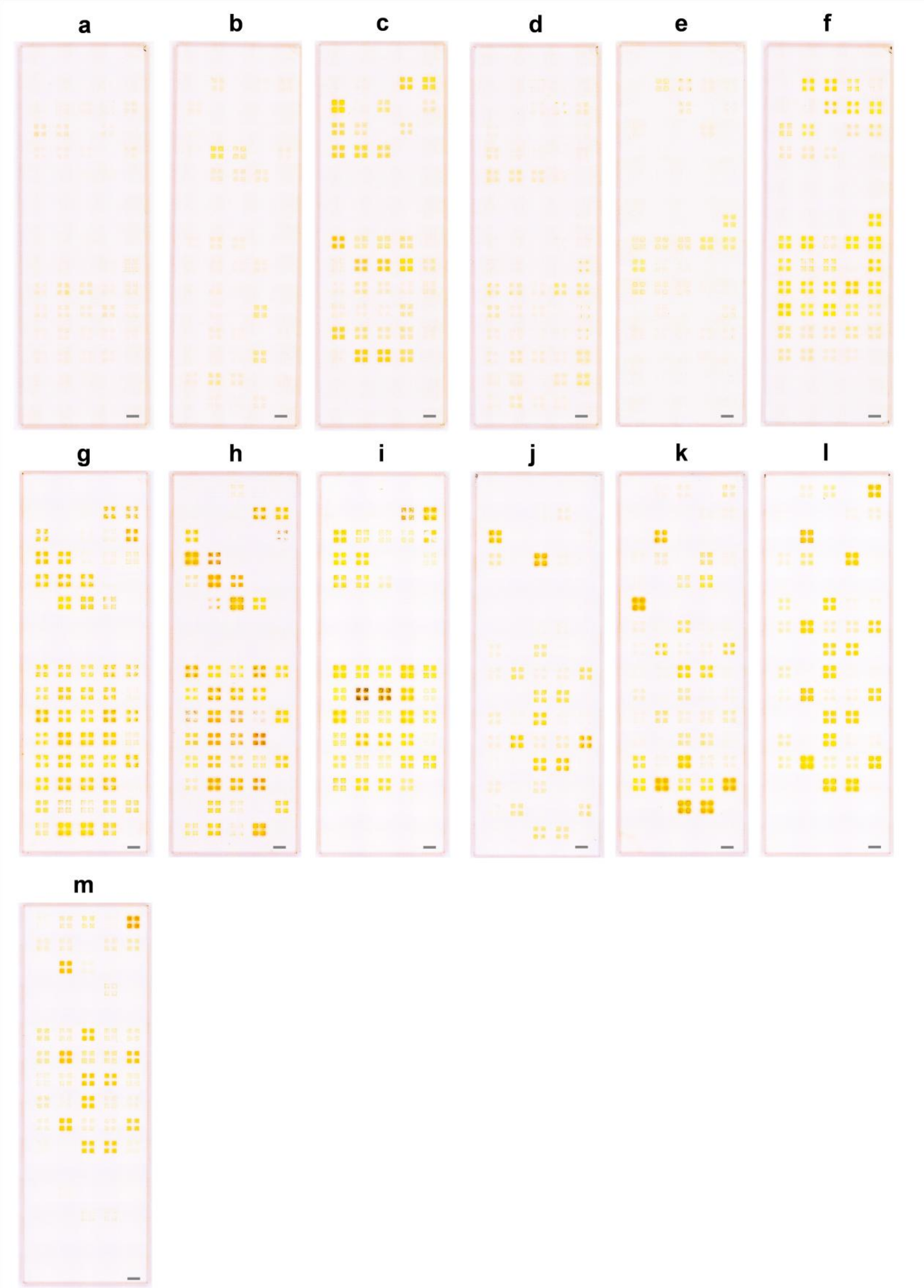

Figure S34. Images of **PaPp** coatings after immersion in $AgNO_3$ solution (10 mM, 48 h, RT). Color development indicates the in-situ formation of AgNPs via Ag^+ reduction. The corresponding coating assignment is shown in **Figure S35** and **Tables S5–S14**. The library shown comprises coatings based on **Pp31-Pp51**. Scale bar: 2.5 mm.

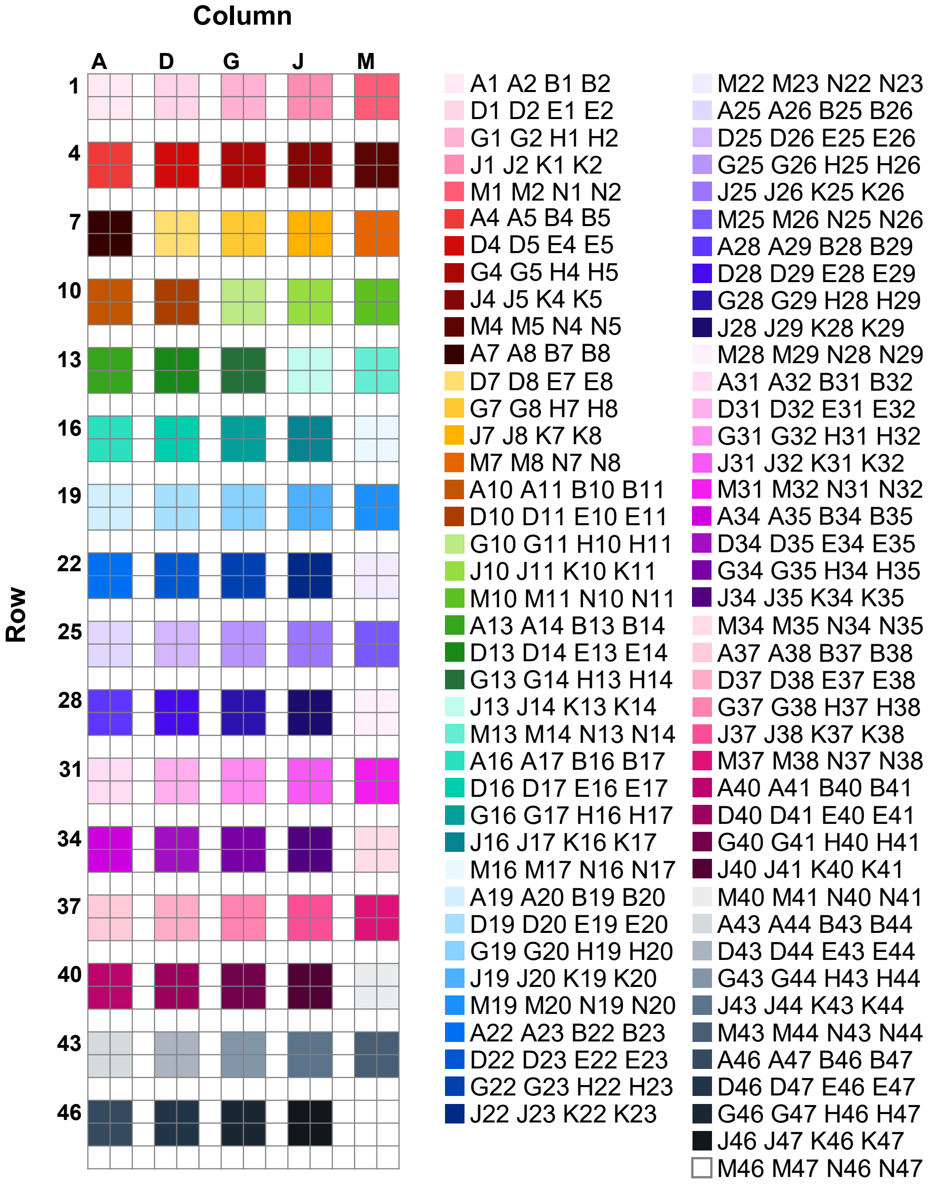

Figure S35. Assignment of **PaPp** coatings screened for metal-reducing ability. The color code corresponds to the coating library listed in **Tables S5–S14**.

Table S5. Color code corresponding to the coating layout in **Figure S35** and the **PaPp** coatings shown in **Figure S33a–c**.

Figure S33a		Figure S33b		Figure S33c	
Pp1	Pa1$_{10}$Pp1Pp2	Pp1	Pa1$_{2}$Pp1Pp2	Pp1	Pa2Pp1Pp2
Pp2	Pa1$_{10}$Pp1Pp3	Pp2	Pa1$_{2}$Pp1Pp3	Pp2	Pa2Pp1Pp3
Pp3	Pa1$_{10}$Pp1Pp4	Pp3	Pa1$_{2}$Pp1Pp4	Pp3	Pa2Pp1Pp4
Pp4	Pa1$_{10}$Pp1Pp5	Pp4	Pa1$_{2}$Pp1Pp5	Pp4	Pa2Pp1Pp5
Pp5	Pa1$_{10}$Pp2Pp3	Pp5	Pa1$_{2}$Pp2Pp3	Pp5	Pa2Pp2Pp3
Pa1$_{10}$	Pa1$_{10}$Pp2Pp4	Pa1$_{2}$	Pa1$_{2}$Pp2Pp4	Pa2	Pa2Pp2Pp4
Pa1$_{10}$Pp1	Pa1$_{10}$Pp2Pp5	Pa1$_{2}$Pp1	Pa1$_{2}$Pp2Pp5	Pa2Pp1	Pa2Pp2Pp5
Pa1$_{10}$Pp2	Pa1$_{10}$Pp3Pp4	Pa1$_{2}$Pp2	Pa1$_{2}$Pp3Pp4	Pa2Pp2	Pa2Pp3Pp4
Pa1$_{10}$Pp3	Pa1$_{10}$Pp3Pp5	Pa1$_{2}$Pp3	Pa1$_{2}$Pp3Pp5	Pa2Pp3	Pa2Pp3Pp5
Pa1$_{10}$Pp4	Pa1$_{10}$Pp4Pp5	Pa1$_{2}$Pp4	Pa1$_{2}$Pp4Pp5	Pa2Pp4	Pa2Pp4Pp5
Pa1$_{10}$Pp5	Pa1$_{60}$Pp1Pp2	Pa1$_{2}$Pp5	Pa8Pp1Pp2	Pa2Pp5	Pa4Pp1Pp2
Pa1$_{60}$	Pa1$_{60}$Pp1Pp3	Pa8	Pa8Pp1Pp3	Pa4	Pa4Pp1Pp3
Pa1$_{60}$Pp1	Pa1$_{60}$Pp1Pp4	Pa8Pp1	Pa8Pp1Pp4	Pa4Pp1	Pa4Pp1Pp4
Pa1$_{60}$Pp2	Pa1$_{60}$Pp1Pp5	Pa8Pp2	Pa8Pp1Pp5	Pa4Pp2	Pa4Pp1Pp5
Pa1$_{60}$Pp3	Pa1$_{60}$Pp2Pp3	Pa8Pp3	Pa8Pp2Pp3	Pa4Pp3	Pa4Pp2Pp3
Pa1$_{60}$Pp4	Pa1$_{60}$Pp2Pp4	Pa8Pp4	Pa8Pp2Pp4	Pa4Pp4	Pa4Pp2Pp4
Pa1$_{60}$Pp5	Pa1$_{60}$Pp2Pp5	Pa8Pp5	Pa8Pp2Pp5	Pa4Pp5	Pa4Pp2Pp5
Pa3	Pa1$_{60}$Pp3Pp4	Pa9	Pa8Pp3Pp4	Pa6	Pa4Pp3Pp4
Pa3Pp1	Pa1$_{60}$Pp3Pp5	Pa9Pp1	Pa8Pp3Pp5	Pa6Pp1	Pa4Pp3Pp5
Pa3Pp2	Pa1$_{60}$Pp4Pp5	Pa9Pp2	Pa8Pp4Pp5	Pa6Pp2	Pa4Pp4Pp5
Pa3Pp3	Pa3Pp1Pp2	Pa9Pp3	Pa9Pp1Pp2	Pa6Pp3	Pa6Pp1Pp2
Pa3Pp4	Pa3Pp1Pp3	Pa9Pp4	Pa9Pp1Pp3	Pa6Pp4	Pa6Pp1Pp3
Pa3Pp5	Pa3Pp1Pp4	Pa9Pp5	Pa9Pp1Pp4	Pa6Pp5	Pa6Pp1Pp4
Pa5	Pa3Pp1Pp5	Pa10	Pa9Pp1Pp5	Pa7	Pa6Pp1Pp5
Pa5Pp1	Pa3Pp2Pp3	Pa10Pp1	Pa9Pp2Pp3	Pa7Pp1	Pa6Pp2Pp3
Pa5Pp2	Pa3Pp2Pp4	Pa10Pp2	Pa9Pp2Pp4	Pa7Pp2	Pa6Pp2Pp4
Pa5Pp3	Pa3Pp2Pp5	Pa10Pp3	Pa9Pp2Pp5	Pa7Pp3	Pa6Pp2Pp5
Pa5Pp4	Pa3Pp3Pp4	Pa10Pp4	Pa9Pp3Pp4	Pa7Pp4	Pa6Pp3Pp4
Pa5Pp5	Pa3Pp3Pp5	Pa10Pp5	Pa9Pp3Pp5	Pa7Pp5	Pa6Pp3Pp5
Pp1Pp2	Pa3Pp4Pp5	Pp1Pp2	Pa9Pp4Pp5	Pp1Pp2	Pa6Pp4Pp5
Pp1Pp3	Pa5Pp1Pp2	Pp1Pp3	Pa10Pp1Pp2	Pp1Pp3	Pa7Pp1Pp2
Pp1Pp4	Pa5Pp1Pp3	Pp1Pp4	Pa10Pp1Pp3	Pp1Pp4	Pa7Pp1Pp3
Pp1Pp5	Pa5Pp1Pp4	Pp1Pp5	Pa10Pp1Pp4	Pp1Pp5	Pa7Pp1Pp4
Pp2Pp3	Pa5Pp1Pp5	Pp2Pp3	Pa10Pp1Pp5	Pp2Pp3	Pa7Pp1Pp5
Pp2Pp4	Pa5Pp2Pp3	Pp2Pp4	Pa10Pp2Pp3	Pp2Pp4	Pa7Pp2Pp3
Pp2Pp5	Pa5Pp2Pp4	Pp2Pp5	Pa10Pp2Pp4	Pp2Pp5	Pa7Pp2Pp4
Pp3Pp4	Pa5Pp2Pp5	Pp3Pp4	Pa10Pp2Pp5	Pp3Pp4	Pa7Pp2Pp5
Pp3Pp5	Pa5Pp3Pp4	Pp3Pp5	Pa10Pp3Pp4	Pp3Pp5	Pa7Pp3Pp4
Pp4Pp5	Pa5Pp3Pp5	Pp4Pp5	Pa10Pp3Pp5	Pp4Pp5	Pa7Pp3Pp5
	Pa5Pp4Pp5		Pa10Pp4Pp5		Pa7Pp4Pp5

Table S6. Color code corresponding to the coating layout in **Figure S35** and the **PaPp** coatings shown in **Figure S33d–f**.

Figure S33d		Figure S33e		Figure S33f	
Pp6	Pa1$_{10}$Pp6Pp7	Pp6	Pa1$_{2}$Pp6Pp7	Pp6	Pa2Pp6Pp7
Pp7	Pa1$_{10}$Pp6Pp8	Pp7	Pa1$_{2}$Pp6Pp8	Pp7	Pa2Pp6Pp8
Pp8	Pa1$_{10}$Pp6Pp9	Pp8	Pa1$_{2}$Pp6Pp9	Pp8	Pa2Pp6Pp9
Pp9	Pa1$_{10}$Pp6Pp10	Pp9	Pa1$_{2}$Pp6Pp10	Pp9	Pa2Pp6Pp10
Pp10	Pa1$_{10}$Pp7Pp8	Pp10	Pa1$_{2}$Pp7Pp8	Pp10	Pa2Pp7Pp8
Pa1$_{10}$	Pa1$_{10}$Pp7Pp9	Pa1$_{2}$	Pa1$_{2}$Pp7Pp9	Pa2	Pa2Pp7Pp9
Pa1$_{10}$Pp6	Pa1$_{10}$Pp7Pp10	Pa1$_{2}$Pp6	Pa1$_{2}$Pp7Pp10	Pa2Pp6	Pa2Pp7Pp10
Pa1$_{10}$Pp7	Pa1$_{10}$Pp8Pp9	Pa1$_{2}$Pp7	Pa1$_{2}$Pp8Pp9	Pa2Pp7	Pa2Pp8Pp9
Pa1$_{10}$Pp8	Pa1$_{10}$Pp8Pp10	Pa1$_{2}$Pp8	Pa1$_{2}$Pp8Pp10	Pa2Pp8	Pa2Pp8Pp10
Pa1$_{10}$Pp9	Pa1$_{10}$Pp9Pp10	Pa1$_{2}$Pp9	Pa1$_{2}$Pp9Pp10	Pa2Pp9	Pa2Pp9Pp10
Pa1$_{10}$Pp10	Pa1$_{60}$Pp6Pp7	Pa1$_{2}$Pp10	Pa8Pp6Pp7	Pa2Pp10	Pa4Pp6Pp7
Pa1$_{60}$	Pa1$_{60}$Pp6Pp8	Pa8	Pa8Pp6Pp8	Pa4	Pa4Pp6Pp8
Pa1$_{60}$Pp6	Pa1$_{60}$Pp6Pp9	Pa8Pp6	Pa8Pp6Pp9	Pa4Pp6	Pa4Pp6Pp9
Pa1$_{60}$Pp7	Pa1$_{60}$Pp6Pp10	Pa8Pp7	Pa8Pp6Pp10	Pa4Pp7	Pa4Pp6Pp10
Pa1$_{60}$Pp8	Pa1$_{60}$Pp7Pp8	Pa8Pp8	Pa8Pp7Pp8	Pa4Pp8	Pa4Pp7Pp8
Pa1$_{60}$Pp9	Pa1$_{60}$Pp7Pp9	Pa8Pp9	Pa8Pp7Pp9	Pa4Pp9	Pa4Pp7Pp9
Pa1$_{60}$Pp10	Pa1$_{60}$Pp7Pp10	Pa8Pp10	Pa8Pp7Pp10	Pa4Pp10	Pa4Pp7Pp10
Pa3	Pa1$_{60}$Pp8Pp9	Pa9	Pa8Pp8Pp9	Pa6	Pa4Pp8Pp9
Pa3Pp6	Pa1$_{60}$Pp8Pp10	Pa9Pp6	Pa8Pp8Pp10	Pa6Pp6	Pa4Pp8Pp10
Pa3Pp7	Pa1$_{60}$Pp9Pp10	Pa9Pp7	Pa8Pp9Pp10	Pa6Pp7	Pa4Pp9Pp10
Pa3Pp8	Pa3Pp6Pp7	Pa9Pp8	Pa9Pp6Pp7	Pa6Pp8	Pa6Pp6Pp7
Pa3Pp9	Pa3Pp6Pp8	Pa9Pp9	Pa9Pp6Pp8	Pa6Pp9	Pa6Pp6Pp8
Pa3Pp10	Pa3Pp6Pp9	Pa9Pp10	Pa9Pp6Pp9	Pa6Pp10	Pa6Pp6Pp9
Pa5	Pa3Pp6Pp10	Pa10	Pa9Pp6Pp10	Pa7	Pa6Pp6Pp10
Pa5Pp6	Pa3Pp7Pp8	Pa10Pp6	Pa9Pp7Pp8	Pa7Pp6	Pa6Pp7Pp8
Pa5Pp7	Pa3Pp7Pp9	Pa10Pp7	Pa9Pp7Pp9	Pa7Pp7	Pa6Pp7Pp9
Pa5Pp8	Pa3Pp7Pp10	Pa10Pp8	Pa9Pp7Pp10	Pa7Pp8	Pa6Pp7Pp10
Pa5Pp9	Pa3Pp8Pp9	Pa10Pp9	Pa9Pp8Pp9	Pa7Pp9	Pa6Pp8Pp9
Pa5Pp10	Pa3Pp8Pp10	Pa10Pp10	Pa9Pp8Pp10	Pa7Pp10	Pa6Pp8Pp10
Pp6Pp7	Pa3Pp9Pp10	Pp6Pp7	Pa9Pp9Pp10	Pp6Pp7	Pa6Pp9Pp10
Pp6Pp8	Pa5Pp6Pp7	Pp6Pp8	Pa10Pp6Pp7	Pp6Pp8	Pa7Pp6Pp7
Pp6Pp9	Pa5Pp6Pp8	Pp6Pp9	Pa10Pp6Pp8	Pp6Pp9	Pa7Pp6Pp8
Pp6Pp10	Pa5Pp6Pp9	Pp6Pp10	Pa10Pp6Pp9	Pp6Pp10	Pa7Pp6Pp9
Pp7Pp8	Pa5Pp6Pp10	Pp7Pp8	Pa10Pp6Pp10	Pp7Pp8	Pa7Pp6Pp10
Pp7Pp9	Pa5Pp7Pp8	Pp7Pp9	Pa10Pp7Pp8	Pp7Pp9	Pa7Pp7Pp8
Pp7Pp10	Pa5Pp7Pp9	Pp7Pp10	Pa10Pp7Pp9	Pp7Pp10	Pa7Pp7Pp9
Pp8Pp9	Pa5Pp7Pp10	Pp8Pp9	Pa10Pp7Pp10	Pp8Pp9	Pa7Pp7Pp10
Pp8Pp10	Pa5Pp8Pp9	Pp8Pp10	Pa10Pp8Pp9	Pp8Pp10	Pa7Pp8Pp9
Pp9Pp10	Pa5Pp8Pp10	Pp9Pp10	Pa10Pp8Pp10	Pp9Pp10	Pa7Pp8Pp10
	Pa5Pp9Pp10		Pa10Pp9Pp10		Pa7Pp9Pp10

Table S7. Color code corresponding to the coating layout in **Figure S35** and the **PaPp** coatings shown in **Figure S33g–i**.

Figure S33g		Figure S33h		Figure S33i	
Pp11	Pa1$_{10}$Pp11Pp12	Pp11	Pa1$_{2}$Pp11Pp12	Pp11	Pa2Pp11Pp12
Pp12	Pa1$_{10}$Pp11Pp13	Pp12	Pa1$_{2}$Pp11Pp13	Pp12	Pa2Pp11Pp13
Pp13	Pa1$_{10}$Pp11Pp14	Pp13	Pa1$_{2}$Pp11Pp14	Pp13	Pa2Pp11Pp14
Pp14	Pa1$_{10}$Pp11Pp15	Pp14	Pa1$_{2}$Pp11Pp15	Pp14	Pa2Pp11Pp15
Pp15	Pa1$_{10}$Pp12Pp13	Pp15	Pa1$_{2}$Pp12Pp13	Pp15	Pa2Pp12Pp13
Pa1$_{10}$	Pa1$_{10}$Pp12Pp14	Pa1$_{2}$	Pa1$_{2}$Pp12Pp14	Pa2	Pa2Pp12Pp14
Pa1$_{10}$Pp11	Pa1$_{10}$Pp12Pp15	Pa1$_{2}$Pp11	Pa1$_{2}$Pp12Pp15	Pa2Pp11	Pa2Pp12Pp15
Pa1$_{10}$Pp12	Pa1$_{10}$Pp13Pp14	Pa1$_{2}$Pp12	Pa1$_{2}$Pp13Pp14	Pa2Pp12	Pa2Pp13Pp14
Pa1$_{10}$Pp13	Pa1$_{10}$Pp13Pp15	Pa1$_{2}$Pp13	Pa1$_{2}$Pp13Pp15	Pa2Pp13	Pa2Pp13Pp15
Pa1$_{10}$Pp14	Pa1$_{10}$Pp14Pp15	Pa1$_{2}$Pp14	Pa1$_{2}$Pp14Pp15	Pa2Pp14	Pa2Pp14Pp15
Pa1$_{10}$Pp15	Pa1$_{60}$Pp11Pp12	Pa1$_{2}$Pp15	Pa8Pp11Pp12	Pa2Pp15	Pa4Pp11Pp12
Pa1$_{60}$	Pa1$_{60}$Pp11Pp13	Pa8	Pa8Pp11Pp13	Pa4	Pa4Pp11Pp13
Pa1$_{60}$Pp11	Pa1$_{60}$Pp11Pp14	Pa8Pp11	Pa8Pp11Pp14	Pa4Pp11	Pa4Pp11Pp14
Pa1$_{60}$Pp12	Pa1$_{60}$Pp11Pp15	Pa8Pp12	Pa8Pp11Pp15	Pa4Pp12	Pa4Pp11Pp15
Pa1$_{60}$Pp13	Pa1$_{60}$Pp12Pp13	Pa8Pp13	Pa8Pp12Pp13	Pa4Pp13	Pa4Pp12Pp13
Pa1$_{60}$Pp14	Pa1$_{60}$Pp12Pp14	Pa8Pp14	Pa8Pp12Pp14	Pa4Pp14	Pa4Pp12Pp14
Pa1$_{60}$Pp15	Pa1$_{60}$Pp12Pp15	Pa8Pp15	Pa8Pp12Pp15	Pa4Pp15	Pa4Pp12Pp15
Pa3	Pa1$_{60}$Pp13Pp14	Pa9	Pa8Pp13Pp14	Pa6	Pa4Pp13Pp14
Pa3Pp11	Pa1$_{60}$Pp13Pp15	Pa9Pp11	Pa8Pp13Pp15	Pa6Pp11	Pa4Pp13Pp15
Pa3Pp12	Pa1$_{60}$Pp14Pp15	Pa9Pp12	Pa8Pp14Pp15	Pa6Pp12	Pa4Pp14Pp15
Pa3Pp13	Pa3Pp11Pp12	Pa9Pp13	Pa9Pp11Pp12	Pa6Pp13	Pa6Pp11Pp12
Pa3Pp14	Pa3Pp11Pp13	Pa9Pp14	Pa9Pp11Pp13	Pa6Pp14	Pa6Pp11Pp13
Pa3Pp15	Pa3Pp11Pp14	Pa9Pp15	Pa9Pp11Pp14	Pa6Pp15	Pa6Pp11Pp14
Pa5	Pa3Pp11Pp15	Pa10	Pa9Pp11Pp15	Pa7	Pa6Pp11Pp15
Pa5Pp11	Pa3Pp12Pp13	Pa10Pp11	Pa9Pp12Pp13	Pa7Pp11	Pa6Pp12Pp13
Pa5Pp12	Pa3Pp12Pp14	Pa10Pp12	Pa9Pp12Pp14	Pa7Pp12	Pa6Pp12Pp14
Pa5Pp13	Pa3Pp12Pp14	Pa10Pp13	Pa9Pp12Pp14	Pa7Pp13	Pa6Pp12Pp14
Pa5Pp14	Pa3Pp13Pp14	Pa10Pp14	Pa9Pp13Pp14	Pa7Pp14	Pa6Pp13Pp14
Pa5Pp15	Pa3Pp13Pp15	Pa10Pp15	Pa9Pp13Pp15	Pa7Pp15	Pa6Pp13Pp15
Pp11Pp12	Pa3Pp14Pp15	Pp11Pp12	Pa9Pp14Pp15	Pp11Pp12	Pa6Pp14Pp15
Pp11Pp13	Pa5Pp11Pp12	Pp11Pp13	Pa10Pp11Pp12	Pp11Pp13	Pa7Pp11Pp12
Pp11Pp14	Pa5Pp11Pp13	Pp11Pp14	Pa10Pp11Pp13	Pp11Pp14	Pa7Pp11Pp13
Pp11Pp15	Pa5Pp11Pp14	Pp11Pp15	Pa10Pp11Pp14	Pp11Pp15	Pa7Pp11Pp14
Pp12Pp13	Pa5Pp11Pp15	Pp12Pp13	Pa10Pp11Pp15	Pp12Pp13	Pa7Pp11Pp15
Pp12Pp14	Pa5Pp12Pp13	Pp12Pp14	Pa10Pp12Pp13	Pp12Pp14	Pa7Pp12Pp13
Pp12Pp15	Pa5Pp12Pp14	Pp12Pp15	Pa10Pp12Pp14	Pp12Pp15	Pa7Pp12Pp14
Pp13Pp14	Pa5Pp12Pp15	Pp13Pp14	Pa10Pp12Pp15	Pp13Pp14	Pa7Pp12Pp15
Pp13Pp15	Pa5Pp13Pp14	Pp13Pp15	Pa10Pp13Pp14	Pp13Pp15	Pa7Pp13Pp14
Pp14Pp15	Pa5Pp13Pp15	Pp14Pp15	Pa10Pp13Pp15	Pp14Pp15	Pa7Pp13Pp15
	Pa5Pp14Pp15		Pa10Pp14Pp15		Pa7Pp14Pp15

Table S8. Color code corresponding to the coating layout in **Figure S35** and the **PaPp** coatings shown in **Figure S33j–l**.

Figure S33j		Figure S33k		Figure S33l	
Pp16	Pa1$_{10}$Pp16Pp17	Pp16	Pa1$_{2}$Pp16Pp17	Pp16	Pa2Pp16Pp17
Pp17	Pa1$_{10}$Pp16Pp18	Pp17	Pa1$_{2}$Pp16Pp18	Pp17	Pa2Pp16Pp18
Pp18	Pa1$_{10}$Pp16Pp19	Pp18	Pa1$_{2}$Pp16Pp19	Pp18	Pa2Pp16Pp19
Pp19	Pa1$_{10}$Pp16Pp20	Pp19	Pa1$_{2}$Pp16Pp20	Pp19	Pa2Pp16Pp20
Pp20	Pa1$_{10}$Pp17Pp18	Pp20	Pa1$_{2}$Pp17Pp18	Pp20	Pa2Pp17Pp18
Pa1$_{10}$	Pa1$_{10}$Pp17Pp19	Pa1$_{2}$	Pa1$_{2}$Pp17Pp19	Pa2	Pa2Pp17Pp19
Pa1$_{10}$Pp16	Pa1$_{10}$Pp17Pp20	Pa1$_{2}$Pp16	Pa1$_{2}$Pp17Pp20	Pa2Pp16	Pa2Pp17Pp20
Pa1$_{10}$Pp17	Pa1$_{10}$Pp18Pp19	Pa1$_{2}$Pp17	Pa1$_{2}$Pp18Pp19	Pa2Pp17	Pa2Pp18Pp19
Pa1$_{10}$Pp18	Pa1$_{10}$Pp18Pp20	Pa1$_{2}$Pp18	Pa1$_{2}$Pp18Pp20	Pa2Pp18	Pa2Pp18Pp20
Pa1$_{10}$Pp19	Pa1$_{10}$Pp19Pp20	Pa1$_{2}$Pp19	Pa1$_{2}$Pp19Pp20	Pa2Pp19	Pa2Pp19Pp20
Pa1$_{10}$Pp20	Pa1$_{60}$Pp16Pp17	Pa1$_{2}$Pp20	Pa8Pp16Pp17	Pa2Pp20	Pa4Pp16Pp17
Pa1$_{60}$	Pa1$_{60}$Pp16Pp18	Pa8	Pa8Pp16Pp18	Pa4	Pa4Pp16Pp18
Pa1$_{60}$Pp16	Pa1$_{60}$Pp16Pp19	Pa8Pp16	Pa8Pp16Pp19	Pa4Pp16	Pa4Pp16Pp19
Pa1$_{60}$Pp17	Pa1$_{60}$Pp16Pp20	Pa8Pp17	Pa8Pp16Pp20	Pa4Pp17	Pa4Pp16Pp20
Pa1$_{60}$Pp18	Pa1$_{60}$Pp17Pp18	Pa8Pp18	Pa8Pp17Pp18	Pa4Pp18	Pa4Pp17Pp18
Pa1$_{60}$Pp19	Pa1$_{60}$Pp17Pp19	Pa8Pp19	Pa8Pp17Pp19	Pa4Pp19	Pa4Pp17Pp19
Pa1$_{60}$Pp20	Pa1$_{60}$Pp17Pp20	Pa8Pp20	Pa8Pp17Pp20	Pa4Pp20	Pa4Pp17Pp20
Pa3	Pa1$_{60}$Pp18Pp19	Pa9	Pa8Pp18Pp19	Pa6	Pa4Pp18Pp19
Pa3Pp16	Pa1$_{60}$Pp18Pp20	Pa9Pp16	Pa8Pp18Pp20	Pa6Pp16	Pa4Pp18Pp20
Pa3Pp17	Pa1$_{60}$Pp19Pp20	Pa9Pp17	Pa8Pp19Pp20	Pa6Pp17	Pa4Pp19Pp20
Pa3Pp18	Pa3Pp16Pp17	Pa9Pp18	Pa9Pp16Pp17	Pa6Pp18	Pa6Pp16Pp17
Pa3Pp19	Pa3Pp16Pp18	Pa9Pp19	Pa9Pp16Pp18	Pa6Pp19	Pa6Pp16Pp18
Pa3Pp20	Pa3Pp16Pp19	Pa9Pp20	Pa9Pp16Pp19	Pa6Pp20	Pa6Pp16Pp19
Pa5	Pa3Pp16Pp20	Pa10	Pa9Pp16Pp20	Pa7	Pa6Pp16Pp20
Pa5Pp16	Pa3Pp17Pp18	Pa10Pp16	Pa9Pp17Pp18	Pa7Pp16	Pa6Pp17Pp18
Pa5Pp17	Pa3Pp17Pp19	Pa10Pp17	Pa9Pp17Pp19	Pa7Pp17	Pa6Pp17Pp19
Pa5Pp18	Pa3Pp17Pp20	Pa10Pp18	Pa9Pp17Pp20	Pa7Pp18	Pa6Pp17Pp20
Pa5Pp19	Pa3Pp18Pp19	Pa10Pp19	Pa9Pp18Pp19	Pa7Pp19	Pa6Pp18Pp19
Pa5Pp20	Pa3Pp18Pp20	Pa10Pp20	Pa9Pp18Pp20	Pa7Pp20	Pa6Pp18Pp20
Pp16Pp17	Pa3Pp19Pp20	Pp16Pp17	Pa9Pp19Pp20	Pp16Pp17	Pa6Pp19Pp20
Pp16Pp18	Pa5Pp16Pp17	Pp16Pp18	Pa10Pp16Pp17	Pp16Pp18	Pa7Pp16Pp17
Pp16Pp19	Pa5Pp16Pp18	Pp16Pp19	Pa10Pp16Pp18	Pp16Pp19	Pa7Pp16Pp18
Pp16Pp20	Pa5Pp16Pp19	Pp16Pp20	Pa10Pp16Pp19	Pp16Pp20	Pa7Pp16Pp19
Pp17Pp18	Pa5Pp16Pp20	Pp17Pp18	Pa10Pp16Pp20	Pp17Pp18	Pa7Pp16Pp20
Pp17Pp19	Pa5Pp17Pp18	Pp17Pp19	Pa10Pp17Pp18	Pp17Pp19	Pa7Pp17Pp18
Pp17Pp20	Pa5Pp17Pp19	Pp17Pp20	Pa10Pp17Pp19	Pp17Pp20	Pa7Pp17Pp19
Pp18Pp19	Pa5Pp17Pp20	Pp18Pp19	Pa10Pp17Pp20	Pp18Pp19	Pa7Pp17Pp20
Pp18Pp20	Pa5Pp18Pp19	Pp18Pp20	Pa10Pp18Pp19	Pp18Pp20	Pa7Pp18Pp19
Pp19Pp20	Pa5Pp18Pp20	Pp19Pp20	Pa10Pp18Pp20	Pp19Pp20	Pa7Pp18Pp20
	Pa5Pp19Pp20		Pa10Pp19Pp20		Pa7Pp19Pp20

Table S9. Color code corresponding to the coating layout in **Figure S35** and the **PaPp** coatings shown in **Figure S33m–o**.

Figure S33m		Figure S33n		Figure S33o	
Pp21	Pa1_{10}Pp21Pp22	Pp21	Pa1_{2}Pp21Pp22	Pp21	Pa2Pp21Pp22
Pp22	Pa1_{10}Pp21Pp23	Pp22	Pa1_{2}Pp21Pp23	Pp22	Pa2Pp21Pp23
Pp23	Pa1_{10}Pp21Pp24	Pp23	Pa1_{2}Pp21Pp24	Pp23	Pa2Pp21Pp24
Pp24	Pa1_{10}Pp21Pp25	Pp24	Pa1_{2}Pp21Pp25	Pp24	Pa2Pp21Pp25
Pp25	Pa1_{10}Pp22Pp23	Pp25	Pa1_{2}Pp22Pp23	Pp25	Pa2Pp22Pp23
Pa1_{10}	Pa1_{10}Pp22Pp24	Pa1_{2}	Pa1_{2}Pp22Pp24	Pa2	Pa2Pp22Pp24
Pa1_{10}Pp21	Pa1_{10}Pp22Pp25	Pa1_{2}Pp21	Pa1_{2}Pp22Pp25	Pa2Pp21	Pa2Pp22Pp25
Pa1_{10}Pp22	Pa1_{10}Pp23Pp24	Pa1_{2}Pp22	Pa1_{2}Pp23Pp24	Pa2Pp22	Pa2Pp23Pp24
Pa1_{10}Pp23	Pa1_{10}Pp23Pp25	Pa1_{2}Pp23	Pa1_{2}Pp23Pp25	Pa2Pp23	Pa2Pp23Pp25
Pa1_{10}Pp24	Pa1_{10}Pp24Pp25	Pa1_{2}Pp24	Pa1_{2}Pp24Pp25	Pa2Pp24	Pa2Pp24Pp25
Pa1_{10}Pp25	Pa1_{60}Pp21Pp22	Pa1_{2}Pp25	Pa8Pp21Pp22	Pa2Pp25	Pa4Pp21Pp22
Pa1_{60}	Pa1_{60}Pp21Pp23	Pa8	Pa8Pp21Pp23	Pa4	Pa4Pp21Pp23
Pa1_{60}Pp21	Pa1_{60}Pp21Pp24	Pa8Pp21	Pa8Pp21Pp24	Pa4Pp21	Pa4Pp21Pp24
Pa1_{60}Pp22	Pa1_{60}Pp21Pp25	Pa8Pp22	Pa8Pp21Pp25	Pa4Pp22	Pa4Pp21Pp25
Pa1_{60}Pp23	Pa1_{60}Pp22Pp23	Pa8Pp23	Pa8Pp22Pp23	Pa4Pp23	Pa4Pp22Pp23
Pa1_{60}Pp24	Pa1_{60}Pp22Pp24	Pa8Pp24	Pa8Pp22Pp24	Pa4Pp24	Pa4Pp22Pp24
Pa1_{60}Pp25	Pa1_{60}Pp22Pp25	Pa8Pp25	Pa8Pp22Pp25	Pa4Pp25	Pa4Pp22Pp25
Pa3	Pa1_{60}Pp23Pp24	Pa9	Pa8Pp23Pp24	Pa6	Pa4Pp23Pp24
Pa3Pp21	Pa1_{60}Pp23Pp24	Pa9Pp21	Pa8Pp23Pp24	Pa6Pp21	Pa4Pp23Pp24
Pa3Pp22	Pa1_{60}Pp24Pp25	Pa9Pp22	Pa8Pp24Pp25	Pa6Pp22	Pa4Pp24Pp25
Pa3Pp23	Pa3Pp21Pp22	Pa9Pp23	Pa9Pp21Pp22	Pa6Pp23	Pa6Pp21Pp22
Pa3Pp24	Pa3Pp21Pp23	Pa9Pp24	Pa9Pp21Pp23	Pa6Pp24	Pa6Pp21Pp23
Pa3Pp25	Pa3Pp21Pp24	Pa9Pp25	Pa9Pp21Pp24	Pa6Pp25	Pa6Pp21Pp24
Pa5	Pa3Pp21Pp25	Pa10	Pa9Pp21Pp25	Pa7	Pa6Pp21Pp25
Pa5Pp21	Pa3Pp22Pp23	Pa10Pp21	Pa9Pp22Pp23	Pa7Pp21	Pa6Pp22Pp23
Pa5Pp22	Pa3Pp22Pp24	Pa10Pp22	Pa9Pp22Pp24	Pa7Pp22	Pa6Pp22Pp24
Pa5Pp23	Pa3Pp22Pp25	Pa10Pp23	Pa9Pp22Pp25	Pa7Pp23	Pa6Pp22Pp25
Pa5Pp24	Pa3Pp23Pp24	Pa10Pp24	Pa9Pp23Pp24	Pa7Pp24	Pa6Pp23Pp24
Pa5Pp25	Pa3Pp23Pp25	Pa10Pp25	Pa9Pp23Pp25	Pa7Pp25	Pa6Pp23Pp25
Pp21Pp22	Pa3Pp24Pp25	Pp21Pp22	Pa9Pp24Pp25	Pp21Pp22	Pa6Pp24Pp25
Pp21Pp23	Pa5Pp21Pp22	Pp21Pp23	Pa10Pp21Pp22	Pp21Pp23	Pa7Pp21Pp22
Pp21Pp24	Pa5Pp21Pp23	Pp21Pp24	Pa10Pp21Pp23	Pp21Pp24	Pa7Pp21Pp23
Pp21Pp25	Pa5Pp21Pp24	Pp21Pp25	Pa10Pp21Pp24	Pp21Pp25	Pa7Pp21Pp24
Pp22Pp23	Pa5Pp21Pp25	Pp22Pp23	Pa10Pp21Pp25	Pp22Pp23	Pa7Pp21Pp25
Pp22Pp24	Pa5Pp22Pp23	Pp22Pp24	Pa10Pp22Pp23	Pp22Pp24	Pa7Pp22Pp23
Pp22Pp25	Pa5Pp22Pp24	Pp22Pp25	Pa10Pp22Pp24	Pp22Pp25	Pa7Pp22Pp24
Pp23Pp24	Pa5Pp22Pp25	Pp23Pp24	Pa10Pp22Pp25	Pp23Pp24	Pa7Pp22Pp25
Pp23Pp25	Pa5Pp23Pp24	Pp23Pp25	Pa10Pp23Pp24	Pp23Pp25	Pa7Pp23Pp24
Pp24Pp25	Pa5Pp23Pp25	Pp24Pp25	Pa10Pp23Pp25	Pp24Pp25	Pa7Pp23Pp25
	Pa5Pp24Pp25		Pa10Pp24Pp25		Pa7Pp24Pp25

Table S10. Color code corresponding to the coating layout in **Figure S35** and the **PaPp** coatings shown in **Figure S33p–r**.

Figure S33p		Figure S33q		Figure S33r	
Pp26	Pa1$_{10}$Pp26Pp27	Pp26	Pa1$_{2}$Pp26Pp27	Pp26	Pa2Pp26Pp27
Pp27	Pa1$_{10}$Pp26Pp28	Pp27	Pa1$_{2}$Pp26Pp28	Pp27	Pa2Pp26Pp28
Pp28	Pa1$_{10}$Pp26Pp29	Pp28	Pa1$_{2}$Pp26Pp29	Pp28	Pa2Pp26Pp29
Pp29	Pa1$_{10}$Pp26Pp30	Pp29	Pa1$_{2}$Pp26Pp30	Pp29	Pa2Pp26Pp30
Pp30	Pa1$_{10}$Pp27Pp28	Pp30	Pa1$_{2}$Pp27Pp28	Pp30	Pa2Pp27Pp28
Pa1$_{10}$	Pa1$_{10}$Pp27Pp29	Pa1$_{2}$	Pa1$_{2}$Pp27Pp29	Pa2	Pa2Pp27Pp29
Pa1$_{10}$Pp26	Pa1$_{10}$Pp27Pp30	Pa1$_{2}$Pp26	Pa1$_{2}$Pp27Pp30	Pa2Pp26	Pa2Pp27Pp30
Pa1$_{10}$Pp27	Pa1$_{10}$Pp28Pp29	Pa1$_{2}$Pp27	Pa1$_{2}$Pp28Pp29	Pa2Pp27	Pa2Pp28Pp29
Pa1$_{10}$Pp28	Pa1$_{10}$Pp28Pp30	Pa1$_{2}$Pp28	Pa1$_{2}$Pp28Pp30	Pa2Pp28	Pa2Pp28Pp30
Pa1$_{10}$Pp29	Pa1$_{10}$Pp29Pp30	Pa1$_{2}$Pp29	Pa1$_{2}$Pp29Pp30	Pa2Pp29	Pa2Pp29Pp30
Pa1$_{10}$Pp30	Pa1$_{60}$Pp26Pp27	Pa1$_{2}$Pp30	Pa8Pp26Pp27	Pa2Pp30	Pa4Pp26Pp27
Pa1$_{60}$	Pa1$_{60}$Pp26Pp28	Pa8	Pa8Pp26Pp28	Pa4	Pa4Pp26Pp28
Pa1$_{60}$Pp26	Pa1$_{60}$Pp26Pp29	Pa8Pp26	Pa8Pp26Pp29	Pa4Pp26	Pa4Pp26Pp29
Pa1$_{60}$Pp27	Pa1$_{60}$Pp26Pp30	Pa8Pp27	Pa8Pp26Pp30	Pa4Pp27	Pa4Pp26Pp30
Pa1$_{60}$Pp28	Pa1$_{60}$Pp27Pp28	Pa8Pp28	Pa8Pp27Pp28	Pa4Pp28	Pa4Pp27Pp28
Pa1$_{60}$Pp29	Pa1$_{60}$Pp27Pp29	Pa8Pp29	Pa8Pp27Pp29	Pa4Pp29	Pa4Pp27Pp29
Pa1$_{60}$Pp30	Pa1$_{60}$Pp27Pp30	Pa8Pp30	Pa8Pp27Pp30	Pa4Pp30	Pa4Pp27Pp30
Pa3	Pa1$_{60}$Pp28Pp29	Pa9	Pa8Pp28Pp29	Pa6	Pa4Pp28Pp29
Pa3Pp26	Pa1$_{60}$Pp28Pp29	Pa9Pp26	Pa8Pp28Pp29	Pa6Pp26	Pa4Pp28Pp29
Pa3Pp27	Pa1$_{60}$Pp29Pp30	Pa9Pp27	Pa8Pp29Pp30	Pa6Pp27	Pa4Pp29Pp30
Pa3Pp28	Pa3Pp26Pp27	Pa9Pp28	Pa9Pp26Pp27	Pa6Pp28	Pa6Pp26Pp27
Pa3Pp29	Pa3Pp26Pp28	Pa9Pp29	Pa9Pp26Pp28	Pa6Pp29	Pa6Pp26Pp28
Pa3Pp30	Pa3Pp26Pp29	Pa9Pp30	Pa9Pp26Pp29	Pa6Pp30	Pa6Pp26Pp29
Pa5	Pa3Pp26Pp30	Pa10	Pa9Pp26Pp30	Pa7	Pa6Pp26Pp30
Pa5Pp26	Pa3Pp27Pp28	Pa10Pp26	Pa9Pp27Pp28	Pa7Pp26	Pa6Pp27Pp28
Pa5Pp27	Pa3Pp27Pp29	Pa10Pp27	Pa9Pp27Pp29	Pa7Pp27	Pa6Pp27Pp29
Pa5Pp28	Pa3Pp27Pp30	Pa10Pp28	Pa9Pp27Pp30	Pa7Pp28	Pa6Pp27Pp30
Pa5Pp29	Pa3Pp28Pp29	Pa10Pp29	Pa9Pp28Pp29	Pa7Pp29	Pa6Pp28Pp29
Pa5Pp30	Pa3Pp28Pp30	Pa10Pp30	Pa9Pp28Pp30	Pa7Pp30	Pa6Pp28Pp30
Pp26Pp27	Pa3Pp29Pp30	Pp26Pp27	Pa9Pp29Pp30	Pp26Pp27	Pa6Pp29Pp30
Pp26Pp28	Pa5Pp26Pp27	Pp26Pp28	Pa10Pp26Pp27	Pp26Pp28	Pa7Pp26Pp27
Pp26Pp29	Pa5Pp26Pp28	Pp26Pp29	Pa10Pp26Pp28	Pp26Pp29	Pa7Pp26Pp28
Pp26Pp30	Pa5Pp26Pp29	Pp26Pp30	Pa10Pp26Pp29	Pp26Pp30	Pa7Pp26Pp29
Pp27Pp28	Pa5Pp26Pp30	Pp27Pp28	Pa10Pp26Pp30	Pp27Pp28	Pa7Pp26Pp30
Pp27Pp29	Pa5Pp27Pp28	Pp27Pp29	Pa10Pp27Pp28	Pp27Pp29	Pa7Pp27Pp28
Pp27Pp30	Pa5Pp27Pp29	Pp27Pp30	Pa10Pp27Pp29	Pp27Pp30	Pa7Pp27Pp29
Pp28Pp29	Pa5Pp27Pp30	Pp28Pp29	Pa10Pp27Pp30	Pp28Pp29	Pa7Pp27Pp30
Pp28Pp30	Pa5Pp28Pp29	Pp28Pp30	Pa10Pp28Pp29	Pp28Pp30	Pa7Pp28Pp29
Pp29Pp30	Pa5Pp28Pp30	Pp29Pp30	Pa10Pp28Pp30	Pp29Pp30	Pa7Pp28Pp30
	Pa5Pp29Pp30		Pa10Pp29Pp30		Pa7Pp29Pp30

Table S11. Color code corresponding to the coating layout in **Figure S35** and the **PaPp** coatings shown in **Figure S34a–c**.

Figure S34a		Figure S34b		Figure S34c	
Pp31	Pa1_{10}Pp31Pp32	Pp31	Pa1_{2}Pp31Pp32	Pp31	Pa2Pp31Pp32
Pp32	Pa1_{10}Pp31Pp33	Pp32	Pa1_{2}Pp31Pp33	Pp32	Pa2Pp31Pp33
Pp33	Pa1_{10}Pp31Pp34	Pp33	Pa1_{2}Pp31Pp34	Pp33	Pa2Pp31Pp34
Pp34	Pa1_{10}Pp31Pp35	Pp34	Pa1_{2}Pp31Pp35	Pp34	Pa2Pp31Pp35
Pp35	Pa1_{10}Pp32Pp33	Pp35	Pa1_{2}Pp32Pp33	Pp35	Pa2Pp32Pp33
Pa1_{10}	Pa1_{10}Pp32Pp34	Pa1_{2}	Pa1_{2}Pp32Pp34	Pa2	Pa2Pp32Pp34
Pa1_{10}Pp31	Pa1_{10}Pp32Pp35	Pa1_{2}Pp31	Pa1_{2}Pp32Pp35	Pa2Pp31	Pa2Pp32Pp35
Pa1_{10}Pp32	Pa1_{10}Pp33Pp34	Pa1_{2}Pp32	Pa1_{2}Pp33Pp34	Pa2Pp32	Pa2Pp33Pp34
Pa1_{10}Pp33	Pa1_{10}Pp33Pp35	Pa1_{2}Pp33	Pa1_{2}Pp33Pp35	Pa2Pp33	Pa2Pp33Pp35
Pa1_{10}Pp34	Pa1_{10}Pp34Pp35	Pa1_{2}Pp34	Pa1_{2}Pp34Pp35	Pa2Pp34	Pa2Pp34Pp35
Pa1_{10}Pp35	Pa1_{60}Pp31Pp32	Pa1_{2}Pp35	Pa8Pp31Pp32	Pa2Pp35	Pa4Pp31Pp32
Pa1_{60}	Pa1_{60}Pp31Pp33	Pa8	Pa8Pp31Pp33	Pa4	Pa4Pp31Pp33
Pa1_{60}Pp31	Pa1_{60}Pp31Pp34	Pa8Pp31	Pa8Pp31Pp34	Pa4Pp31	Pa4Pp31Pp34
Pa1_{60}Pp32	Pa1_{60}Pp31Pp35	Pa8Pp32	Pa8Pp31Pp35	Pa4Pp32	Pa4Pp31Pp35
Pa1_{60}Pp33	Pa1_{60}Pp32Pp33	Pa8Pp33	Pa8Pp32Pp33	Pa4Pp33	Pa4Pp32Pp33
Pa1_{60}Pp34	Pa1_{60}Pp32Pp34	Pa8Pp34	Pa8Pp32Pp34	Pa4Pp34	Pa4Pp32Pp34
Pa1_{60}Pp35	Pa1_{60}Pp32Pp35	Pa8Pp35	Pa8Pp32Pp35	Pa4Pp35	Pa4Pp32Pp35
Pa3	Pa1_{60}Pp33Pp34	Pa9	Pa8Pp33Pp34	Pa6	Pa4Pp33Pp34
Pa3Pp31	Pa1_{60}Pp33Pp35	Pa9Pp31	Pa8Pp33Pp35	Pa6Pp31	Pa4Pp33Pp35
Pa3Pp32	Pa1_{60}Pp34Pp35	Pa9Pp32	Pa8Pp34Pp35	Pa6Pp32	Pa4Pp34Pp35
Pa3Pp33	Pa3Pp31Pp32	Pa9Pp33	Pa9Pp31Pp32	Pa6Pp33	Pa6Pp31Pp32
Pa3Pp34	Pa3Pp31Pp33	Pa9Pp34	Pa9Pp31Pp33	Pa6Pp34	Pa6Pp31Pp33
Pa3Pp35	Pa3Pp31Pp34	Pa9Pp35	Pa9Pp31Pp34	Pa6Pp35	Pa6Pp31Pp34
Pa5	Pa3Pp31Pp35	Pa10	Pa9Pp31Pp35	Pa7	Pa6Pp31Pp35
Pa5Pp31	Pa3Pp32Pp33	Pa10Pp31	Pa9Pp32Pp33	Pa7Pp31	Pa6Pp32Pp33
Pa5Pp32	Pa3Pp32Pp34	Pa10Pp32	Pa9Pp32Pp34	Pa7Pp32	Pa6Pp32Pp34
Pa5Pp33	Pa3Pp32Pp35	Pa10Pp33	Pa9Pp32Pp35	Pa7Pp33	Pa6Pp32Pp35
Pa5Pp34	Pa3Pp33Pp34	Pa10Pp34	Pa9Pp33Pp34	Pa7Pp34	Pa6Pp33Pp34
Pa5Pp35	Pa3Pp33Pp35	Pa10Pp35	Pa9Pp33Pp35	Pa7Pp35	Pa6Pp33Pp35
Pp31Pp32	Pa3Pp34Pp35	Pp31Pp32	Pa9Pp34Pp35	Pp31Pp32	Pa6Pp34Pp35
Pp31Pp33	Pa5Pp31Pp32	Pp31Pp33	Pa10Pp31Pp32	Pp31Pp33	Pa7Pp31Pp32
Pp31Pp34	Pa5Pp31Pp33	Pp31Pp34	Pa10Pp31Pp33	Pp31Pp34	Pa7Pp31Pp33
Pp31Pp35	Pa5Pp31Pp34	Pp31Pp35	Pa10Pp31Pp34	Pp31Pp35	Pa7Pp31Pp34
Pp32Pp33	Pa5Pp31Pp35	Pp32Pp33	Pa10Pp31Pp35	Pp32Pp33	Pa7Pp31Pp35
Pp32Pp34	Pa5Pp32Pp33	Pp32Pp34	Pa10Pp32Pp33	Pp32Pp34	Pa7Pp32Pp33
Pp32Pp35	Pa5Pp32Pp34	Pp32Pp35	Pa10Pp32Pp34	Pp32Pp35	Pa7Pp32Pp34
Pp33Pp34	Pa5Pp32Pp35	Pp33Pp34	Pa10Pp32Pp35	Pp33Pp34	Pa7Pp32Pp35
Pp33Pp35	Pa5Pp33Pp34	Pp33Pp35	Pa10Pp33Pp34	Pp33Pp35	Pa7Pp33Pp34
Pp34Pp35	Pa5Pp33Pp35	Pp34Pp35	Pa10Pp33Pp35	Pp34Pp35	Pa7Pp33Pp35
	Pa5Pp34Pp35		Pa10Pp34Pp35		Pa7Pp34Pp35

Table S12. Color code corresponding to the coating layout in **Figure S35** and the **PaPp** coatings shown in **Figure S34d–f**.

Figure S34d		Figure S34e		Figure S34f	
Pp36	Pa1$_{10}$Pp36Pp37	Pp36	Pa1$_{2}$Pp36Pp37	Pp36	Pa2Pp36Pp37
Pp37	Pa1$_{10}$Pp36Pp38	Pp37	Pa1$_{2}$Pp36Pp38	Pp37	Pa2Pp36Pp38
Pp38	Pa1$_{10}$Pp36Pp39	Pp38	Pa1$_{2}$Pp36Pp39	Pp38	Pa2Pp36Pp39
Pp39	Pa1$_{10}$Pp36Pp40	Pp39	Pa1$_{2}$Pp36Pp40	Pp39	Pa2Pp36Pp40
Pp40	Pa1$_{10}$Pp37Pp38	Pp40	Pa1$_{2}$Pp37Pp38	Pp40	Pa2Pp37Pp38
Pa1$_{10}$	Pa1$_{10}$Pp37Pp39	Pa1$_{2}$	Pa1$_{2}$Pp37Pp39	Pa2	Pa2Pp37Pp39
Pa1$_{10}$Pp36	Pa1$_{10}$Pp37Pp40	Pa1$_{2}$Pp36	Pa1$_{2}$Pp37Pp40	Pa2Pp36	Pa2Pp37Pp40
Pa1$_{10}$Pp37	Pa1$_{10}$Pp38Pp39	Pa1$_{2}$Pp37	Pa1$_{2}$Pp38Pp39	Pa2Pp37	Pa2Pp38Pp39
Pa1$_{10}$Pp38	Pa1$_{10}$Pp38Pp40	Pa1$_{2}$Pp38	Pa1$_{2}$Pp38Pp40	Pa2Pp38	Pa2Pp38Pp40
Pa1$_{10}$Pp39	Pa1$_{10}$Pp39Pp40	Pa1$_{2}$Pp39	Pa1$_{2}$Pp39Pp40	Pa2Pp39	Pa2Pp39Pp40
Pa1$_{10}$Pp40	Pa1$_{60}$Pp36Pp37	Pa1$_{2}$Pp40	Pa8Pp36Pp37	Pa2Pp40	Pa4Pp36Pp37
Pa1$_{60}$	Pa1$_{60}$Pp36Pp38	Pa8	Pa8Pp36Pp38	Pa4	Pa4Pp36Pp38
Pa1$_{60}$Pp36	Pa1$_{60}$Pp36Pp39	Pa8Pp36	Pa8Pp36Pp39	Pa4Pp36	Pa4Pp36Pp39
Pa1$_{60}$Pp37	Pa1$_{60}$Pp36Pp40	Pa8Pp37	Pa8Pp36Pp40	Pa4Pp37	Pa4Pp36Pp40
Pa1$_{60}$Pp38	Pa1$_{60}$Pp37Pp38	Pa8Pp38	Pa8Pp37Pp38	Pa4Pp38	Pa4Pp37Pp38
Pa1$_{60}$Pp39	Pa1$_{60}$Pp37Pp39	Pa8Pp39	Pa8Pp37Pp39	Pa4Pp39	Pa4Pp37Pp39
Pa1$_{60}$Pp40	Pa1$_{60}$Pp37Pp40	Pa8Pp40	Pa8Pp37Pp40	Pa4Pp40	Pa4Pp37Pp40
Pa3	Pa1$_{60}$Pp38Pp39	Pa9	Pa8Pp38Pp39	Pa6	Pa4Pp38Pp39
Pa3Pp36	Pa1$_{60}$Pp38Pp40	Pa9Pp36	Pa8Pp38Pp40	Pa6Pp36	Pa4Pp38Pp40
Pa3Pp37	Pa1$_{60}$Pp39Pp40	Pa9Pp37	Pa8Pp39Pp40	Pa6Pp37	Pa4Pp39Pp40
Pa3Pp38	Pa3Pp36Pp37	Pa9Pp38	Pa9Pp36Pp37	Pa6Pp38	Pa6Pp36Pp37
Pa3Pp39	Pa3Pp36Pp38	Pa9Pp39	Pa9Pp36Pp38	Pa6Pp39	Pa6Pp36Pp38
Pa3Pp40	Pa3Pp36Pp39	Pa9Pp40	Pa9Pp36Pp39	Pa6Pp40	Pa6Pp36Pp39
Pa5	Pa3Pp36Pp40	Pa10	Pa9Pp36Pp40	Pa7	Pa6Pp36Pp40
Pa5Pp36	Pa3Pp37Pp38	Pa10Pp36	Pa9Pp37Pp38	Pa7Pp36	Pa6Pp37Pp38
Pa5Pp37	Pa3Pp37Pp39	Pa10Pp37	Pa9Pp37Pp39	Pa7Pp37	Pa6Pp37Pp39
Pa5Pp38	Pa3Pp37Pp40	Pa10Pp38	Pa9Pp37Pp40	Pa7Pp38	Pa6Pp37Pp40
Pa5Pp39	Pa3Pp38Pp39	Pa10Pp39	Pa9Pp38Pp39	Pa7Pp39	Pa6Pp38Pp39
Pa5Pp40	Pa3Pp38Pp40	Pa10Pp40	Pa9Pp38Pp40	Pa7Pp40	Pa6Pp38Pp40
Pp36Pp37	Pa3Pp39Pp40	Pp36Pp37	Pa9Pp39Pp40	Pp36Pp37	Pa6Pp39Pp40
Pp36Pp38	Pa5Pp36Pp37	Pp36Pp38	Pa10Pp36Pp37	Pp36Pp38	Pa7Pp36Pp37
Pp36Pp39	Pa5Pp36Pp38	Pp36Pp39	Pa10Pp36Pp38	Pp36Pp39	Pa7Pp36Pp38
Pp36Pp40	Pa5Pp36Pp39	Pp36Pp40	Pa10Pp36Pp39	Pp36Pp40	Pa7Pp36Pp39
Pp37Pp38	Pa5Pp36Pp40	Pp37Pp38	Pa10Pp36Pp40	Pp37Pp38	Pa7Pp36Pp40
Pp37Pp39	Pa5Pp37Pp38	Pp37Pp39	Pa10Pp37Pp38	Pp37Pp39	Pa7Pp37Pp38
Pp37Pp40	Pa5Pp37Pp39	Pp37Pp40	Pa10Pp37Pp39	Pp37Pp40	Pa7Pp37Pp39
Pp38Pp39	Pa5Pp37Pp40	Pp38Pp39	Pa10Pp37Pp40	Pp38Pp39	Pa7Pp37Pp40
Pp38Pp40	Pa5Pp38Pp39	Pp38Pp40	Pa10Pp38Pp39	Pp38Pp40	Pa7Pp38Pp39
Pp39Pp40	Pa5Pp38Pp40	Pp39Pp40	Pa10Pp38Pp40	Pp39Pp40	Pa7Pp38Pp40
	Pa5Pp39Pp40		Pa10Pp39Pp40		Pa7Pp39Pp40

Table S13. Color code corresponding to the coating layout in **Figure S35** and the **PaPp** coatings shown in **Figure S34g–i**.

Figure S34g		Figure S34h		Figure S34i	
Pp41	Pa1$_{10}$Pp41Pp42	Pp41	Pa1$_{2}$Pp41Pp42	Pp41	Pa2Pp41Pp42
Pp42	Pa1$_{10}$Pp41Pp43	Pp42	Pa1$_{2}$Pp41Pp43	Pp42	Pa2Pp41Pp43
Pp43	Pa1$_{10}$Pp41Pp44	Pp43	Pa1$_{2}$Pp41Pp44	Pp43	Pa2Pp41Pp44
Pp44	Pa1$_{10}$Pp41Pp45	Pp44	Pa1$_{2}$Pp41Pp45	Pp44	Pa2Pp41Pp45
Pp45	Pa1$_{10}$Pp42Pp43	Pp45	Pa1$_{2}$Pp42Pp43	Pp45	Pa2Pp42Pp43
Pa1$_{10}$	Pa1$_{10}$Pp42Pp44	Pa1$_{2}$	Pa1$_{2}$Pp42Pp44	Pa2	Pa2Pp42Pp44
Pa1$_{10}$Pp41	Pa1$_{10}$Pp42Pp45	Pa1$_{2}$Pp41	Pa1$_{2}$Pp42Pp45	Pa2Pp41	Pa2Pp42Pp45
Pa1$_{10}$Pp42	Pa1$_{10}$Pp43Pp44	Pa1$_{2}$Pp42	Pa1$_{2}$Pp43Pp44	Pa2Pp42	Pa2Pp43Pp44
Pa1$_{10}$Pp43	Pa1$_{10}$Pp43Pp45	Pa1$_{2}$Pp43	Pa1$_{2}$Pp43Pp45	Pa2Pp43	Pa2Pp43Pp45
Pa1$_{10}$Pp44	Pa1$_{10}$Pp44Pp45	Pa1$_{2}$Pp44	Pa1$_{2}$Pp44Pp45	Pa2Pp44	Pa2Pp44Pp45
Pa1$_{10}$Pp45	Pa1$_{60}$Pp41Pp42	Pa1$_{2}$Pp45	Pa8Pp41Pp42	Pa2Pp45	Pa4Pp41Pp42
Pa1$_{60}$	Pa1$_{60}$Pp41Pp43	Pa8	Pa8Pp41Pp43	Pa4	Pa4Pp41Pp43
Pa1$_{60}$Pp41	Pa1$_{60}$Pp41Pp44	Pa8Pp41	Pa8Pp41Pp44	Pa4Pp41	Pa4Pp41Pp44
Pa1$_{60}$Pp42	Pa1$_{60}$Pp41Pp45	Pa8Pp42	Pa8Pp41Pp45	Pa4Pp42	Pa4Pp41Pp45
Pa1$_{60}$Pp43	Pa1$_{60}$Pp42Pp43	Pa8Pp43	Pa8Pp42Pp43	Pa4Pp43	Pa4Pp42Pp43
Pa1$_{60}$Pp44	Pa1$_{60}$Pp42Pp44	Pa8Pp44	Pa8Pp42Pp44	Pa4Pp44	Pa4Pp42Pp44
Pa1$_{60}$Pp45	Pa1$_{60}$Pp42Pp45	Pa8Pp45	Pa8Pp42Pp45	Pa4Pp45	Pa4Pp42Pp45
Pa3	Pa1$_{60}$Pp43Pp44	Pa9	Pa8Pp43Pp44	Pa6	Pa4Pp43Pp44
Pa3Pp41	Pa1$_{60}$Pp43Pp45	Pa9Pp41	Pa8Pp43Pp45	Pa6Pp41	Pa4Pp43Pp45
Pa3Pp42	Pa1$_{60}$Pp44Pp45	Pa9Pp42	Pa8Pp44Pp45	Pa6Pp42	Pa4Pp44Pp45
Pa3Pp43	Pa3Pp41Pp42	Pa9Pp43	Pa9Pp41Pp42	Pa6Pp43	Pa6Pp41Pp42
Pa3Pp44	Pa3Pp41Pp43	Pa9Pp44	Pa9Pp41Pp43	Pa6Pp44	Pa6Pp41Pp43
Pa3Pp45	Pa3Pp41Pp44	Pa9Pp45	Pa9Pp41Pp44	Pa6Pp45	Pa6Pp41Pp44
Pa5	Pa3Pp41Pp45	Pa10	Pa9Pp41Pp45	Pa7	Pa6Pp41Pp45
Pa5Pp41	Pa3Pp42Pp43	Pa10Pp41	Pa9Pp42Pp43	Pa7Pp41	Pa6Pp42Pp43
Pa5Pp42	Pa3Pp42Pp44	Pa10Pp42	Pa9Pp42Pp44	Pa7Pp42	Pa6Pp42Pp44
Pa5Pp43	Pa3Pp42Pp45	Pa10Pp43	Pa9Pp42Pp45	Pa7Pp43	Pa6Pp42Pp45
Pa5Pp44	Pa3Pp43Pp44	Pa10Pp44	Pa9Pp43Pp44	Pa7Pp44	Pa6Pp43Pp44
Pa5Pp45	Pa3Pp43Pp45	Pa10Pp45	Pa9Pp43Pp45	Pa7Pp45	Pa6Pp43Pp45
Pp41Pp42	Pa3Pp44Pp45	Pp41Pp42	Pa9Pp44Pp45	Pp41Pp42	Pa6Pp44Pp45
Pp41Pp43	Pa5Pp41Pp42	Pp41Pp43	Pa10Pp41Pp42	Pp41Pp43	Pa7Pp41Pp42
Pp41Pp44	Pa5Pp41Pp43	Pp41Pp44	Pa10Pp41Pp43	Pp41Pp44	Pa7Pp41Pp43
Pp41Pp45	Pa5Pp41Pp44	Pp41Pp45	Pa10Pp41Pp44	Pp41Pp45	Pa7Pp41Pp44
Pp42Pp43	Pa5Pp41Pp45	Pp42Pp43	Pa10Pp41Pp45	Pp42Pp43	Pa7Pp41Pp45
Pp42Pp44	Pa5Pp42Pp43	Pp42Pp44	Pa10Pp42Pp43	Pp42Pp44	Pa7Pp42Pp43
Pp42Pp45	Pa5Pp42Pp44	Pp42Pp45	Pa10Pp42Pp44	Pp42Pp45	Pa7Pp42Pp44
Pp43Pp44	Pa5Pp42Pp45	Pp43Pp44	Pa10Pp42Pp45	Pp43Pp44	Pa7Pp42Pp45
Pp43Pp45	Pa5Pp43Pp44	Pp43Pp45	Pa10Pp43Pp44	Pp43Pp45	Pa7Pp43Pp44
Pp44Pp45	Pa5Pp43Pp45	Pp44Pp45	Pa10Pp43Pp45	Pp44Pp45	Pa7Pp43Pp45
	Pa5Pp44Pp45		Pa10Pp44Pp45		Pa7Pp44Pp45

Table S14. Color code corresponding to the coating layout in **Figure S35** and the **PaPp** coatings shown in **Figure S34j–m**.

Figure S34j		Figure S34k		Figure S34l	
Pp46	Pa1$_{10}$Pp46Pp51	Pa1$_{2}$	Pa3Pp48Pp49	Pa9	Pa9Pp50Pp51
Pp47	Pa1$_{10}$Pp47Pp48	Pa1$_{2}$Pp46	Pa3Pp48Pp50	Pa9Pp46	Pa10Pp46Pp47
Pp48	Pa1$_{10}$Pp47Pp49	Pa1$_{2}$Pp47	Pa3Pp48Pp51	Pa9Pp47	Pa10Pp46Pp48
Pp49	Pa1$_{10}$Pp47Pp50	Pa1$_{2}$Pp48	Pa3Pp49Pp50	Pa9Pp48	Pa10Pp46Pp49
Pp50	Pa1$_{10}$Pp47Pp51	Pa1$_{2}$Pp49	Pa3Pp49Pp51	Pa9Pp49	Pa10Pp46Pp50
Pp51	Pa1$_{10}$Pp48Pp49	Pa1$_{2}$Pp50	Pa3Pp50Pp51	Pa9Pp50	Pa10Pp46Pp51
Pa1$_{10}$	Pa1$_{10}$Pp48Pp50	Pa1$_{2}$Pp51	Pa5Pp46Pp47	Pa9Pp51	Pa10Pp47Pp48
Pa1$_{10}$Pp46	Pa1$_{10}$Pp48Pp51	Pa3	Pa5Pp46Pp48	Pa10	Pa10Pp47Pp49
Pa1$_{10}$Pp47	Pa1$_{10}$Pp49Pp50	Pa3Pp46	Pa5Pp46Pp49	Pa10Pp46	Pa10Pp47Pp50
Pa1$_{10}$Pp48	Pa1$_{10}$Pp49Pp51	Pa3Pp47	Pa5Pp46Pp50	Pa10Pp47	Pa10Pp47Pp51
Pa1$_{10}$Pp49	Pa1$_{10}$Pp50Pp51	Pa3Pp48	Pa5Pp46Pp51	Pa10Pp48	Pa10Pp48Pp49
Pa1$_{10}$Pp50	Pa1$_{60}$Pp46Pp47	Pa3Pp49	Pa5Pp47Pp48	Pa10Pp49	Pa10Pp48Pp50
Pa1$_{10}$Pp51	Pa1$_{60}$Pp46Pp48	Pa3Pp50	Pa5Pp47Pp49	Pa10Pp50	Pa10Pp48Pp51
Pa1$_{60}$	Pa1$_{60}$Pp46Pp49	Pa3Pp51	Pa5Pp47Pp50	Pa10Pp51	Pa10Pp49Pp50
Pa1$_{60}$Pp46	Pa1$_{60}$Pp46Pp50	Pa5	Pa5Pp47Pp51	Pa2	Pa10Pp49Pp51
Pa1$_{60}$Pp47	Pa1$_{60}$Pp46Pp51	Pa5Pp46	Pa5Pp48Pp49	Pa2Pp46	Pa10Pp50Pp51
Pa1$_{60}$Pp48	Pa1$_{60}$Pp47Pp48	Pa5Pp47	Pa5Pp48Pp50	Pa2Pp47	Pa2Pp46Pp47
Pa1$_{60}$Pp49	Pa1$_{60}$Pp47Pp49	Pa5Pp48	Pa5Pp48Pp51	Pa2Pp48	Pa2Pp46Pp48
Pa1$_{60}$Pp50	Pa1$_{60}$Pp47Pp50	Pa5Pp49	Pa5Pp49Pp50	Pa2Pp49	Pa2Pp46Pp49
Pa1$_{60}$Pp51	Pa1$_{60}$Pp47Pp51	Pa5Pp50	Pa5Pp49Pp51	Pa2Pp50	Pa2Pp46Pp50
Pp46Pp47	Pa1$_{60}$Pp48Pp49	Pa5Pp51	Pa5Pp50Pp51	Pa2Pp51	Pa2Pp46Pp51
Pp46Pp48	Pa1$_{60}$Pp48Pp50	Pa8	Pa8Pp46Pp47	-	Pa2Pp47Pp48
Pp46Pp49	Pa1$_{60}$Pp48Pp51	Pa8Pp46	Pa8Pp46Pp48	-	Pa2Pp47Pp49
Pp46Pp50	Pa1$_{60}$Pp49Pp50	Pa8Pp47	Pa8Pp46Pp49	-	Pa2Pp47Pp50
Pp46Pp51	Pa1$_{60}$Pp49Pp51	Pa8Pp48	Pa8Pp46Pp50	-	Pa2Pp47Pp51
Pp47Pp48	Pa1$_{60}$Pp50Pp51	Pa8Pp49	Pa8Pp46Pp51	Pa9Pp46Pp47	Pa2Pp48Pp49
Pp47Pp49	Pa1$_{2}$Pp46Pp47	Pa8Pp50	Pa8Pp47Pp48	Pa9Pp46Pp48	Pa2Pp48Pp50
Pp47Pp50	Pa1$_{2}$Pp46Pp48	Pa8Pp51	Pa8Pp47Pp49	Pa9Pp46Pp49	Pa2Pp48Pp51
Pp47Pp51	Pa1$_{2}$Pp46Pp49	-	Pa8Pp47Pp50	Pa9Pp46Pp50	Pa2Pp49Pp50
Pp48Pp49	Pa1$_{2}$Pp46Pp50	-	Pa8Pp47Pp51	Pa9Pp46Pp51	Pa2Pp49Pp51
Pp48Pp50	Pa1$_{2}$Pp46Pp51	Pa3Pp46Pp47	Pa8Pp48Pp49	Pa9Pp47Pp48	Pa2Pp50Pp51
Pp48Pp51	Pa1$_{2}$Pp47Pp48	Pa3Pp46Pp48	Pa8Pp48Pp50	Pa9Pp47Pp49	
Pp49Pp50	Pa1$_{2}$Pp47Pp49	Pa3Pp46Pp49	Pa8Pp48Pp51	Pa9Pp47Pp50	
Pp49Pp51	Pa1$_{2}$Pp47Pp50	Pa3Pp46Pp50	Pa8Pp49Pp50	Pa9Pp47Pp51	
Pp50Pp51	Pa1$_{2}$Pp47Pp51	Pa3Pp46Pp51	Pa8Pp49Pp51	Pa9Pp48Pp49	
Pa1$_{10}$Pp46Pp47	Pa1$_{2}$Pp48Pp49	Pa3Pp47Pp48	Pa8Pp50Pp51	Pa9Pp48Pp50	
Pa1$_{10}$Pp46Pp48	Pa1$_{2}$Pp48Pp50	Pa3Pp47Pp49		Pa9Pp48Pp51	
Pa1$_{10}$Pp46Pp49	Pa1$_{2}$Pp48Pp51	Pa3Pp47Pp50		Pa9Pp49Pp50	
Pa1$_{10}$Pp46Pp50	Pa1$_{2}$Pp49Pp50	Pa3Pp47Pp51		Pa9Pp49Pp51	
	Pa1$_{2}$Pp49Pp51				
	Pa1$_{2}$Pp50Pp51				

Figure S34m			
Pa4	Pa4Pp50Pp51	-	Pa7Pp47Pp48
Pa4Pp46	Pa6Pp46Pp47	-	Pa7Pp47Pp49
Pa4Pp47	Pa6Pp46Pp48	-	Pa7Pp47Pp50
Pa4Pp48	Pa6Pp46Pp49	-	Pa7Pp47Pp51
Pa4Pp49	Pa6Pp46Pp50	Pa4Pp46Pp47	Pa7Pp48Pp49
Pa4Pp50	Pa6Pp46Pp51	Pa4Pp46Pp48	Pa7Pp48Pp50
Pa4Pp51	Pa6Pp47Pp48	Pa4Pp46Pp49	Pa7Pp48Pp51
Pa6	Pa6Pp47Pp49	Pa4Pp46Pp50	Pa7Pp49Pp50
Pa6Pp46	Pa6Pp47Pp50	Pa4Pp46Pp51	Pa7Pp49Pp51
Pa6Pp47	Pa6Pp47Pp51	Pa4Pp47Pp48	Pa7Pp50Pp51
Pa6Pp48	Pa6Pp48Pp49	Pa4Pp47Pp49	
Pa6Pp49	Pa6Pp48Pp50	Pa4Pp47Pp50	
Pa6Pp50	Pa6Pp48Pp51	Pa4Pp47Pp51	
Pa6Pp51	Pa6Pp49Pp50	Pa4Pp48Pp49	
Pa7	Pa6Pp49Pp51	Pa4Pp48Pp50	
Pa7Pp46	Pa6Pp50Pp51	Pa4Pp48Pp51	
Pa7Pp47	Pa7Pp46Pp47	Pa4Pp49Pp50	
Pa7Pp48	Pa7Pp46Pp48	Pa4Pp49Pp51	
Pa7Pp49	Pa7Pp46Pp49		
Pa7Pp50	Pa7Pp46Pp50		
Pa7Pp51	Pa7Pp46Pp51		

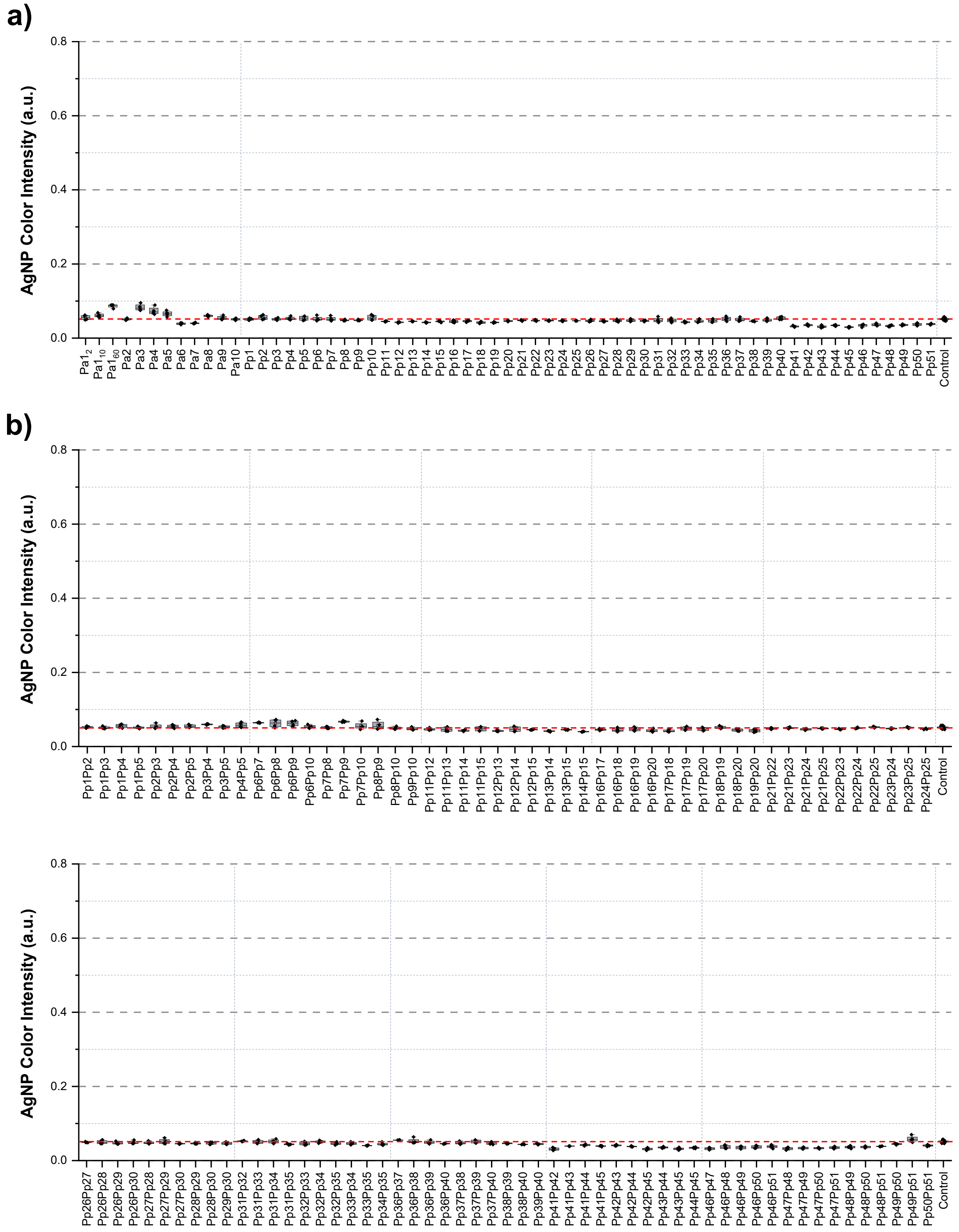

Figure S36. UHT screening of silver-reducing activity. Color intensities of AgNPs formed by a) individual precursors (**Pa1$_2$-Pa10, Pp1-Pp51**) and b) binary combinations (**PpPp**) derived from **Pp1-Pp51**. Uncoated spots served as controls.

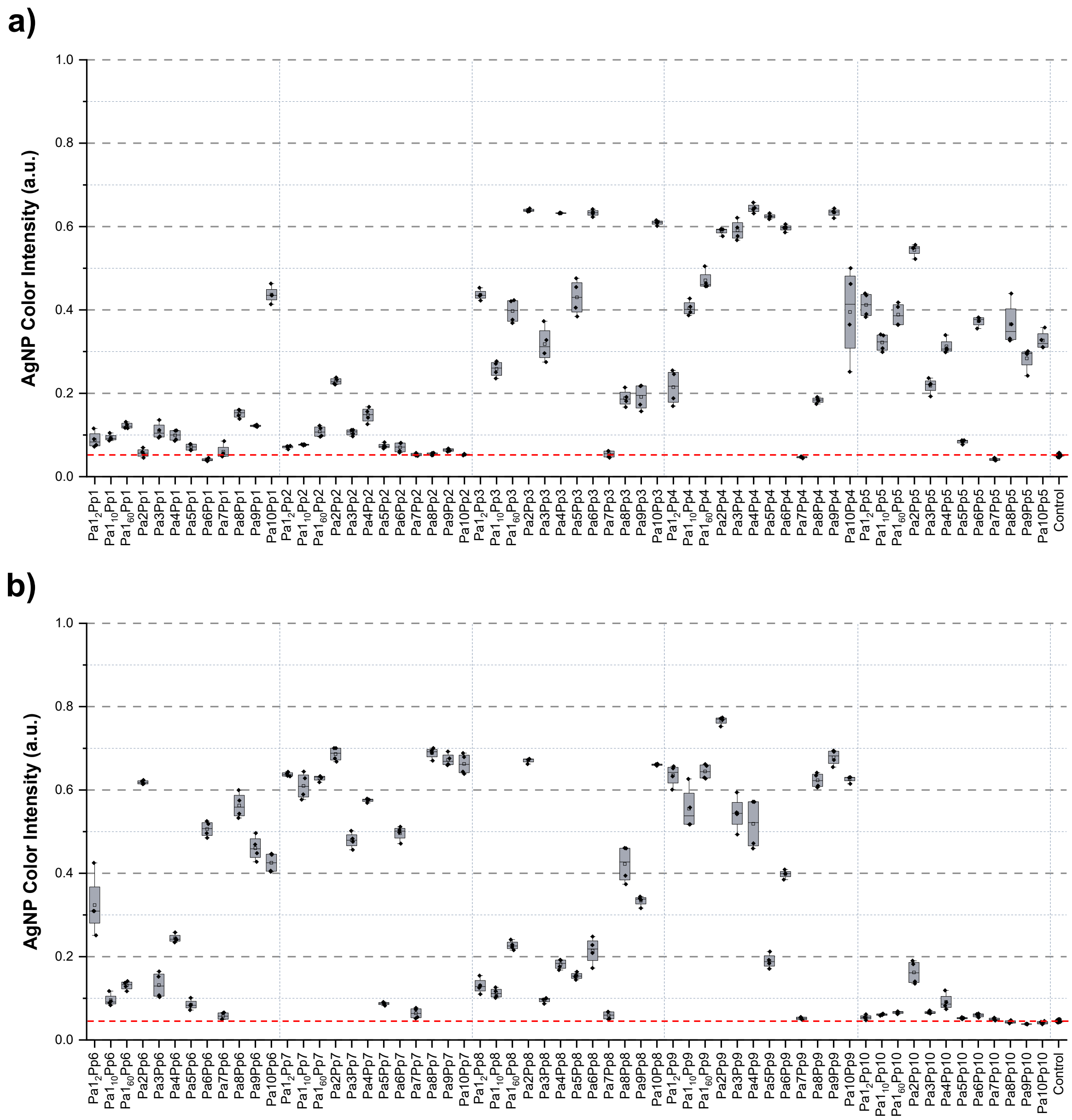

Figure S37. UHT screening of silver-reducing activity. Shown are the color intensities of AgNPs formed on **PaPp** coatings prepared from **Pa1$_2$-Pa10** crosslinked with a) **Pp1-Pp5** and b) **Pp6-Pp10**. Uncoated spots served as controls.

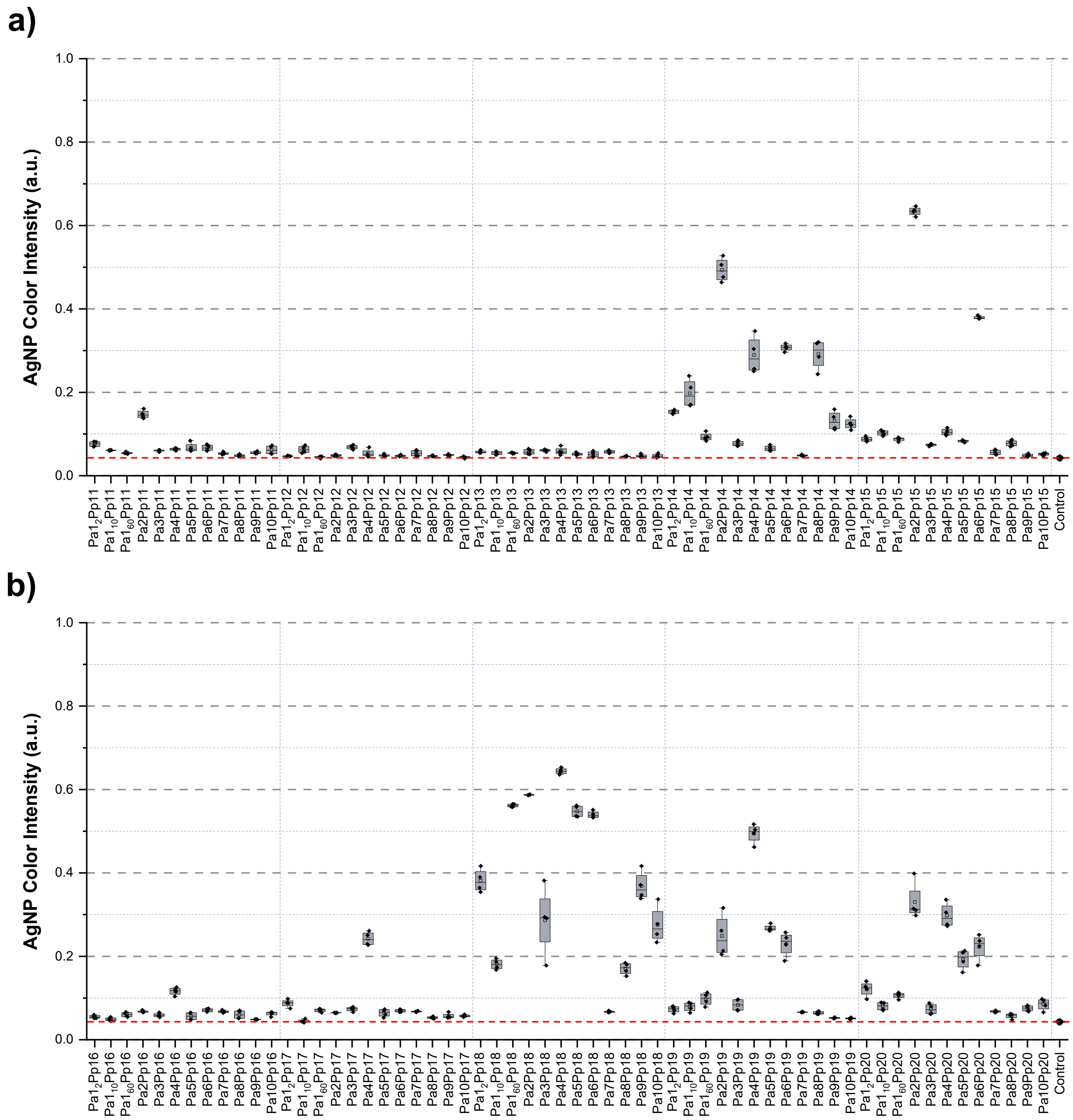

Figure S38. UHT screening of silver-reducing activity. Shown are the color intensities of AgNPs formed on **PaPp** coatings prepared from **Pa1$_2$-Pa10** crosslinked with a) **Pp11-Pp15** and b) **Pp16-Pp20**. Uncoated spots served as controls.

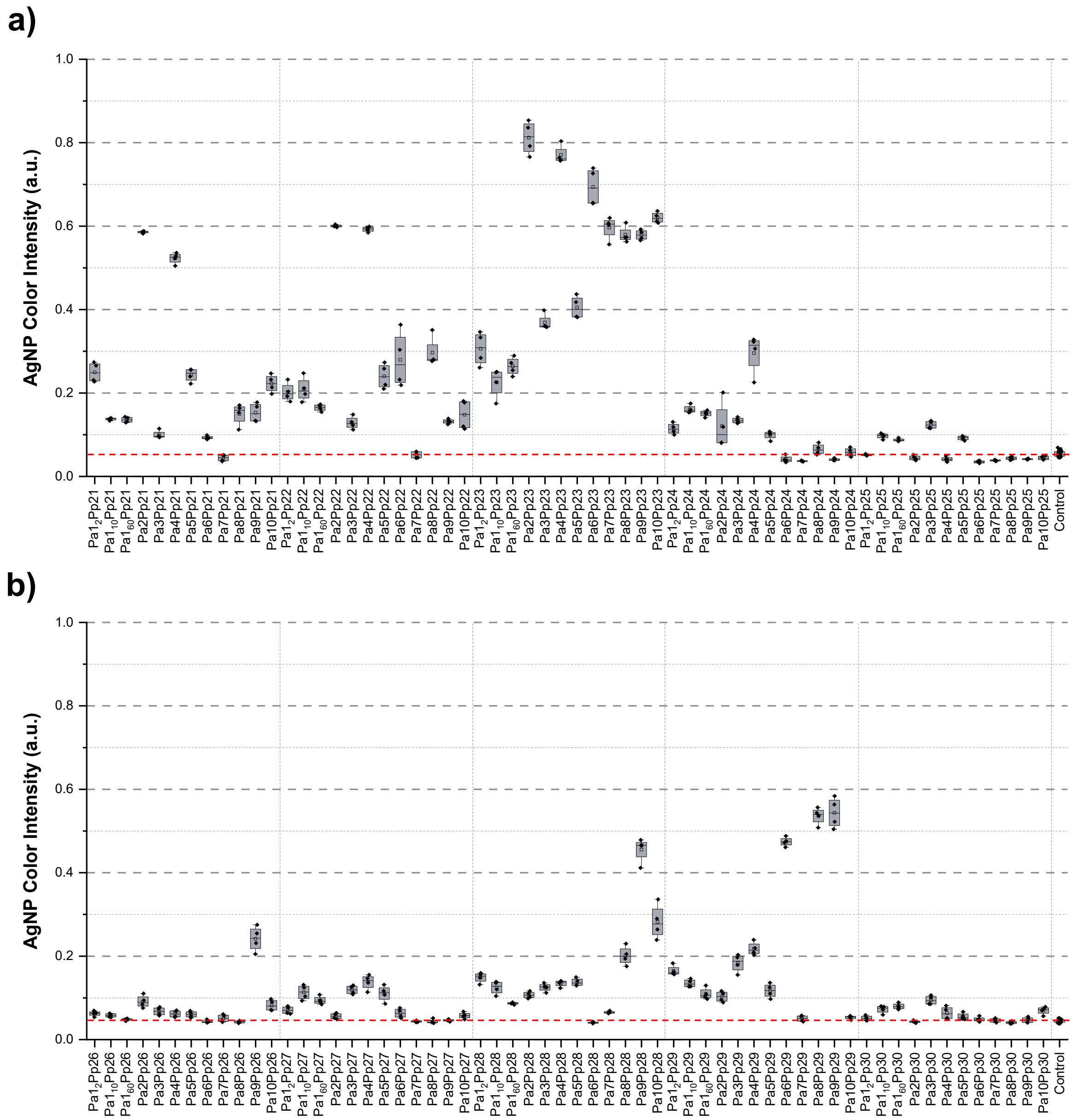

Figure S39. UHT screening of silver-reducing activity. Shown are the color intensities of AgNPs formed on **PaPp** coatings prepared from **Pa1$_2$-Pa10** crosslinked with a) **Pp21-Pp25** and b) **Pp26-Pp30**. Uncoated spots served as controls.

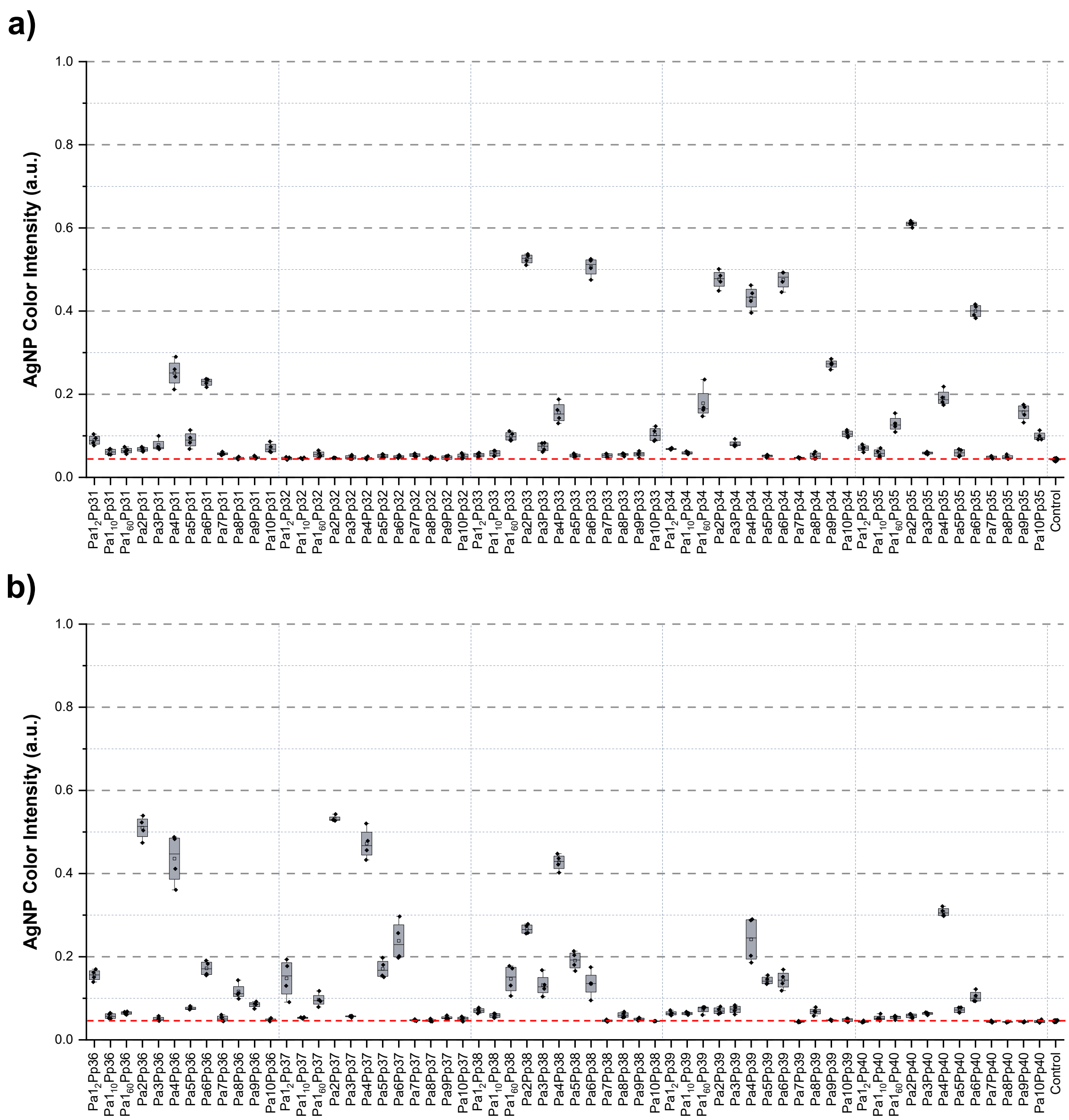

Figure S40. UHT screening of silver-reducing activity. Shown are the color intensities of AgNPs formed on **PaPp** coatings prepared from **Pa1$_2$-Pa10** crosslinked with a) **Pp31-Pp35** and b) **Pp36-Pp40**. Uncoated spots served as controls.

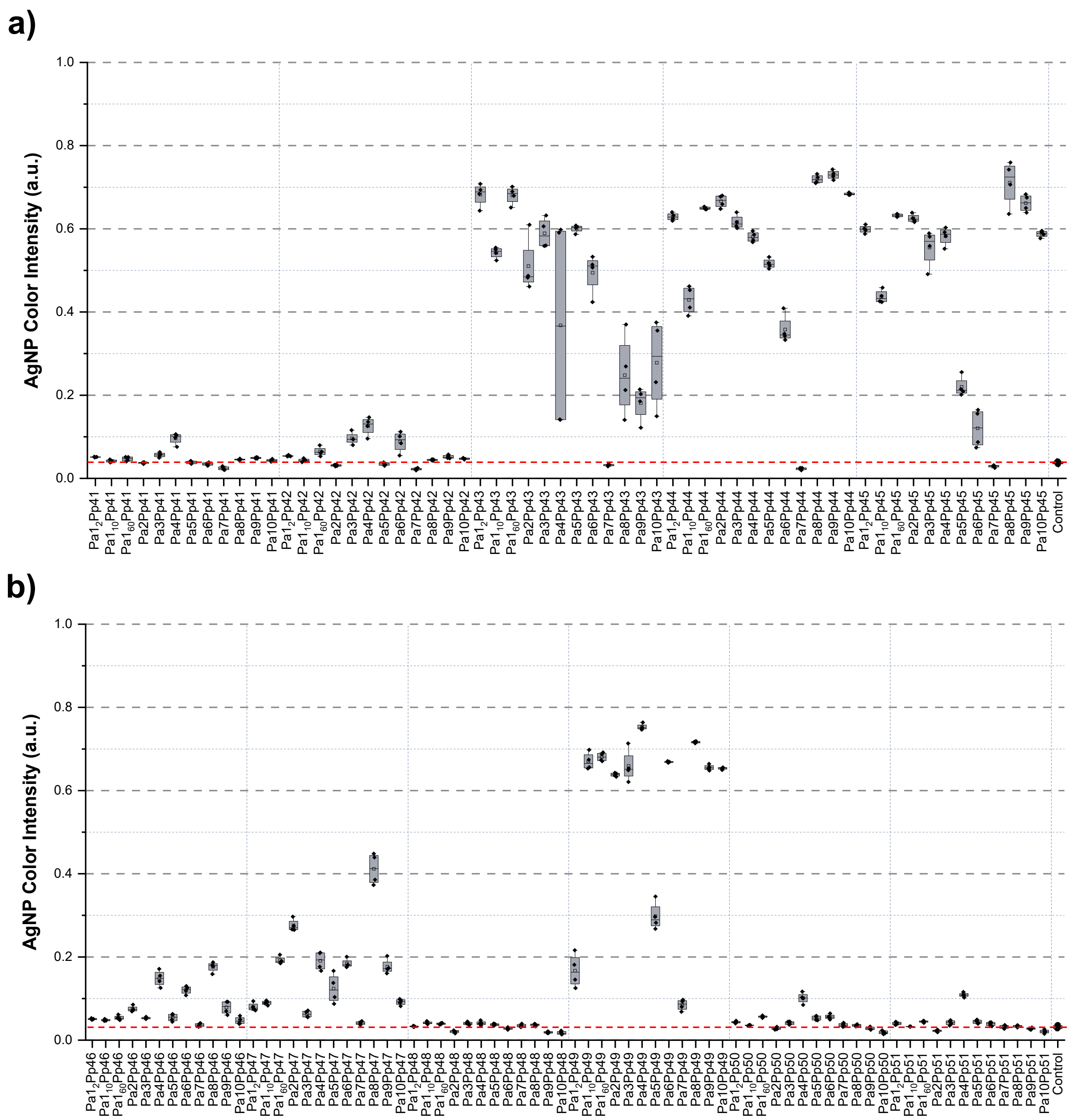

Figure S41. UHT screening of silver-reducing activity. Shown are the color intensities of AgNPs formed on **PaPp** coatings prepared from **Pa1$_2$-Pa10** crosslinked with a) **Pp41-Pp45** and b) **Pp46-Pp51**. Uncoated spots served as controls.

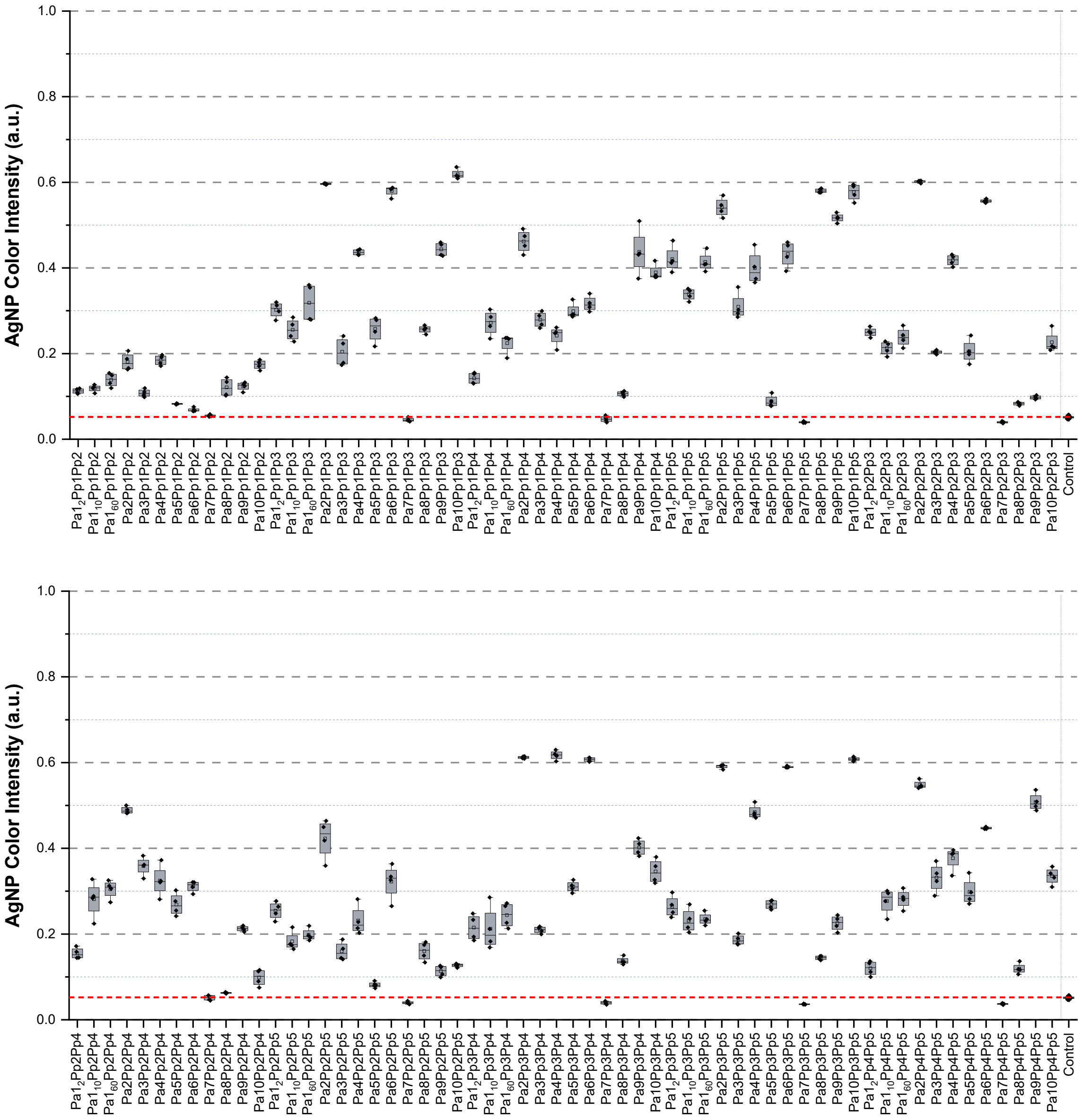

Figure S42. UHT screening of silver-reducing activity. Shown are the color intensities of AgNPs formed on **PaPp** coatings prepared from **Pa1$_2$-Pa10** crosslinked with binary precursor combinations (**PpPp**) derived from **Pp1-Pp5**, respectively. Uncoated spots served as controls.

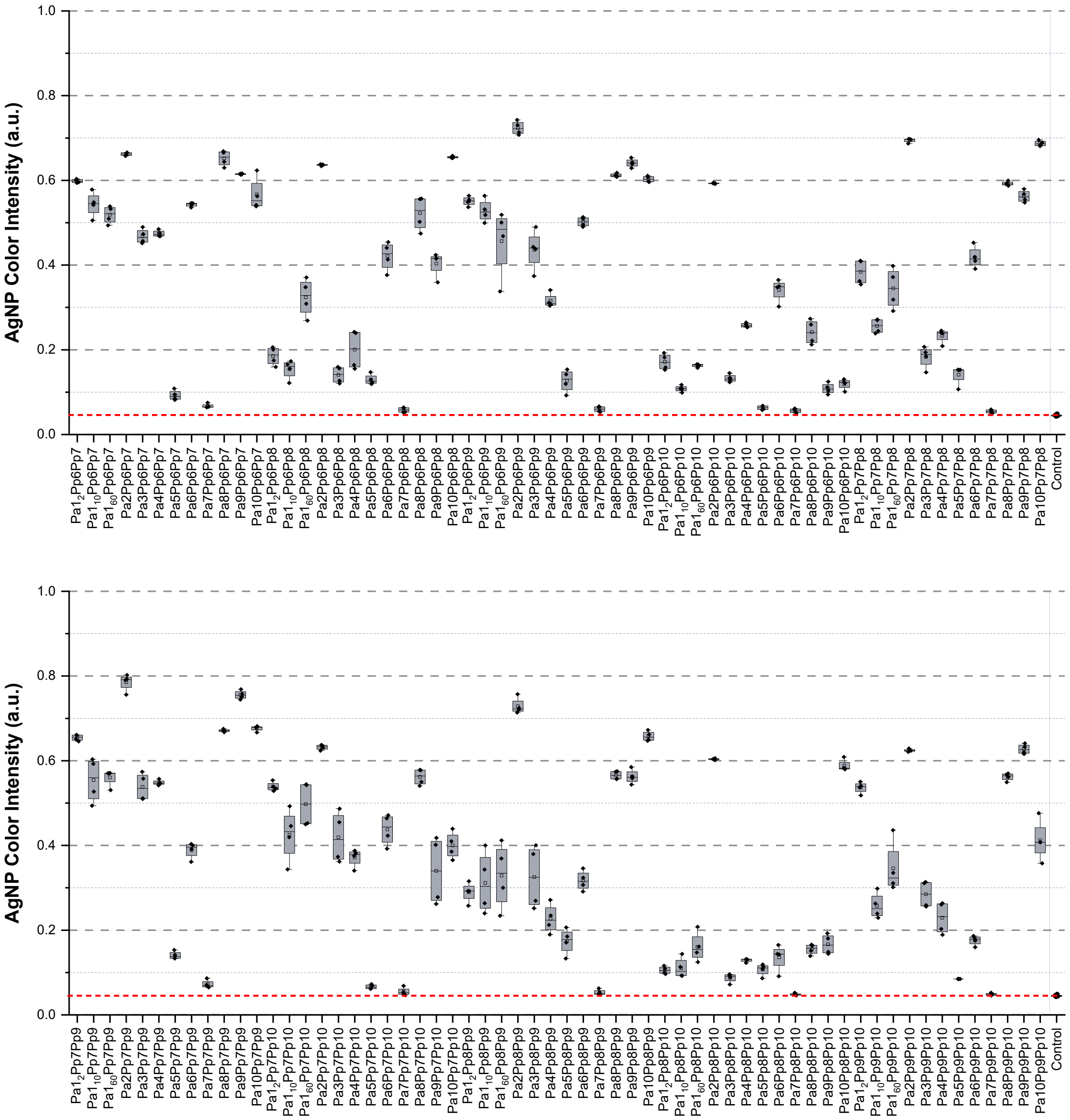

Figure S43. UHT screening of silver-reducing activity. Shown are the color intensities of AgNPs formed on **PaPp** coatings prepared from **Pa1$_2$-Pa10** crosslinked with binary precursor combinations (**PpPp**) derived from **Pp6-Pp10**, respectively. Uncoated spots served as controls.

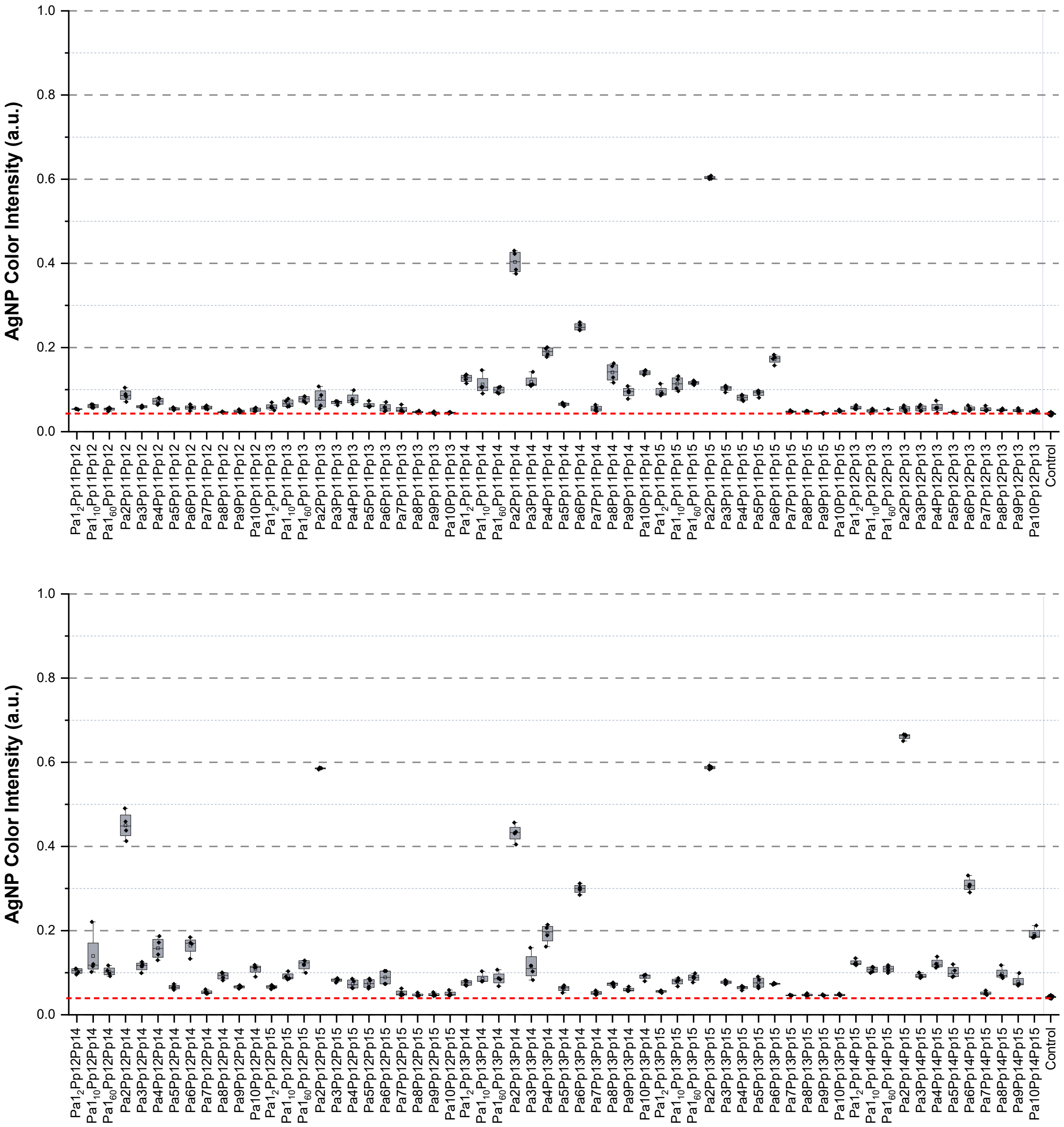

Figure S44. UHT screening of silver-reducing activity. Shown are the color intensities of AgNPs formed on **PaPp** coatings prepared from **Pa1₂-Pa10** crosslinked with binary precursor combinations (**PpPp**) derived from **Pp11-Pp15**, respectively. Uncoated spots served as controls.

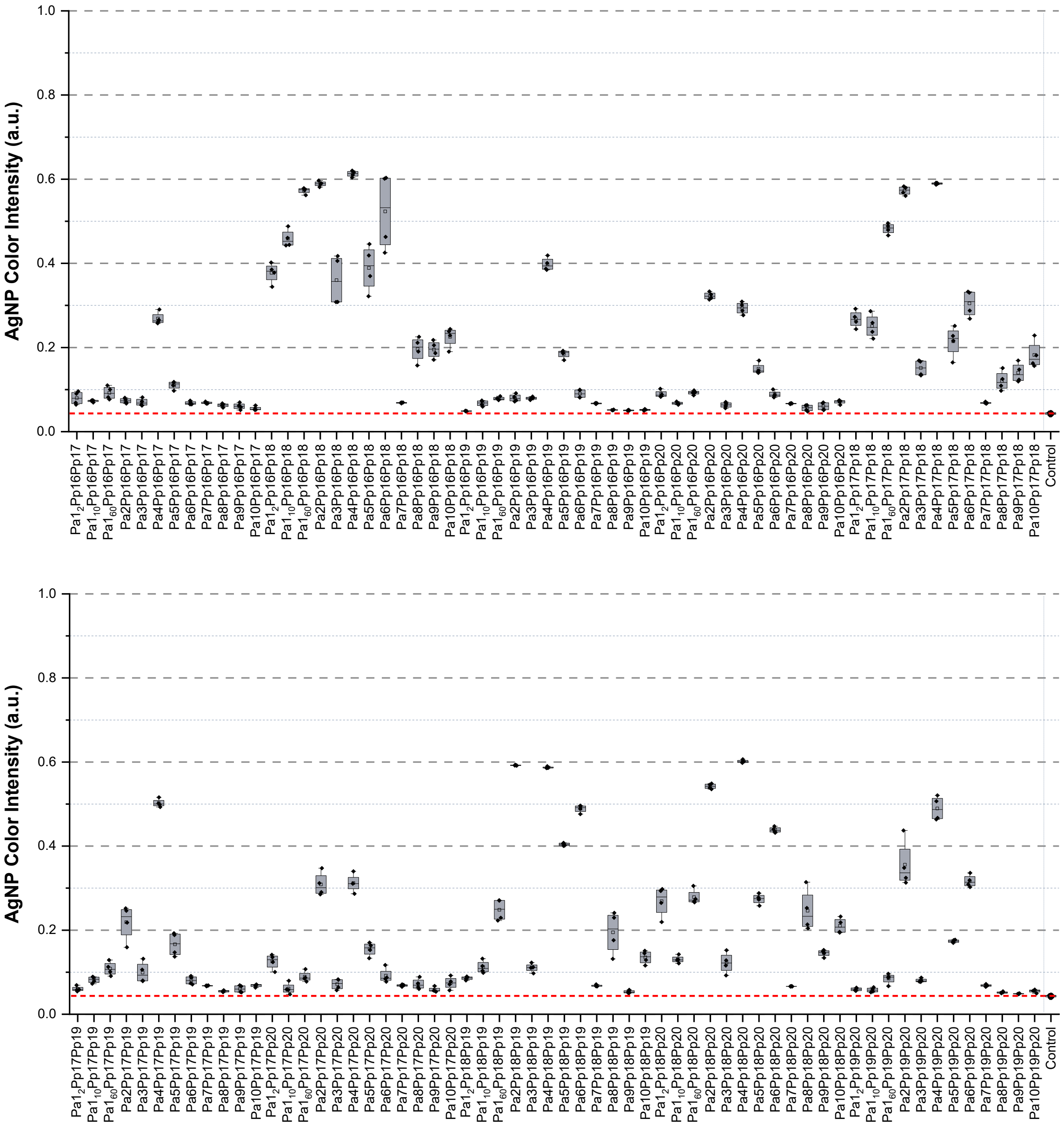

Figure S45. UHT screening of silver-reducing activity. Shown are the color intensities of AgNPs formed on **PaPp** coatings prepared from **Pa1$_2$-Pa10** crosslinked with binary precursor combinations (**PpPp**) derived from **Pp16-Pp20**, respectively. Uncoated spots served as controls.

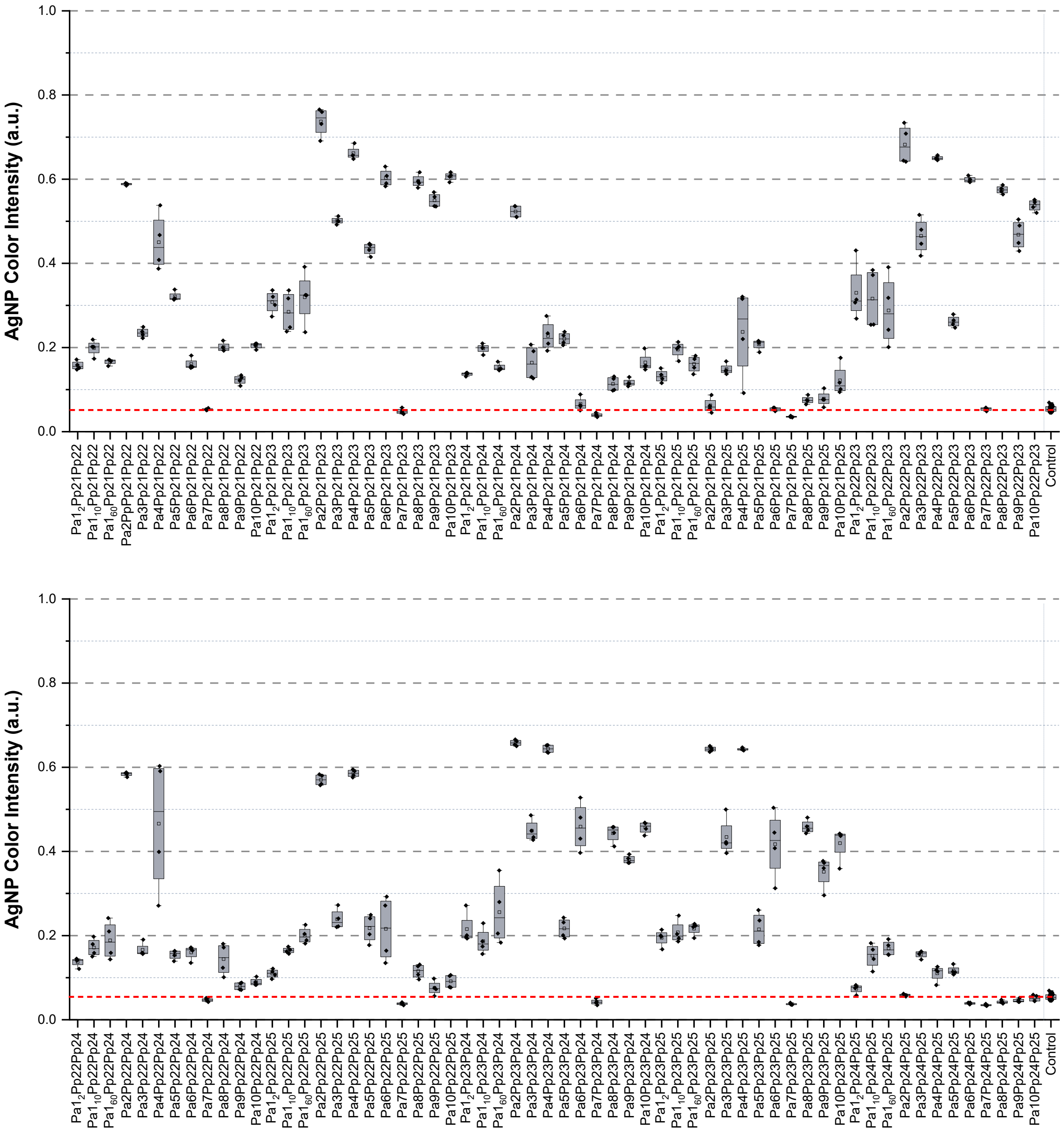

Figure S46. UHT screening of silver-reducing activity. Shown are the color intensities of AgNPs formed on **PaPp** coatings prepared from **Pa1$_2$-Pa10** crosslinked with binary precursor combinations (**PpPp**) derived from **Pp21-Pp25**, respectively. Uncoated spots served as controls.

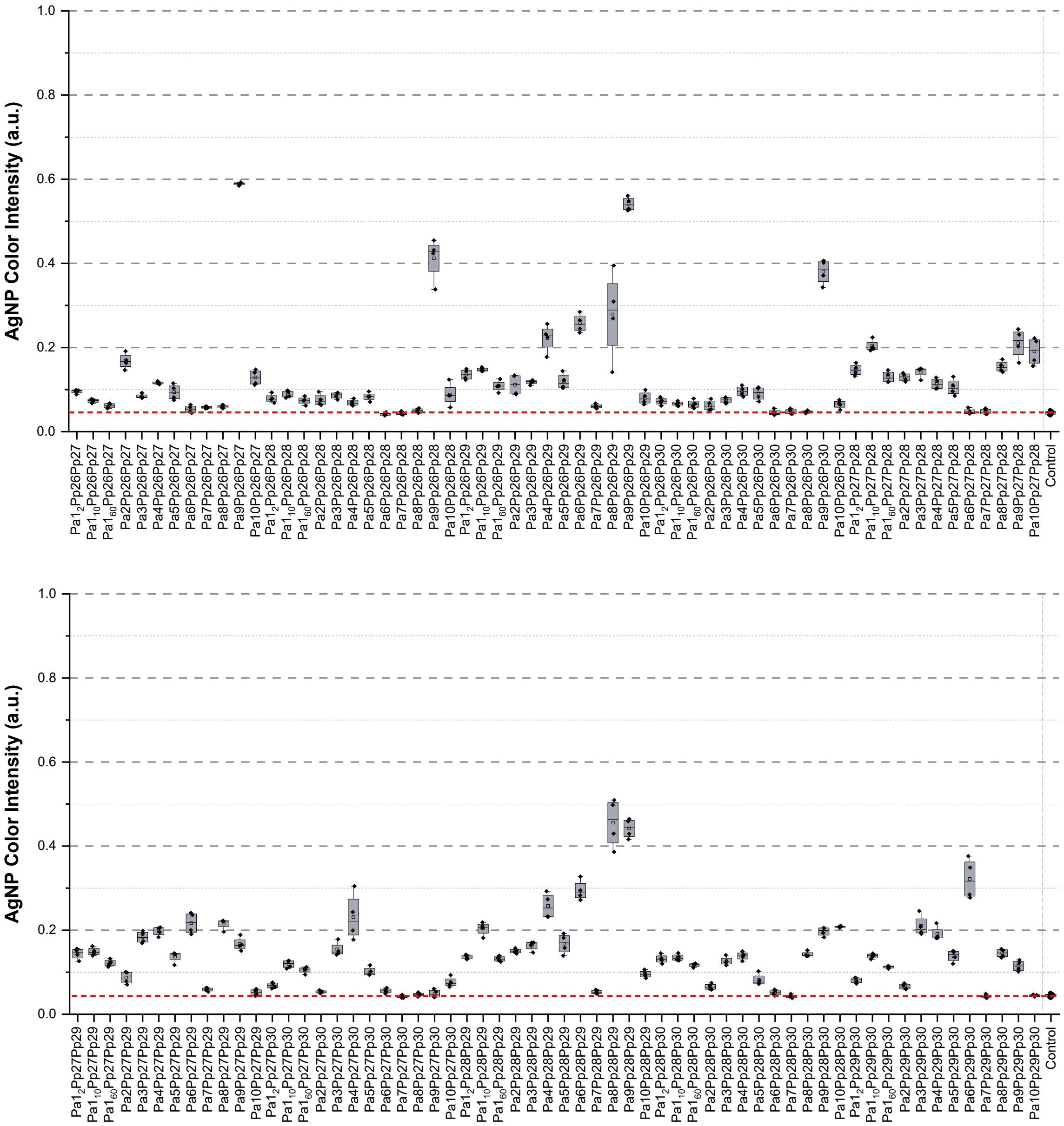

Figure S47. UHT screening of silver-reducing activity. Shown are the color intensities of AgNPs formed on **PaPp** coatings prepared from **Pa1$_2$-Pa10** crosslinked with binary precursor combinations (**PpPp**) derived from **Pp26-Pp30**, respectively. Uncoated spots served as controls.

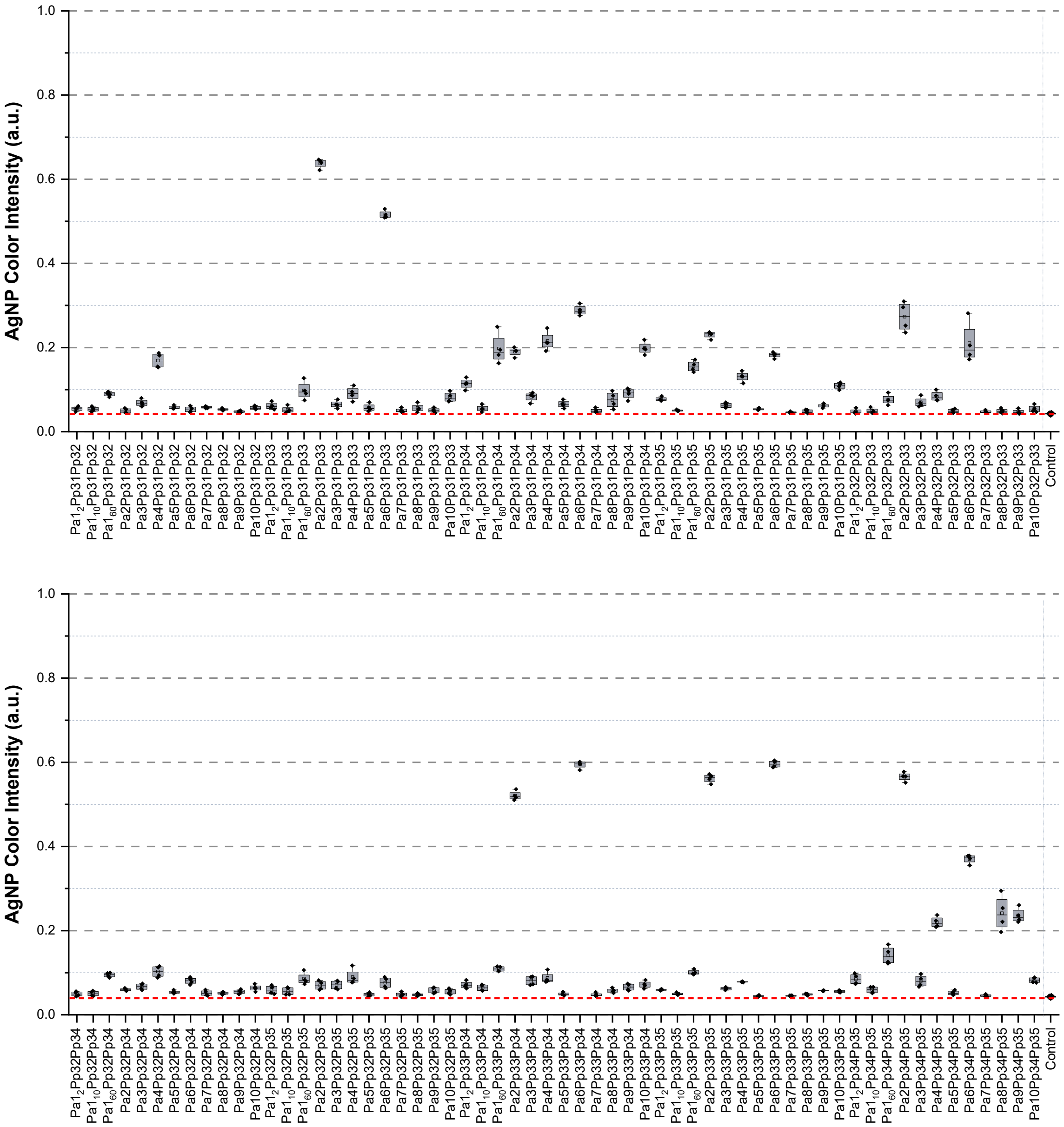

Figure S48. UHT screening of silver-reducing activity. Shown are the color intensities of AgNPs formed on **PaPp** coatings prepared from **Pa1$_2$-Pa10** crosslinked with binary precursor combinations (**PpPp**) derived from **Pp31-Pp35**, respectively. Uncoated spots served as controls.

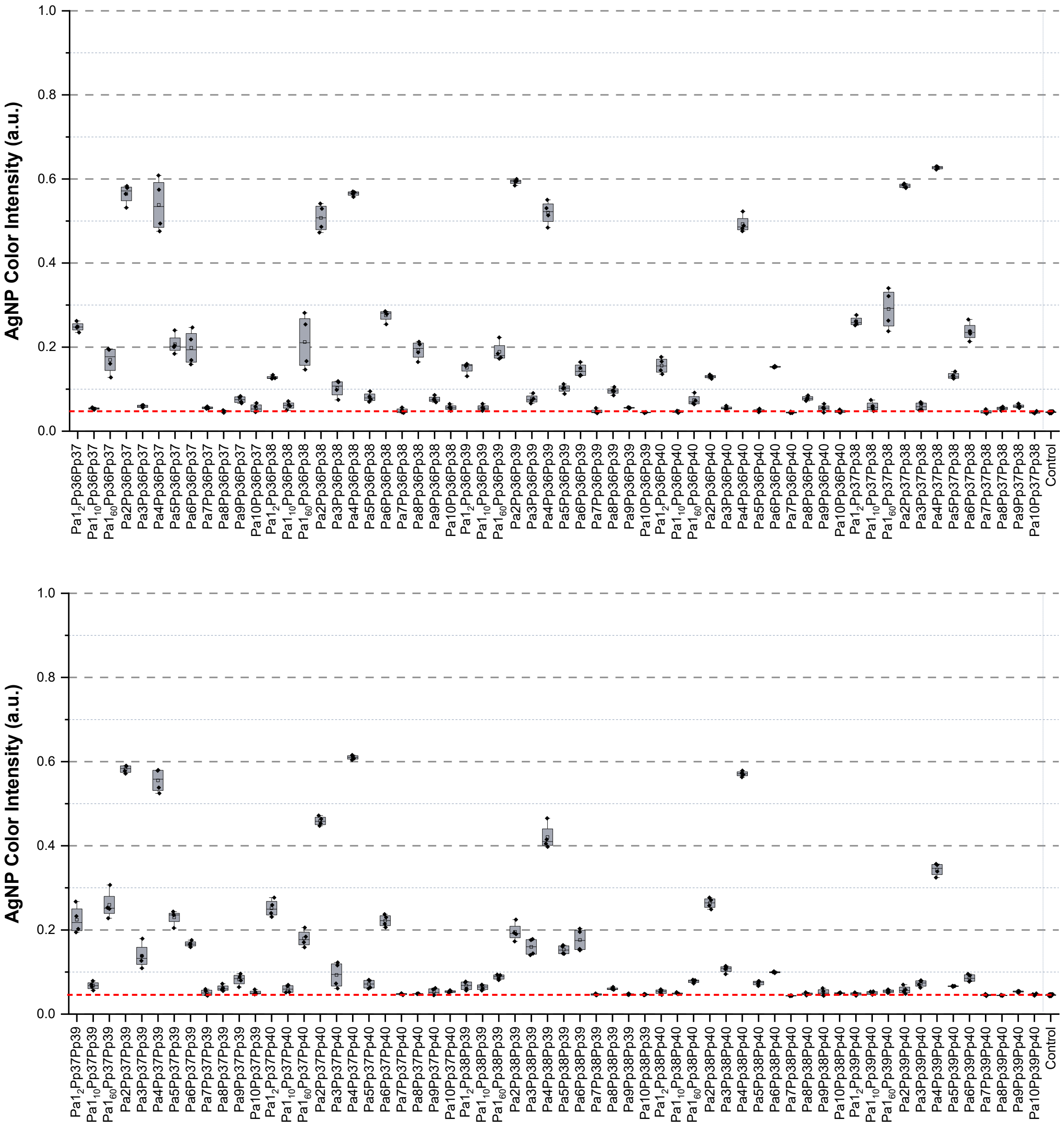

Figure S49. UHT screening of silver-reducing activity. Shown are the color intensities of AgNPs formed on **PaPp** coatings prepared from **Pa1$_2$-Pa10** crosslinked with binary precursor combinations (**PpPp**) derived from **Pp36-Pp40**, respectively. Uncoated spots served as controls.

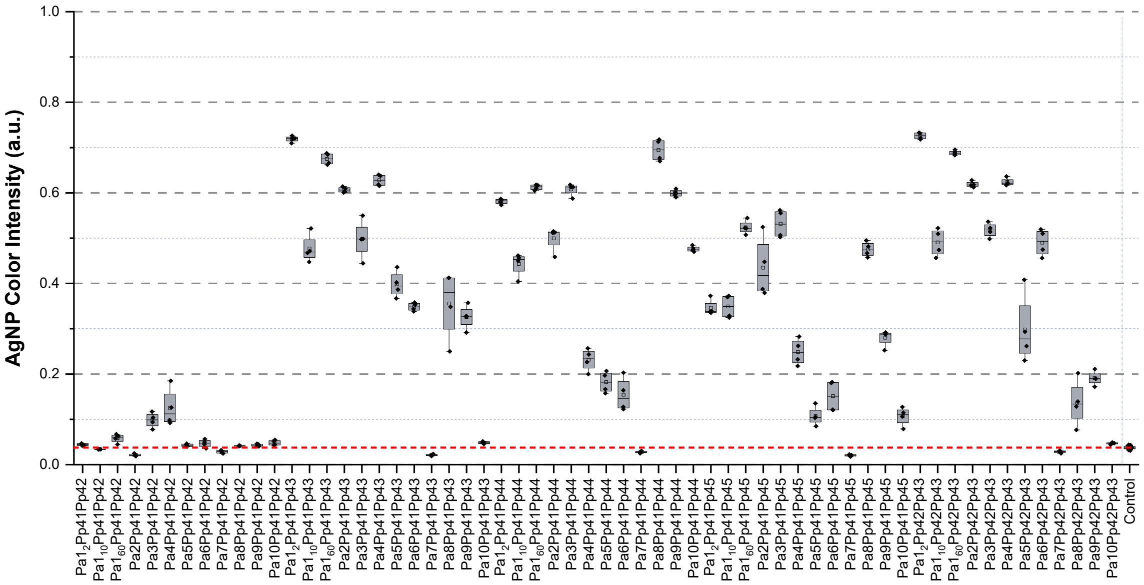

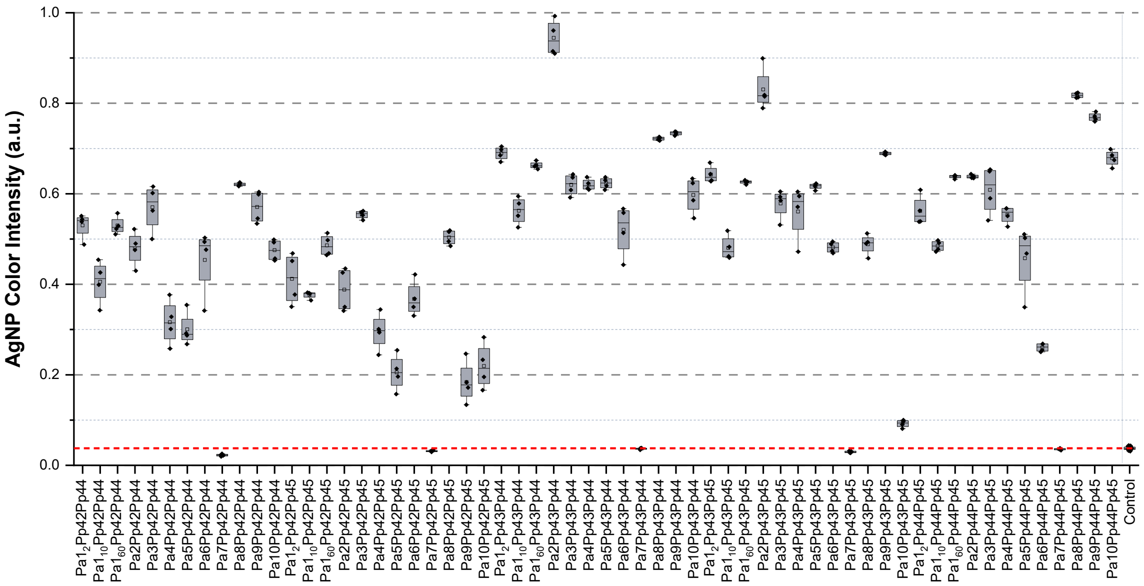

Figure S50. UHT screening of silver-reducing activity. Shown are the color intensities of AgNPs formed on **PaPp** coatings prepared from **Pa1$_2$-Pa10** crosslinked with binary precursor combinations (**PpPp**) derived from **Pp41-Pp45**, respectively. Uncoated spots served as controls.

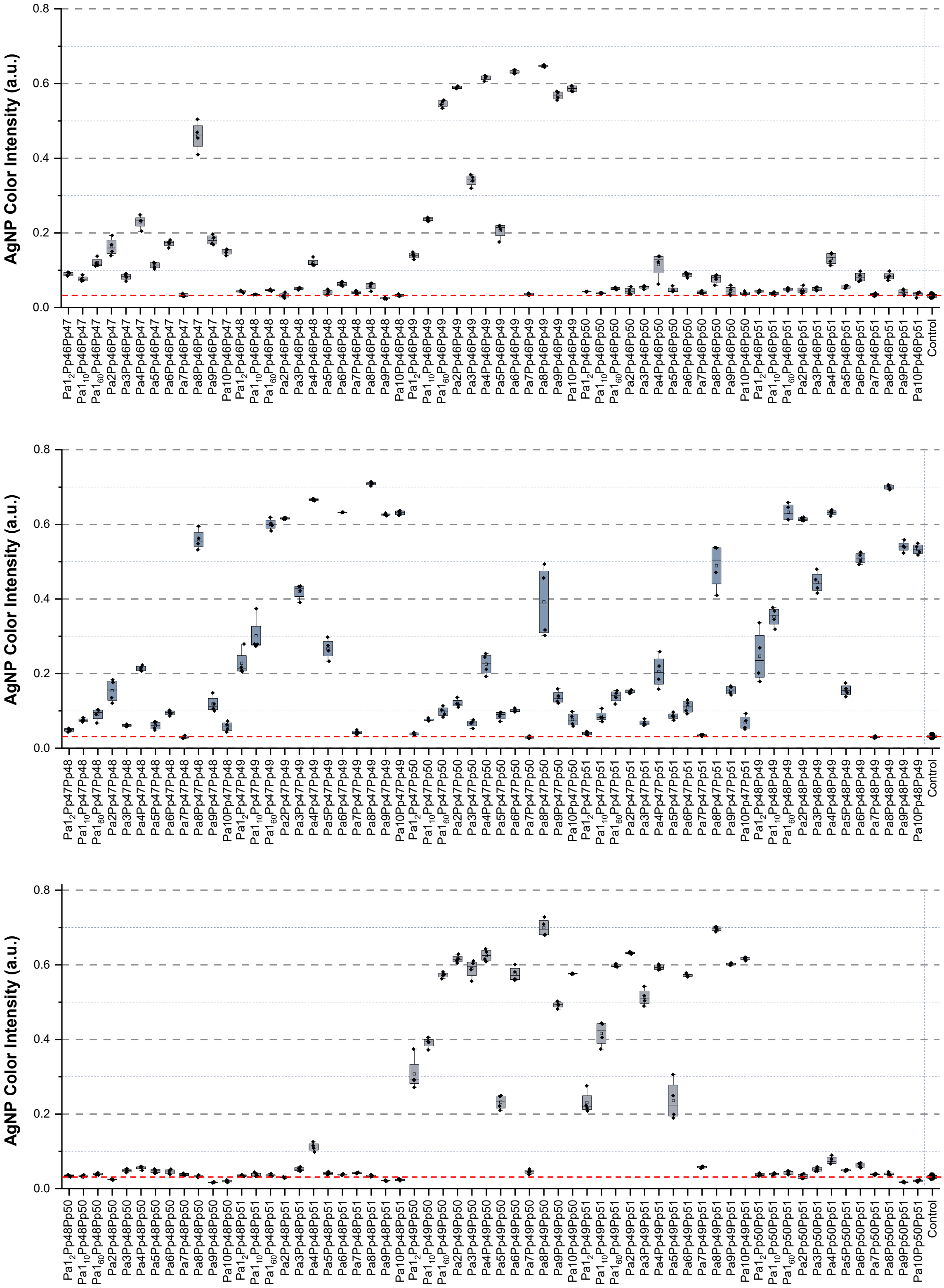

Figure S51. UHT screening of silver-reducing activity. Shown are the color intensities of AgNPs formed on **PaPp** coatings prepared from **Pa1$_2$-Pa10** crosslinked with binary precursor combinations (**PpPp**) derived from **Pp46-Pp51**, respectively. Uncoated spots served as controls.

6. UHT Screening of Antibacterial Activity

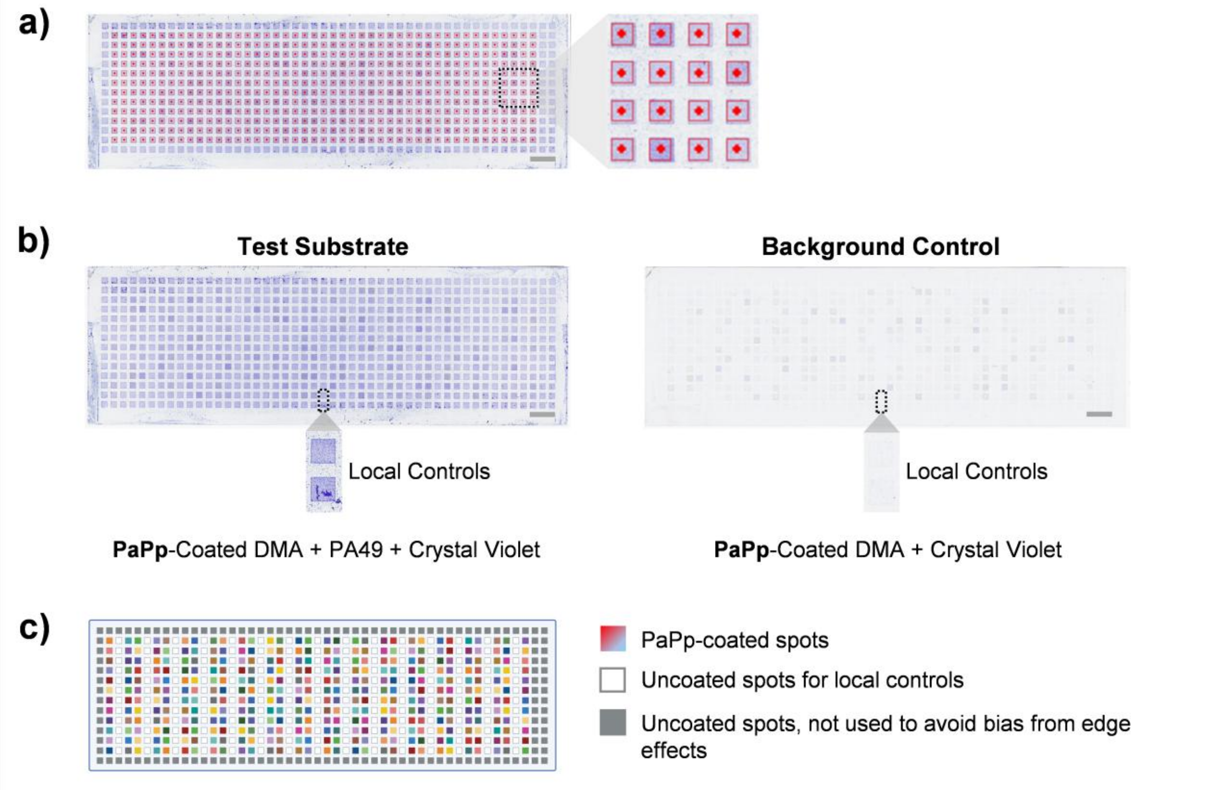

Figure S52. a) Grid Screener detection (red squares) of DMA bearing 1 mm × 1 mm spots. b) Comparison between the test substrate (**PaPp**-coated DMA immersed in *P. aeruginosa* PA49 bacterial solution and stained with crystal violet) and the background control (**PaPp**-coated DMA stained with crystal violet). c) Printing layout of **PaPp** for UHT screening of antibacterial coatings. Spot size: 1 mm × 1 mm. Scale bar: 4 mm.

a)

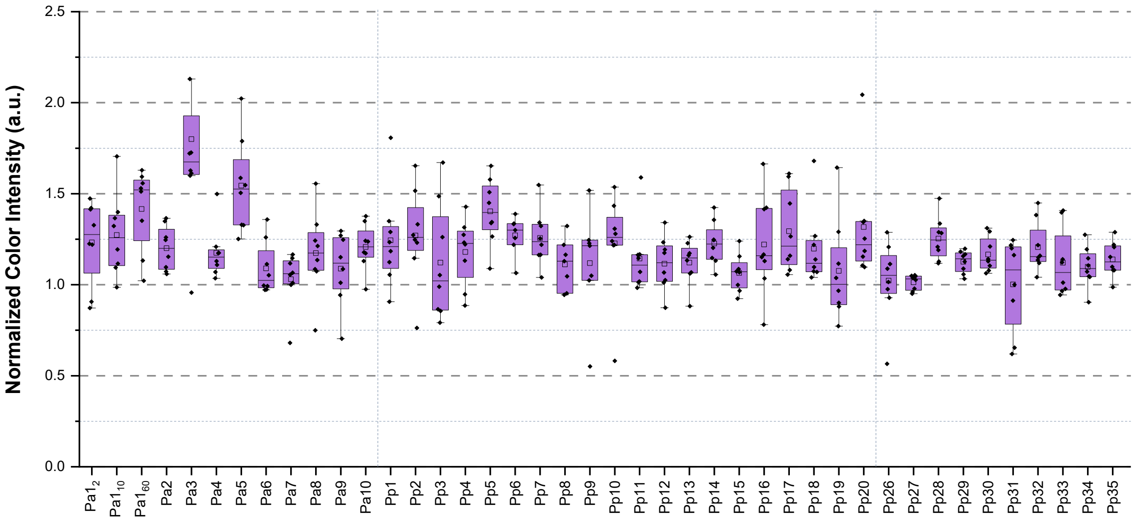

b)

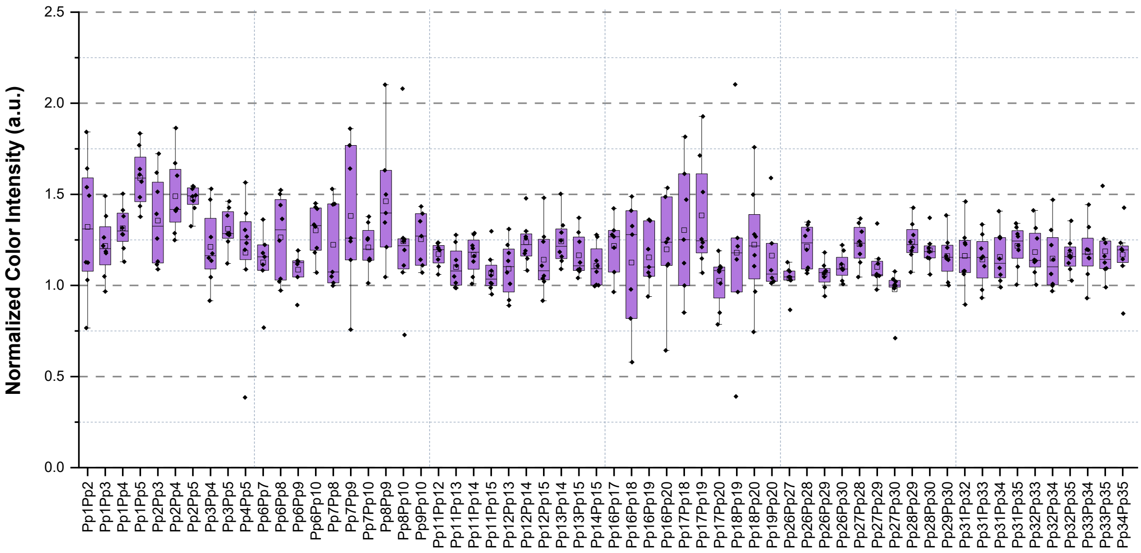

Figure S53. UHT screening of antibacterial activity against *P. aeruginosa* PA49. Normalized crystal violet color intensities corresponding to surface-adhered bacterial biomass on a) individual precursors **Pa1$_2$-Pa10**, **Pp1-Pp20**, and **Pp26-Pp35**, and b) binary combinations (**PpPp**) derived from **Pp1-Pp20** and **Pp26-Pp35**. Lower values indicate higher antibacterial activity.

a)

b)

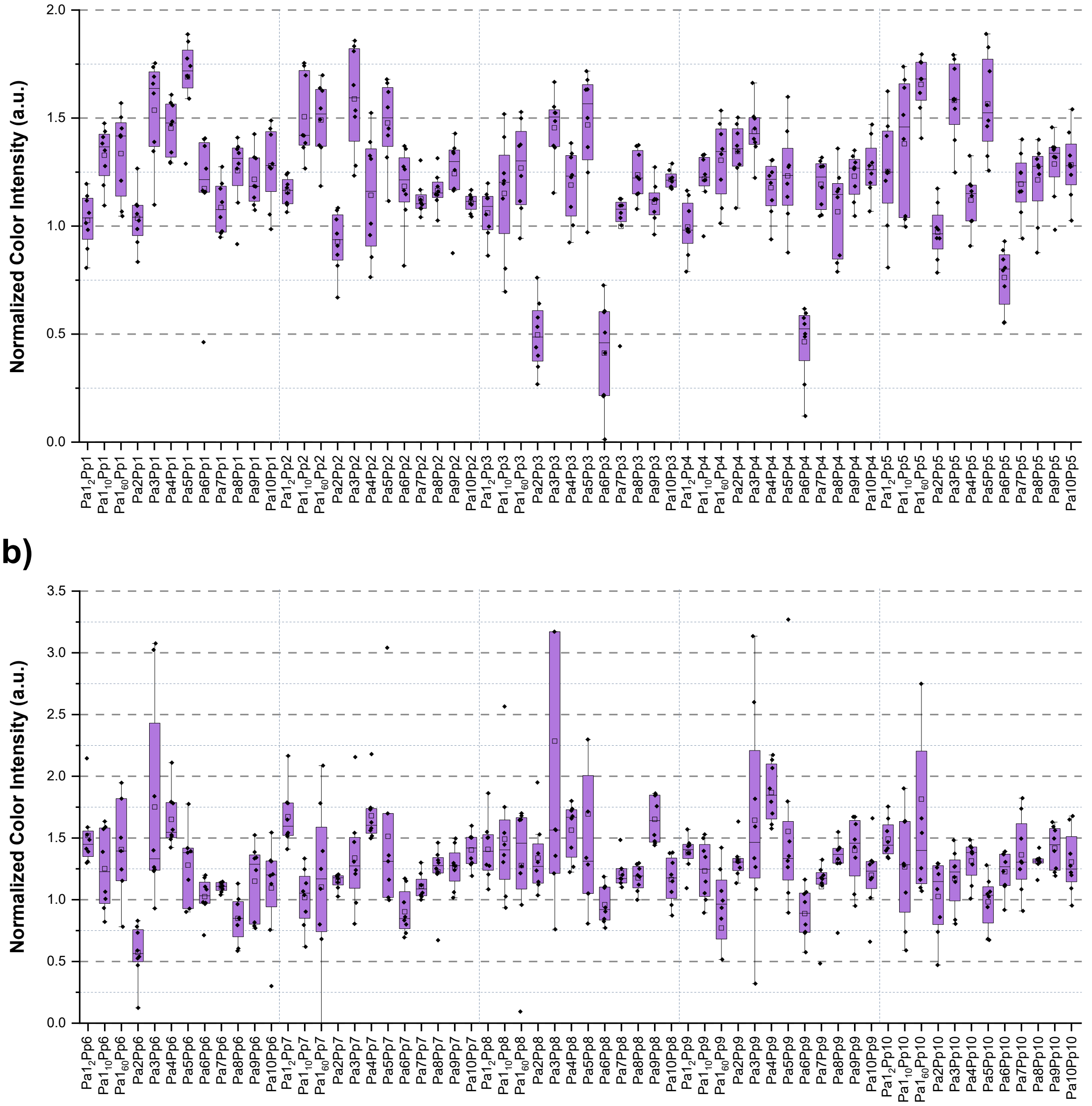

Figure S54. UHT screening of antibacterial activity against *P. aeruginosa* PA49. Normalized crystal violet color intensities corresponding to surface-adhered bacterial biomass on **PaPp** coatings prepared from **Pa1$_2$-Pa10** crosslinked with a) **Pp1-Pp5** and b) **Pp6-Pp10**. Lower values indicate higher antibacterial activity.

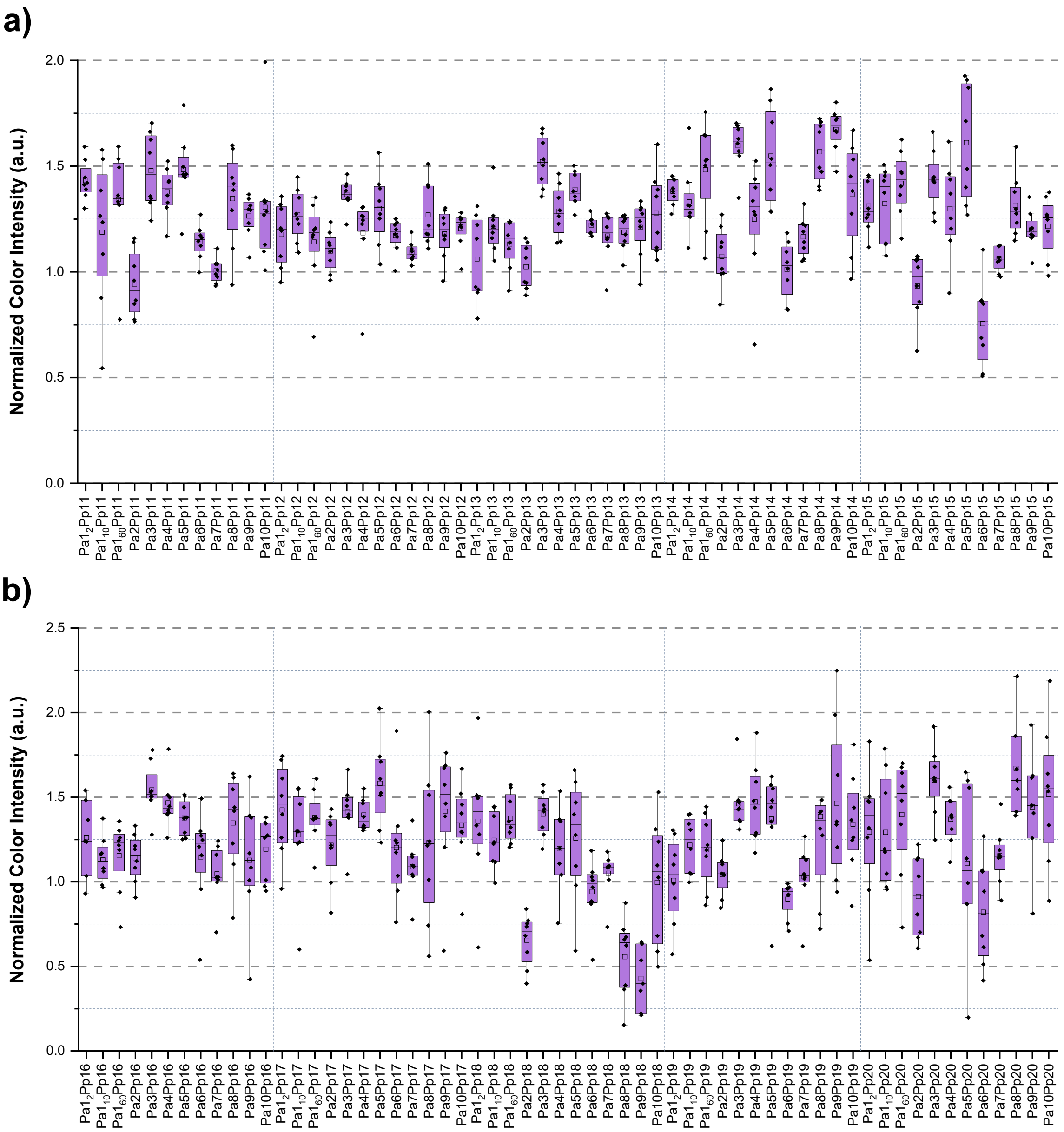

Figure S55. UHT screening of antibacterial activity against *P. aeruginosa* PA49. Normalized crystal violet color intensities corresponding to surface-adhered bacterial biomass on **PaPp** coatings prepared from **Pa1$_2$-Pa10** crosslinked with a) **Pp11-Pp15** and b) **Pp16-Pp20**. Lower values indicate higher antibacterial activity.

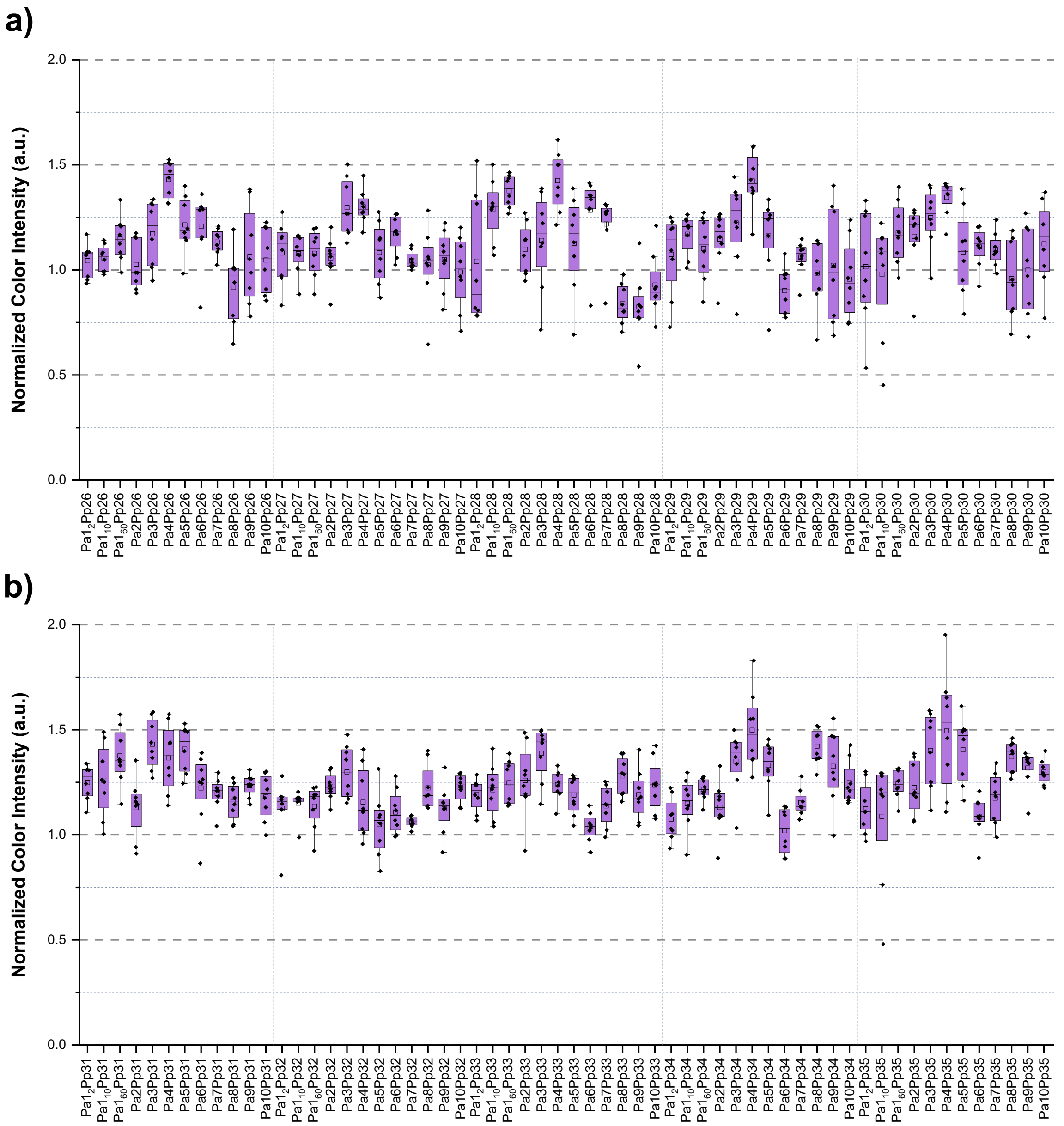

Figure S56. UHT screening of antibacterial activity against *P. aeruginosa* PA49. Normalized crystal violet color intensities corresponding to surface-adhered bacterial biomass on **PaPp** coatings prepared from **Pa1$_2$-Pa10** crosslinked with a) **Pp26-Pp30** and b) **Pp31-Pp35**. Lower values indicate higher antibacterial activity.

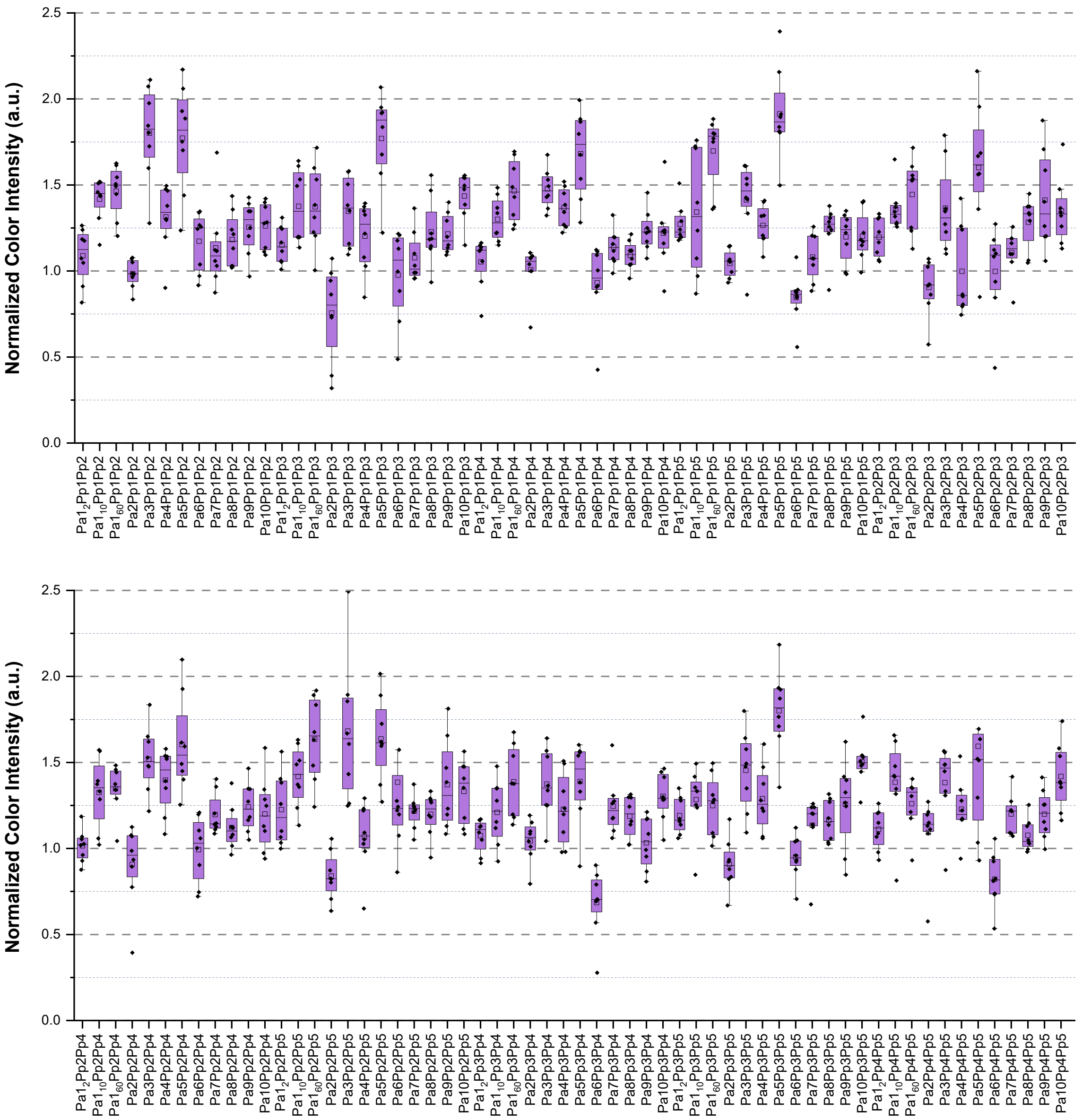

Figure S57. UHT screening of antibacterial activity against *P. aeruginosa* PA49. Normalized crystal violet color intensities corresponding to surface-adhered bacterial biomass on **PaPp** coatings prepared from **Pa1$_2$-Pa10** crosslinked with binary combinations (**PpPp**) derived from **Pp1-Pp5**, respectively. Lower values indicate higher antibacterial activity.

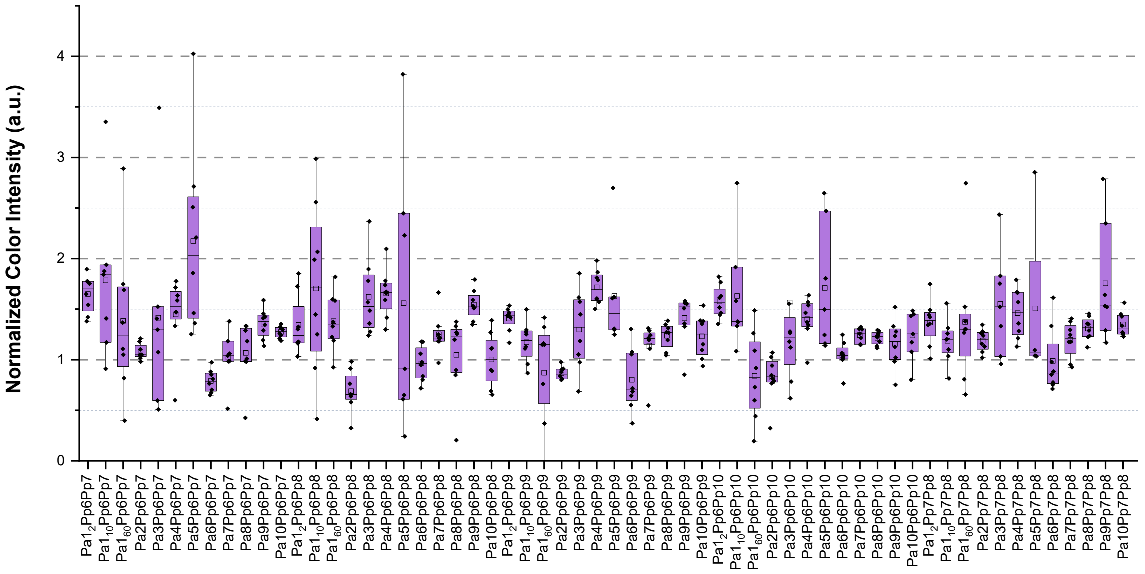

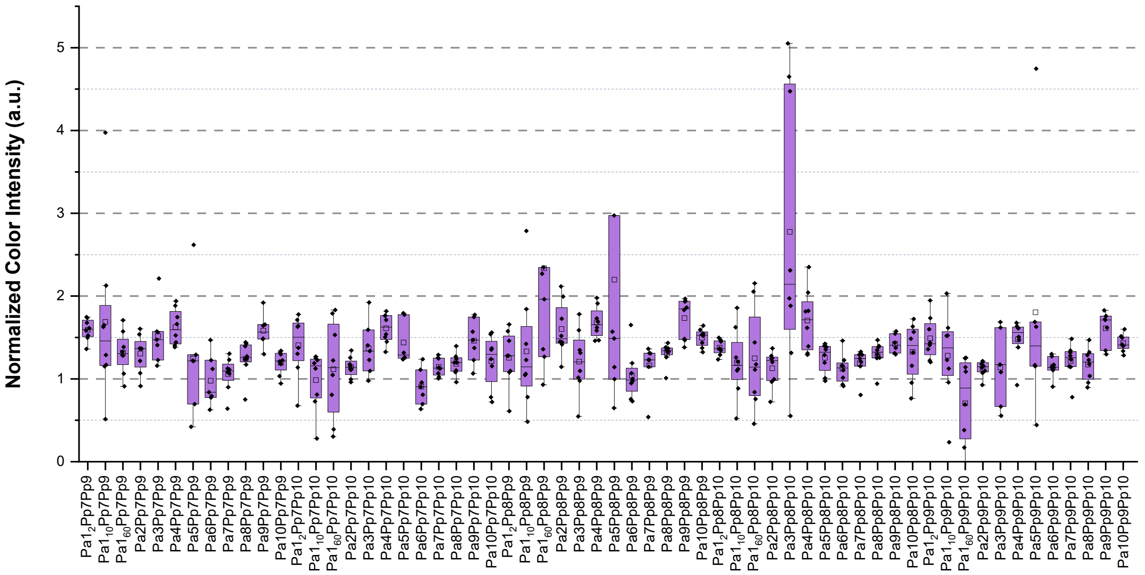

Figure S58. UHT screening of antibacterial activity against *P. aeruginosa* PA49. Normalized crystal violet color intensities corresponding to surface-adhered bacterial biomass on **PaPp** coatings prepared from **Pa1$_{2}$-Pa10** crosslinked with binary combinations (**PpPp**) derived from **Pp6-Pp10**, respectively. Lower values indicate higher antibacterial activity.

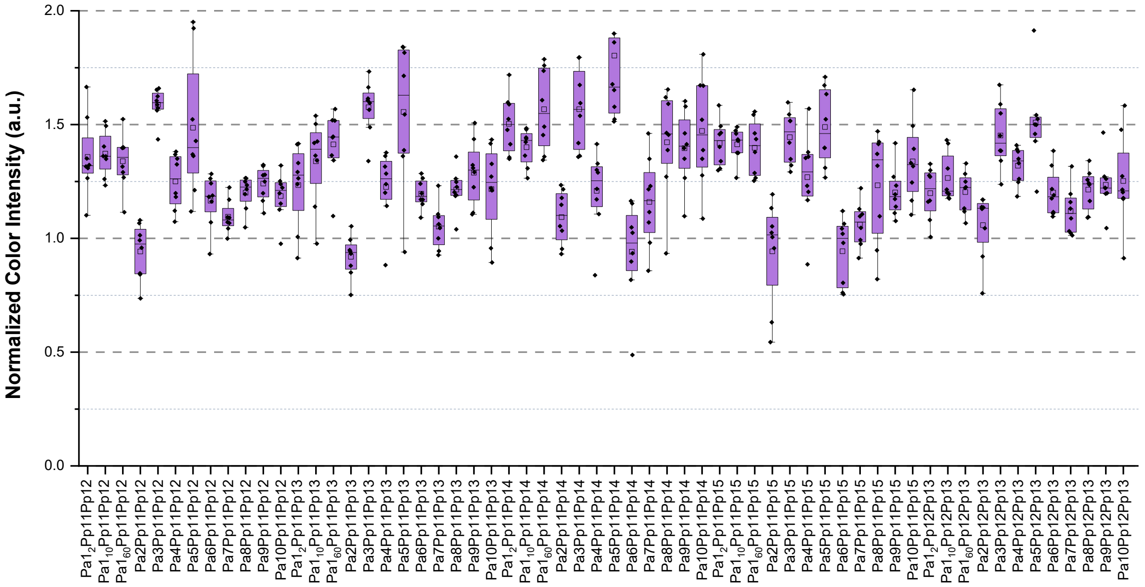

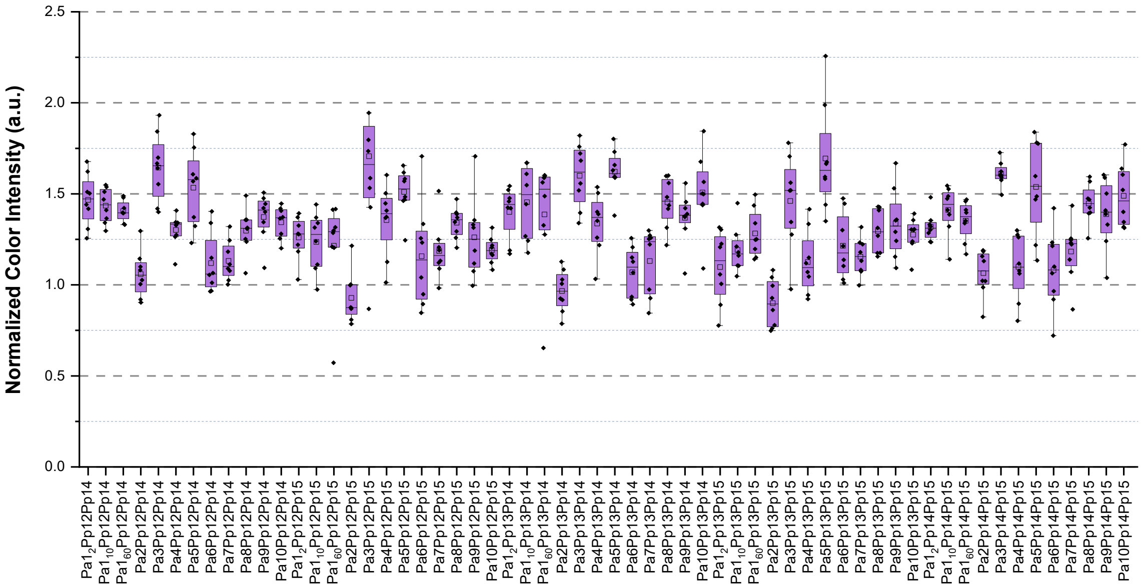

Figure S59. UHT screening of antibacterial activity against *P. aeruginosa* PA49. Normalized crystal violet color intensities corresponding to surface-adhered bacterial biomass on **PaPp** coatings prepared from **Pa1$_2$-Pa10** crosslinked with binary combinations (**PpPp**) derived from **Pp11-Pp15**, respectively. Lower values indicate higher antibacterial activity.

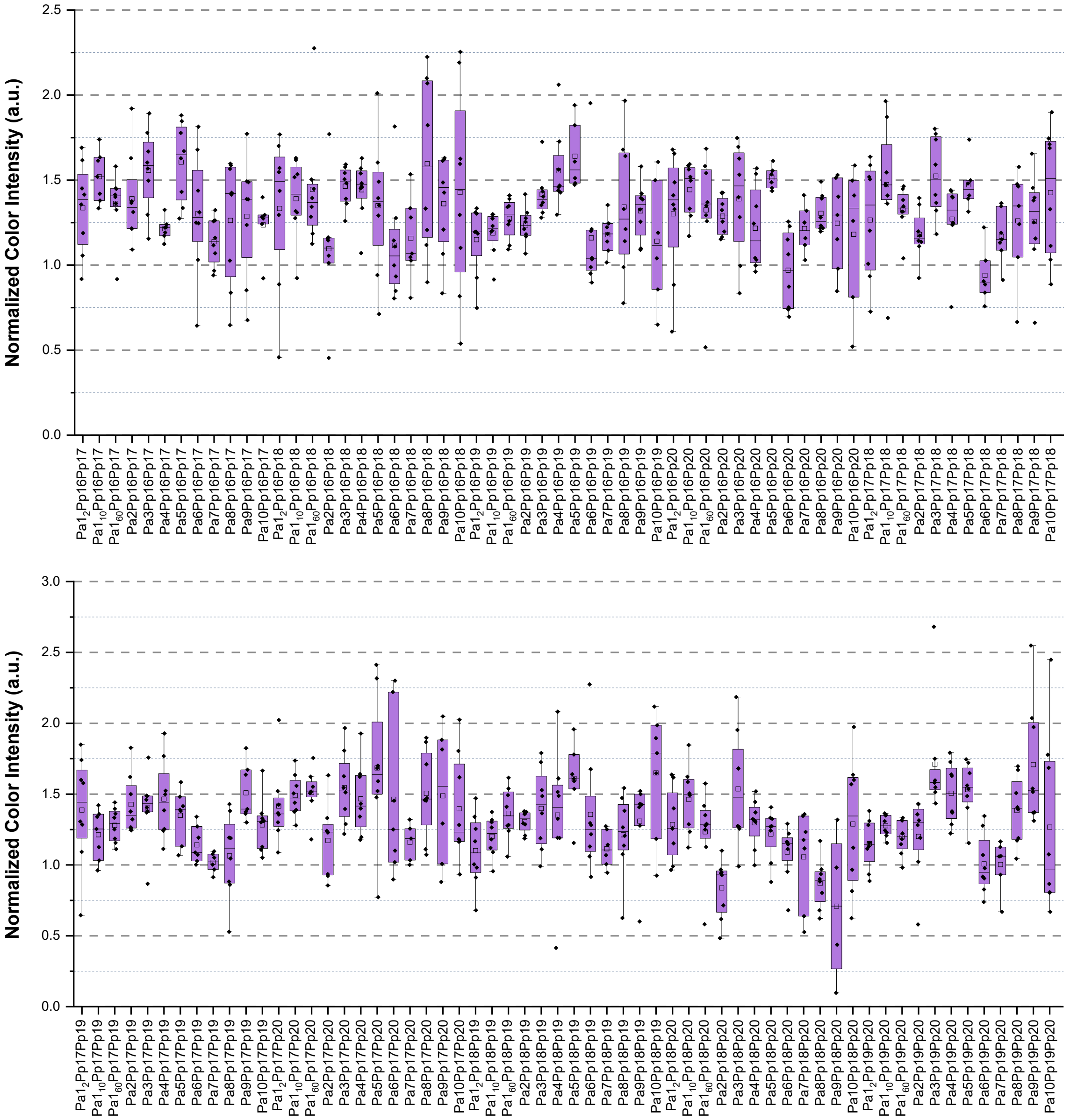

Figure S60. UHT screening of antibacterial activity against *P. aeruginosa* PA49. Normalized crystal violet color intensities corresponding to surface-adhered bacterial biomass on **PaPp** coatings prepared from **Pa1$_2$-Pa10** crosslinked with binary combinations (**PpPp**) derived from **Pp16-Pp20**, respectively. Lower values indicate higher antibacterial activity.

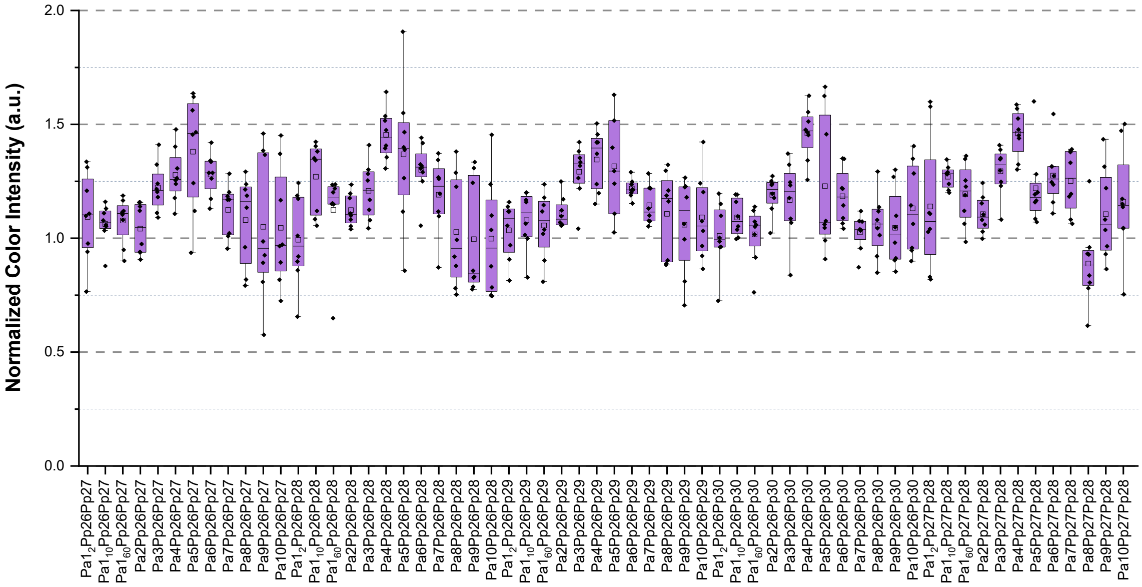

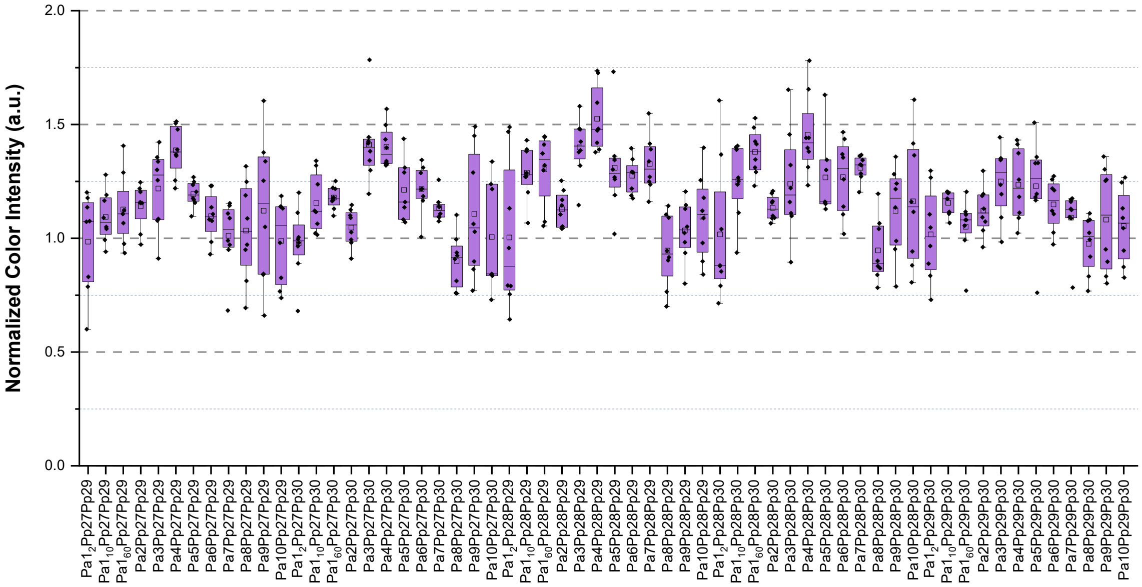

Figure S61. UHT screening of antibacterial activity against *P. aeruginosa* PA49. Normalized crystal violet color intensities corresponding to surface-adhered bacterial biomass on **PaPp** coatings prepared from **Pa1$_2$-Pa10** crosslinked with binary combinations (**PpPp**) derived from **Pp26-Pp30**, respectively. Lower values indicate higher antibacterial activity.

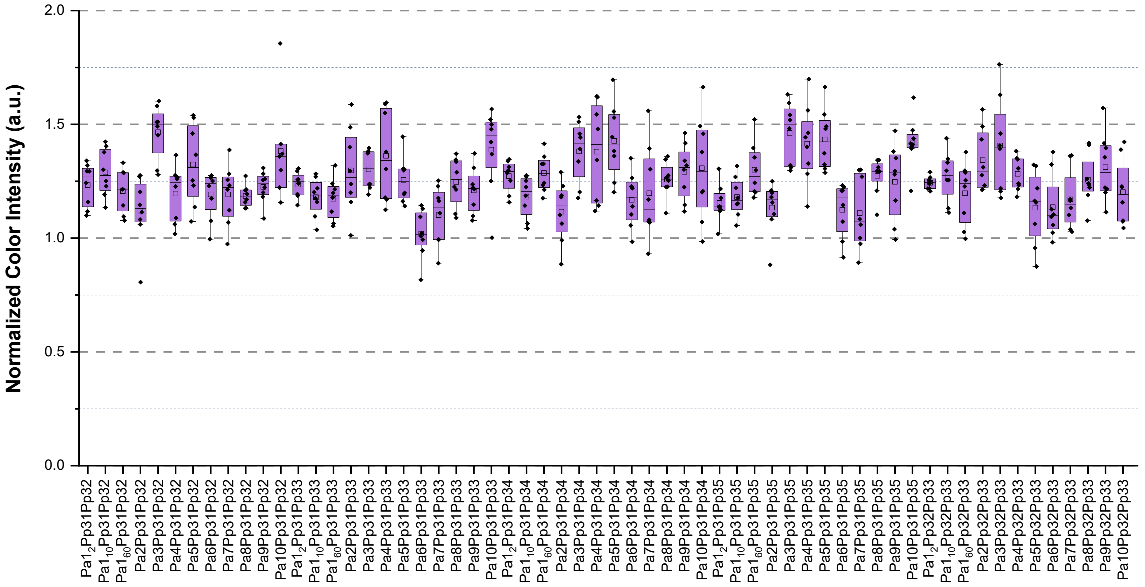

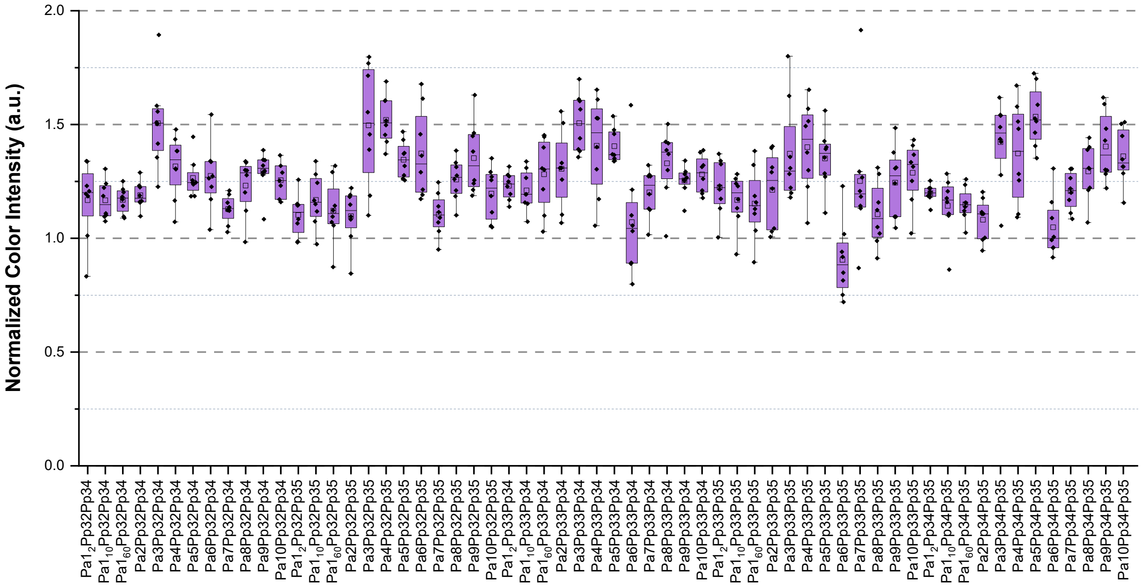

Figure S62. UHT screening of antibacterial activity against *P. aeruginosa* PA49. Normalized crystal violet color intensities corresponding to surface-adhered bacterial biomass on **PaPp** coatings prepared from **Pa1$_2$-Pa10** crosslinked with binary combinations (**PpPp**) derived from **Pp31-Pp35**, respectively. Lower values indicate higher antibacterial activity.

7. Validation of Antibacterial Hits

Table S15. Water contact angles (WCA, mean of three measurements) and surface roughness of **PaPp**-coated coverslips. Surface roughness was determined from scan areas of 160 µm × 160 µm and 1.6 mm × 1.6 mm.

Coatings	WCA (°)	Surface roughness (nm), 160 µm × 160 µm	Surface roughness (nm), 1.6 mm × 1.6 mm
Uncoated	62.5 ± 2.7	n.a	6.15
Pa2	71.9 ± 0.9	1.04	17.2
Pa6	60.8 ± 3.2	4.86	21.3
Pa5Pp3	80.4 ± 2.7	1.35	16.5
Pa2Pp3_1:1	88.2 ± 6.4	7.27	59.5
Pa2Pp3_1:3	81.6 ± 1.1	6.49	62.9
Pa2Pp3_1:5	88.8 ± 1.0	3.72	19.3
Pa2Pp3_3:1	68.4 ± 1.4	1.16	17.4
Pa2Pp3_5:1	65.9 ± 1.2	0.97	17.3
Pa2Pp6	78.2 ± 0.8	1.07	19.4
Pa6Pp3_1:1	82.3 ± 2.0	1.07	18.2
Pa6Pp3_1:3	75.8 ± 3.8	1.15	15.9
Pa6Pp3_1:5	87.5 ± 1.4	1.03	18.8
Pa6Pp3_3:1	68.0 ± 2.7	1.14	17
Pa6Pp3_5:1	65.0 ± 1.6	1.24	17.5
Pa6Pp4	92.4 ± 1.0	1.28	17.7
Pa8Pp18	69.0 ± 1.4	1.12	20.3
Pa9Pp18	63.4 ± 0.6	1.18	21

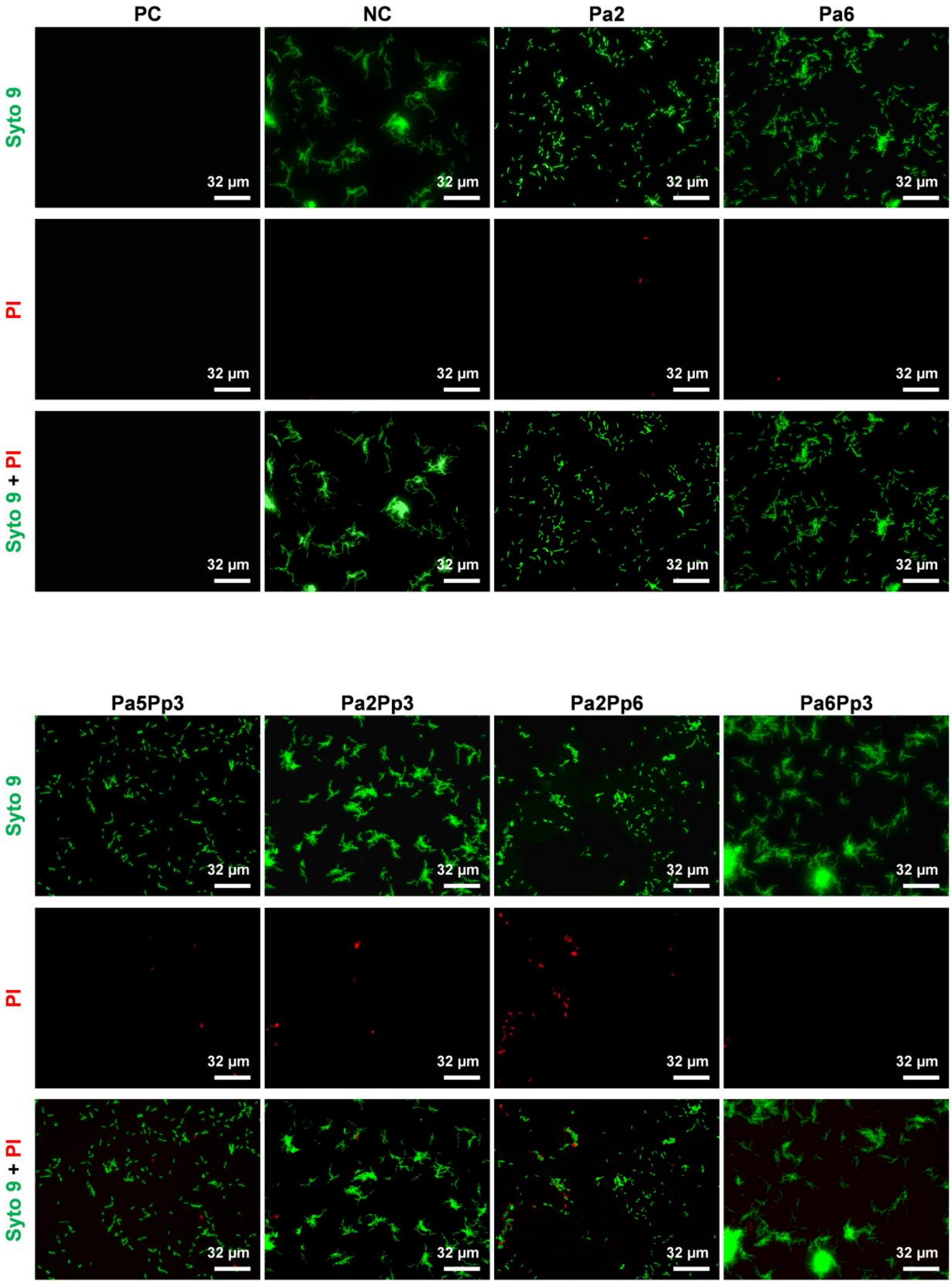

Figure S63. Live/dead staining images of *P. aeruginosa* PA49 on **PaPp**-coated coverslips after 4 h of incubation in bacterial suspension (MH medium). Syto 9 stains live bacteria, while propidium iodide (PI) stains dead bacteria. PC = positive control, uncoated coverslips incubated in MH medium. NC = negative control, uncoated coverslips incubated in PA49 suspension. Brightness, contrast, and saturation were adjusted for visualization purposes.

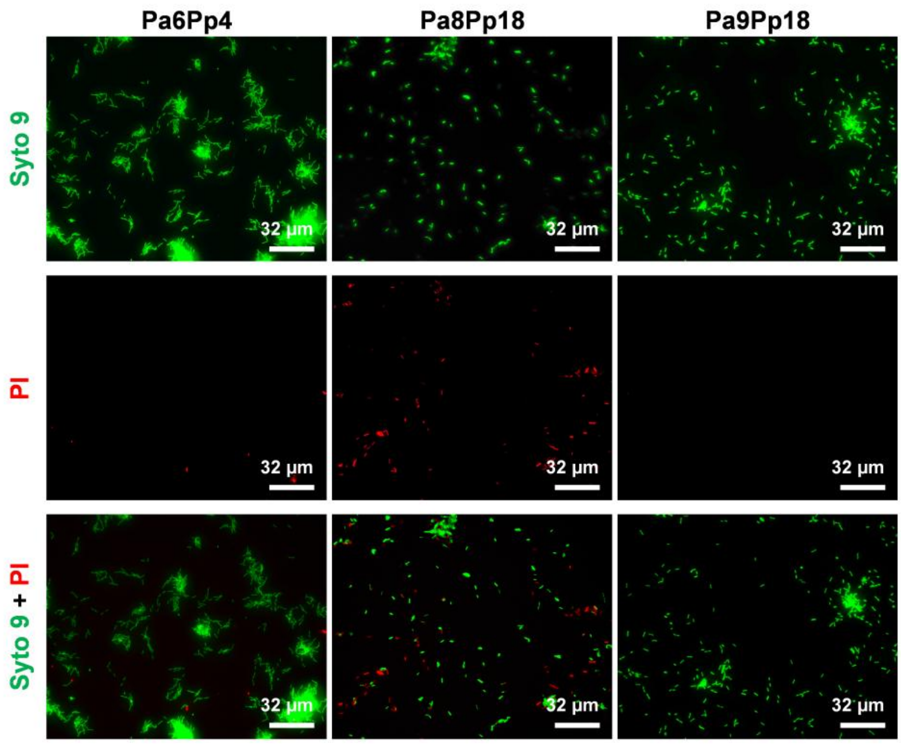

Figure S64. Live/dead staining images of *P. aeruginosa* PA49 on **PaPp**-coated coverslips after 4 h of incubation in bacterial suspension (MH medium). Syto 9 stains live bacteria, while propidium iodide (PI) stains dead bacteria. Brightness, contrast, and saturation were adjusted for visualization purposes.

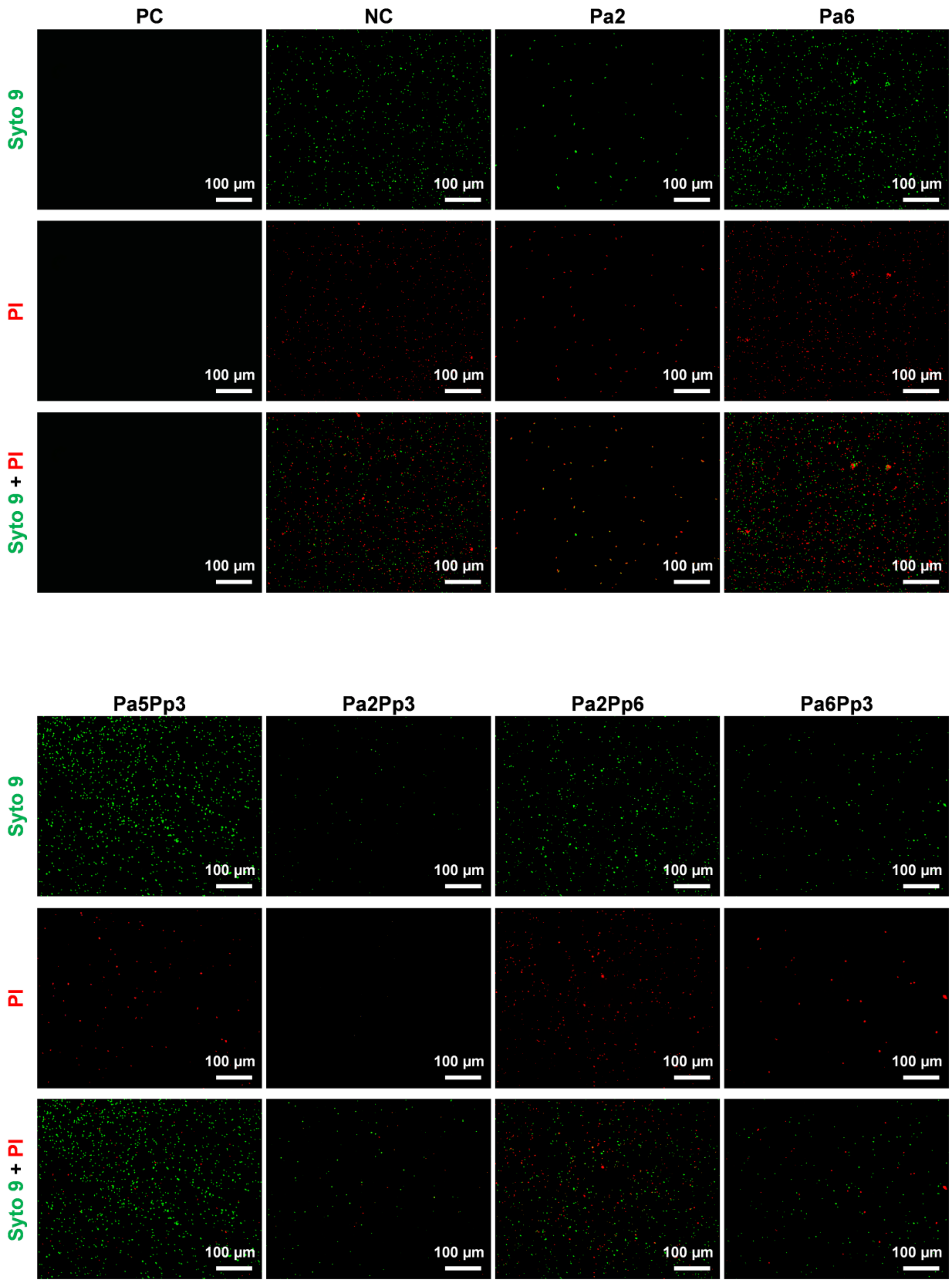

Figure S65. Live/dead staining images of *E. coli* DSM498 on **PaPp**-coated coverslips after 4 h of incubation in bacterial suspension (MH medium). Syto 9 stains live bacteria, while propidium iodide (PI) stains dead bacteria. PC = positive control, uncoated coverslips incubated in MH medium. NC = negative control, uncoated coverslips incubated in bacterial suspension. Brightness, contrast, and saturation were adjusted for visualization purposes.

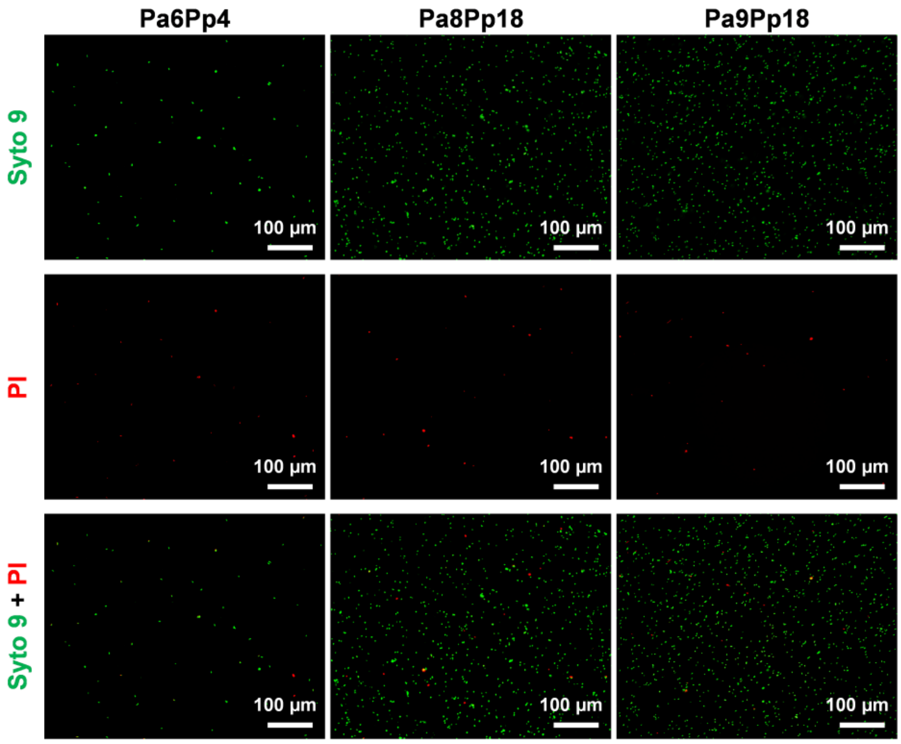

Figure S66. Live/dead staining images of *E. coli* DSM498 on **PaPp**-coated coverslips after 4 h of incubation in bacterial suspension (MH medium). Syto 9 stains live bacteria, while propidium iodide (PI) stains dead bacteria. Brightness, contrast, and saturation were adjusted for visualization purposes.

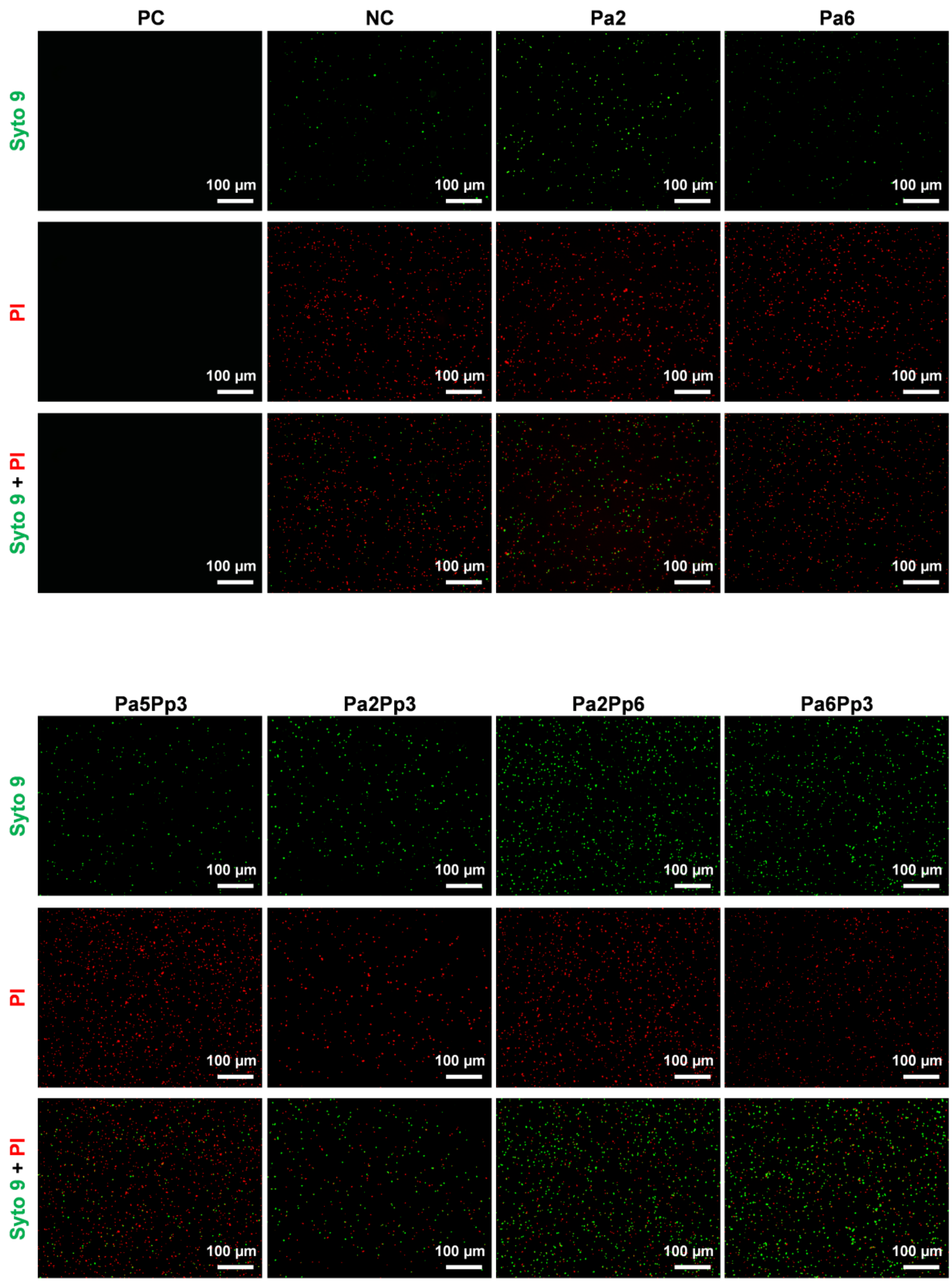

Figure S67. Live/dead staining images of *S. aureus* A1 on **PaPp**-coated coverslips after 4 h of incubation in bacterial suspension (MH medium). Syto 9 stains live bacteria, while propidium iodide (PI) stains dead bacteria. PC = positive control, uncoated coverslips incubated in MH medium. NC = negative control, uncoated coverslips incubated in bacterial suspension. Brightness, contrast, and saturation were adjusted for visualization purposes.

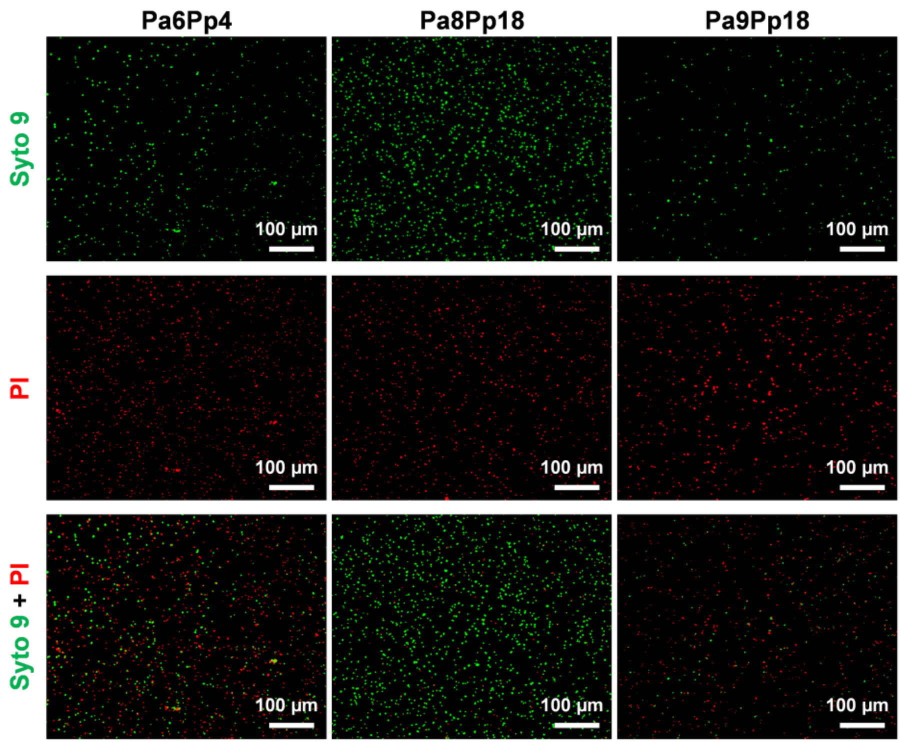

Figure S68. Live/dead staining images of *S. aureus* A1 on **PaPp**-coated coverslips after 4 h of incubation in bacterial suspension (MH medium). Syto 9 stains live bacteria, while propidium iodide (PI) stains dead bacteria. Brightness, contrast, and saturation were adjusted for visualization purposes.

a) *P. aeruginosa* PA49

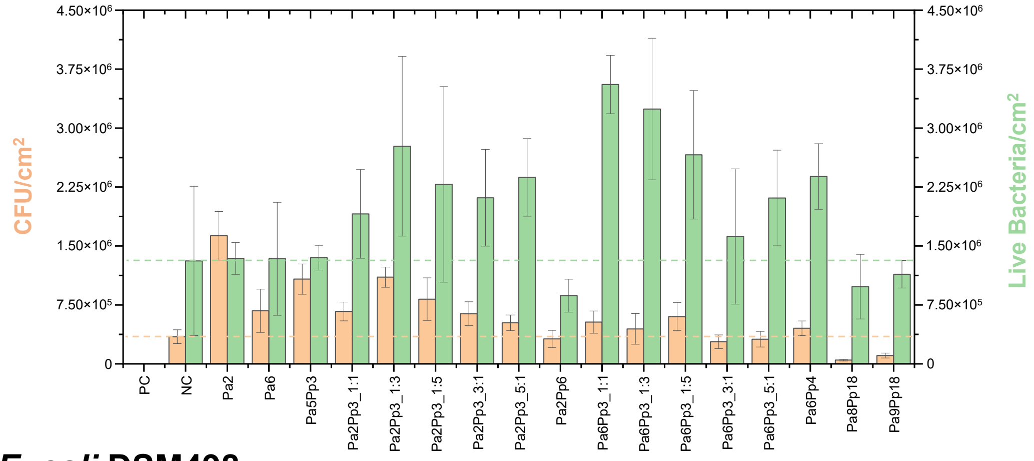

b) *E. coli* DSM498

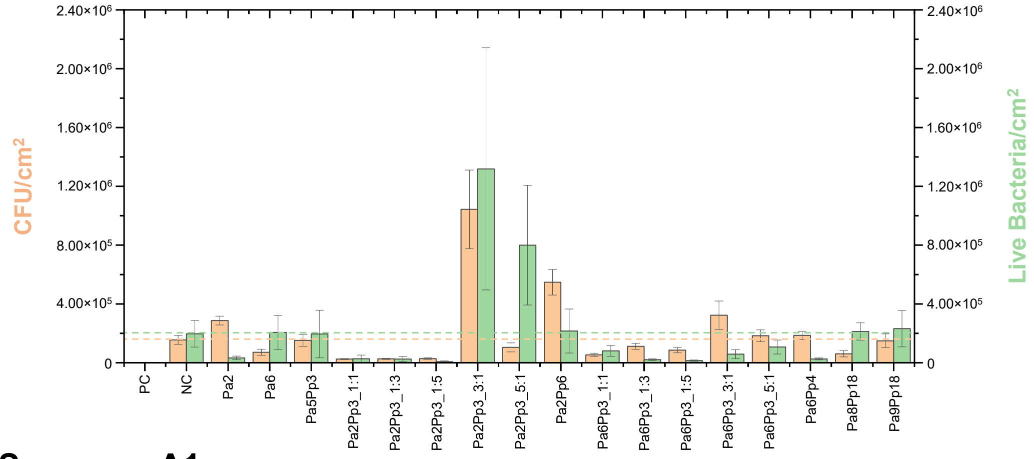

c) *S. aureus* A1

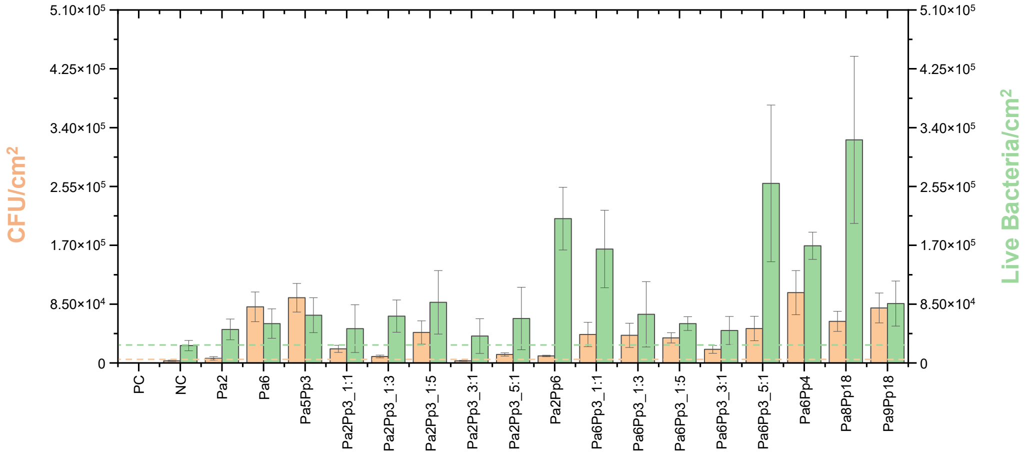

Figure S69. Recovered colony-forming units (CFUs) and number of live bacteria (Syto 9-stained) on **PaPp**-coated coverslips against a) *P. aeruginosa* PA49, b) *E. coli* DSM498, and c) *S. aureus* A1. Data represent the mean of seven data points for live bacteria and fifteen data points for recovered CFUs, each obtained from triplicate experiments, except for the live/dead assay of **Pa2Pp3** and **Pa6Pp3** against *E. coli*, for which thirteen data points were obtained from five experiments. Reference coatings: positive control (PC; uncoated coverslips in MH medium), negative control (NC; uncoated coverslips in bacterial suspension), and non-hit coatings (**Pa2**, **Pa6**, **Pa5Pp3**). Dashed lines indicate the NC reference level.

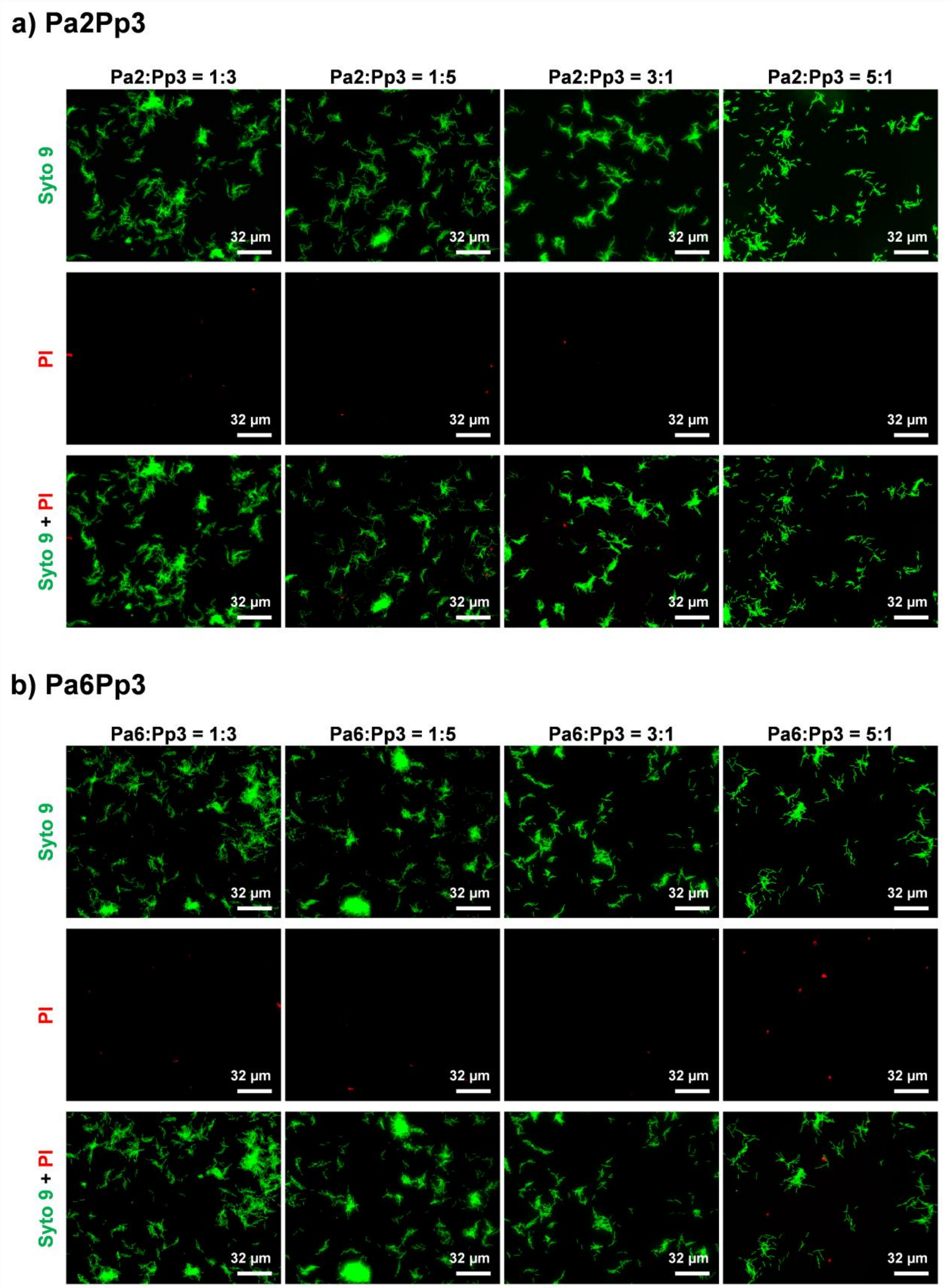

Figure S70. Live/dead staining images of *P. aeruginosa* PA49 on a) **Pa2Pp3**- and b) **Pa6Pp3**-coated coverslips after 4 h of incubation in bacterial suspension (MH medium). The **Pa:Pp** ratio was varied. Syto 9 stains live bacteria, while propidium iodide (PI) stains dead bacteria. PC = positive control, uncoated coverslips incubated in MH medium. NC = negative control, uncoated coverslips incubated in PA49 suspension. Brightness, contrast, and saturation were adjusted for visualization purposes.

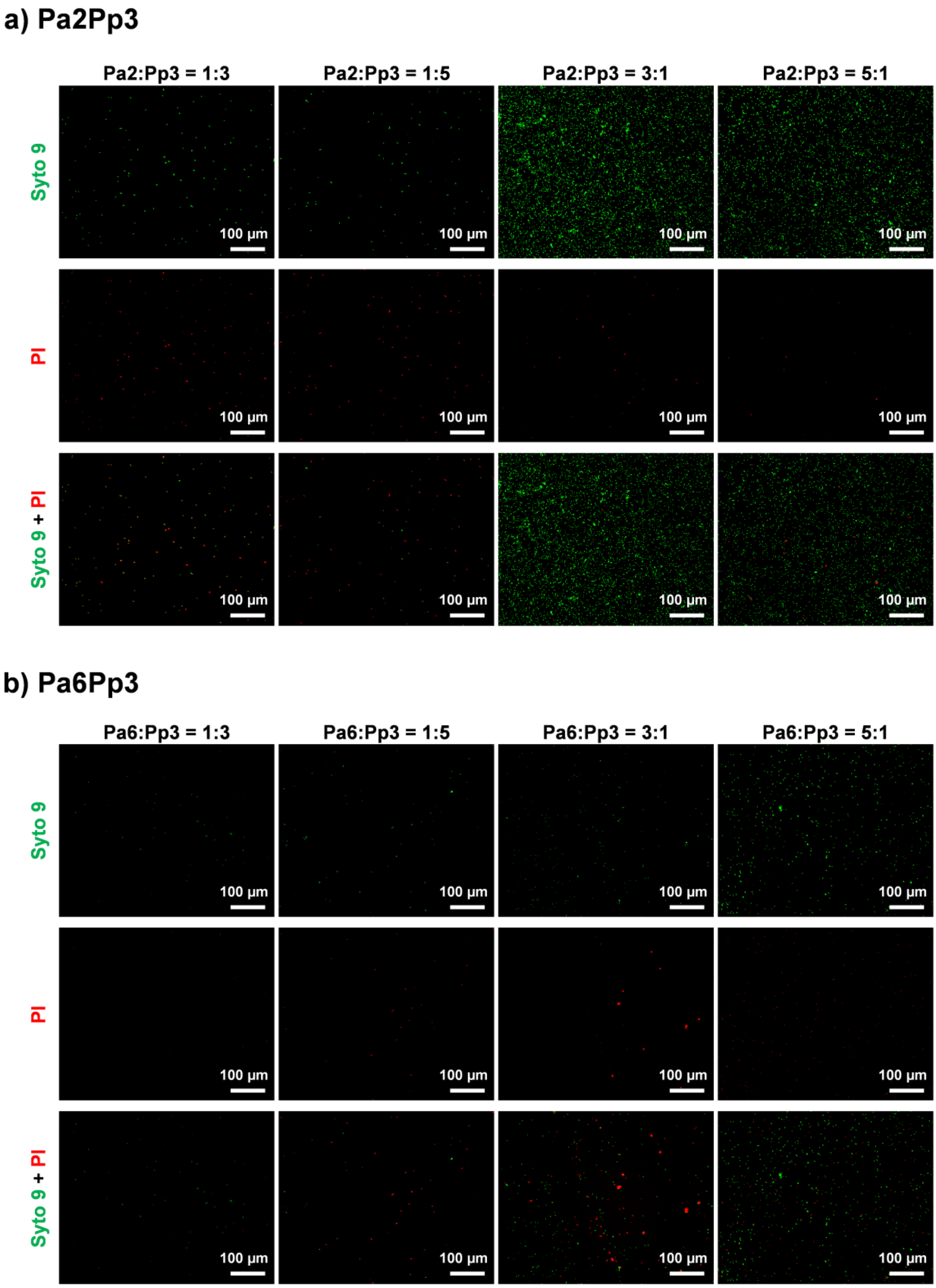

Figure S71. Live/dead staining images of *E. coli* DSM498 on a) **Pa2Pp3**- and b) **Pa6Pp3**-coated coverslips after 4 h of incubation in bacterial suspension (MH medium). The **Pa:Pp** ratio was varied. Syto 9 stains live bacteria, while propidium iodide (PI) stains dead bacteria. PC = positive control, uncoated coverslips incubated in MH medium. NC = negative control, uncoated coverslips incubated in bacterial suspension. Brightness, contrast, and saturation were adjusted for visualization purposes.

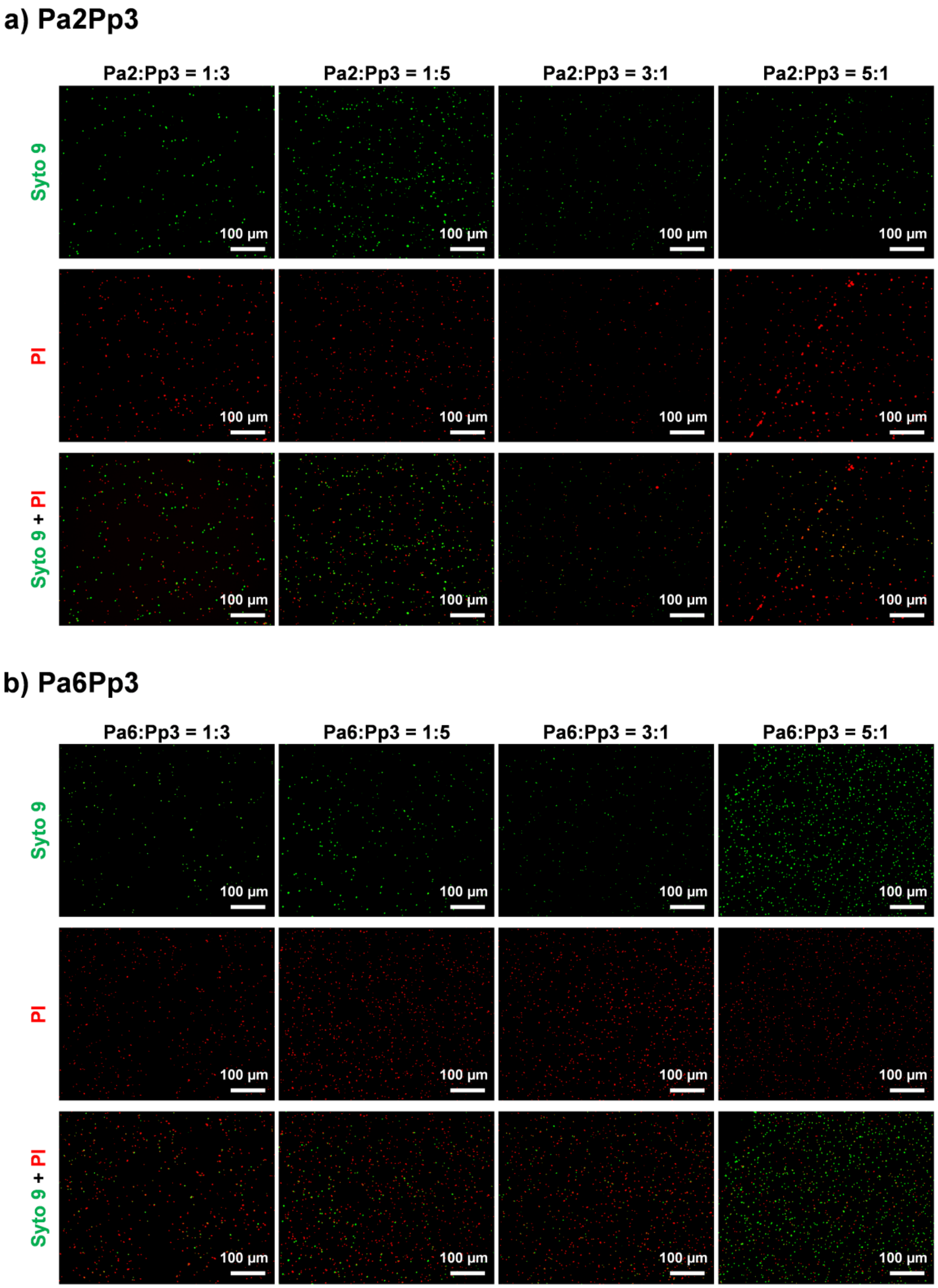

Figure S72. Live/dead staining images of *S. aureus* A1 on a) **Pa2Pp3**- and b) **Pa6Pp3**-coated coverslips after 4 h of incubation in bacterial suspension (MH medium). The **Pa:Pp** ratio was varied. Syto 9 stains live bacteria, while propidium iodide (PI) stains dead bacteria. PC = positive control, uncoated coverslips incubated in MH medium. NC = negative control, uncoated coverslips incubated in bacterial suspension. Brightness, contrast, and saturation were adjusted for visualization purposes.

8. UHT Screening of Coating Compatibility with Adherent Human Cells

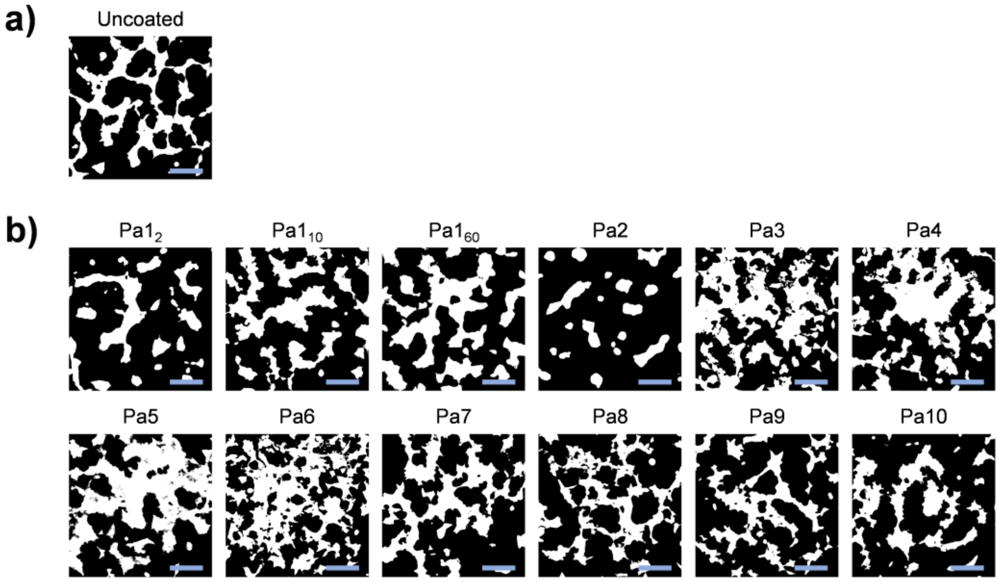

Figure S73. Binary masks of HeLa-RFP cells on a) uncoated and b) **Pa**-coated spots of the DMA, showing cell morphology. Cells on **Pa**-coated spots exhibit altered spreading and clustering compared to the uncoated spot. Scale bar: 150 µm.

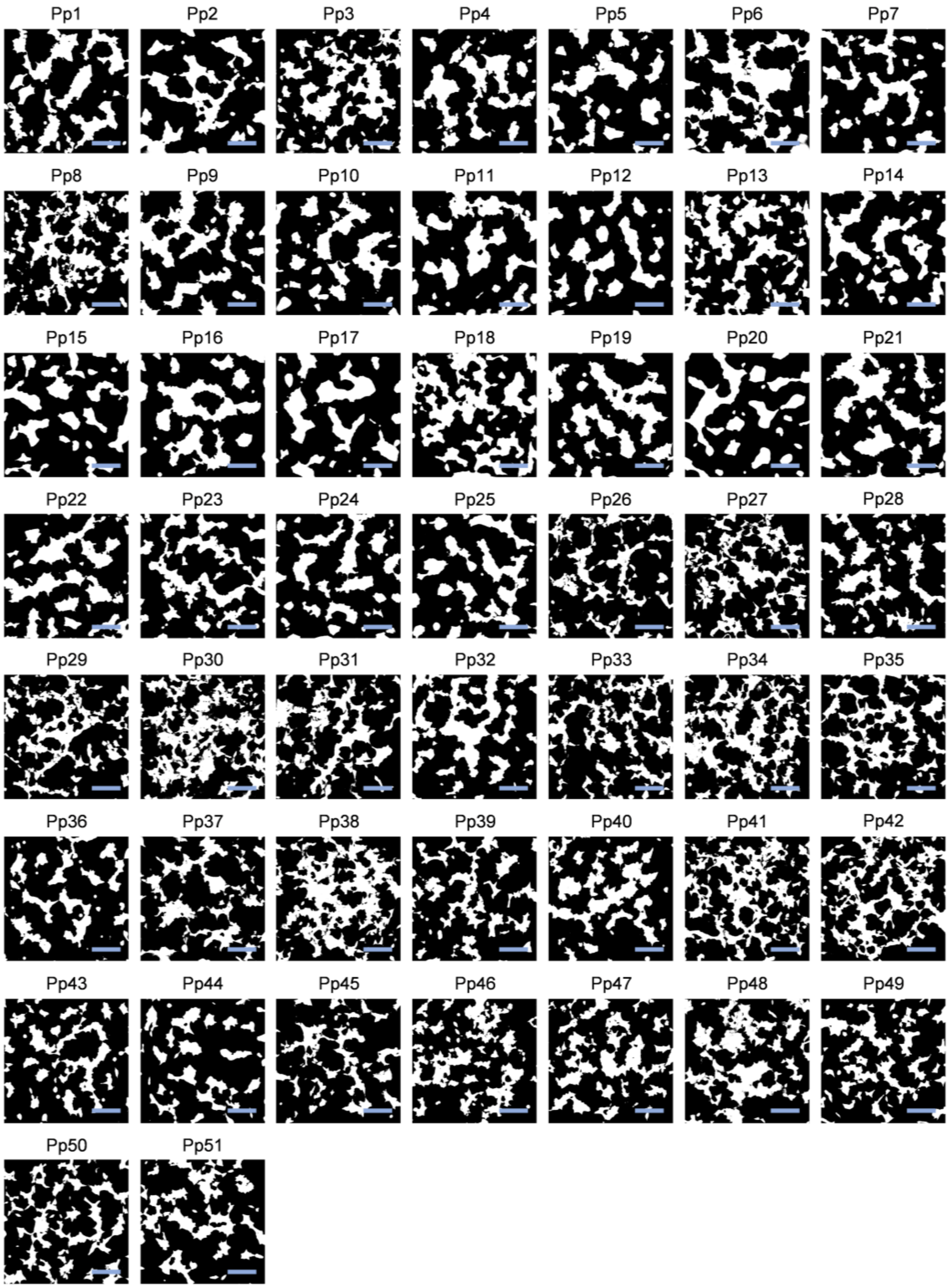

Figure S74. Binary masks of HeLa-RFP cells on **Pp**-coated spots of the DMA, showing variations in cell morphology across different coatings. Scale bar: 150 µm.

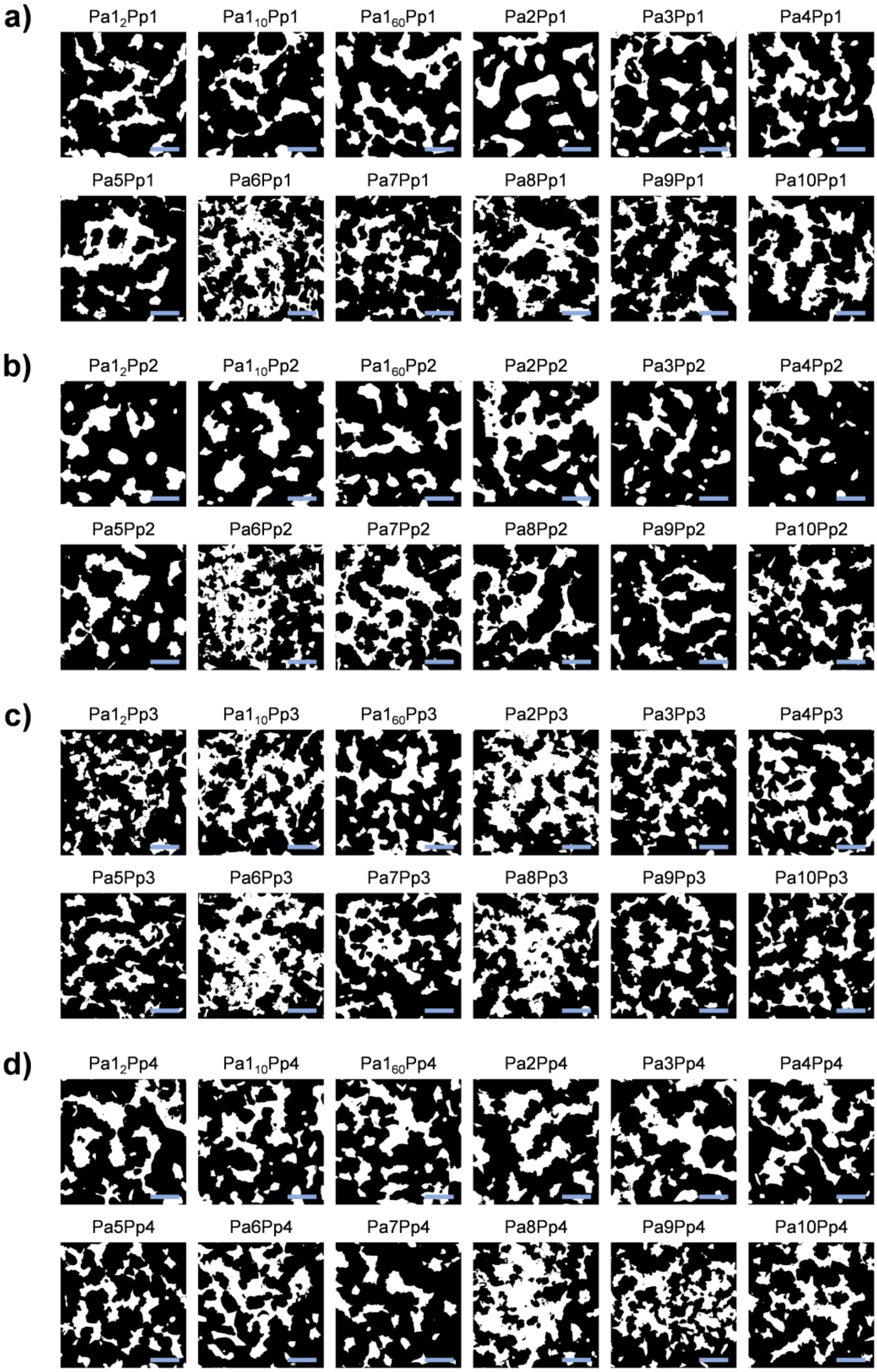

Figure S75. Binary masks of HeLa-RFP cells on coatings prepared from a) **Pp1**, b) **Pp2**, c) **Pp3**, and d) **Pp4**, each crosslinked with **Pa1$_2$-Pa10**, showing variations in cell morphology across the different coatings. Scale bar: 150 µm.

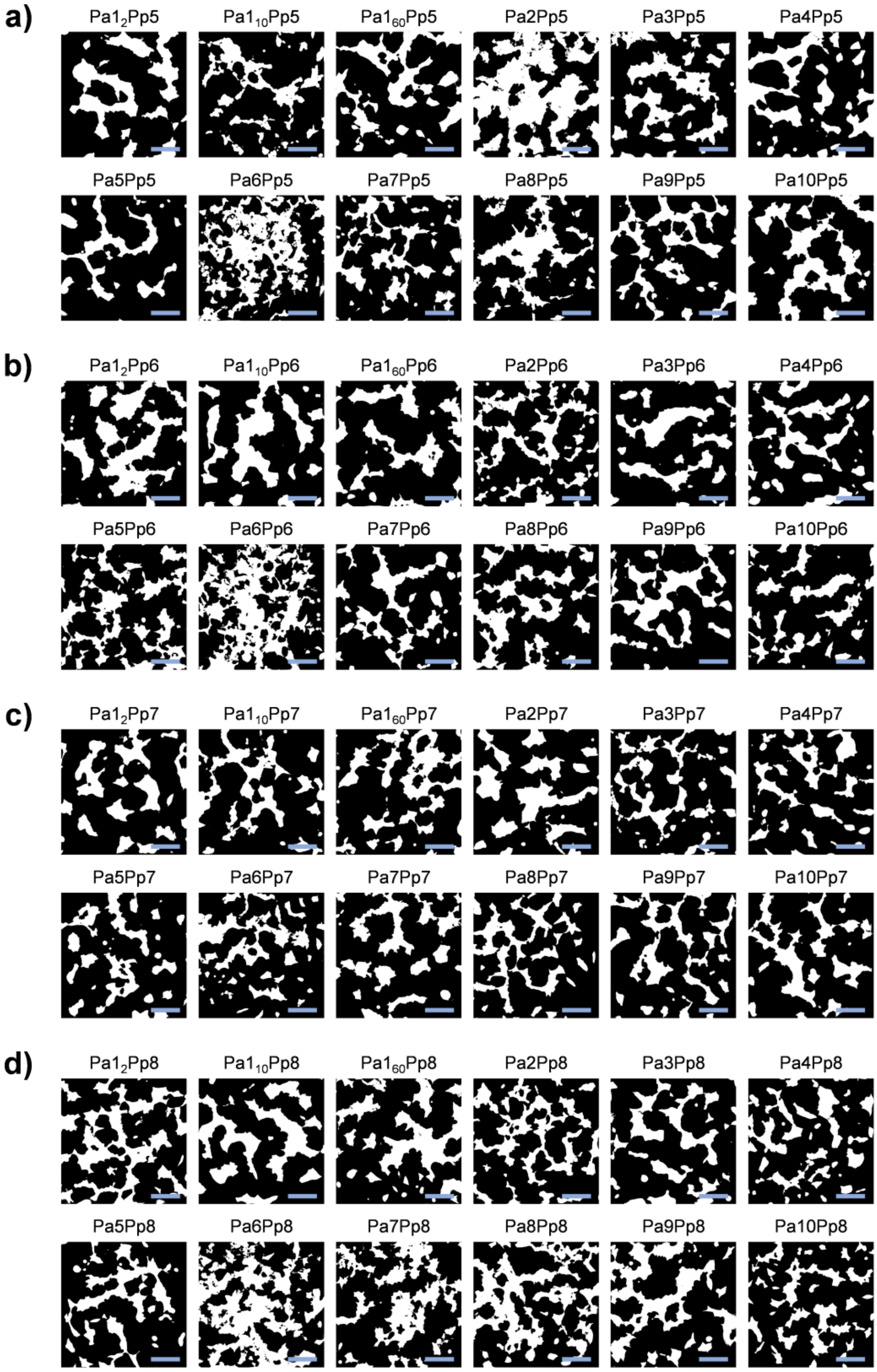

Figure S76. Binary masks of HeLa-RFP cells on coatings prepared from a) **Pp5**, b) **Pp6**, c) **Pp7**, and d) **Pp8**, each crosslinked with **Pa1$_2$-Pa10**, showing variations in cell morphology across the different coatings. Scale bar: 150 μm.

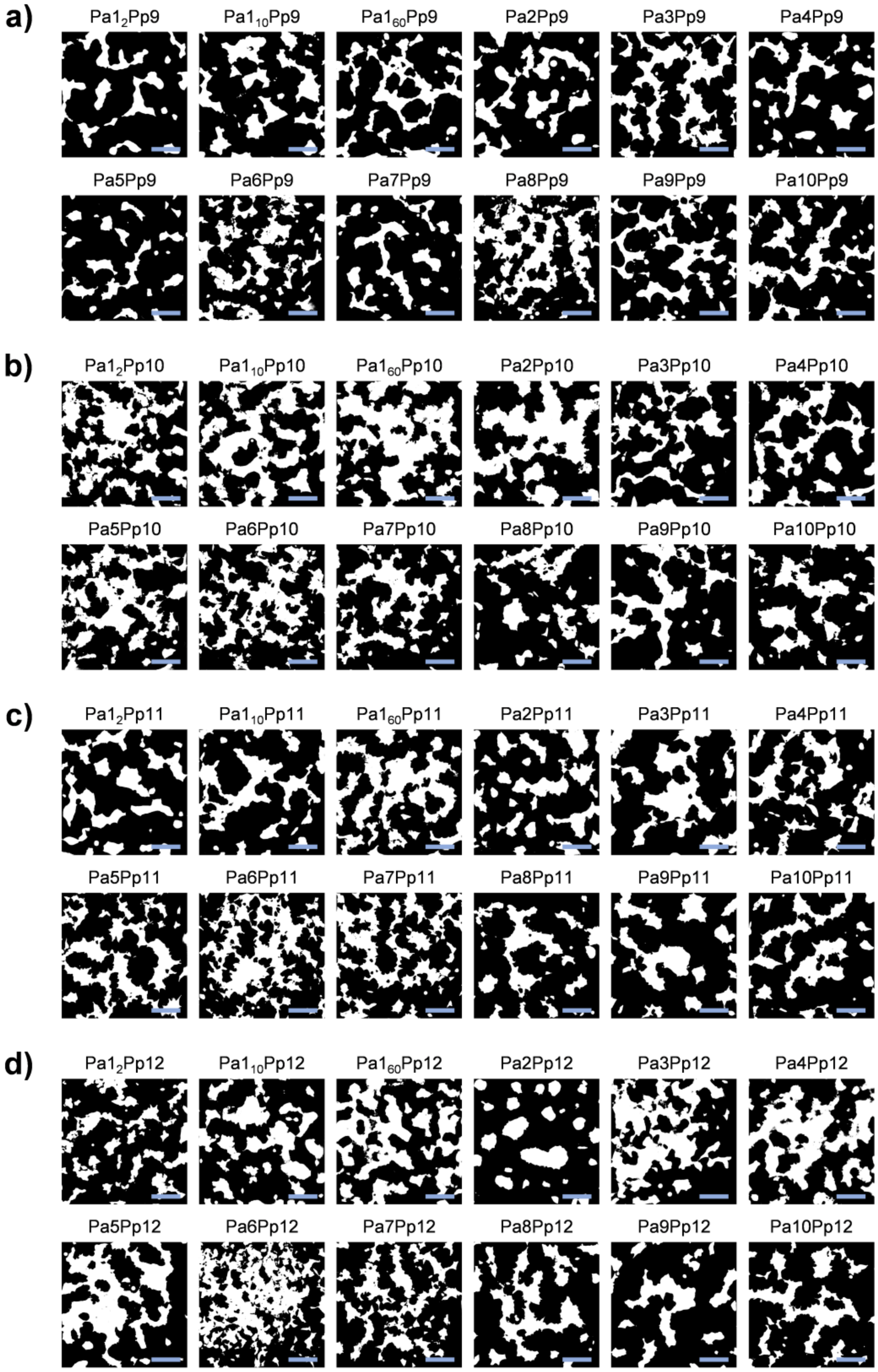

Figure S77. Binary masks of HeLa-RFP cells on coatings prepared from a) **Pp9**, b) **Pp10**, c) **Pp11**, and d) **Pp12**, each crosslinked with **Pa1$_2$-Pa10**, showing variations in cell morphology across the different coatings. Scale bar: 150 µm.

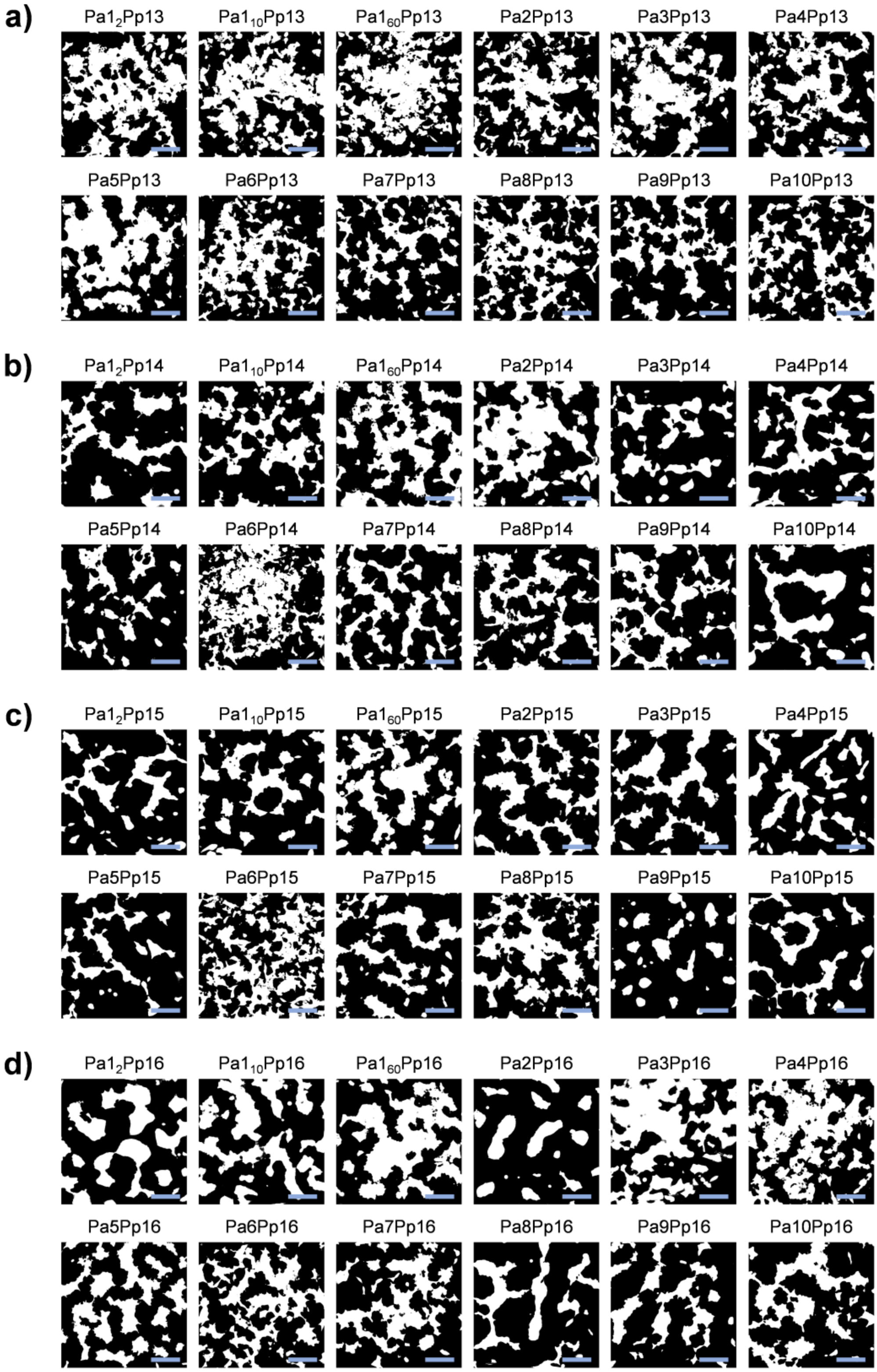

Figure S78. Binary masks of HeLa-RFP cells on coatings prepared from a) **Pp13**, b) **Pp14**, c) **Pp15**, and d) **Pp16**, each crosslinked with **Pa1$_2$-Pa10**, showing variations in cell morphology across the different coatings. Scale bar: 150 µm.

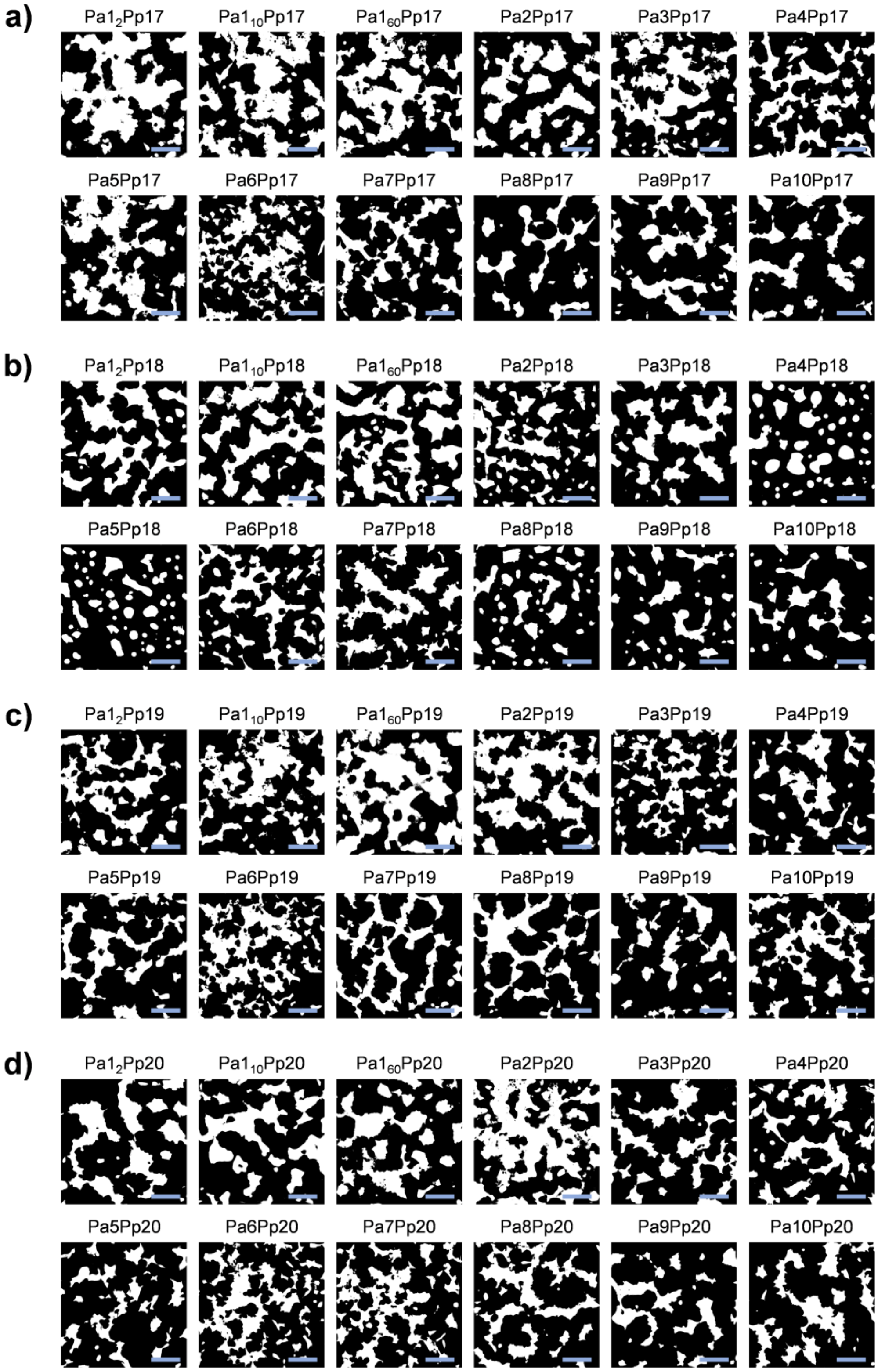

Figure S79. Binary masks of HeLa-RFP cells on coatings prepared from a) **Pp17**, b) **Pp18**, c) **Pp19**, and d) **Pp20**, each crosslinked with **Pa1$_2$-Pa10**, showing variations in cell morphology across the different coatings. Scale bar: 150 µm.

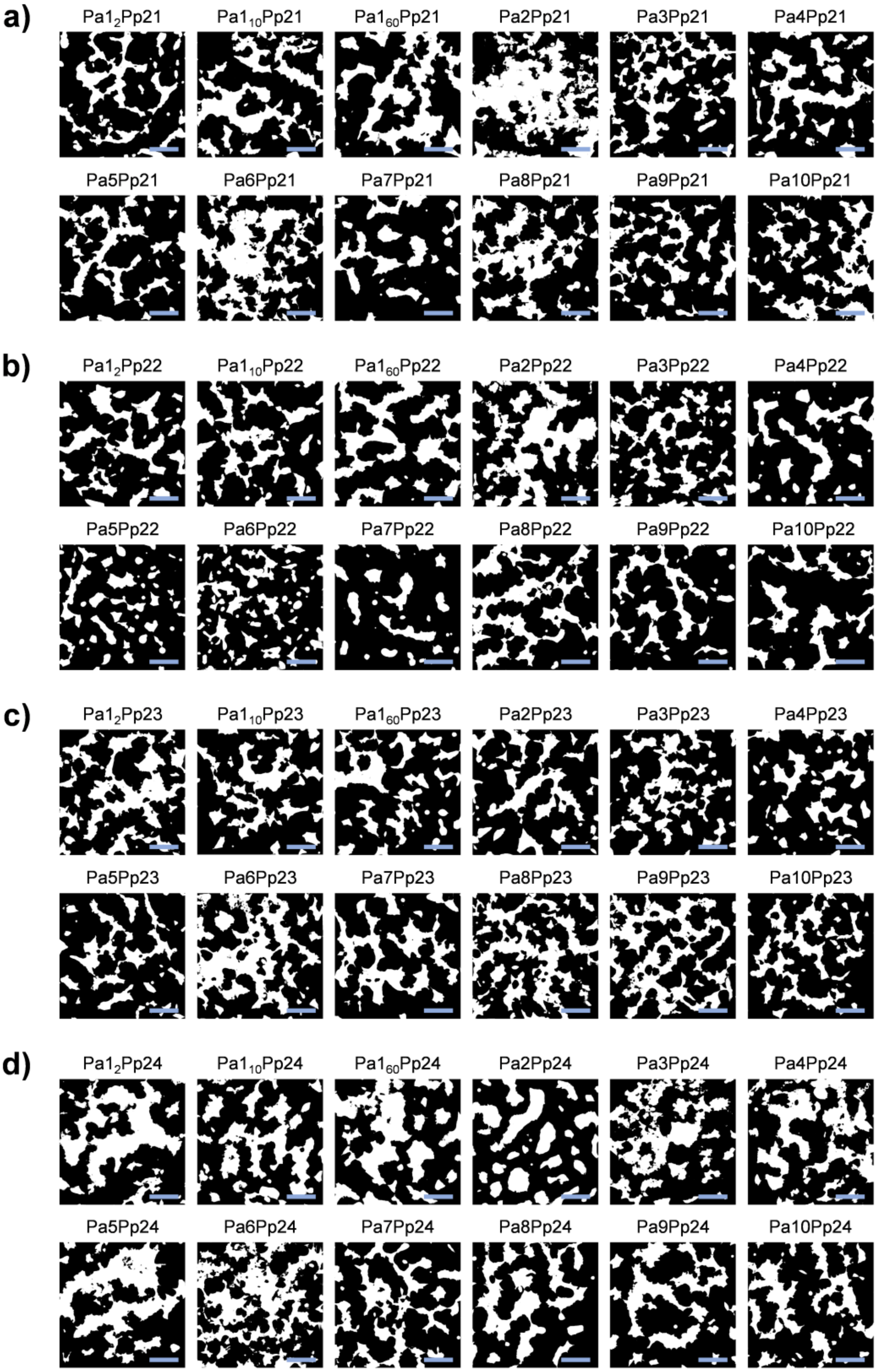

Figure S80. Binary masks of HeLa-RFP cells on coatings prepared from a) **Pp21**, b) **Pp22**, c) **Pp23**, and d) **Pp24**, each crosslinked with **$Pa1_2$-Pa10**, showing variations in cell morphology across the different coatings. Scale bar: 150 µm.

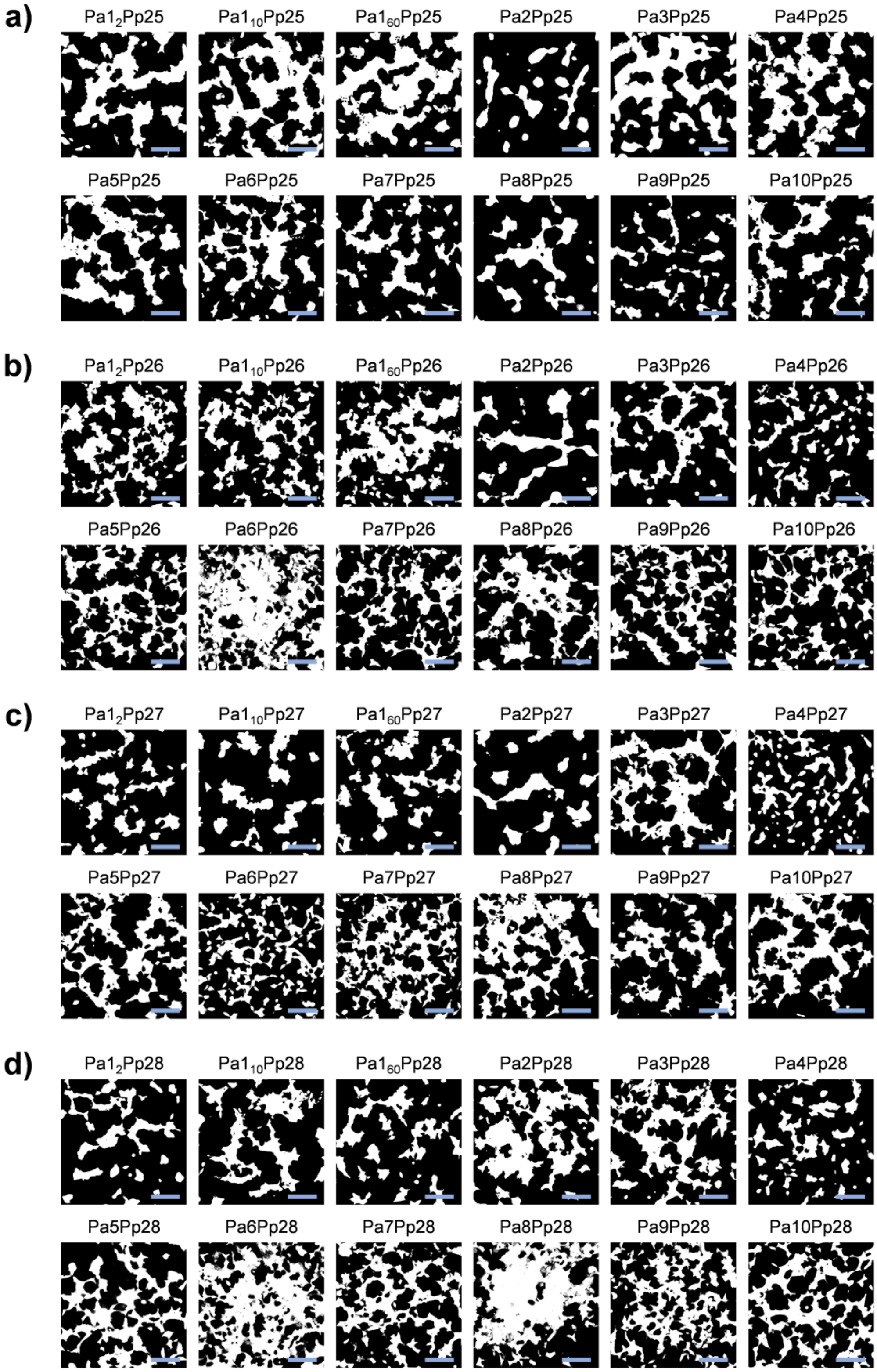

Figure S81. Binary masks of HeLa-RFP cells on coatings prepared from a) **Pp25**, b) **Pp26**, c) **Pp27**, and d) **Pp28**, each crosslinked with **Pa1$_2$-Pa10**, showing variations in cell morphology across the different coatings. Scale bar: 150 µm.

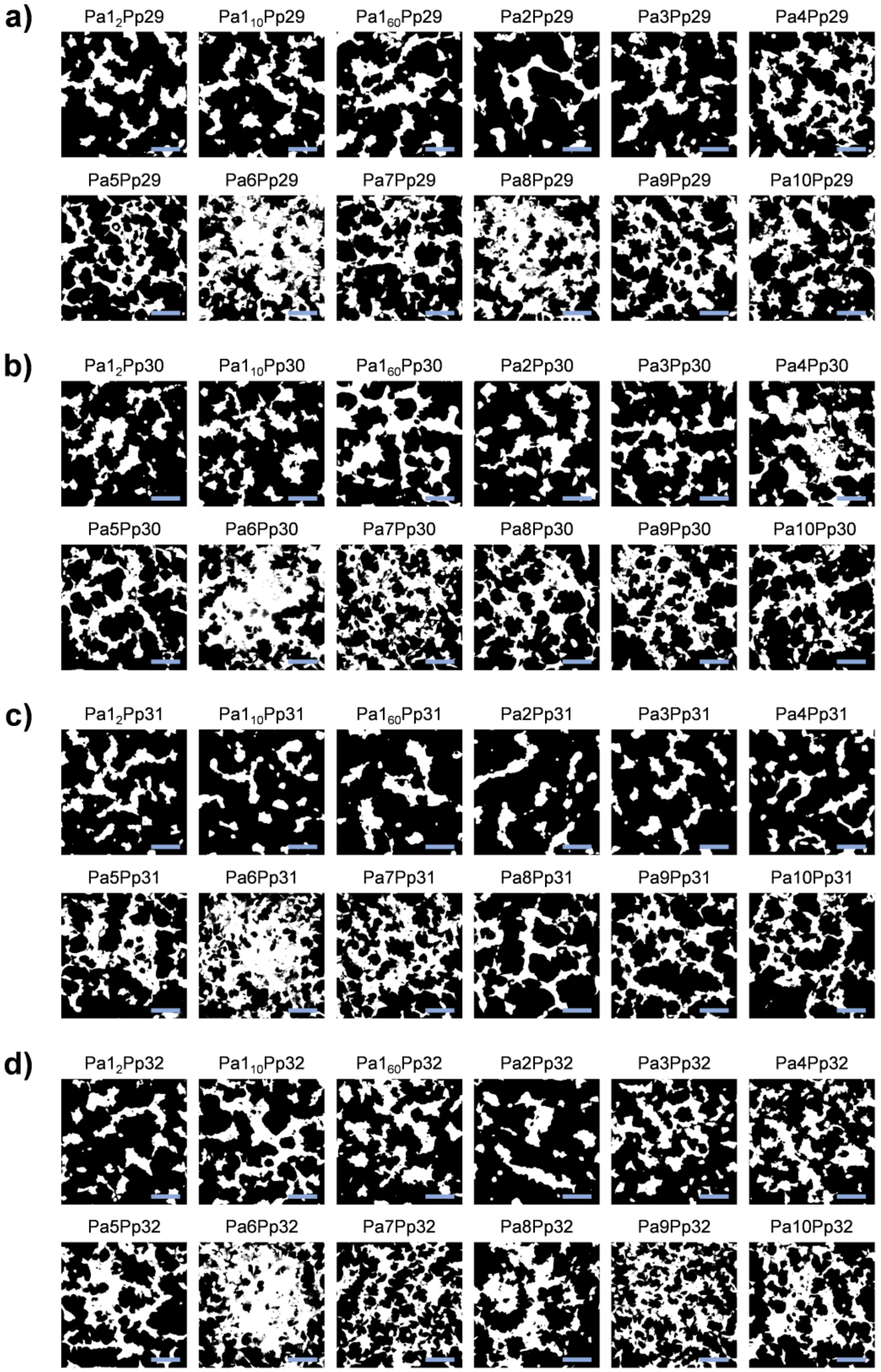

Figure S82. Binary masks of HeLa-RFP cells on coatings prepared from a) **Pp29**, b) **Pp30**, c) **Pp31**, and d) **Pp32**, each crosslinked with **Pa1$_2$-Pa10**, showing variations in cell morphology across the different coatings. Scale bar: 150 µm.

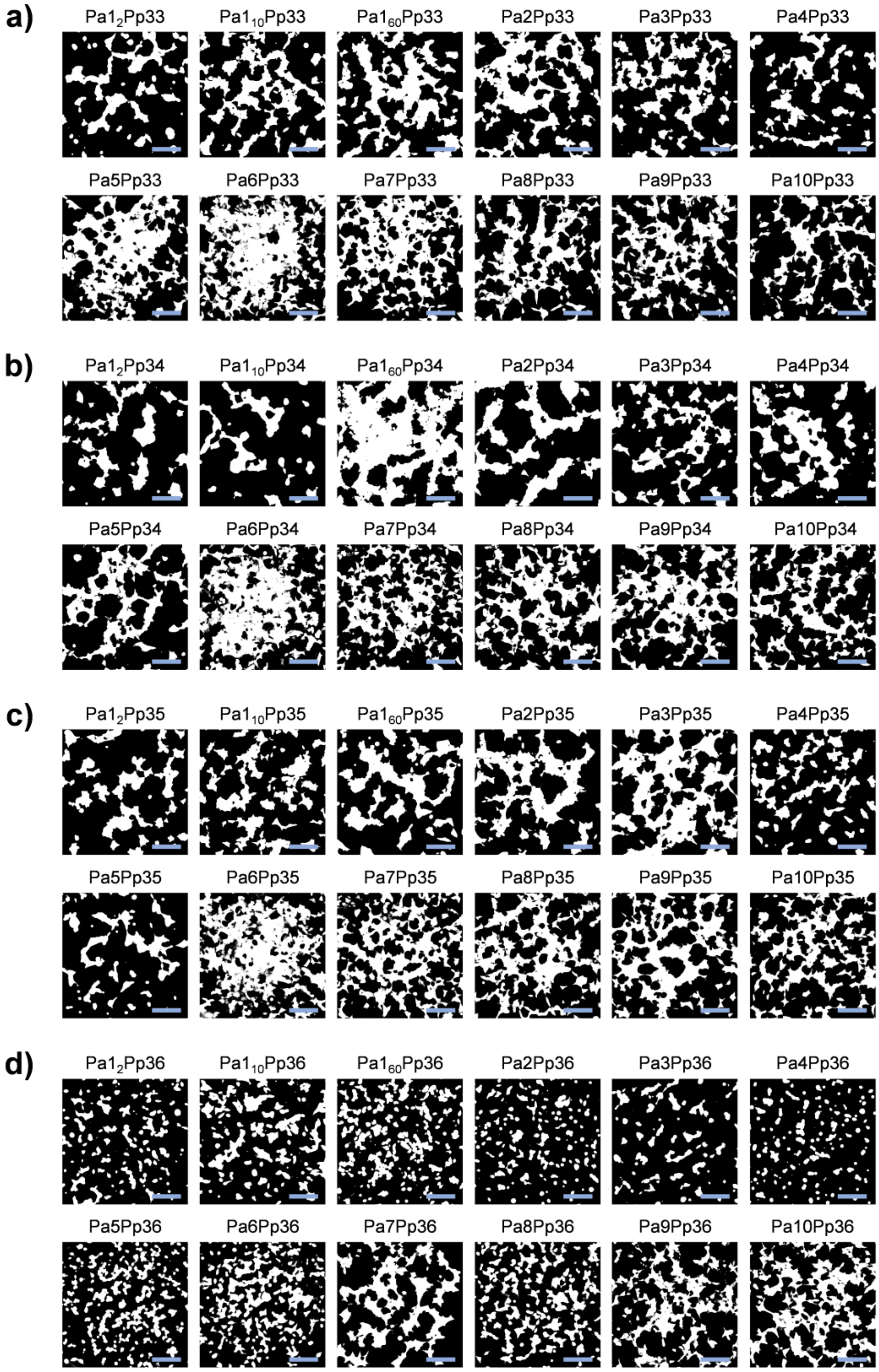

Figure S83. Binary masks of HeLa-RFP cells on coatings prepared from a) **Pp33**, b) **Pp34**, c) **Pp35**, and d) **Pp36**, each crosslinked with **Pa1$_2$-Pa10**, showing variations in cell morphology across the different coatings. Scale bar: 150 µm.

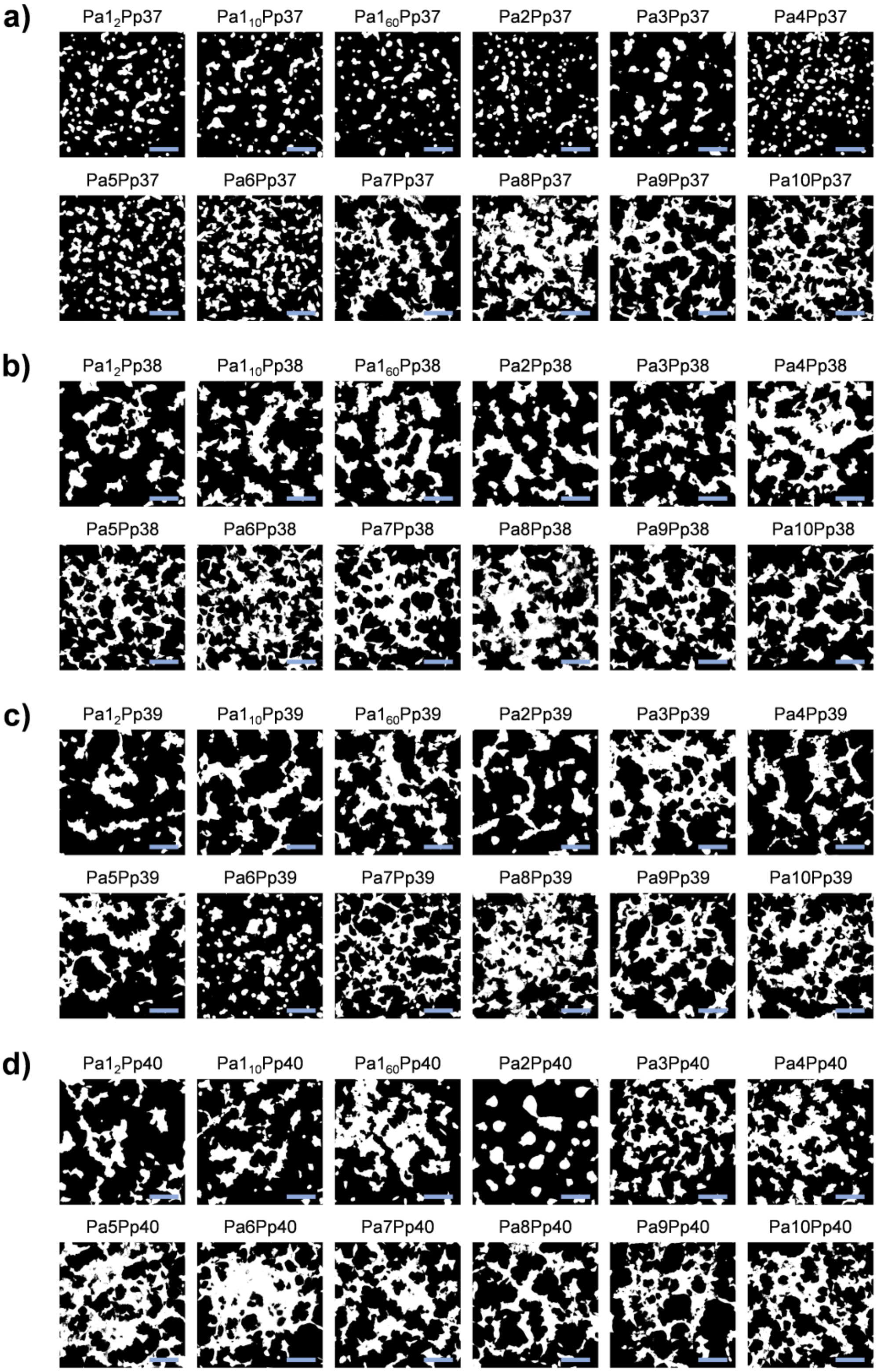

Figure S84. Binary masks of HeLa-RFP cells on coatings prepared from a) **Pp37**, b) **Pp38**, c) **Pp39**, and d) **Pp40**, each crosslinked with **Pa1$_2$-Pa10**, showing variations in cell morphology across the different coatings. Scale bar: 150 µm.

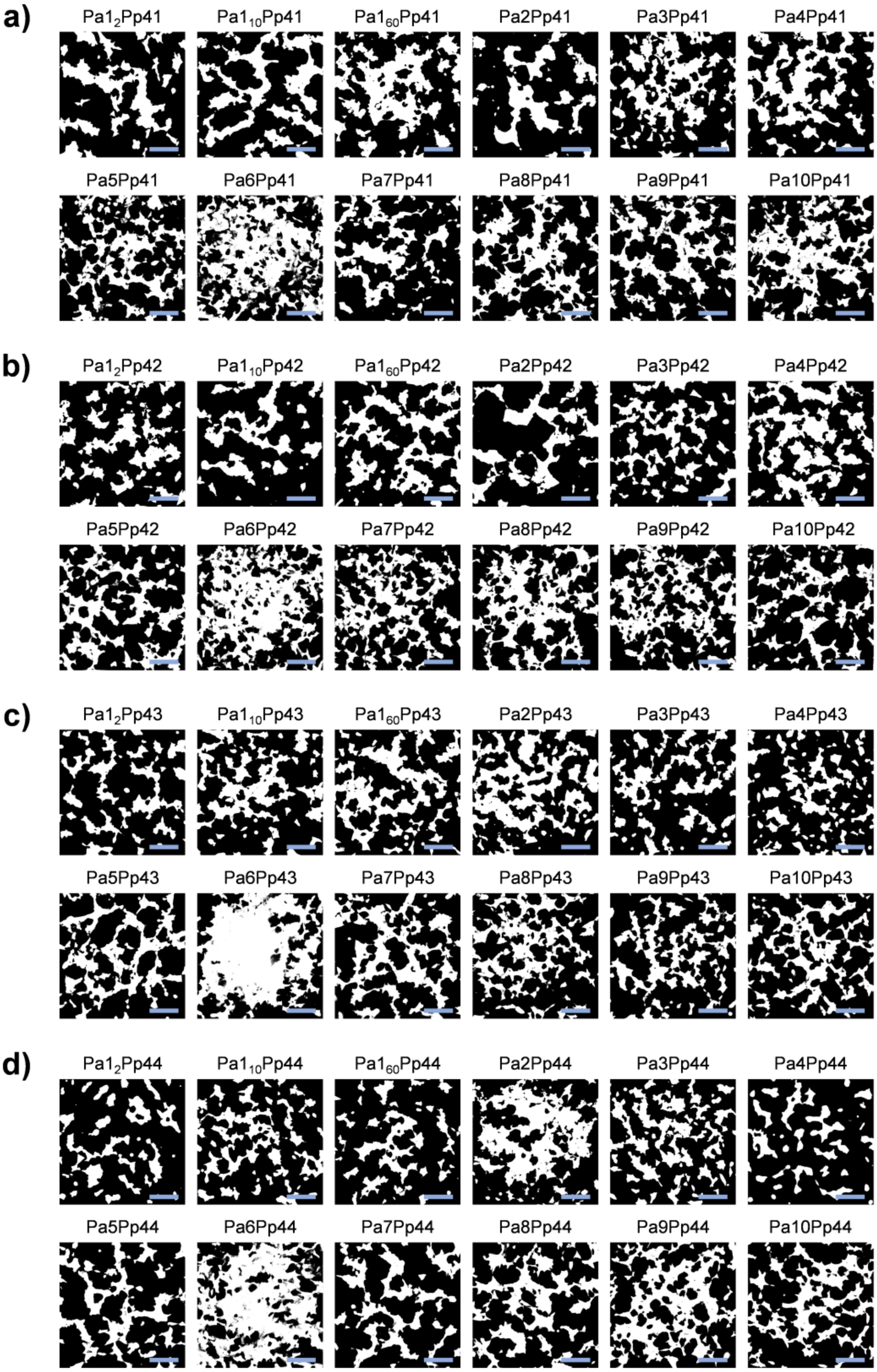

Figure S85. Binary masks of HeLa-RFP cells on coatings prepared from a) **Pp41**, b) **Pp42**, c) **Pp43**, and d) **Pp44**, each crosslinked with **Pa1$_2$-Pa10**, showing variations in cell morphology across the different coatings. Scale bar: 150 µm.

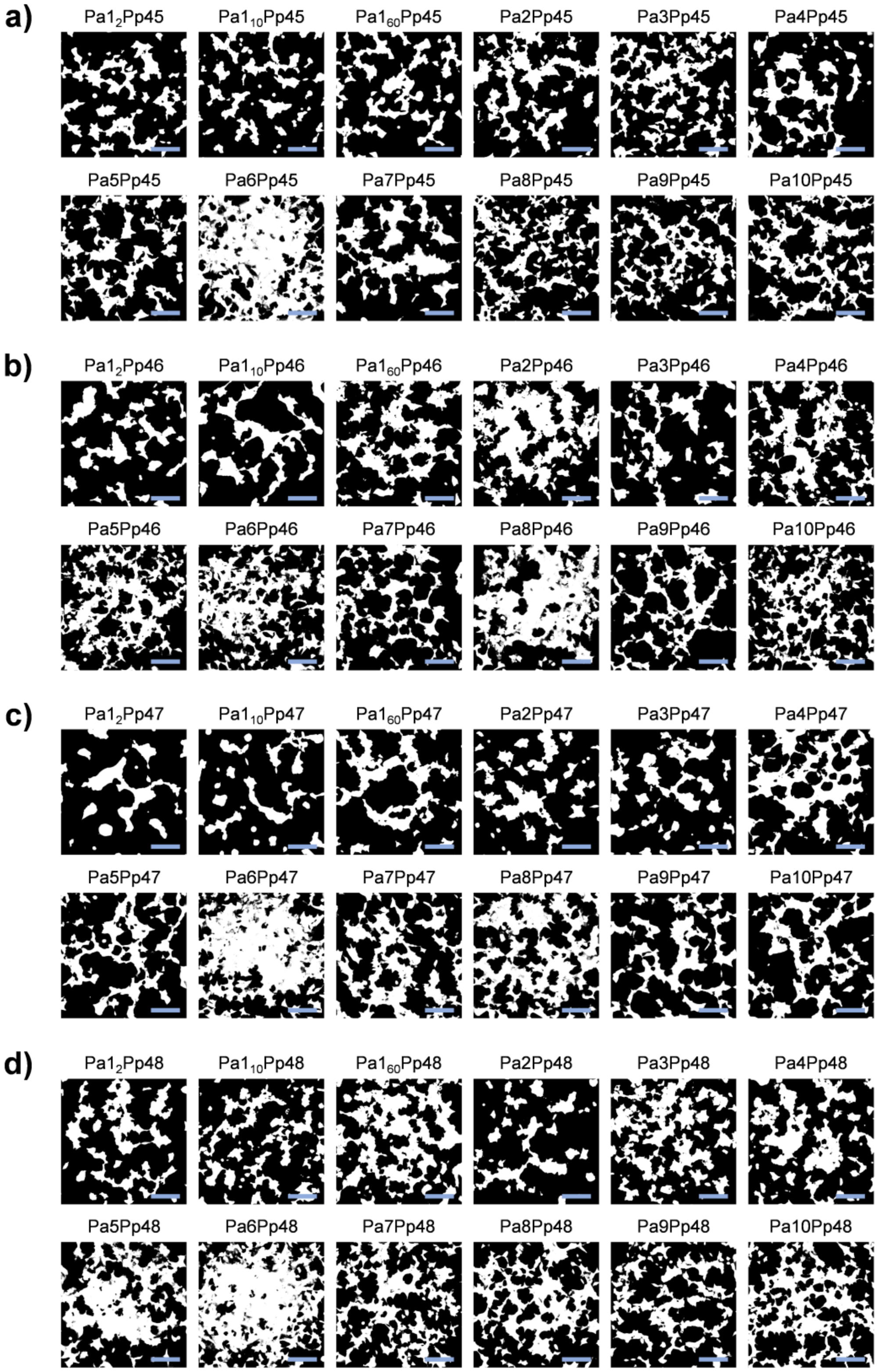

Figure S86. Binary masks of HeLa-RFP cells on coatings prepared from a) **Pp45**, b) **Pp46**, c) **Pp47**, and d) **Pp48**, each crosslinked with **Pa1$_2$-Pa10**, showing variations in cell morphology across the different coatings. Scale bar: 150 µm.

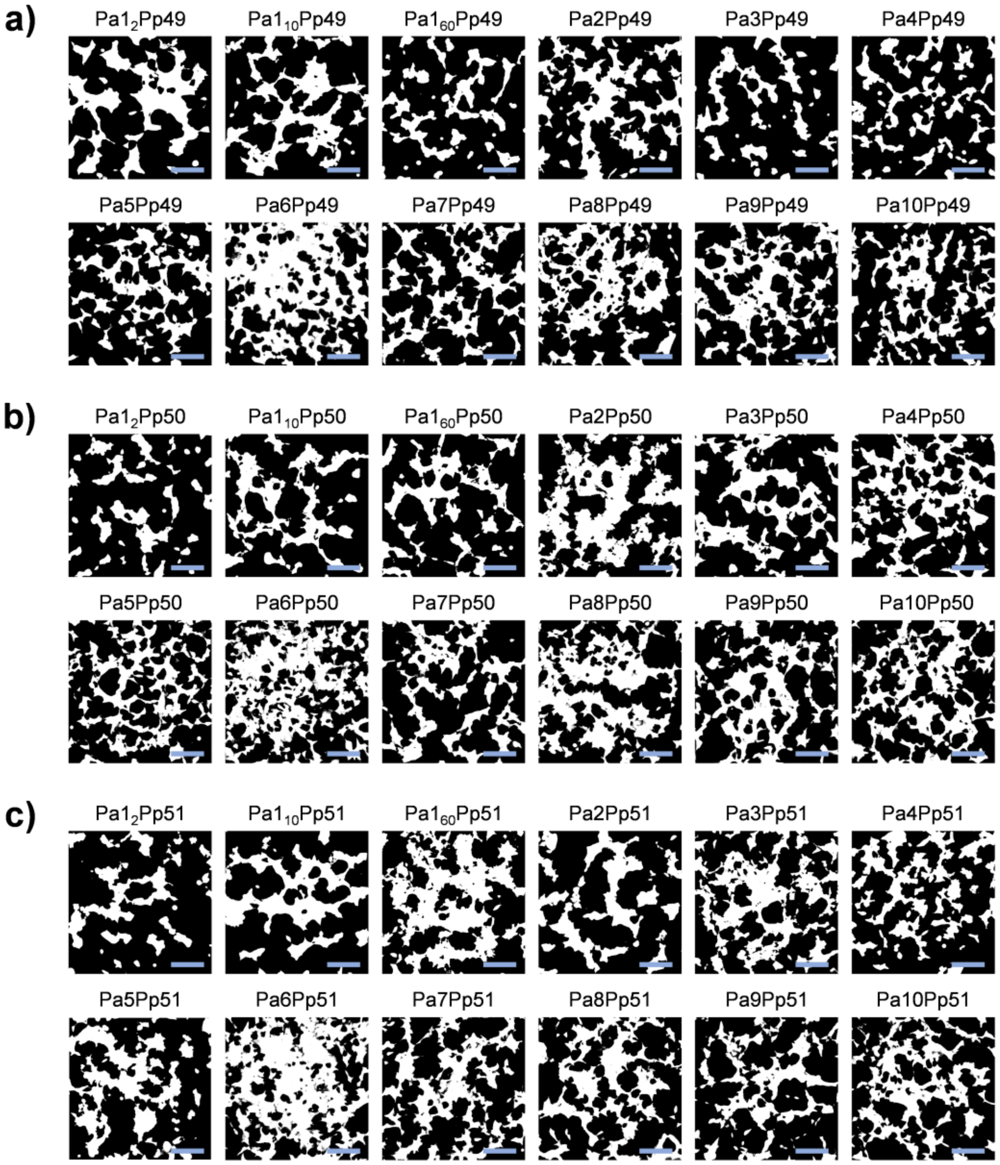

Figure S87. Binary masks of HeLa-RFP cells on coatings prepared from a) **Pp49**, b) **Pp50**, and c) **Pp51**, each crosslinked with **Pa1$_2$-Pa10**, showing variations in cell morphology across the different coatings. Scale bar: 150 µm.

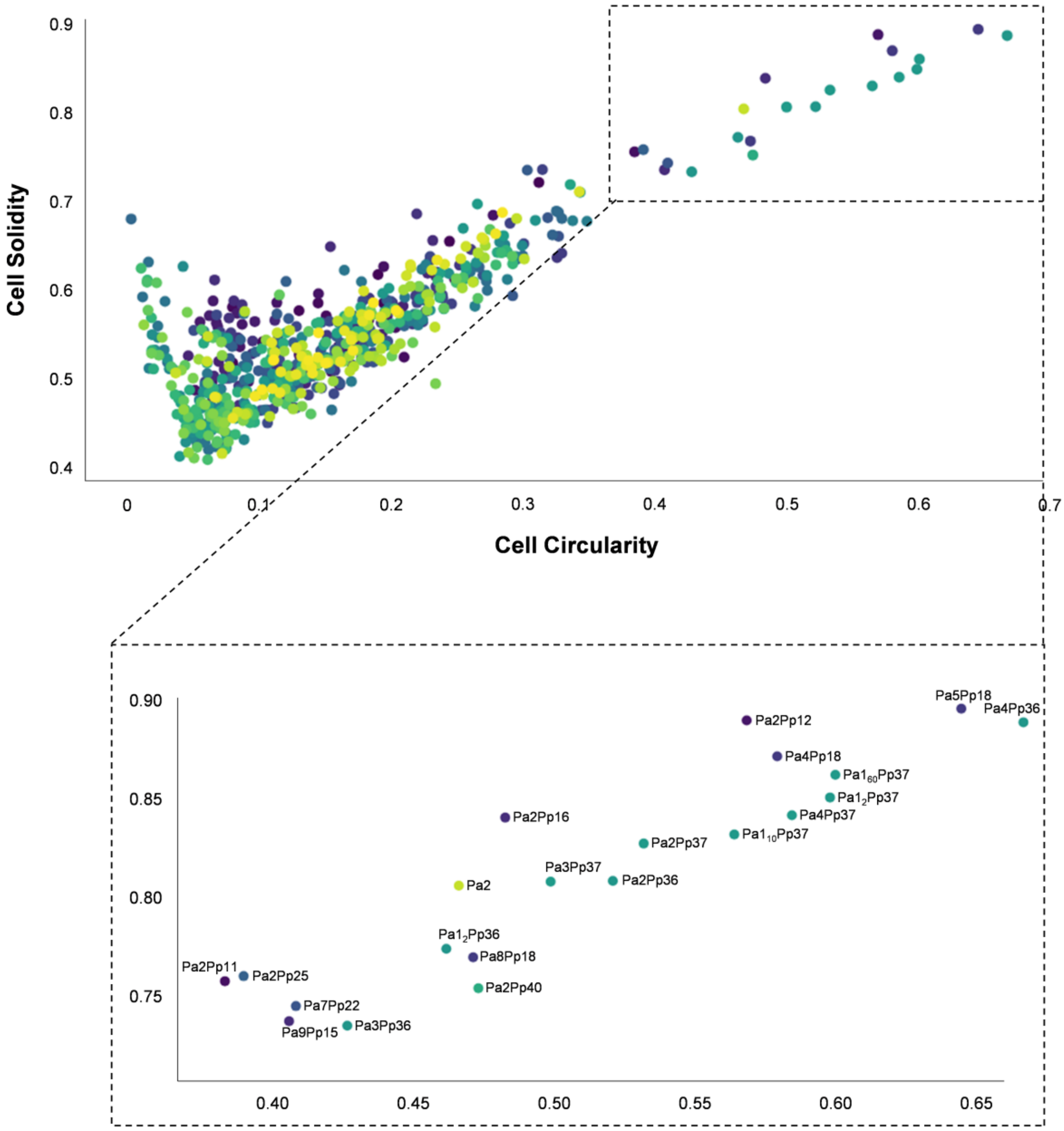

Figure S88. Correlation between cell solidity and circularity of HeLa-RFP cells cultured on 675 **PaPp** combinations (12 **Pa**, 51 **Pp**, and 612 **PaPp**). Triplicate samples of the 675 coatings were randomly distributed across four DMAs. Data shown represent mean values.

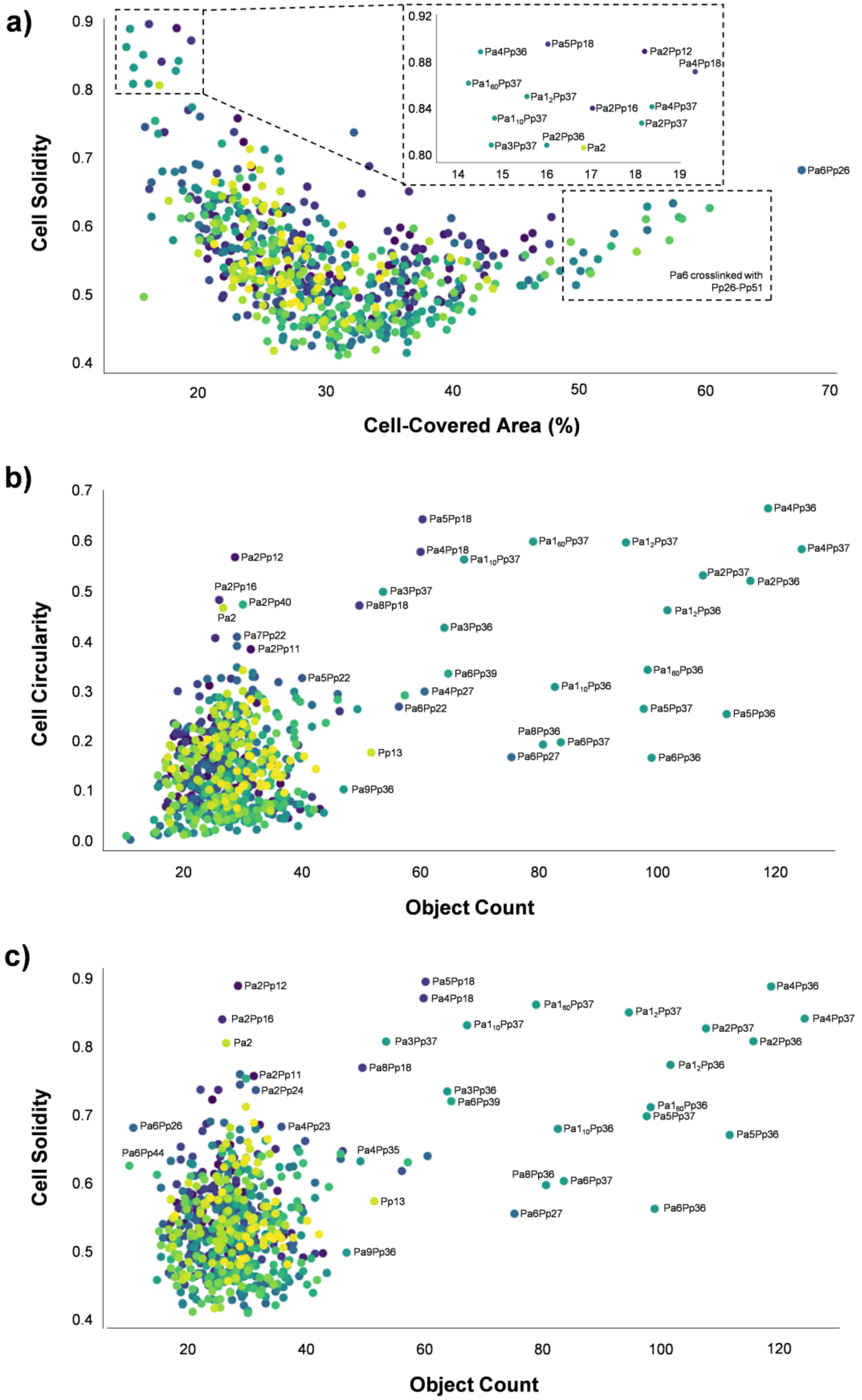

Figure S89. Correlation between a) cell solidity and covered area, b) cell circularity and object count, and c) cell solidity and object count for HeLa-RFP cells cultured on 675 **PaPp** combinations (12 **Pa**, 51 **Pp**, and 612 **PaPp**). Triplicate samples of the 675 coatings were randomly distributed across four DMAs. Data shown represent mean values.

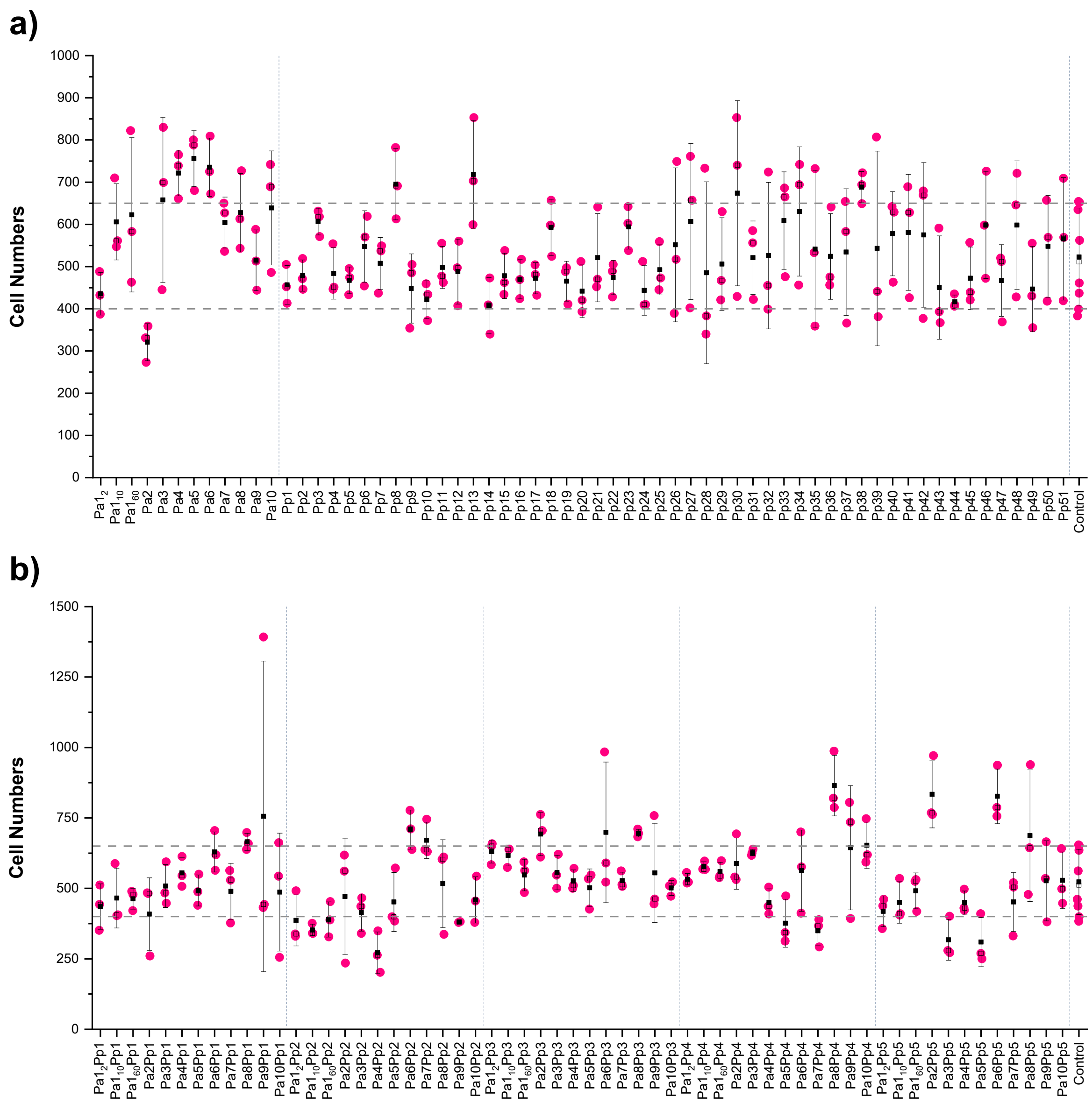

Figure S90. Numbers of Hoechst-stained nuclei of HeLa-RFP cells cultured on a) individual precursors **Pa1$_2$-Pa10** and **Pp1-Pp51**, and b) **PaPp** coatings prepared from **Pa1$_2$-Pa10** crosslinked with **Pp1-Pp5**. Uncoated spots served as controls. ■, mean; □, median.

a)

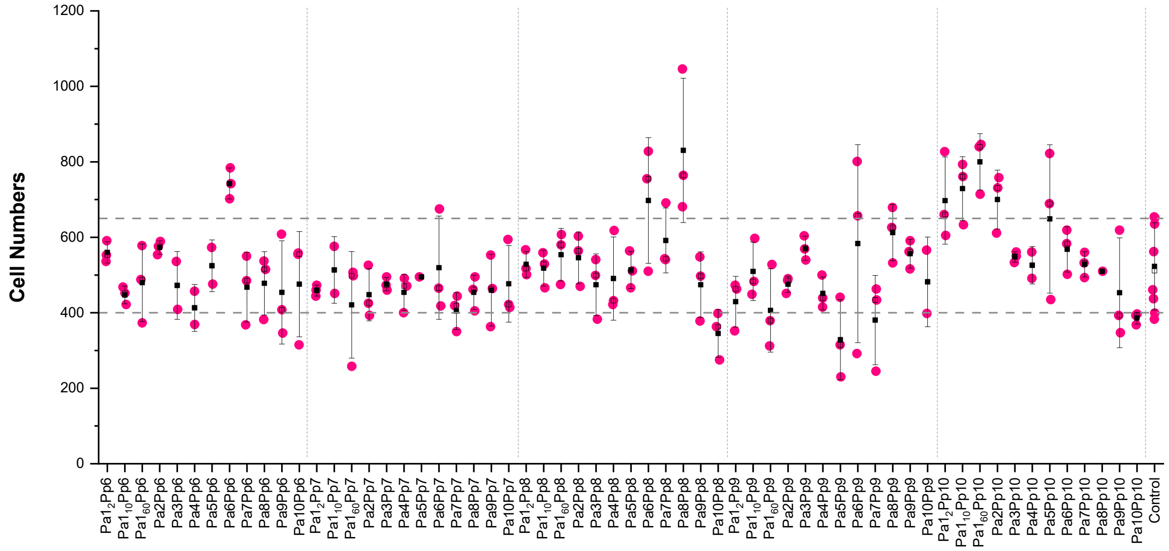

b)

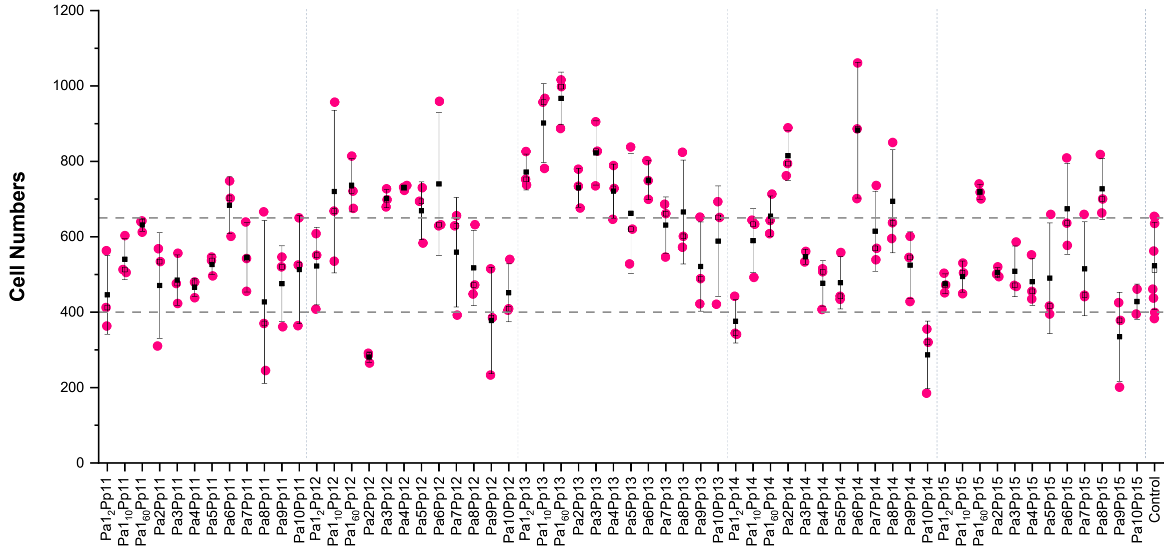

Figure S91. Numbers of Hoechst-stained nuclei of HeLa-RFP cells cultured on **PaPp** coatings prepared from **Pa1$_2$-Pa10** crosslinked with a) **Pp6-Pp10** and b) **Pp11-Pp15**. Uncoated spots served as controls. ■, mean; □, median.

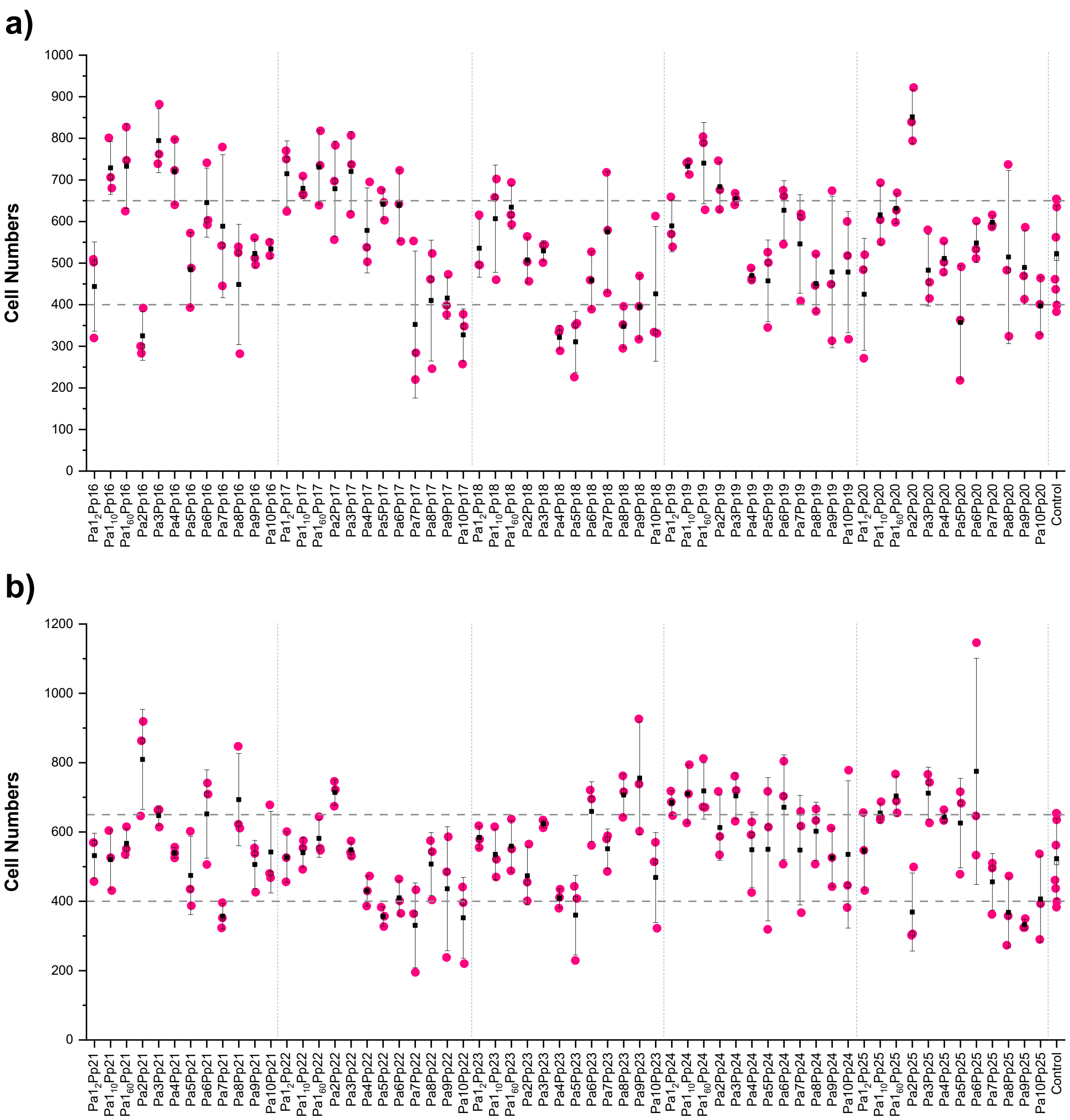

Figure S92. Numbers of Hoechst-stained nuclei of HeLa-RFP cells cultured on **PaPp** coatings prepared from **Pa1$_2$-Pa10** crosslinked with a) **Pp16-Pp20** and b) **Pp21-Pp25**. Uncoated spots served as controls. ■, mean; □, median.

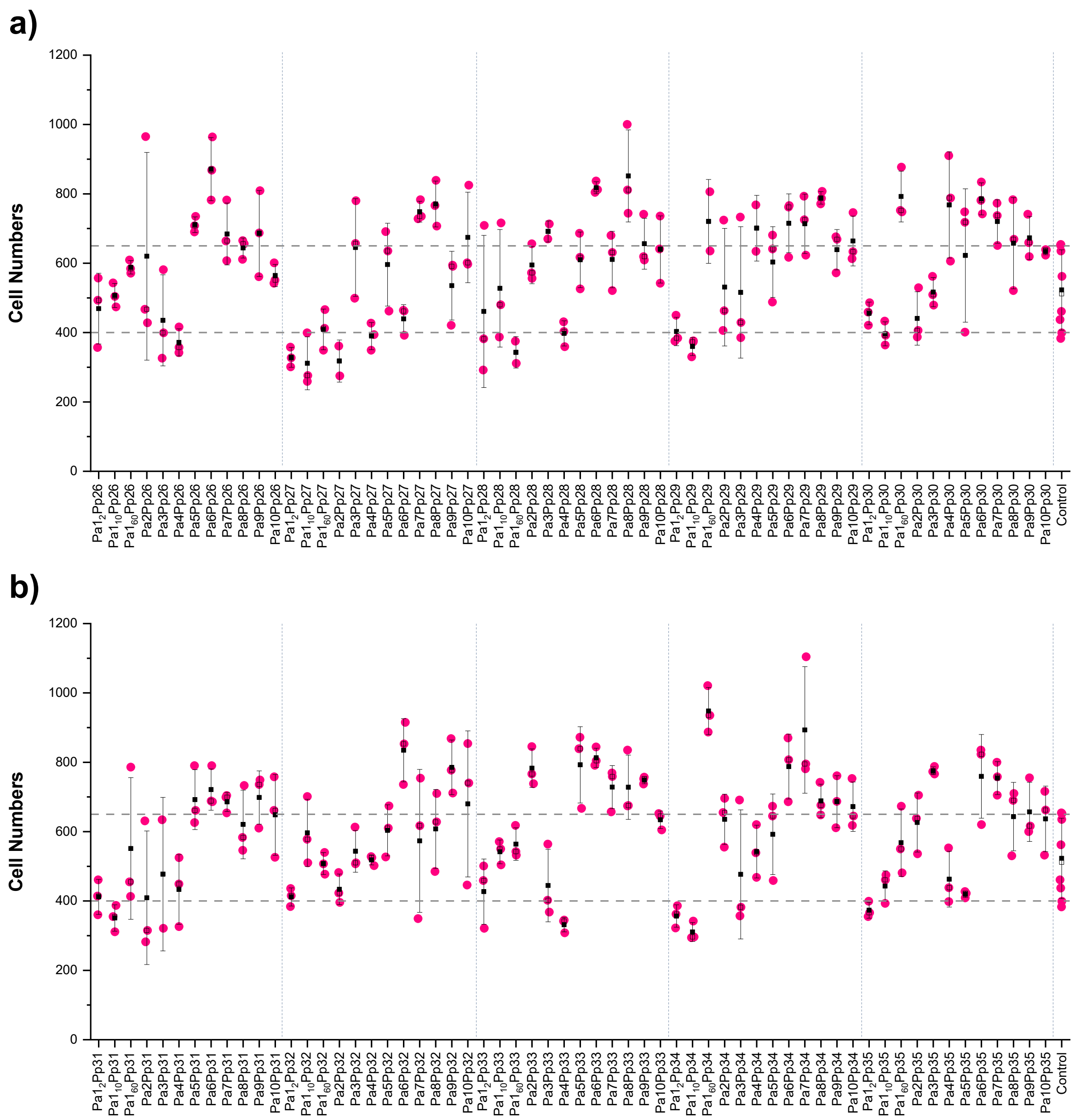

Figure S93. Numbers of Hoechst-stained nuclei of HeLa-RFP cells cultured on **PaPp** coatings prepared from **Pa1$_2$-Pa10** crosslinked with a) **Pp26-Pp30** and b) **Pp31-Pp35**. Uncoated spots served as controls. ■, mean; □, median.

a)

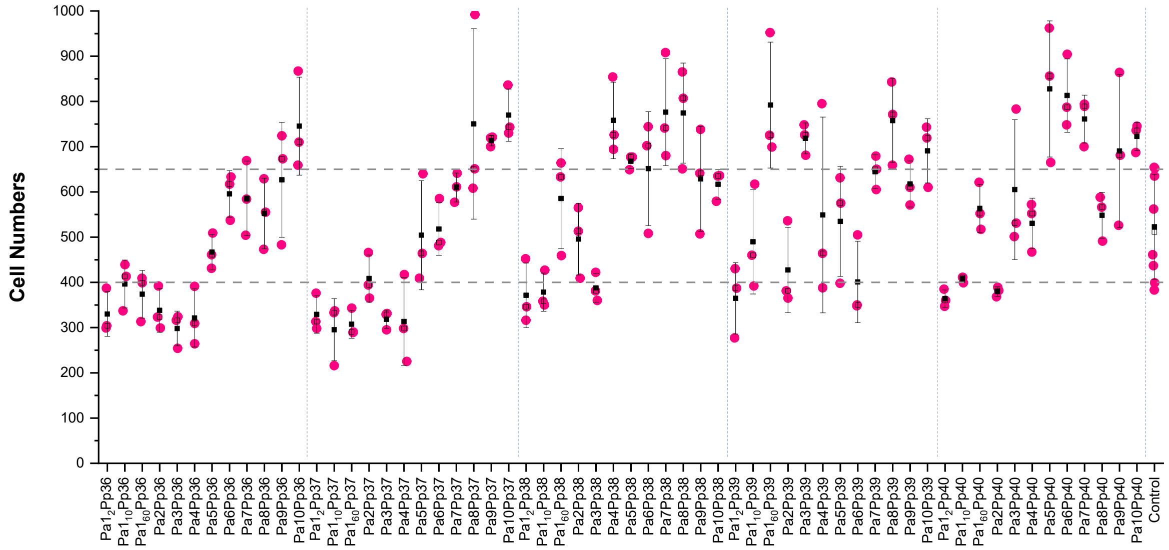

b)

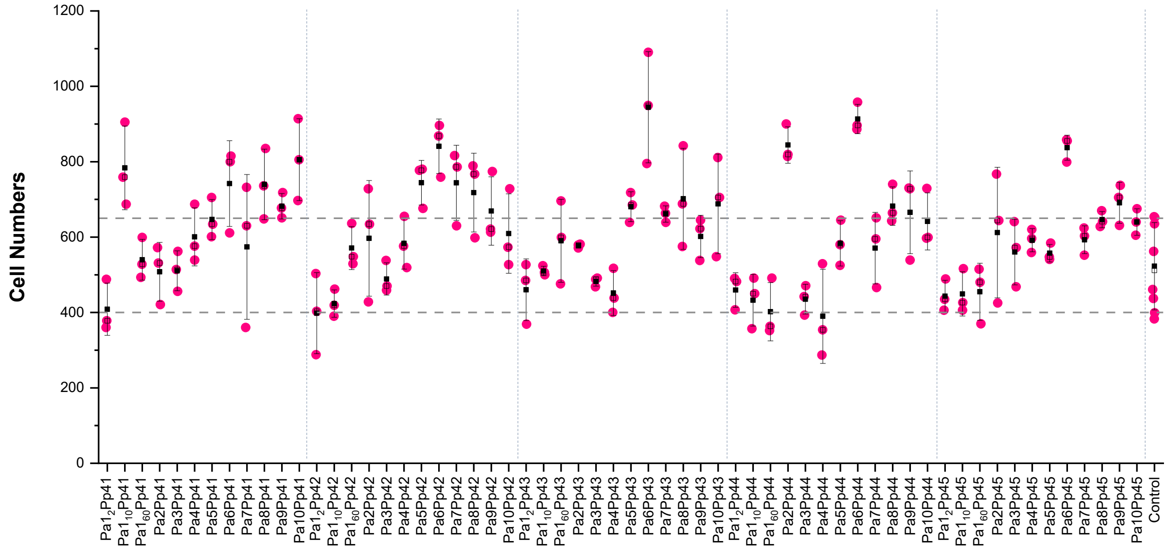

Figure S94. Numbers of Hoechst-stained nuclei of HeLa-RFP cells cultured on **PaPp** coatings prepared from **Pa1$_2$-Pa10** crosslinked with a) **Pp36-Pp40** and b) **Pp41-Pp45**. Uncoated spots served as controls. ■, mean; □, median.

a)

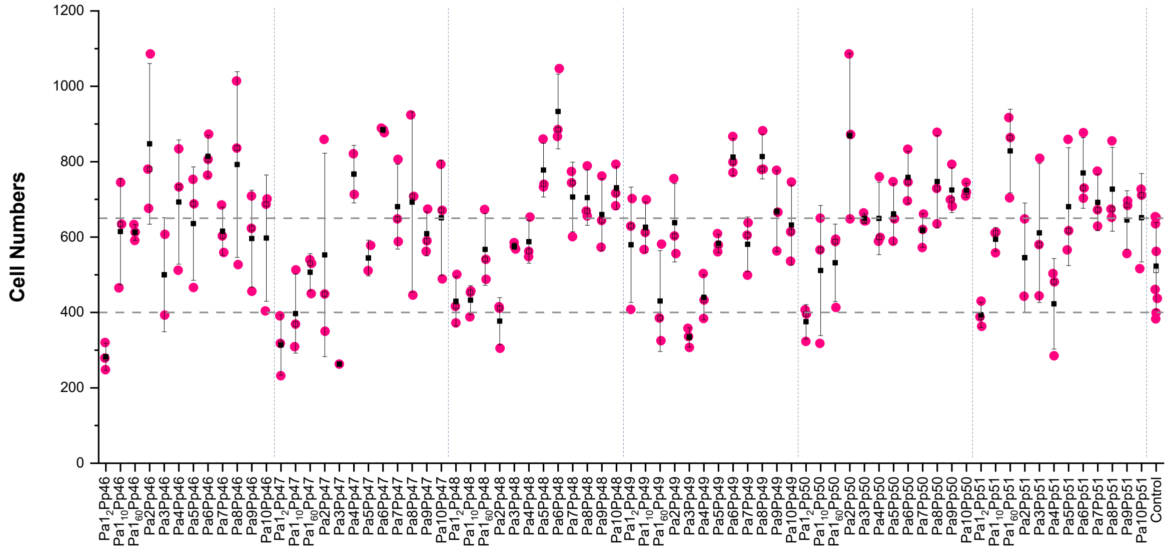

Figure S95. Numbers of Hoechst-stained nuclei of HeLa-RFP cells cultured on **PaPp** coatings prepared from **Pa1$_2$-Pa10** crosslinked with **Pp46-Pp51**. Uncoated spots served as controls. ■, mean; □, median.

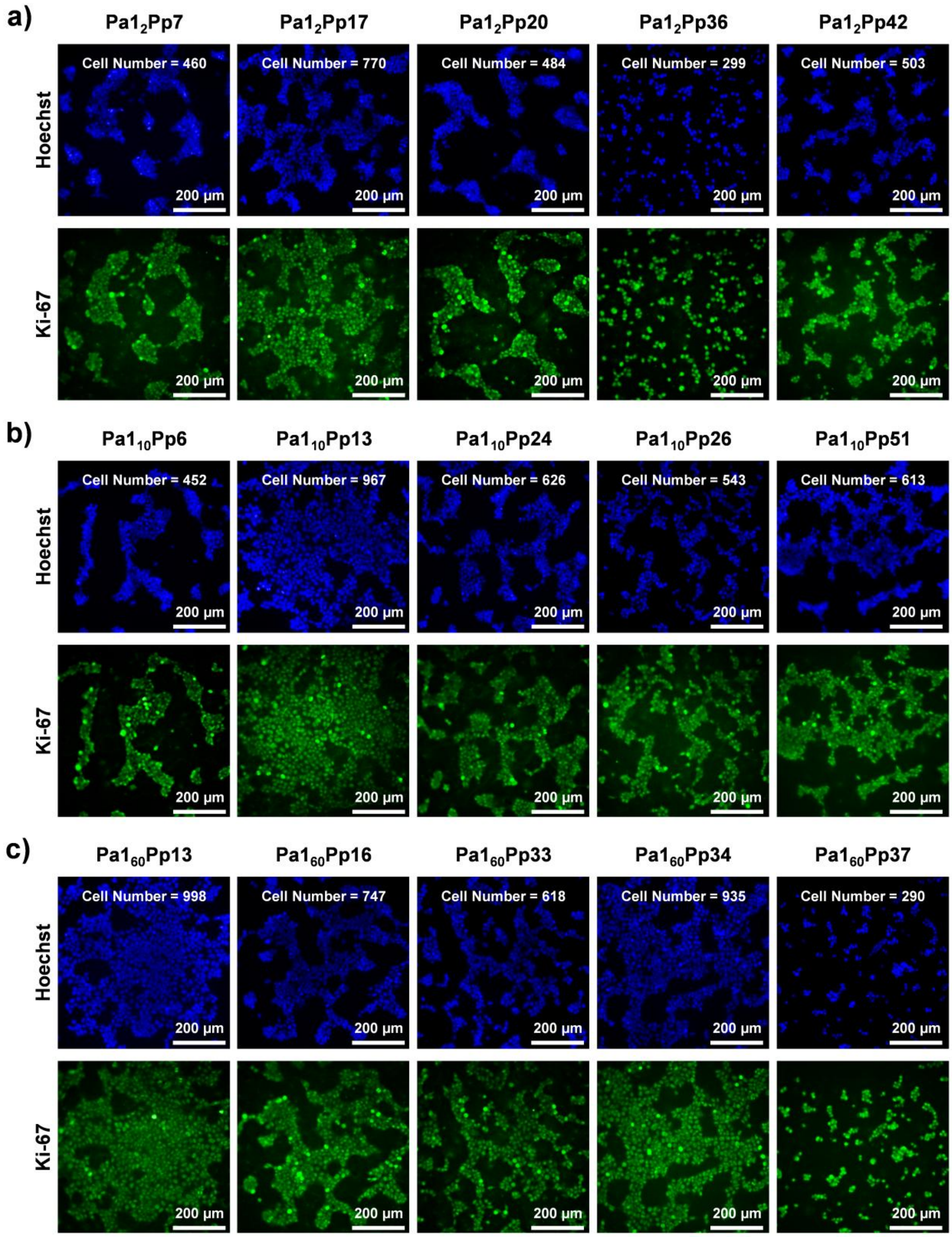

Figure S96. Hoechst- and Ki-67-stained nuclei of HeLa-RFP cells cultured on **PaPp** coatings prepared from a) **Pa1$_2$**, b) **Pa1$_{10}$**, and c) **Pa1$_{60}$**, each crosslinked with randomly selected **Pp**.

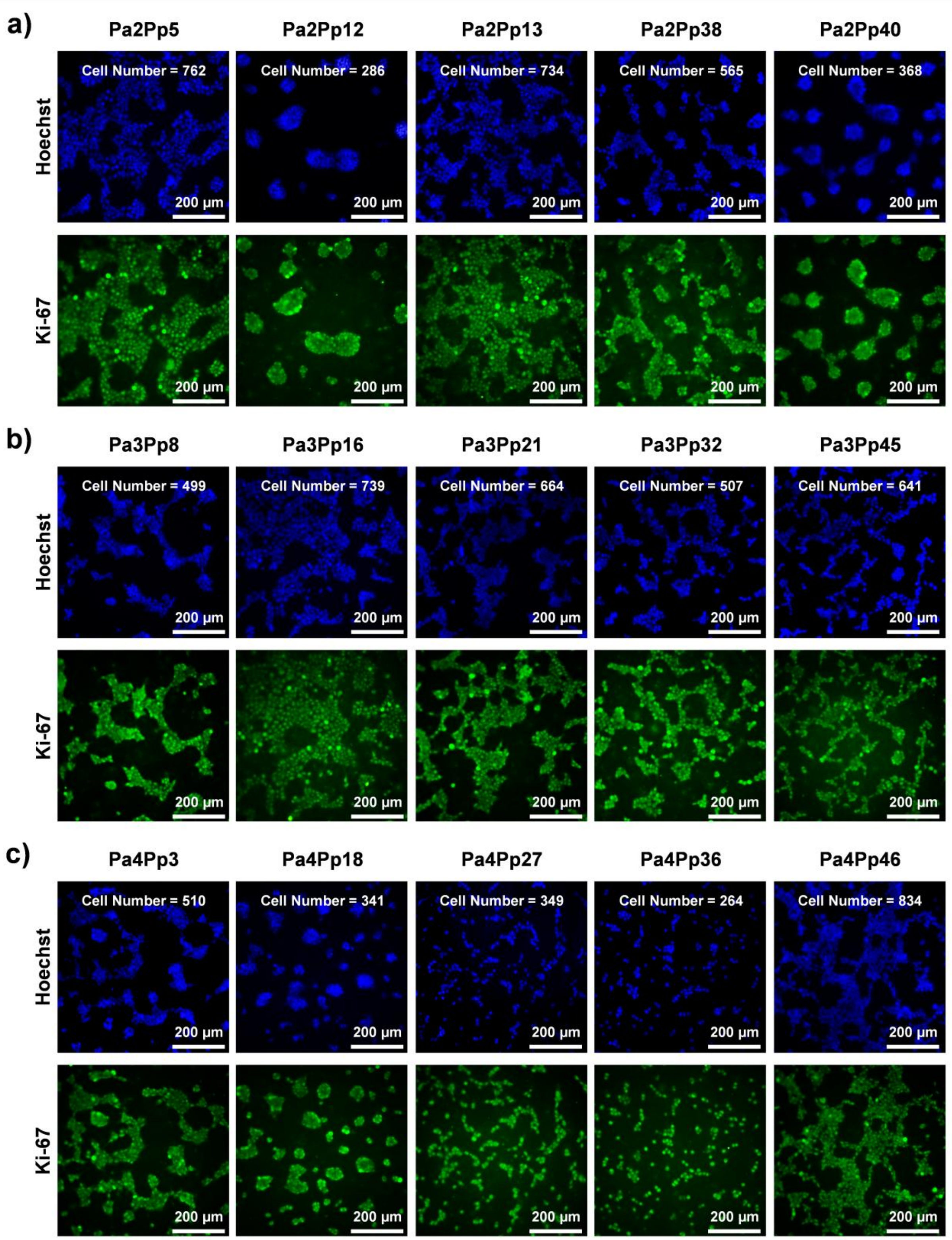

Figure S97. Hoechst- and Ki-67-stained nuclei of HeLa-RFP cells cultured on **PaPp** coatings prepared from a) **Pa2**, b) **Pa3**, and c) **Pa4**, each crosslinked with randomly selected **Pp**.

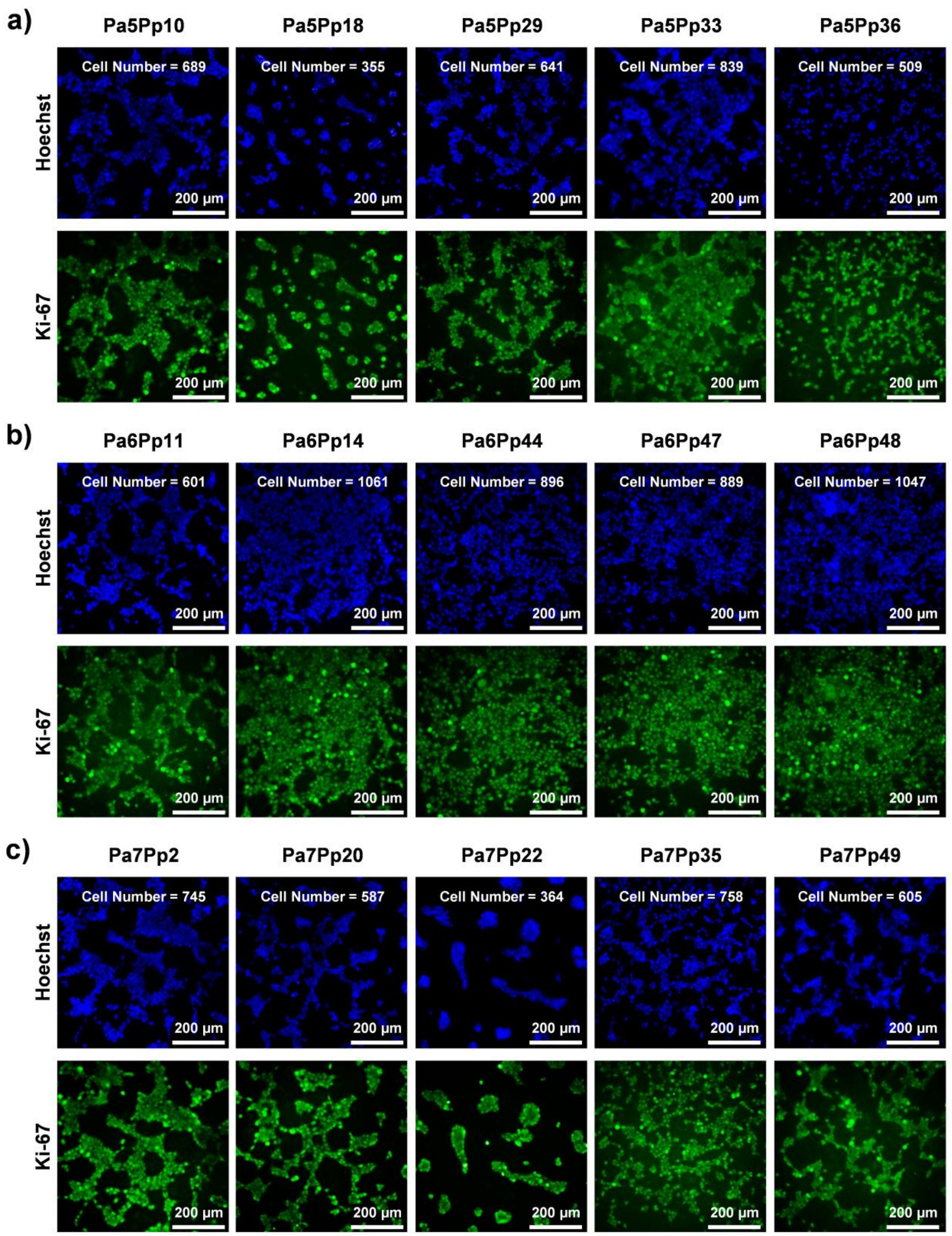

Figure S98. Hoechst- and Ki-67-stained nuclei of HeLa-RFP cells cultured on **PaPp** coatings prepared from a) **Pa5**, b) **Pa6**, and c) **Pa7**, each crosslinked with randomly selected **Pp**.

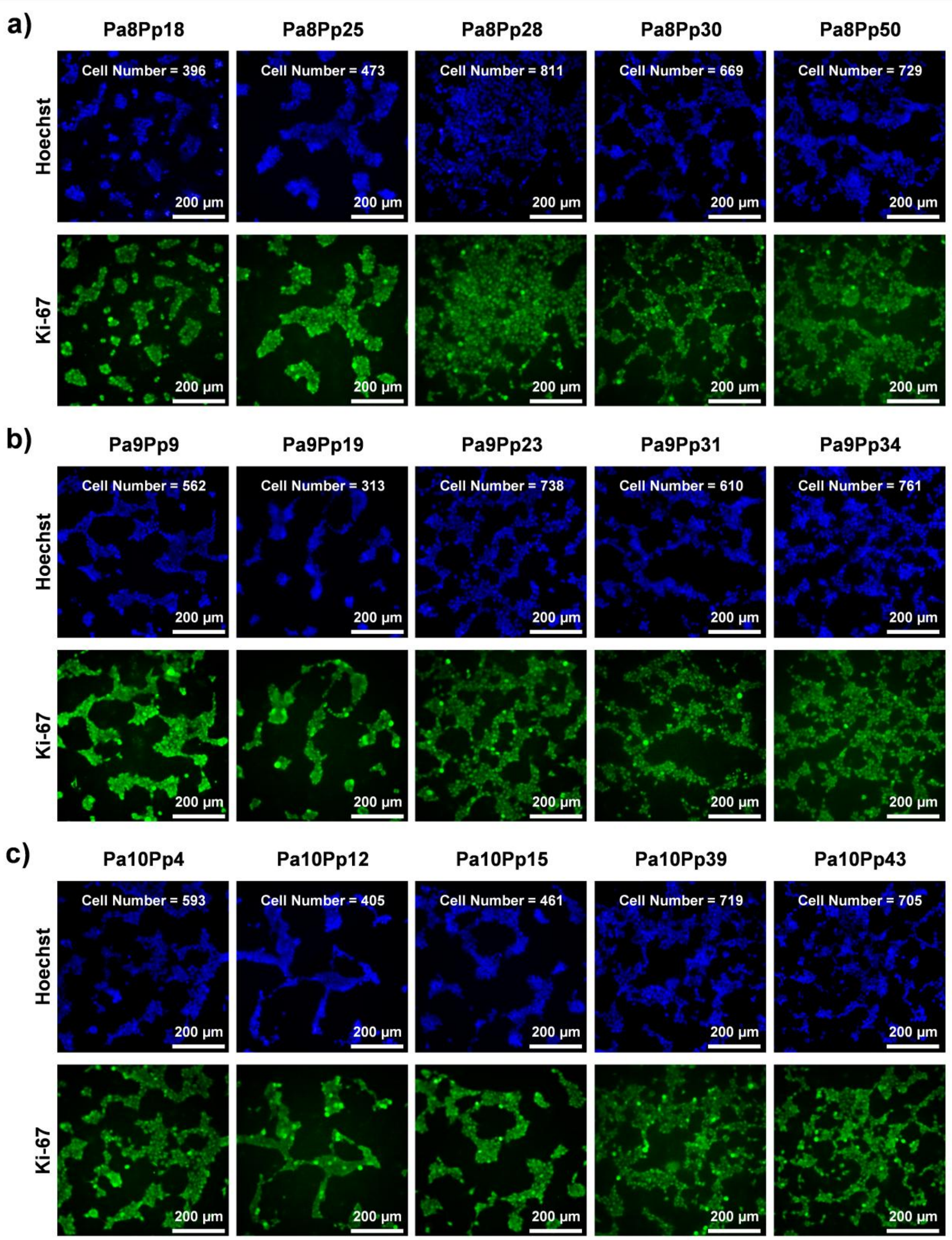

Figure S99. Hoechst- and Ki-67-stained nuclei of HeLa-RFP cells cultured on **PaPp** coatings prepared from a) **Pa8**, b) **Pa9**, and c) **Pa10**, each crosslinked with randomly selected **Pp**.

9. Summary of UHT Stability and Functionality Screening Results

Table S16. Summary of UHT stability and functionality screening results for **PaPp** coatings. Green indicates a positive outcome, and red indicates a negative outcome. Data are shown for both precursor **Pa** and precursor **Pp**.

Coatings	Stability (CNO^-)[a]	Stability (CN^-)[b]	Green Fluorescence[c]	Blue Fluorescence[d]	Red Fluorescence[e]	Metal-Reducing Activity[f]	Antibacterial Activity[g]	Compatibility with Human Cells[h]
$Pa1_2$	Green	Green	Red	Red	Red	Red	Red	Green
$Pa1_{10}$	Green	Green	Red	Red	Red	Red	Red	Green
$Pa1_{60}$	Green	Green	Red	Red	Red	Red	Red	Green
Pa2	Green	Green	Red	Red	Red	Red	Red	Green
Pa3	Green	Green	Green	Green	Red	Red	Red	Green
Pa4	Green	Green	Green	Green	Green	Red	Red	Green
Pa5	Green	Green	Green	Green	Red	Red	Red	Green
Pa6	Green	Green	Red	Red	Red	Red	Red	Green
Pa7	Red	Red	Red	Red	Red	Red	Red	Green
Pa8	Red	Red	Red	Red	Red	Red	Red	Green
Pa9	Red	Green	Red	Red	Red	Red	Red	Green
Pa10	Red	Red	Red	Red	Red	Red	Red	Green
Pp1	Red	Red	Red	Red	Red	Red	Red	Green
Pp2	Red	Red	Red	Red	Red	Red	Red	Green
Pp3	Red	Red	Red	Red	Red	Red	Red	Green
Pp4	Red	Red	Red	Red	Red	Red	Red	Green
Pp5	Red	Red	Red	Red	Red	Red	Red	Green
Pp6	Red	Red	Red	Red	Red	Red	Red	Green
Pp7	Red	Red	Red	Red	Red	Red	Red	Green
Pp8	Red	Red	Red	Red	Red	Red	Red	Green
Pp9	Red	Red	Red	Red	Red	Red	Red	Green
Pp10	Red	Red	Red	Red	Red	Red	Red	Green
Pp11	Red	Red	Red	Red	Red	Red	Red	Green
Pp12	Green	Red	Red	Red	Red	Red	Red	Green
Pp13	Red	Red	Red	Red	Red	Red	Red	Green
Pp14	Red	Red	Red	Red	Red	Red	Red	Green
Pp15	Red	Red	Red	Red	Red	Red	Red	Green
Pp16	Red	Red	Red	Red	Red	Red	Red	Green
Pp17	Red	Red	Red	Red	Red	Red	Red	Green
Pp18	Red	Red	Red	Red	Red	Red	Red	Green
Pp19	Red	Red	Red	Red	Red	Red	Red	Green
Pp20	Red	Red	Red	Red	Red	Red	Red	Green
Pp21	Red	Red	Red	Red	Red	Red	Red	Green
Pp22	Red	Red	Red	Red	Red	Red	Red	Green
Pp23	Red	Red	Red	Red	Red	Red	Red	Green
Pp24	Red	Red	Red	Red	Red	Red	Red	Green
Pp25	Green	Green	Red	Red	Red	Red	Red	Green
Pp26	Red	Red	Red	Red	Red	Red	Red	Green
Pp27	Red	Red	Red	Red	Red	Red	Red	Green
Pp28	Red	Red	Red	Red	Red	Red	Red	Green
Pp29	Red	Red	Red	Red	Red	Red	Red	Green
Pp30	Red	Red	Red	Red	Red	Red	Red	Green
Pp31	Red	Red	Red	Red	Red	Red	Red	Green
Pp32	Red	Red	Red	Red	Red	Red	Red	Green
Pp33	Green	Red	Red	Red	Red	Red	Red	Green
Pp34	Red	Red	Red	Red	Red	Red	Red	Green
Pp35	Red	Red	Red	Red	Red	Red	Red	Green
Pp36	Red	Red	Red	Red	Red	Red	Red	Green
Pp37*	Red	Red	Red	Red	Red	Red	Red	Green
Pp38*	Green	Green	Red	Red	Red	Red	Red	Green
Pp39	Red	Red	Red	Red	Red	Red	Red	Green
Pp40	Red	Red	Red	Red	Red	Red	Red	Green
Pp41	Red	Red	Red	Red	Red	Red	Red	Green
Pp42	Red	Red	Red	Red	Red	Red	Red	Green
Pp43*	Red	Red	Red	Red	Red	Red	Red	Green
Pp44*	Red	Red	Red	Red	Red	Red	Red	Green
Pp45*	Red	Red	Red	Red	Red	Red	Red	Green
Pp46*	Red	Green	Red	Red	Red	Red	Red	Green
Pp47	Red	Green	Red	Red	Red	Red	Red	Green
Pp48	Red	Red	Red	Red	Red	Red	Red	Green
Pp49*	Red	Green	Red	Red	Red	Red	Red	Green
Pp50*	Red	Green	Red	Red	Red	Red	Red	Green
Pp51	Red	Green	Red	Red	Red	Red	Red	Green

[a,b]Ion count intensities of CNO^- (a) and CN^- (b) measured by ToF-SIMS. Measurements were performed on ITO-coated DMAs, except for coatings marked with an asterisk (*), which were measured on standard DMAs. Thresholds were set at >25,100 (CNO^-) and >44,100 (CN^-) for ITO-DMAs, and >1,700 (CNO^-) and >2,500 (CN^-) for standard DMAs. [c]Threshold set at median >1,500. [d]Threshold set at median >225. [e]Threshold set at median >225. [f]Threshold set at median >0.15. [g]Threshold set at median <0.7. [h]Criterion: Ki-67 positive.

Table S17. Summary of UHT stability and functionality screening results for **PaPp** coatings. Green indicates a positive outcome, and red indicates a negative outcome. Data are shown for **PaPp** coatings formed by crosslinking **Pa1$_2$-Pa10** with **Pp1-Pp5**, respectively.

Coatings	Stability (CNO^-)[a]	Stability (CN^-)[b]	Green Fluorescence[c]	Blue Fluorescence[d]	Red Fluorescence[e]	Metal-Reducing Activity[f]	Antibacterial Activity[g]	Compatibility with Human Cells[h]
Pa1$_2$Pp1								
Pa1$_{10}$Pp1								
Pa1$_{60}$Pp1								
Pa2Pp1								
Pa3Pp1								
Pa4Pp1								
Pa5Pp1								
Pa6Pp1								
Pa7Pp1								
Pa8Pp1								
Pa9Pp1								
Pa10Pp1								
Pa1$_2$Pp2								
Pa1$_{10}$Pp2								
Pa1$_{60}$Pp2								
Pa2Pp2								
Pa3Pp2								
Pa4Pp2								
Pa5Pp2								
Pa6Pp2								
Pa7Pp2								
Pa8Pp2								
Pa9Pp2								
Pa10Pp2								
Pa1$_2$Pp3								
Pa1$_{10}$Pp3								
Pa1$_{60}$Pp3								
Pa2Pp3								
Pa3Pp3								
Pa4Pp3								
Pa5Pp3								
Pa6Pp3								
Pa7Pp3								
Pa8Pp3								
Pa9Pp3								
Pa10Pp3								
Pa1$_2$Pp4								
Pa1$_{10}$Pp4								
Pa1$_{60}$Pp4								
Pa2Pp4								
Pa3Pp4								
Pa4Pp4								
Pa5Pp4								
Pa6Pp4								
Pa7Pp4								
Pa8Pp4								
Pa9Pp4								
Pa10Pp4								
Pa1$_2$Pp5								
Pa1$_{10}$Pp5								
Pa1$_{60}$Pp5								
Pa2Pp5								
Pa3Pp5								
Pa4Pp5								
Pa5Pp5								
Pa6Pp5								
Pa7Pp5								
Pa8Pp5								
Pa9Pp5								
Pa10Pp5								

[a,b]Ion count intensities of CNO^- (a) and CN^- (b) measured by ToF-SIMS. Measurements were performed on ITO-coated DMAs, except for coatings marked with an asterisk (*), which were measured on standard DMAs. Thresholds were set at >25,100 (CNO^-) and >44,100 (CN^-) for ITO-DMAs, and >1,700 (CNO^-) and >2,500 (CN^-) for standard DMAs. [c]Threshold set at median >1,500. [d]Threshold set at median >225. [e]Threshold set at median >225. [f]Threshold set at median >0.15. [g]Threshold set at median <0.7. [h]Criterion: Ki-67 positive.

Table S18. Summary of UHT stability and functionality screening results for **PaPp** coatings. Green indicates a positive outcome, and red indicates a negative outcome. Data are shown for **PaPp** coatings formed by crosslinking **Pa1$_2$-Pa10** with **Pp6-Pp10**, respectively.

Coatings	Stability (CNO^-)[a]	Stability (CN^-)[b]	Green Fluorescence[c]	Blue Fluorescence[d]	Red Fluorescence[e]	Metal-Reducing Activity[f]	Antibacterial Activity[g]	Compatibility with Human Cells[h]
Pa1$_2$Pp6	Green	Green	Green	Red	Red	Green	Red	Green
Pa1$_{10}$Pp6	Green	Green	Red	Red	Red	Red	Red	Green
Pa1$_{60}$Pp6	Green	Green	Green	Red	Red	Red	Red	Green
Pa2Pp6	Green	Green	Green	Green	Green	Green	Green	Green
Pa3Pp6	Green	Green	Green	Red	Red	Red	Red	Green
Pa4Pp6	Green	Green	Green	Green	Red	Green	Red	Green
Pa5Pp6	Green	Green	Green	Green	Red	Red	Red	Green
Pa6Pp6	Green	Green	Green	Green	Red	Green	Red	Green
Pa7Pp6	Red	Red	Red	Red	Red	Red	Red	Green
Pa8Pp6	Green	Green	Green	Green	Green	Green	Red	Green
Pa9Pp6	Green	Green	Green	Green	Green	Green	Red	Green
Pa10Pp6	Green	Green	Green	Red	Green	Green	Red	Green
Pa1$_2$Pp7	Green	Green	Green	Red	Red	Green	Red	Green
Pa1$_{10}$Pp7	Green	Green	Red	Red	Red	Green	Red	Green
Pa1$_{60}$Pp7	Green	Green	Green	Red	Red	Green	Red	Green
Pa2Pp7	Green	Green	Green	Green	Green	Green	Red	Green
Pa3Pp7	Green	Green	Green	Red	Red	Green	Red	Green
Pa4Pp7	Green	Green	Green	Red	Green	Green	Red	Green
Pa5Pp7	Green	Green	Green	Green	Green	Red	Red	Green
Pa6Pp7	Green	Green	Green	Green	Green	Green	Red	Green
Pa7Pp7	Red	Red	Red	Red	Red	Red	Red	Green
Pa8Pp7	Green	Green	Green	Green	Green	Green	Red	Green
Pa9Pp7*	Green	Green	Green	Green	Green	Green	Red	Green
Pa10Pp7*	Green	Green	Green	Red	Green	Green	Red	Green
Pa1$_2$Pp8	Green	Green	Red	Red	Red	Red	Red	Green
Pa1$_{10}$Pp8	Green	Green	Red	Red	Red	Red	Red	Green
Pa1$_{60}$Pp8	Green	Green	Red	Red	Red	Green	Red	Green
Pa2Pp8	Green	Green	Green	Red	Red	Green	Red	Green
Pa3Pp8	Green	Green	Green	Red	Red	Red	Red	Green
Pa4Pp8	Green	Green	Green	Red	Red	Green	Red	Green
Pa5Pp8	Green	Green	Green	Green	Red	Green	Red	Green
Pa6Pp8	Green	Green	Green	Green	Green	Green	Red	Green
Pa7Pp8	Red	Red	Red	Red	Red	Red	Red	Green
Pa8Pp8	Green	Green	Green	Green	Green	Green	Red	Green
Pa9Pp8*	Green	Green	Green	Red	Red	Green	Red	Green
Pa10Pp8*	Green	Green	Red	Red	Green	Green	Red	Green
Pa1$_2$Pp9	Green	Green	Green	Red	Red	Green	Red	Green
Pa1$_{10}$Pp9	Green	Green	Red	Red	Red	Green	Red	Green
Pa1$_{60}$Pp9	Green	Green	Red	Red	Red	Green	Red	Green
Pa2Pp9	Green	Green	Green	Red	Green	Green	Red	Green
Pa3Pp9	Green	Green	Green	Red	Red	Green	Red	Green
Pa4Pp9	Green	Green	Green	Red	Green	Green	Red	Green
Pa5Pp9	Green	Green	Green	Green	Green	Green	Red	Green
Pa6Pp9	Green	Green	Green	Green	Green	Green	Red	Green
Pa7Pp9	Red	Red	Red	Red	Red	Red	Red	Green
Pa8Pp9	Green	Green	Green	Green	Green	Green	Red	Green
Pa9Pp9	Green	Green	Green	Red	Green	Green	Red	Green
Pa10Pp9	Green	Green	Red	Red	Green	Green	Red	Green
Pa1$_2$Pp10	Green	Green	Green	Red	Red	Red	Red	Green
Pa1$_{10}$Pp10	Green	Green	Green	Green	Red	Red	Red	Green
Pa1$_{60}$Pp10	Green	Green	Green	Green	Red	Red	Red	Green
Pa2Pp10	Green	Green	Green	Green	Red	Green	Red	Green
Pa3Pp10	Green	Green	Green	Green	Red	Red	Red	Green
Pa4Pp10	Green	Green	Green	Green	Red	Red	Red	Green
Pa5Pp10	Green	Green	Green	Green	Red	Red	Red	Green
Pa6Pp10	Green	Green	Green	Green	Green	Red	Red	Green
Pa7Pp10	Red	Red	Red	Red	Red	Red	Red	Green
Pa8Pp10	Green	Green	Green	Green	Red	Red	Red	Green
Pa9Pp10	Green	Green	Red	Red	Red	Red	Red	Green
Pa10Pp10	Green	Green	Green	Red	Red	Red	Red	Green

[a,b]Ion count intensities of CNO^- (a) and CN^- (b) measured by ToF-SIMS. Measurements were performed on ITO-coated DMAs, except for coatings marked with an asterisk (*), which were measured on standard DMAs. Thresholds were set at >25,100 (CNO^-) and >44,100 (CN^-) for ITO-DMAs, and >1,700 (CNO^-) and >2,500 (CN^-) for standard DMAs. [c]Threshold set at median >1,500. [d]Threshold set at median >225. [e]Threshold set at median >225. [f]Threshold set at median >0.15. [g]Threshold set at median <0.7. [h]Criterion: Ki-67 positive.

Table S19. Summary of UHT stability and functionality screening results for **PaPp** coatings. Green indicates a positive outcome, and red indicates a negative outcome. Data are shown for **PaPp** coatings formed by crosslinking **$Pa1_2$-Pa10** with **Pp11-Pp15**, respectively.

Coatings	Stability (CNO^-)[a]	Stability (CN^-)[b]	Green Fluorescence[c]	Blue Fluorescence[d]	Red Fluorescence[e]	Metal-Reducing Activity[f]	Antibacterial Activity[g]	Compatibility with Human Cells[h]
$Pa1_2Pp11$	Green	Green	Green	Green	Red	Red	Red	Green
$Pa1_{10}Pp11$	Green	Green	Green	Red	Red	Red	Red	Green
$Pa1_{60}Pp11$	Green	Green	Green	Green	Red	Red	Red	Green
Pa2Pp11	Green	Green	Green	Red	Red	Red	Red	Green
Pa3Pp11	Green	Green	Green	Green	Red	Red	Red	Green
Pa4Pp11	Green	Green	Green	Green	Red	Red	Red	Green
Pa5Pp11	Green	Green	Green	Green	Green	Red	Red	Green
Pa6Pp11	Green	Green	Green	Green	Red	Red	Red	Green
Pa7Pp11	Green	Green	Red	Red	Red	Red	Red	Green
Pa8Pp11	Green	Green	Green	Green	Red	Red	Red	Green
Pa9Pp11	Green	Green	Red	Red	Red	Red	Red	Green
Pa10Pp11	Green	Green	Green	Green	Red	Red	Red	Green
$Pa1_2Pp12$	Green	Green	Red	Red	Red	Red	Red	Green
$Pa1_{10}Pp12$	Green	Green	Red	Red	Red	Red	Red	Green
$Pa1_{60}Pp12$	Green	Green	Red	Red	Red	Red	Red	Green
Pa2Pp12	Green	Green	Red	Red	Red	Red	Red	Green
Pa3Pp12	Green	Green	Green	Red	Red	Red	Red	Green
Pa4Pp12	Green	Green	Green	Green	Red	Red	Red	Green
Pa5Pp12	Green	Green	Green	Green	Red	Red	Red	Green
Pa6Pp12	Green	Green	Red	Red	Red	Red	Red	Green
Pa7Pp12	Green	Green	Red	Red	Red	Red	Red	Green
Pa8Pp12	Green	Green	Red	Red	Red	Red	Red	Green
Pa9Pp12	Green	Green	Red	Red	Red	Red	Red	Green
Pa10Pp12	Green	Green	Red	Red	Red	Red	Red	Green
$Pa1_2Pp13$	Green	Green	Red	Red	Red	Red	Red	Green
$Pa1_{10}Pp13$	Green	Green	Red	Red	Red	Red	Red	Green
$Pa1_{60}Pp13$	Green	Green	Red	Red	Red	Red	Red	Green
Pa2Pp13	Green	Green	Red	Red	Red	Red	Red	Green
Pa3Pp13	Green	Green	Green	Green	Red	Red	Red	Green
Pa4Pp13	Green	Green	Green	Green	Green	Red	Red	Green
Pa5Pp13	Green	Green	Green	Green	Green	Red	Red	Green
Pa6Pp13	Green	Green	Green	Green	Red	Red	Red	Green
Pa7Pp13	Red	Red	Red	Red	Red	Red	Red	Green
Pa8Pp13	Red	Green	Green	Red	Red	Red	Red	Green
Pa9Pp13	Green	Green	Red	Red	Red	Red	Red	Green
Pa10Pp13	Green	Green	Green	Red	Red	Red	Red	Green
$Pa1_2Pp14$	Green	Green	Green	Red	Red	Green	Red	Green
$Pa1_{10}Pp14$	Green	Green	Red	Red	Red	Green	Red	Green
$Pa1_{60}Pp14$	Green	Green	Green	Red	Red	Red	Red	Green
Pa2Pp14	Green	Green	Green	Green	Green	Green	Red	Green
Pa3Pp14	Green	Green	Green	Red	Red	Red	Red	Green
Pa4Pp14	Green	Green	Green	Green	Red	Green	Red	Green
Pa5Pp14	Green	Green	Green	Green	Red	Red	Red	Green
Pa6Pp14	Green	Green	Green	Green	Green	Green	Red	Green
Pa7Pp14	Red	Red	Red	Red	Red	Red	Red	Green
Pa8Pp14	Green	Green	Green	Green	Red	Green	Red	Green
Pa9Pp14	Green	Green	Green	Green	Red	Red	Red	Green
Pa10Pp14	Green	Green	Green	Green	Red	Red	Red	Green
$Pa1_2Pp15$*	Green	Green	Red	Red	Red	Red	Red	Green
$Pa1_{10}Pp15$	Green	Green	Red	Red	Red	Red	Red	Green
$Pa1_{60}Pp15$	Green	Green	Green	Red	Red	Red	Red	Green
Pa2Pp15	Green	Green	Green	Red	Red	Green	Red	Green
Pa3Pp15	Green	Green	Green	Red	Red	Red	Red	Green
Pa4Pp15	Green	Green	Green	Green	Green	Red	Red	Green
Pa5Pp15	Green	Green	Green	Green	Green	Red	Red	Green
Pa6Pp15	Green	Green	Green	Green	Green	Green	Red	Green
Pa7Pp15	Red	Red	Red	Red	Red	Red	Red	Green
Pa8Pp15*	Green	Green	Green	Green	Green	Red	Red	Green
Pa9Pp15*	Green	Green	Red	Red	Red	Red	Red	Green
Pa10Pp15*	Green	Green	Red	Red	Red	Red	Red	Green

[a,b]Ion count intensities of CNO^- (a) and CN^- (b) measured by ToF-SIMS. Measurements were performed on ITO-coated DMAs, except for coatings marked with an asterisk (*), which were measured on standard DMAs. Thresholds were set at >25,100 (CNO^-) and >44,100 (CN^-) for ITO-DMAs, and >1,700 (CNO^-) and >2,500 (CN^-) for standard DMAs. [c]Threshold set at median >1,500. [d]Threshold set at median >225. [e]Threshold set at median >225. [f]Threshold set at median >0.15. [g]Threshold set at median <0.7. [h]Criterion: Ki-67 positive.

Table S20. Summary of UHT stability and functionality screening results for **PaPp** coatings. Green indicates a positive outcome, and red indicates a negative outcome. Data are shown for **PaPp** coatings formed by crosslinking **$Pa1_2$-Pa10** with **Pp16-Pp20**, respectively.

Coatings	Stability (CNO^-)[a]	Stability (CN^-)[b]	Green Fluorescence[c]	Blue Fluorescence[d]	Red Fluorescence[e]	Metal-Reducing Activity[f]	Antibacterial Activity[g]	Compatibility with Human Cells[h]
$Pa1_2$Pp16*	Green	Green	Red	Red	Red	Red	Red	Green
$Pa1_{10}$Pp16	Green	Green	Red	Red	Red	Red	Red	Green
$Pa1_{60}$Pp16	Green	Green	Red	Red	Red	Red	Red	Green
Pa2Pp16	Green	Green	Red	Red	Red	Red	Red	Green
Pa3Pp16	Green	Green	Green	Red	Red	Red	Red	Green
Pa4Pp16	Green	Green	Green	Red	Red	Red	Red	Green
Pa5Pp16	Green	Green	Green	Green	Red	Red	Red	Green
Pa6Pp16	Green	Green	Green	Green	Red	Red	Red	Green
Pa7Pp16	Red	Green	Red	Red	Red	Red	Red	Green
Pa8Pp16*	Green	Green	Red	Red	Red	Red	Red	Green
Pa9Pp16*	Green	Green	Red	Green	Red	Red	Red	Green
Pa10Pp16*	Green	Green	Red	Red	Red	Red	Red	Green
$Pa1_2$Pp17	Green	Green	Green	Red	Red	Red	Red	Green
$Pa1_{10}$Pp17	Green	Green	Red	Red	Red	Red	Red	Green
$Pa1_{60}$Pp17	Green	Green	Green	Red	Red	Red	Red	Green
Pa2Pp17	Green	Green	Green	Red	Red	Red	Red	Green
Pa3Pp17	Green	Green	Green	Red	Red	Red	Red	Green
Pa4Pp17	Green	Green	Green	Green	Red	Green	Red	Green
Pa5Pp17	Green	Green	Green	Green	Red	Red	Red	Green
Pa6Pp17	Green	Green	Green	Green	Green	Red	Red	Green
Pa7Pp17	Green	Green	Red	Red	Red	Red	Red	Green
Pa8Pp17	Green	Green	Red	Red	Red	Red	Red	Green
Pa9Pp17*	Green	Green	Red	Red	Red	Red	Red	Green
Pa10Pp17*	Green	Green	Green	Red	Red	Red	Red	Green
$Pa1_2$Pp18	Green	Green	Green	Red	Red	Green	Red	Green
$Pa1_{10}$Pp18	Green	Green	Red	Red	Red	Green	Red	Green
$Pa1_{60}$Pp18	Green	Green	Green	Red	Red	Green	Red	Green
Pa2Pp18	Green	Green	Green	Green	Green	Green	Red	Green
Pa3Pp18	Green	Green	Green	Red	Red	Green	Red	Green
Pa4Pp18	Green	Green	Green	Green	Red	Green	Red	Green
Pa5Pp18	Green	Green	Green	Green	Green	Green	Red	Green
Pa6Pp18	Green	Green	Green	Green	Green	Green	Red	Green
Pa7Pp18	Red	Red	Red	Red	Red	Red	Red	Green
Pa8Pp18	Green	Green	Green	Green	Red	Green	Green	Green
Pa9Pp18*	Green	Green	Green	Green	Red	Green	Green	Green
Pa10Pp18*	Green	Green	Green	Green	Green	Green	Red	Green
$Pa1_2$Pp19*	Green	Green	Red	Red	Red	Red	Red	Green
$Pa1_{10}$Pp19	Green	Green	Red	Red	Red	Red	Red	Green
$Pa1_{60}$Pp19	Green	Green	Red	Red	Red	Red	Red	Green
Pa2Pp19	Green	Green	Green	Green	Red	Green	Red	Green
Pa3Pp19	Green	Green	Green	Red	Red	Red	Red	Green
Pa4Pp19	Green	Green	Green	Green	Red	Green	Red	Green
Pa5Pp19	Green	Green	Green	Green	Red	Green	Red	Green
Pa6Pp19	Green	Green	Green	Green	Green	Green	Red	Green
Pa7Pp19	Red	Red	Red	Red	Red	Red	Red	Green
Pa8Pp19*	Green	Green	Green	Green	Red	Red	Red	Green
Pa9Pp19	Green	Green	Red	Red	Red	Red	Red	Green
Pa10Pp19	Green	Green	Green	Green	Red	Red	Red	Green
$Pa1_2$Pp20*	Green	Green	Red	Red	Red	Red	Red	Green
$Pa1_{10}$Pp20	Green	Green	Red	Red	Red	Red	Red	Green
$Pa1_{60}$Pp20	Green	Green	Red	Red	Red	Red	Red	Green
Pa2Pp20	Green	Green	Green	Green	Red	Green	Red	Green
Pa3Pp20	Green	Green	Red	Red	Red	Red	Red	Green
Pa4Pp20	Green	Green	Green	Red	Red	Green	Red	Green
Pa5Pp20	Green	Green	Green	Green	Red	Green	Red	Green
Pa6Pp20	Green	Green	Green	Green	Red	Green	Red	Green
Pa7Pp20	Red	Red	Red	Red	Red	Red	Red	Green
Pa8Pp20*	Green	Green	Green	Green	Red	Red	Red	Green
Pa9Pp20	Green	Green	Red	Red	Red	Red	Red	Green
Pa10Pp20	Green	Green	Green	Red	Red	Red	Red	Green

[a,b]Ion count intensities of CNO^- (a) and CN^- (b) measured by ToF-SIMS. Measurements were performed on ITO-coated DMAs, except for coatings marked with an asterisk (*), which were measured on standard DMAs. Thresholds were set at >25,100 (CNO^-) and >44,100 (CN^-) for ITO-DMAs, and >1,700 (CNO^-) and >2,500 (CN^-) for standard DMAs. [c]Threshold set at median >1,500. [d]Threshold set at median >225. [e]Threshold set at median >225. [f]Threshold set at median >0.15. [g]Threshold set at median <0.7. [h]Criterion: Ki-67 positive.

Table S21. Summary of UHT stability and functionality screening results for **PaPp** coatings. Green indicates a positive outcome, and red indicates a negative outcome. Data are shown for **PaPp** coatings formed by crosslinking **Pa1$_2$-Pa10** with **Pp21-Pp25**, respectively.

Coatings	Stability (CNO^-)[a]	Stability (CN^-)[b]	Green Fluorescence[c]	Blue Fluorescence[d]	Red Fluorescence[e]	Metal-Reducing Activity[f]	Antibacterial Activity[g]	Compatibility with Human Cells[h]
Pa1$_2$Pp21	Green	Green	Green	Red	Red	Green	Red	Green
Pa1$_{10}$Pp21	Green	Green	Red	Red	Red	Red	Red	Green
Pa1$_{60}$Pp21	Green	Green	Red	Red	Red	Red	Red	Green
Pa2Pp21	Green	Green	Green	Green	Red	Green	Red	Green
Pa3Pp21	Green	Green	Red	Red	Red	Red	Red	Green
Pa4Pp21	Green	Green	Green	Green	Red	Green	Red	Green
Pa5Pp21	Green	Green	Green	Green	Red	Green	Red	Green
Pa6Pp21	Green	Green	Green	Green	Red	Red	Red	Green
Pa7Pp21	Red	Red	Red	Red	Red	Red	Red	Green
Pa8Pp21	Green	Green	Green	Green	Red	Green	Red	Green
Pa9Pp21	Green	Green	Red	Red	Red	Green	Red	Green
Pa10Pp21	Green	Green	Green	Red	Red	Green	Red	Green
Pa1$_2$Pp22	Green	Green	Red	Red	Red	Green	Red	Green
Pa1$_{10}$Pp22	Green	Green	Red	Red	Red	Green	Red	Green
Pa1$_{60}$Pp22	Green	Green	Red	Red	Red	Green	Red	Green
Pa2Pp22	Green	Green	Green	Green	Green	Green	Red	Green
Pa3Pp22	Green	Green	Red	Red	Red	Red	Red	Green
Pa4Pp22	Green	Green	Green	Green	Red	Green	Red	Green
Pa5Pp22	Green	Green	Green	Green	Red	Green	Red	Green
Pa6Pp22	Green	Green	Green	Green	Red	Green	Red	Green
Pa7Pp22	Red	Red	Red	Red	Red	Red	Red	Green
Pa8Pp22	Green	Green	Green	Green	Green	Green	Red	Green
Pa9Pp22	Green	Green	Green	Red	Red	Red	Red	Green
Pa10Pp22	Green	Green	Green	Red	Red	Red	Red	Green
Pa1$_2$Pp23	Green	Green	Red	Red	Red	Green	Red	Green
Pa1$_{10}$Pp23	Green	Green	Red	Red	Red	Green	Red	Green
Pa1$_{60}$Pp23	Green	Green	Red	Red	Red	Green	Red	Green
Pa2Pp23	Green	Green	Green	Red	Green	Green	Red	Green
Pa3Pp23	Green	Green	Green	Red	Red	Green	Red	Green
Pa4Pp23	Green	Green	Green	Red	Green	Green	Red	Green
Pa5Pp23	Green	Green	Green	Green	Green	Green	Red	Green
Pa6Pp23	Green	Green	Green	Green	Green	Green	Red	Green
Pa7Pp23	Red	Red	Red	Red	Red	Red	Red	Green
Pa8Pp23	Green	Green	Green	Red	Green	Green	Red	Green
Pa9Pp23	Green	Green	Green	Red	Green	Green	Red	Green
Pa10Pp23	Green	Green	Red	Red	Red	Green	Red	Green
Pa1$_2$Pp24	Green	Green	Red	Red	Red	Red	Red	Green
Pa1$_{10}$Pp24	Green	Green	Red	Red	Red	Green	Red	Green
Pa1$_{60}$Pp24	Green	Green	Green	Red	Red	Green	Red	Green
Pa2Pp24	Green	Green	Green	Green	Red	Red	Red	Green
Pa3Pp24	Green	Green	Green	Red	Red	Red	Red	Green
Pa4Pp24	Green	Green	Green	Green	Red	Green	Red	Green
Pa5Pp24	Green	Green	Green	Green	Red	Red	Red	Green
Pa6Pp24	Green	Green	Green	Green	Green	Red	Red	Green
Pa7Pp24	Red	Red	Red	Red	Red	Red	Red	Green
Pa8Pp24	Green	Green	Green	Red	Red	Red	Red	Green
Pa9Pp24	Green	Green	Red	Red	Red	Red	Red	Green
Pa10Pp24	Green	Green	Green	Red	Red	Red	Red	Green
Pa1$_2$Pp25	Green	Green	Red	Red	Red	Red	Red	Green
Pa1$_{10}$Pp25	Green	Green	Red	Red	Red	Red	Red	Green
Pa1$_{60}$Pp25*	Green	Green	Green	Red	Red	Red	Red	Green
Pa2Pp25	Green	Green	Red	Red	Red	Red	Red	Green
Pa3Pp25*	Green	Green	Green	Red	Red	Red	Red	Green
Pa4Pp25	Green	Green	Green	Green	Red	Red	Red	Green
Pa5Pp25	Green	Green	Green	Green	Red	Red	Red	Green
Pa6Pp25*	Green	Green	Green	Green	Red	Red	Red	Green
Pa7Pp25*	Green	Green	Red	Red	Red	Red	Red	Green
Pa8Pp25	Green	Green	Red	Red	Red	Red	Red	Green
Pa9Pp25	Green	Green	Red	Red	Red	Red	Red	Green
Pa10Pp25	Green	Green	Green	Red	Red	Red	Red	Green

[a,b]Ion count intensities of CNO^- (a) and CN^- (b) measured by ToF-SIMS. Measurements were performed on ITO-coated DMAs, except for coatings marked with an asterisk (*), which were measured on standard DMAs. Thresholds were set at >25,100 (CNO^-) and >44,100 (CN^-) for ITO-DMAs, and >1,700 (CNO^-) and >2,500 (CN^-) for standard DMAs. [c]Threshold set at median >1,500. [d]Threshold set at median >225. [e]Threshold set at median >225. [f]Threshold set at median >0.15. [g]Threshold set at median <0.7. [h]Criterion: Ki-67 positive.

Table S22. Summary of UHT stability and functionality screening results for **PaPp** coatings. Green indicates a positive outcome, and red indicates a negative outcome. Data are shown for **PaPp** coatings formed by crosslinking **Pa1$_2$-Pa10** with **Pp26-Pp30**, respectively.

Coatings	Stability (CNO^-)[a]	Stability (CN^-)[b]	Green Fluorescence[c]	Blue Fluorescence[d]	Red Fluorescence[e]	Metal-Reducing Activity[f]	Antibacterial Activity[g]	Compatibility with Human Cells[h]
Pa1$_2$Pp26	Green	Green	Green	Red	Red	Red	Red	Green
Pa1$_{10}$Pp26	Green	Green	Red	Red	Red	Red	Red	Green
Pa1$_{60}$Pp26*	Green	Green	Green	Red	Red	Red	Red	Green
Pa2Pp26	Green	Green	Green	Green	Red	Red	Red	Green
Pa3Pp26*	Green	Green	Green	Green	Red	Red	Red	Green
Pa4Pp26	Green	Green	Green	Green	Red	Red	Red	Green
Pa5Pp26	Green	Green	Green	Green	Red	Red	Red	Green
Pa6Pp26*	Green	Green	Green	Red	Red	Red	Red	Green
Pa7Pp26*	Red	Green	Red	Red	Red	Red	Red	Green
Pa8Pp26	Green	Green	Green	Green	Red	Red	Red	Green
Pa9Pp26	Green	Green	Green	Green	Red	Green	Red	Green
Pa10Pp26	Green	Green	Green	Green	Red	Red	Red	Green
Pa1$_2$Pp27	Green	Green	Green	Red	Red	Red	Red	Green
Pa1$_{10}$Pp27	Green	Green	Red	Red	Red	Red	Red	Green
Pa1$_{60}$Pp27	Green	Green	Red	Red	Red	Red	Red	Green
Pa2Pp27	Green	Green	Green	Green	Red	Red	Red	Green
Pa3Pp27	Green	Green	Red	Red	Red	Red	Red	Green
Pa4Pp27	Green	Green	Green	Green	Green	Red	Red	Green
Pa5Pp27	Green	Green	Green	Green	Red	Red	Red	Green
Pa6Pp27*	Green	Green	Green	Green	Red	Red	Red	Green
Pa7Pp27*	Red	Red	Red	Red	Red	Red	Red	Green
Pa8Pp27	Green	Green	Green	Green	Red	Red	Red	Green
Pa9Pp27	Green	Green	Red	Red	Red	Red	Red	Green
Pa10Pp27	Green	Green	Green	Green	Red	Red	Red	Green
Pa1$_2$Pp28	Green	Green	Red	Red	Red	Green	Red	Green
Pa1$_{10}$Pp28	Green	Green	Red	Red	Red	Red	Red	Green
Pa1$_{60}$Pp28	Green	Green	Red	Red	Red	Red	Red	Green
Pa2Pp28	Green	Green	Green	Green	Green	Red	Red	Green
Pa3Pp28	Green	Green	Red	Red	Red	Red	Red	Green
Pa4Pp28	Green	Green	Green	Red	Green	Red	Red	Green
Pa5Pp28	Green	Green	Green	Red	Green	Red	Red	Green
Pa6Pp28*	Green	Green	Green	Red	Green	Red	Red	Green
Pa7Pp28*	Red	Green	Red	Red	Red	Red	Red	Green
Pa8Pp28	Green	Green	Green	Green	Green	Green	Red	Green
Pa9Pp28	Green	Green	Green	Red	Green	Green	Red	Green
Pa10Pp28	Green	Green	Green	Red	Green	Green	Red	Green
Pa1$_2$Pp29	Green	Green	Green	Red	Red	Green	Red	Green
Pa1$_{10}$Pp29	Green	Green	Green	Red	Red	Red	Red	Green
Pa1$_{60}$Pp29	Green	Green	Green	Red	Red	Red	Red	Green
Pa2Pp29	Green	Green	Green	Green	Red	Red	Red	Green
Pa3Pp29	Green	Green	Green	Red	Red	Green	Red	Green
Pa4Pp29	Green	Green	Green	Red	Red	Green	Red	Green
Pa5Pp29	Green	Green	Green	Green	Red	Red	Red	Green
Pa6Pp29*	Green	Green	Green	Green	Green	Green	Red	Green
Pa7Pp29*	Red	Green	Red	Red	Red	Red	Red	Green
Pa8Pp29	Green	Green	Green	Green	Red	Green	Red	Green
Pa9Pp29	Green	Green	Green	Red	Red	Green	Red	Green
Pa10Pp29	Green	Green	Green	Red	Red	Red	Red	Green
Pa1$_2$Pp30	Green	Green	Green	Red	Red	Red	Red	Green
Pa1$_{10}$Pp30	Green	Green	Red	Red	Red	Red	Red	Green
Pa1$_{60}$Pp30	Green	Green	Green	Red	Red	Red	Red	Green
Pa2Pp30	Green	Green	Green	Green	Red	Red	Red	Green
Pa3Pp30	Green	Green	Red	Red	Red	Red	Red	Green
Pa4Pp30	Green	Green	Green	Red	Red	Red	Red	Green
Pa5Pp30	Green	Green	Green	Red	Green	Red	Red	Green
Pa6Pp30*	Green	Green	Green	Red	Red	Red	Red	Green
Pa7Pp30*	Red	Green	Red	Red	Red	Red	Red	Green
Pa8Pp30	Green	Green	Red	Red	Red	Red	Red	Green
Pa9Pp30	Green	Green	Red	Red	Red	Red	Red	Green
Pa10Pp30	Green	Green	Green	Green	Red	Red	Red	Green

[a,b]Ion count intensities of CNO^- (a) and CN^- (b) measured by ToF-SIMS. Measurements were performed on ITO-coated DMAs, except for coatings marked with an asterisk (*), which were measured on standard DMAs. Thresholds were set at >25,100 (CNO^-) and >44,100 (CN^-) for ITO-DMAs, and >1,700 (CNO^-) and >2,500 (CN^-) for standard DMAs. [c]Threshold set at median >1,500. [d]Threshold set at median >225. [e]Threshold set at median >225. [f]Threshold set at median >0.15. [g]Threshold set at median <0.7. [h]Criterion: Ki-67 positive.

Table S23. Summary of UHT stability and functionality screening results for **PaPp** coatings. Green indicates a positive outcome, and red indicates a negative outcome. Data are shown for **PaPp** coatings formed by crosslinking **Pa1$_2$-Pa10** with **Pp31-Pp35**, respectively.

Coatings	Stability (CNO^-)[a]	Stability (CN^-)[b]	Green Fluorescence[c]	Blue Fluorescence[d]	Red Fluorescence[e]	Metal-Reducing Activity[f]	Antibacterial Activity[g]	Compatibility with Human Cells[h]
Pa1$_2$Pp31								
Pa1$_{10}$Pp31								
Pa1$_{60}$Pp31								
Pa2Pp31								
Pa3Pp31								
Pa4Pp31								
Pa5Pp31*								
Pa6Pp31								
Pa7Pp31								
Pa8Pp31								
Pa9Pp31								
Pa10Pp31								
Pa1$_2$Pp32								
Pa1$_{10}$Pp32								
Pa1$_{60}$Pp32								
Pa2Pp32								
Pa3Pp32								
Pa4Pp32								
Pa5Pp32*								
Pa6Pp32								
Pa7Pp32								
Pa8Pp32								
Pa9Pp32								
Pa10Pp32								
Pa1$_2$Pp33								
Pa1$_{10}$Pp33								
Pa1$_{60}$Pp33								
Pa2Pp33								
Pa3Pp33								
Pa4Pp33								
Pa5Pp33*								
Pa6Pp33								
Pa7Pp33								
Pa8Pp33								
Pa9Pp33								
Pa10Pp33								
Pa1$_2$Pp34								
Pa1$_{10}$Pp34								
Pa1$_{60}$Pp34								
Pa2Pp34								
Pa3Pp34								
Pa4Pp34								
Pa5Pp34*								
Pa6Pp34								
Pa7Pp34								
Pa8Pp34								
Pa9Pp34								
Pa10Pp34								
Pa1$_2$Pp35*								
Pa1$_{10}$Pp35								
Pa1$_{60}$Pp35								
Pa2Pp35								
Pa3Pp35								
Pa4Pp35								
Pa5Pp35								
Pa6Pp35								
Pa7Pp35								
Pa8Pp35*								
Pa9Pp35*								
Pa10Pp35*								

[a,b]Ion count intensities of CNO^- (a) and CN^- (b) measured by ToF-SIMS. Measurements were performed on ITO-coated DMAs, except for coatings marked with an asterisk (*), which were measured on standard DMAs. Thresholds were set at >25,100 (CNO^-) and >44,100 (CN^-) for ITO-DMAs, and >1,700 (CNO^-) and >2,500 (CN^-) for standard DMAs. [c]Threshold set at median >1,500. [d]Threshold set at median >225. [e]Threshold set at median >225. [f]Threshold set at median >0.15. [g]Threshold set at median <0.7. [h]Criterion: Ki-67 positive.

Table S24. Summary of UHT stability and functionality screening results for **PaPp** coatings. Green indicates a positive outcome, and red indicates a negative outcome. Data are shown for **PaPp** coatings formed by crosslinking **Pa1$_2$-Pa10** with **Pp36-Pp40**, respectively.

Coatings	Stability (CNO^-)[a]	Stability (CN^-)[b]	Green Fluorescence[c]	Blue Fluorescence[d]	Red Fluorescence[e]	Metal-Reducing Activity[f]	Antibacterial Activity[g]	Compatibility with Human Cells[h]
Pa1$_2$Pp36*	Green	Green	Green	Green	Red	Green	Red	Green
Pa1$_{10}$Pp36	Green	Green	Green	Red	Red	Red	Red	Green
Pa1$_{60}$Pp36	Green	Green	Green	Green	Red	Red	Red	Green
Pa2Pp36	Green	Green	Green	Green	Red	Green	Red	Green
Pa3Pp36	Green	Green	Green	Green	Red	Red	Red	Green
Pa4Pp36	Green	Green	Green	Green	Red	Green	Red	Green
Pa5Pp36	Green	Green	Green	Green	Red	Red	Red	Green
Pa6Pp36	Green	Green	Green	Green	Green	Green	Red	Green
Pa7Pp36	Red	Red	Red	Red	Red	Red	Red	Green
Pa8Pp36*	Green	Green	Green	Green	Red	Red	Red	Green
Pa9Pp36*	Green	Green	Green	Red	Red	Red	Red	Green
Pa10Pp36*	Green	Green	Green	Red	Red	Red	Red	Green
Pa1$_2$Pp37*	Green	Green	Red	Red	Red	Green	Red	Green
Pa1$_{10}$Pp37*	Green	Green	Red	Red	Red	Red	Red	Green
Pa1$_{60}$Pp37	Green	Green	Red	Red	Red	Red	Red	Green
Pa2Pp37	Green	Green	Green	Green	Red	Green	Red	Green
Pa3Pp37	Green	Green	Green	Red	Red	Red	Red	Green
Pa4Pp37	Green	Green	Green	Green	Red	Green	Red	Green
Pa5Pp37	Green	Green	Green	Green	Red	Green	Red	Green
Pa6Pp37	Green	Green	Green	Green	Green	Green	Red	Green
Pa7Pp37	Red	Red	Red	Red	Red	Red	Red	Green
Pa8Pp37*	Green	Green	Red	Red	Red	Red	Red	Green
Pa9Pp37*	Green	Green	Red	Red	Red	Red	Red	Green
Pa10Pp37*	Green	Green	Green	Red	Red	Red	Red	Green
Pa1$_2$Pp38*	Green	Green	Green	Red	Red	Red	Red	Green
Pa1$_{10}$Pp38*	Green	Green	Red	Red	Red	Red	Red	Green
Pa1$_{60}$Pp38	Green	Green	Green	Red	Red	Green	Red	Green
Pa2Pp38	Green	Green	Green	Green	Red	Green	Red	Green
Pa3Pp38	Green	Green	Green	Red	Red	Red	Red	Green
Pa4Pp38	Green	Green	Green	Green	Green	Green	Red	Green
Pa5Pp38	Green	Green	Green	Green	Green	Green	Red	Green
Pa6Pp38	Green	Green	Green	Red	Green	Red	Red	Green
Pa7Pp38	Red	Green	Red	Red	Red	Red	Red	Green
Pa8Pp38*	Green	Green	Green	Green	Green	Red	Red	Green
Pa9Pp38*	Green	Green	Green	Red	Red	Red	Red	Green
Pa10Pp38*	Green	Green	Green	Green	Red	Red	Red	Green
Pa1$_2$Pp39*	Green	Green	Green	Red	Red	Red	Red	Green
Pa1$_{10}$Pp39	Green	Green	Green	Red	Red	Red	Red	Green
Pa1$_{60}$Pp39	Green	Green	Green	Red	Red	Red	Red	Green
Pa2Pp39	Green	Green	Green	Green	Red	Red	Red	Green
Pa3Pp39	Green	Green	Green	Red	Red	Red	Red	Green
Pa4Pp39	Green	Green	Green	Green	Green	Green	Red	Green
Pa5Pp39	Green	Green	Green	Green	Green	Red	Red	Green
Pa6Pp39	Green	Green	Green	Green	Green	Red	Red	Green
Pa7Pp39	Red	Red	Red	Red	Red	Red	Red	Green
Pa8Pp39*	Green	Green	Green	Green	Green	Red	Red	Green
Pa9Pp39*	Green	Green	Green	Green	Green	Red	Red	Green
Pa10Pp39*	Green	Green	Green	Green	Green	Red	Red	Green
Pa1$_2$Pp40*	Green	Green	Red	Red	Red	Red	Red	Green
Pa1$_{10}$Pp40	Green	Green	Green	Red	Red	Red	Red	Green
Pa1$_{60}$Pp40	Green	Green	Green	Green	Red	Red	Red	Green
Pa2Pp40	Green	Green	Green	Green	Red	Red	Red	Green
Pa3Pp40	Green	Green	Green	Green	Red	Red	Red	Green
Pa4Pp40	Green	Green	Green	Green	Green	Green	Red	Green
Pa5Pp40	Green	Green	Green	Green	Green	Red	Red	Green
Pa6Pp40	Green	Green	Green	Green	Green	Red	Red	Green
Pa7Pp40	Red	Red	Red	Red	Red	Red	Red	Green
Pa8Pp40*	Green	Green	Green	Green	Red	Red	Red	Green
Pa9Pp40*	Green	Green	Green	Red	Red	Red	Red	Green
Pa10Pp40*	Green	Green	Green	Green	Red	Red	Red	Green

[a,b]Ion count intensities of CNO^- (a) and CN^- (b) measured by ToF-SIMS. Measurements were performed on ITO-coated DMAs, except for coatings marked with an asterisk (*), which were measured on standard DMAs. Thresholds were set at >25,100 (CNO^-) and >44,100 (CN^-) for ITO-DMAs, and >1,700 (CNO^-) and >2,500 (CN^-) for standard DMAs. [c]Threshold set at median >1,500. [d]Threshold set at median >225. [e]Threshold set at median >225. [f]Threshold set at median >0.15. [g]Threshold set at median <0.7. [h]Criterion: Ki-67 positive.

Table S25. Summary of UHT stability and functionality screening results for **PaPp** coatings. Green indicates a positive outcome, and red indicates a negative outcome. Data are shown for **PaPp** coatings formed by crosslinking **Pa1$_2$-Pa10** with **Pp41-Pp45**, respectively.

Coatings	Stability (CNO^-)[a]	Stability (CN^-)[b]	Green Fluorescence[c]	Blue Fluorescence[d]	Red Fluorescence[e]	Metal-Reducing Activity[f]	Antibacterial Activity[g]	Compatibility with Human Cells[h]
Pa1$_2$Pp41	Green	Green	Red	Red	Red	Red	Red	Green
Pa1$_{10}$Pp41	Green	Green	Red	Red	Red	Red	Red	Green
Pa1$_{60}$Pp41*	Green	Green	Green	Red	Red	Red	Red	Green
Pa2Pp41	Green	Green	Green	Green	Red	Red	Red	Green
Pa3Pp41*	Green	Green	Green	Red	Red	Red	Red	Green
Pa4Pp41	Green	Green	Green	Green	Green	Red	Red	Green
Pa5Pp41	Green	Green	Green	Green	Green	Red	Red	Green
Pa6Pp41	Green	Green	Green	Red	Red	Red	Red	Green
Pa7Pp41	Red	Red	Red	Red	Red	Red	Red	Green
Pa8Pp41	Green	Green	Green	Green	Red	Red	Red	Green
Pa9Pp41	Green	Green	Green	Green	Red	Red	Red	Green
Pa10Pp41	Green	Green	Green	Green	Red	Red	Red	Green
Pa1$_2$Pp42	Green	Green	Red	Red	Red	Red	Red	Green
Pa1$_{10}$Pp42	Green	Green	Green	Red	Red	Red	Red	Green
Pa1$_{60}$Pp42*	Green	Green	Green	Green	Red	Red	Red	Green
Pa2Pp42	Green	Green	Green	Green	Red	Red	Red	Green
Pa3Pp42*	Green	Green	Green	Green	Red	Red	Red	Green
Pa4Pp42	Green	Green	Green	Green	Green	Red	Red	Green
Pa5Pp42	Green	Green	Green	Green	Red	Red	Red	Green
Pa6Pp42	Green	Green	Green	Green	Red	Red	Red	Green
Pa7Pp42	Red	Red	Red	Red	Red	Red	Red	Green
Pa8Pp42	Green	Green	Green	Red	Red	Red	Red	Green
Pa9Pp42	Green	Green	Green	Red	Red	Red	Red	Green
Pa10Pp42	Green	Green	Green	Green	Red	Red	Red	Green
Pa1$_2$Pp43	Green	Green	Red	Red	Red	Green	Red	Green
Pa1$_{10}$Pp43*	Green	Green	Red	Red	Red	Green	Red	Green
Pa1$_{60}$Pp43*	Green	Green	Green	Red	Red	Green	Red	Green
Pa2Pp43	Green	Green	Green	Green	Red	Green	Red	Green
Pa3Pp43*	Green	Green	Red	Red	Red	Green	Red	Green
Pa4Pp43	Green	Green	Green	Green	Red	Green	Red	Green
Pa5Pp43	Green	Green	Green	Green	Red	Green	Red	Green
Pa6Pp43	Green	Green	Green	Green	Red	Green	Red	Green
Pa7Pp43	Red	Red	Red	Red	Red	Red	Red	Green
Pa8Pp43	Green	Green	Red	Red	Red	Green	Red	Green
Pa9Pp43	Green	Green	Red	Red	Red	Green	Red	Green
Pa10Pp43	Green	Green	Red	Red	Red	Green	Red	Green
Pa1$_2$Pp44	Green	Green	Red	Red	Red	Green	Red	Green
Pa1$_{10}$Pp44*	Green	Green	Green	Red	Red	Green	Red	Green
Pa1$_{60}$Pp44*	Green	Green	Green	Red	Red	Green	Red	Green
Pa2Pp44	Green	Green	Green	Green	Red	Green	Red	Green
Pa3Pp44*	Green	Green	Green	Green	Red	Green	Red	Green
Pa4Pp44	Green	Green	Green	Green	Red	Green	Red	Green
Pa5Pp44	Green	Green	Green	Green	Red	Green	Red	Green
Pa6Pp44	Green	Green	Green	Green	Red	Green	Red	Green
Pa7Pp44	Red	Red	Red	Red	Red	Red	Red	Green
Pa8Pp44	Green	Green	Green	Green	Red	Green	Red	Green
Pa9Pp44	Green	Green	Green	Green	Red	Green	Red	Green
Pa10Pp44	Green	Green	Green	Red	Red	Green	Red	Green
Pa1$_2$Pp45	Green	Green	Red	Red	Red	Green	Red	Green
Pa1$_{10}$Pp45*	Green	Green	Red	Red	Red	Green	Red	Green
Pa1$_{60}$Pp45*	Green	Green	Green	Red	Red	Green	Red	Green
Pa2Pp45	Green	Green	Green	Green	Red	Green	Red	Green
Pa3Pp45*	Green	Green	Green	Red	Red	Green	Red	Green
Pa4Pp45	Green	Green	Green	Green	Red	Green	Red	Green
Pa5Pp45	Green	Green	Green	Green	Red	Green	Red	Green
Pa6Pp45	Green	Green	Green	Green	Red	Red	Red	Green
Pa7Pp45	Red	Red	Red	Red	Red	Red	Red	Green
Pa8Pp45	Green	Green	Green	Green	Red	Green	Red	Green
Pa9Pp45	Green	Green	Green	Red	Red	Green	Red	Green
Pa10Pp45	Green	Green	Green	Red	Red	Green	Red	Green

[a,b]Ion count intensities of CNO^- (a) and CN^- (b) measured by ToF-SIMS. Measurements were performed on ITO-coated DMAs, except for coatings marked with an asterisk (*), which were measured on standard DMAs. Thresholds were set at >25,100 (CNO^-) and >44,100 (CN^-) for ITO-DMAs, and >1,700 (CNO^-) and >2,500 (CN^-) for standard DMAs. [c]Threshold set at median >1,500. [d]Threshold set at median >225. [e]Threshold set at median >225. [f]Threshold set at median >0.15. [g]Threshold set at median <0.7. [h]Criterion: Ki-67 positive.

Table S26. Summary of UHT stability and functionality screening results for **PaPp** coatings. Green indicates a positive outcome, and red indicates a negative outcome. Data are shown for **PaPp** coatings formed by crosslinking **Pa1$_2$-Pa10** with **Pp46-Pp50**, respectively.

Coatings	Stability (CNO$^-$)[a]	Stability (CN$^-$)[b]	Green Fluorescence[c]	Blue Fluorescence[d]	Red Fluorescence[e]	Metal-Reducing Activity[f]	Antibacterial Activity[g]	Compatibility with Human Cells[h]
Pa1$_{2}$Pp46								
Pa1$_{10}$Pp46*								
Pa1$_{60}$Pp46*								
Pa2Pp46								
Pa3Pp46*								
Pa4Pp46								
Pa5Pp46								
Pa6Pp46								
Pa7Pp46								
Pa8Pp46								
Pa9Pp46								
Pa10Pp46								
Pa1$_{2}$Pp47								
Pa1$_{10}$Pp47								
Pa1$_{60}$Pp47								
Pa2Pp47								
Pa3Pp47								
Pa4Pp47								
Pa5Pp47								
Pa6Pp47								
Pa7Pp47								
Pa8Pp47								
Pa9Pp47								
Pa10Pp47								
Pa1$_{2}$Pp48								
Pa1$_{10}$Pp48								
Pa1$_{60}$Pp48								
Pa2Pp48								
Pa3Pp48								
Pa4Pp48								
Pa5Pp48								
Pa6Pp48								
Pa7Pp48								
Pa8Pp48								
Pa9Pp48								
Pa10Pp48								
Pa1$_{2}$Pp49								
Pa1$_{10}$Pp49*								
Pa1$_{60}$Pp49								
Pa2Pp49								
Pa3Pp49								
Pa4Pp49								
Pa5Pp49								
Pa6Pp49								
Pa7Pp49								
Pa8Pp49								
Pa9Pp49								
Pa10Pp49								
Pa1$_{2}$Pp50								
Pa1$_{10}$Pp50*								
Pa1$_{60}$Pp50								
Pa2Pp50								
Pa3Pp50								
Pa4Pp50								
Pa5Pp50								
Pa6Pp50								
Pa7Pp50								
Pa8Pp50								
Pa9Pp50								
Pa10Pp50								

[a,b]Ion count intensities of CNO$^-$ (a) and CN$^-$ (b) measured by ToF-SIMS. Measurements were performed on ITO-coated DMAs, except for coatings marked with an asterisk (*), which were measured on standard DMAs. Thresholds were set at >25,100 (CNO$^-$) and >44,100 (CN$^-$) for ITO-DMAs, and >1,700 (CNO$^-$) and >2,500 (CN$^-$) for standard DMAs. [c]Threshold set at median >1,500. [d]Threshold set at median >225. [e]Threshold set at median >225. [f]Threshold set at median >0.15. [g]Threshold set at median <0.7. [h]Criterion: Ki-67 positive.

Table S27. Summary of UHT stability and functionality screening results for **PaPp** coatings. Green indicates a positive outcome, and red indicates a negative outcome. Data are shown for **PaPp** coatings formed by crosslinking **Pa1$_2$-Pa10** with **Pp51**, respectively.

Coatings	Stability (CNO^-)[a]	Stability (CN^-)[b]	Green Fluorescence[c]	Blue Fluorescence[d]	Red Fluorescence[e]	Metal-Reducing Activity[f]	Antibacterial Activity[g]	Compatibility with Human Cells[h]
Pa1$_2$Pp51								
Pa1$_{10}$Pp51								
Pa1$_{60}$Pp51								
Pa2Pp51								
Pa3Pp51								
Pa4Pp51								
Pa5Pp51								
Pa6Pp51								
Pa7Pp51								
Pa8Pp51								
Pa9Pp51								
Pa10Pp51								

[a,b]Ion count intensities of CNO^- (a) and CN^- (b) measured by ToF-SIMS. Measurements were performed on ITO-coated DMAs, except for coatings marked with an asterisk (*), which were measured on standard DMAs. Thresholds were set at >25,100 (CNO^-) and >44,100 (CN^-) for ITO-DMAs, and >1,700 (CNO^-) and >2,500 (CN^-) for standard DMAs. [c]Threshold set at median >1,500. [d]Threshold set at median >225. [e]Threshold set at median >225. [f]Threshold set at median >0.15. [g]Threshold set at median <0.7. [h]Criterion: Ki-67 positive.
